# N-Heterocyclic carbenes on copper: a dynamic and sensitive interplay between surface functionalization and etching

**DOI:** 10.1039/d6sc04254f

**Published:** 2026-08-26

**Authors:** Arne Nalop, Himadri Sekhar Sasmal, Leon Schlosser, Jens Paweletz, Alex-Cristian Tomut, Constantin G. Daniliuc, Ayan Jati, Jakob Henz, Steffen M. Lohrmann, Michael Holtkamp, Alexander Köhrer, Florian Schreiner, Uwe Karst, Bart Jan Ravoo, Nikos Doltsinis, Frank Glorius

**Affiliations:** a Institute of Organic Chemistry, University of Münster Corrensstraße 36 Münster Germany glorius@uni-muenster.de; b Institute for Solid State Theory and Center for Multiscale Theory and Computation, University of Münster Germany; c Institute of Physics, University of Münster Germany; d Institute of Inorganic and Analytical Chemistry, University of Münster Germany; e Institute of Physical Chemistry, University of Münster Germany

## Abstract

Within the expanding field of N-heterocyclic carbenes (NHCs) as surface ligands, this work investigates the etching of copper by (benz-)imidazol-2-ylidenes during surface functionalization. In order to control and distinguish between self-assembled monolayer (SAM) formation and NHC-mediated oxidative copper etching, experimental parameters were optimized. NHC design, concentration, solvent choice, temperature, visible light exposure, oxygen level, and deposition time dependencies were investigated, providing insight into the dynamic and sensitive interplay between functionalization and etching. Consistent with all experimental results, we present an etching mechanism driven by the formation of bis(NHC) Cu^I^ complexes. A previously unidentified, albeit frequently reported N 1s X-ray photoelectron spectroscopy (XPS) peak with a binding energy (BE) of 398.8(2) eV is cautiously interpreted as two NHCs bound to the same Cu^0^ adatom – the transient intermediate for metal etching. Its structure, its N 1s BE, and its high reduction potential (*E*_Cu^0^/Cu^I^_) are supported by DFT. As this work sheds new light on N 1s XPS spectra interpretation and on NHC-mediated phase transition of copper, it helps to unify seemingly conflicting literature reports.

## Abstract 2

Within the expanding field of N-heterocyclic carbenes (NHCs) as surface ligands, this work investigates the etching of copper by (benz-)imidazol-2-ylidenes during surface functionalization.

## Graphical abstract

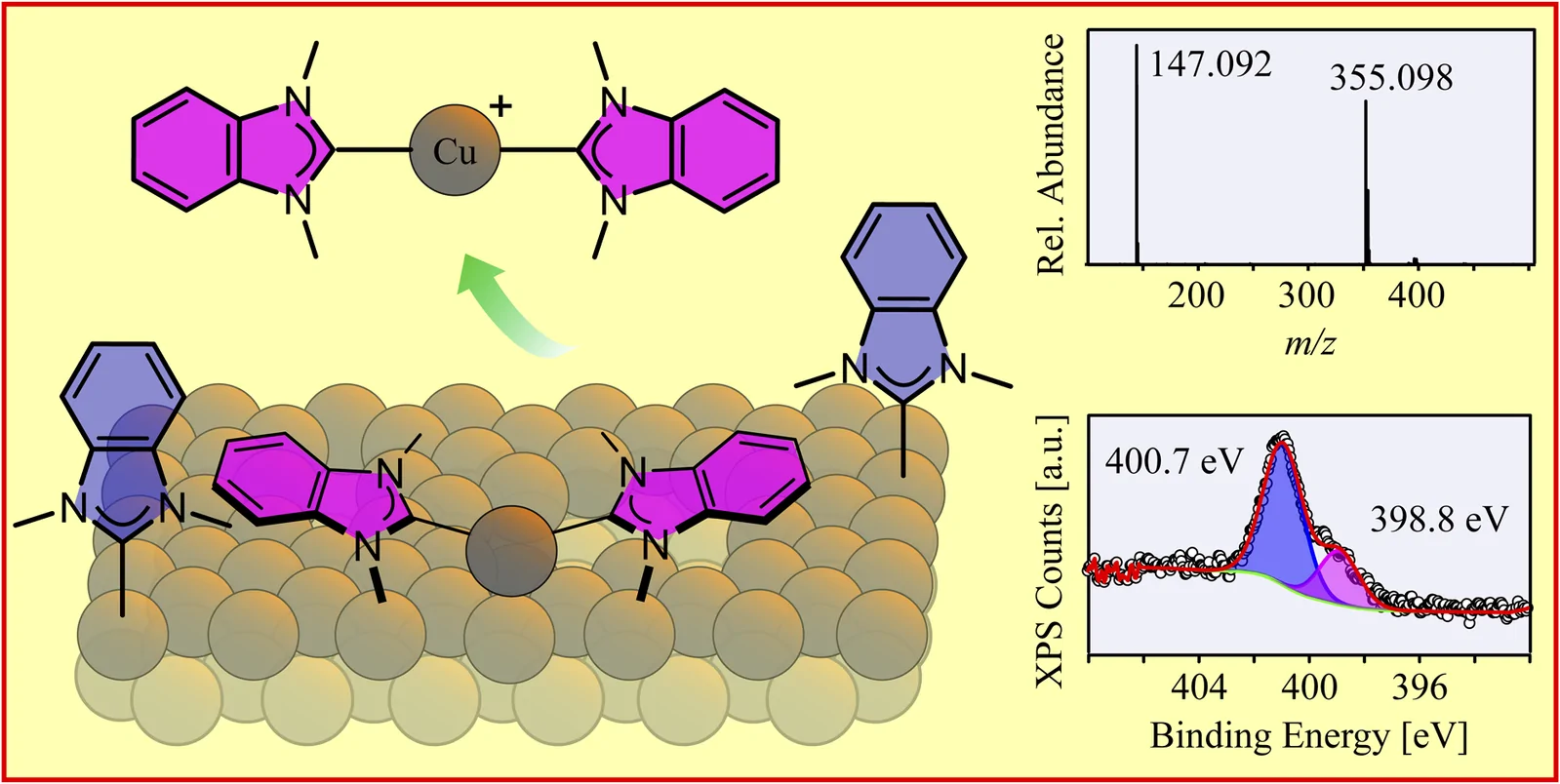

## Introduction

In addition to their great significance as ligands in homogeneous catalysis,^1–4^ N-heterocyclic carbenes (NHCs) have gained increasing attention as surface ligands.^5,6^ During the last decade, research on NHCs-on-surfaces has explored the binding affinity and geometry of different NHCs on various metals,^7–18^ metal oxides,^19,20^ and semimetals.^21–23^ Beyond this, chemisorbed NHC monolayers were tested for numerous applications ranging from nanoparticle (NP) stabilization^24–26^ and heterogeneous catalysis^27–31^ to bio-sensing,^32^ photovoltaic devices,^33^ corrosion protection^34–38^ and transistors.^39–41^

Recently, several studies observed NHCs to not only bind to metallic substrates,^42,43^ but rather to extract metal atoms from surfaces, enabling slow etching.^44–47^

In their work from 2020, Baddeley, Crudden, Horton *et al.* used benzimidazolium hydrogen carbonate solutions to reduce copper oxides and to functionalize the resultant Cu^0^ with a protective, self-assembled monolayer (SAM) of NHCs.^46^ Similarly, Tosoni, Heyde, Glorius *et al.* thermally removed Cu_*x*_O overlayers from NHC-modified copper oxide under ultra high vacuum conditions.^48^ Later in 2023, Chin, Reithofer *et al.* observed the reverse phase transition of copper-bound NHCs. Here, a soluble NHC Cu^I^ complex was reduced with NH_3_·BH_3_, forming metallic, NHC-stabilized Cu^0^-NPs.^49^ In the same year, Glorius, Ravoo *et al.* found NHCs to either accelerate or inhibit oxidative, thiourea-based etching of Au, depending on NHC design and surface coverage.^44^ Finally, by addition of an electron-withdrawing nitro group, Kiesewetter, Dwyer *et al.* designed an NHC capable of etching metallic Au without the need for oxidative additives.^45^

Strikingly, NHCs have been reported to serve as reducing, oxidizing, redox-neutral, protective, or activating agents, depending on the individual experimental setup in the respective work.

Surface functionalization and etching are two different, dynamic, yet concurrent phenomena associated with NHCs. Sometimes, these two processes compete and suppress each other during a particular application. However, no studies to date have systematically investigated the parameters controlling the dynamic interplay between these two concurrent phenomena. Whereas etching accompanied by chemical flattening and cleaning of metal surfaces may be desired in some cases,^46,48,50^ many other applications rely on the inertness of surface ligands. Especially for functionalization of precisely fabricated nanowires^51^ or thin-layer substrates,^40^ neither changes in surface morphology nor in thickness are desired.^52,53^ The same applies to exchanging the ligands of size-controlled, preformed metal NPs.^54–56^ Further, heterogeneous catalysts harnessing NHC functionalized NPs could suffer from leaching effects, as potentially formed metal NHC complexes undermine the heterogeneity of the catalyst and alter the catalytic reactivity, selectivity, and recyclability.^27^

**Fig. 1:**
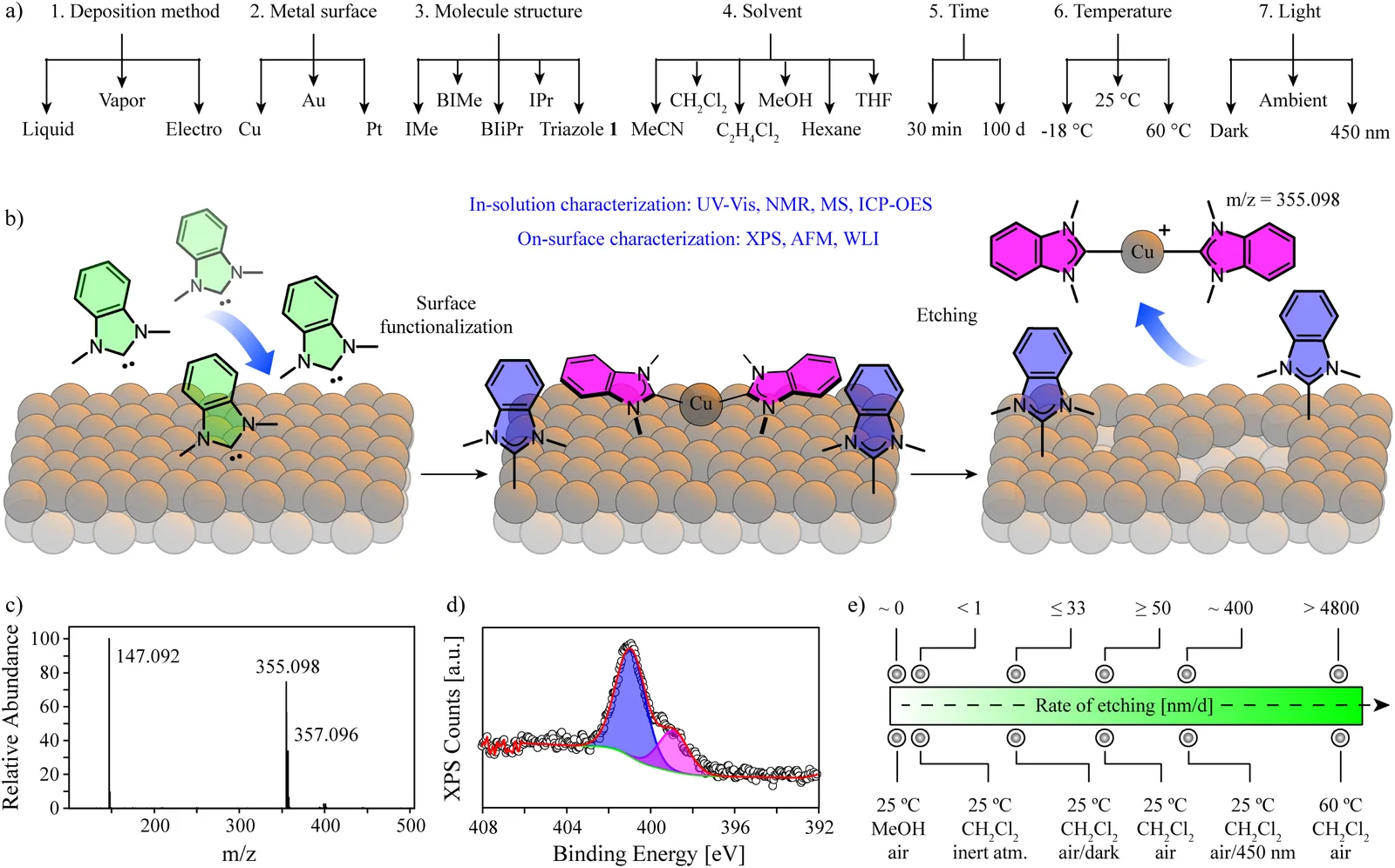
(a) Overview of experimental parameters applied for copper modification. Characterization methods are listed in blue color. IMe is 1,3-dimethylimidazol-2-ylidene, BIMe is 1,3-dimethylbenzimidazol-2-ylidene, BIiPr is 1,3-diisopropylbenzimidazol-2-ylidene, IPr is 1,3-bis(2,6-diisopropylphenyl)imidazol-2-ylidene, and triazole 1 is 2-dimethoxyphenyl-6,7-dihydropyrrolo[2,1-*c*] [1,2,4]triazol-2-ylidene. (b) Scheme of copper functionalization with BIMe and etching. (c) Exemplary mass spectrometry (MS) spectrum of the etching solution. (d) Exemplary X-ray photoelectron spectroscopy (XPS) spectrum of the functionalized surface. The peak colors blue and magenta are used to highlight the peak-to-species assignment. (e) Correlation of selected modification conditions to etching rates.

Unifying previous findings and advancing the field, this report enables control of surface functionalization and surface etching with NHCs (see Fig. 1). In this regard, modification conditions were explored and mechanistic insight into NHC-mediated phase transition of metals is provided.

## Results and discussion

### A curiosity in XPS analysis

**Fig. 2:**
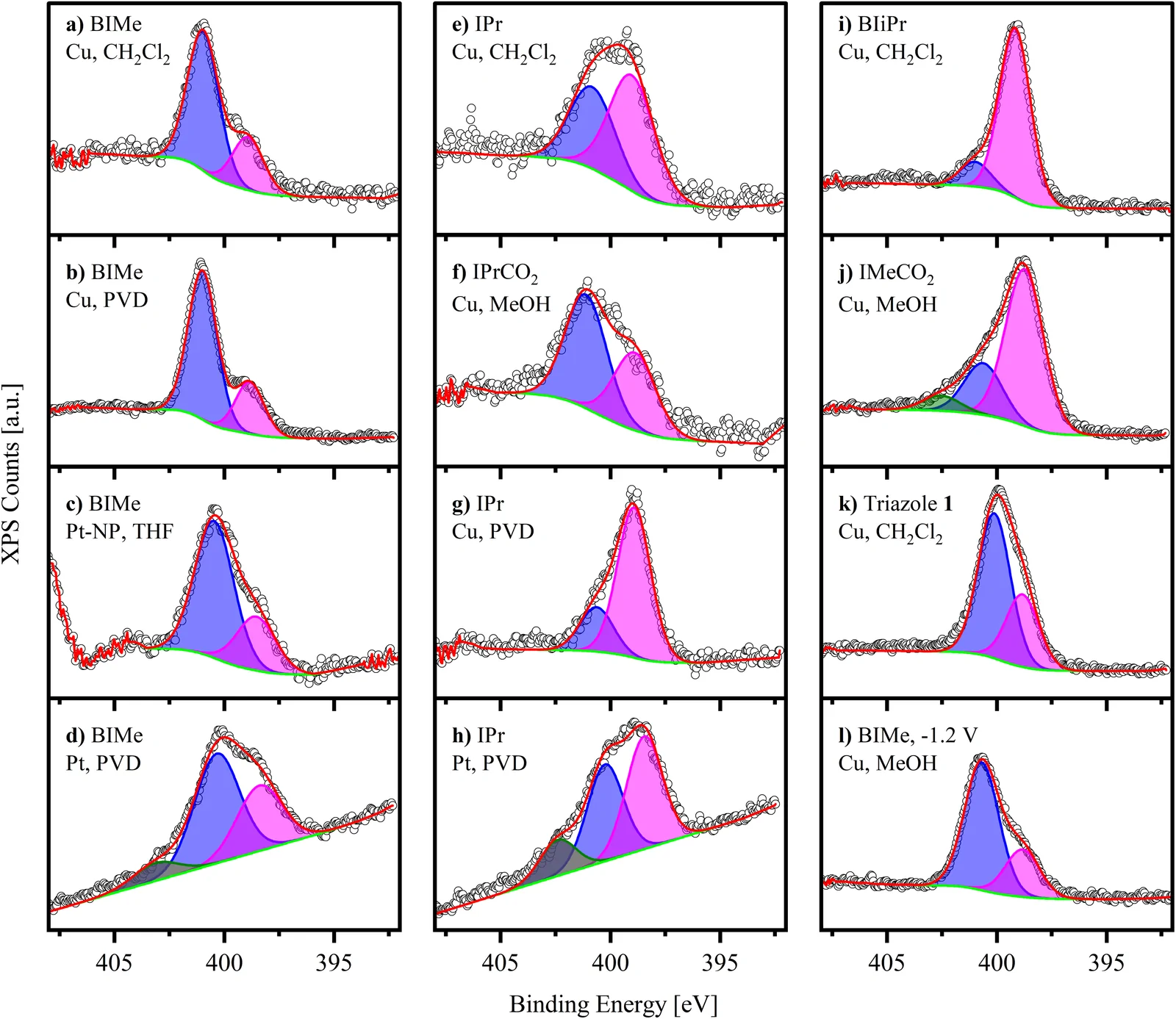
N 1s XPS spectra of five different NHCs on copper and platinum surfaces. If not otherwise noted (*e.g.*, IMeCO_2_), the imidazolium bicarbonate salt was used as NHC precursor. In case of liquid-phase deposition, the respective solvent is noted (CH_2_Cl_2_, MeOH or THF). In (b, d, g, and h) thermally activated vapor-phase deposition was used (a physical vapor deposition type process, PVD). In (l) the imidazolium salt BIMeH·I was used in electrodeposition in MeOH.^60^ Other element and survey spectra can be found in the SI, Section 4.4, including detailed individual deposition procedures.

**Fig. 3:**
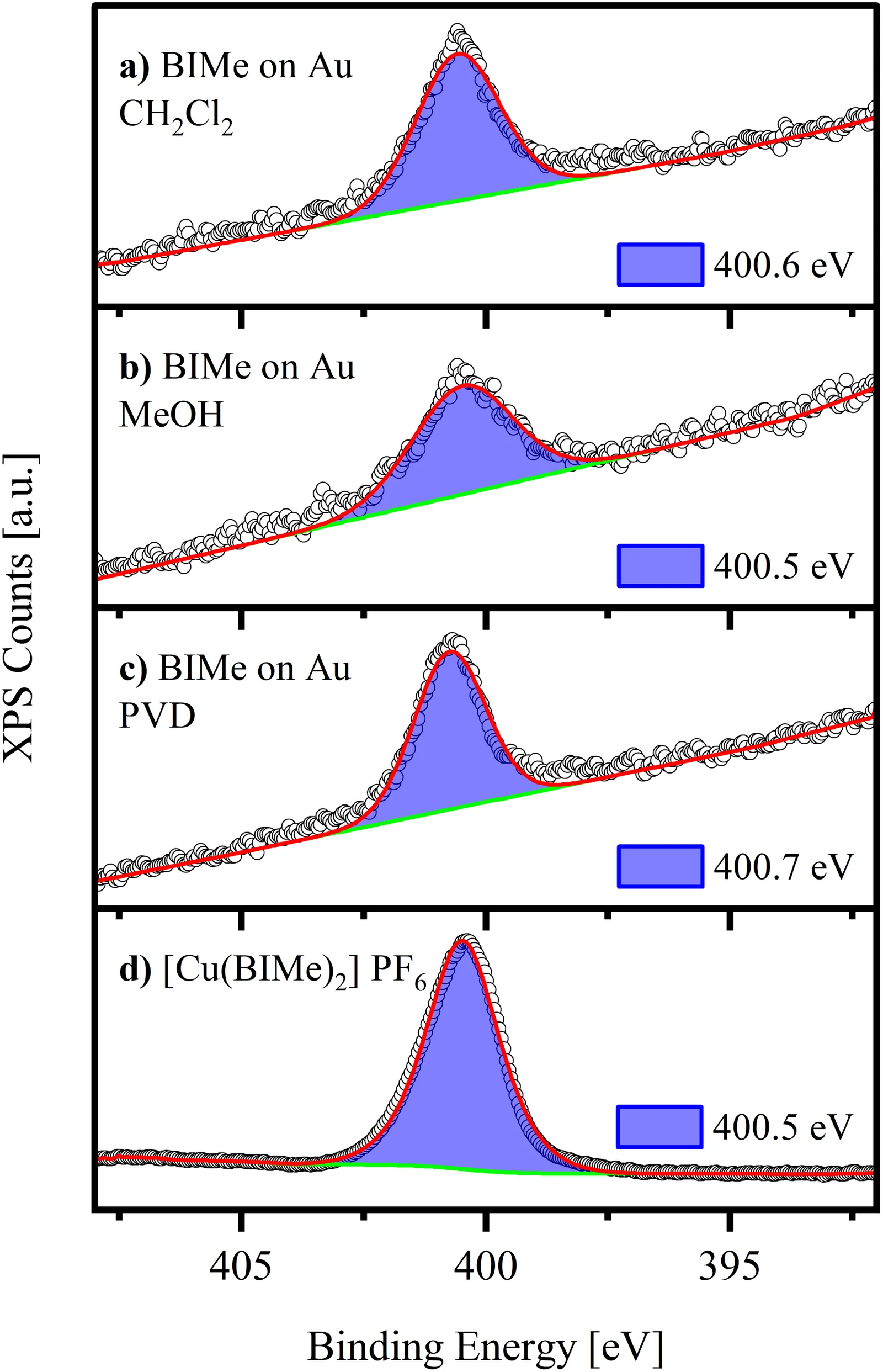
N 1s XPS spectra of BIMe on gold deposited (a) from CH_2_Cl_2_, (b) from MeOH and (c) *via* PVD. (d) N 1s XPS spectrum of [Cu(BIMe)_2_]PF_6_ powder serves as comparison. Other element and survey spectra can be found in the SI, Section 4.5, including detailed individual deposition procedures for (a–c).

When modifying copper with NHCs and analyzing the surface *via* XPS, we repeatedly observed not only the typical metal-bound NHC peak^57^ at a binding energy (BE) of around 400.7(2) eV, but also a second peak at around 398.8(2) eV. Expecting an impurity, we varied the solvent, the NHC precursor, the deposition method (wet- *versus* vapor-phase deposition), the metal substrate, and even compared flat surfaces to NPs. Still, the 398.8 eV peak was observed across a wide range of experiments (see Fig. 2a–l). As an initial control experiment, we recorded N 1s XPS spectra of the same spot twice in a row without significant change in peak ratio or signal intensity (see SI, Section 4.11). Thus, the 398.8 eV peak is not an artifact due to decomposition caused by X-ray irradiation during XPS analysis.^58,59^ As a reference, the substrate was switched to gold, and various of the aforementioned methods were tested resulting in one single, expected, well-reported NHC peak at 400.6(1) eV (see Fig. 3a–c). Also, powder XPS of bis(NHC) Cu^I^ complexes gave exclusively one peak at 400.5 eV (see Fig. 3d).

In most previous work on NHCs-on-surfaces, solely one N 1s peak around 400.7 eV was reported, as metal bound NHCs have two chemically equivalent N atoms. Only occasionally were two peaks reported for NHCs, including a variety of interpretations. In 2019, Gross *et al.* found two N 1s peaks on gold and on platinum upon modification with chiral, non-aromatic NHCs comprising a saturated backbone (imidazolidin-2-ylidenes).^61^ The peaks were found at N 1s BEs of 399.5 eV and 400.8 eV in the case of Au and 398.8 eV, and 400.5 eV in the case of Pt.^61^

Two years later, and in collaboration with our group, similar two peaks (399.5 eV and 400.8 eV) were found – this time on palladium and with aromatic imidazol-2-ylidenes.^10^ In both works, the presence of two peaks was attributed to dehydrogenation and hydrogenation of the heterocycle, respectively. No mechanism for this challenging (de-)hydrogenation was given, nor were spectra showing exclusively either of both species provided. Also, the electron-rich, reduced heterocycle imidazolidin-2-ylidene was assigned the higher N 1s BE.^10,61^

In the following years, both peaks were observed on flat copper (398.9 eV and 400.7 eV)^62^ and Cu NPs (398.5 eV and 400.7 eV)^49^ and claimed to originate from the very same surface-bound NHC molecule, but from different C–N bonds. This peak assignment is highly unusual, as N 1s core electrons are not involved in any chemical bond formation.^63–65^ Further, both nitrogen atoms in (benz-)imidazol-2-ylidene are chemically equivalent without any differences in bond order. Also, according to their N 1s peak interpretation, one would expect peak intensity ratios of 1 : 1, which conflicts with their reported spectra.^49,62^

A third explanation found in the literature is adatom formation. Scanning tunneling microscopy (STM) studies confirmed the existence of a binding mode resembling a bis(NHC) adatom complex on coinage metals.^9,57,66–69^ On Au, the adatom complex formation was connected to a shift of 0.6 eV towards lower N 1s BEs.^57^ This shift may be hard to detect *via* XPS if both species coexist and spectrally overlap. To our knowledge, for metals other than Au, no correlation between bis(NHC) adatom complex formation and N 1s BE has been reported.

Conventionally, the additional peak at 398.8 eV suggests the presence of a more electron-rich nitrogen atom compared to the classical metal-bound carbene. Both signals either originate from two chemically distinct nitrogen atoms within the same molecule due to breaking of symmetry, or from two distinct surface-bound species. The latter case includes three scenarios: (i) the 398.8 eV N 1s peak originates from an impurity (improbable due to a wide range of modification methods and independent reports) (ii) it originates from a chemically transformed (*e.g.*, reduced or decomposed); NHC derivative, or (iii) it originates from an alternative binding mode with altered M–N interaction (*e.g.*, upright *versus* flat-lying, step-edge *versus* flat surface, Cu^0^*versus* Cu^I^). One way or the other, this impurity, transformed NHC derivative, or alternative binding mode is able to compete with NHCs for binding sites during the adsorption process. This is remarkable, as NHCs are known to replace other adsorbates due to their extraordinary affinity to many transition metals.^70,71^

When checking XPS databases, it stands out that either peak could originate from a wide variety of organic compound classes showing similar N 1s BEs.^65,72^ It should be noted that observing two distinct peaks translates into having two or more chemically distinct N-species on the surface, as several species may overlap in XPS showing the same N 1s BE. Therefore, further analytical methods will be applied (*vide infra*).

### Controlling the etching

**Fig. 4:**
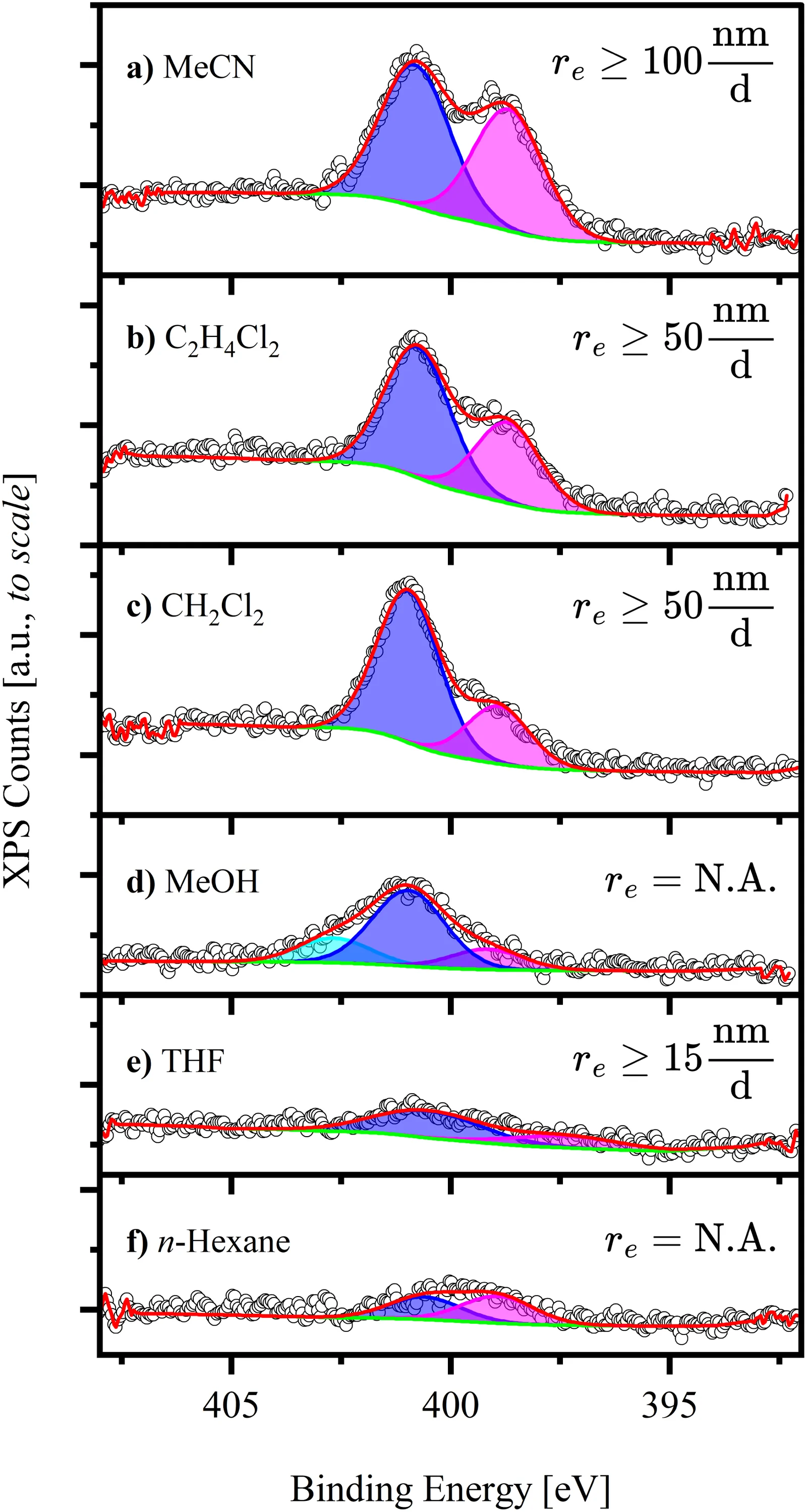
N 1s XPS spectra of BIMe on copper deposited from six different solvents with a modification time of 3 h: (a–c) from polar aprotic solvents, (d) from a protic solvent (*i.e.* methanol), and (e and f) from non-polar aprotic solvents. Other element and survey spectra can be found in the SI, Section 4.6, including detailed deposition procedures. The deposition in MeCN needs to be treated with caution, as MeCN chemisorbs on Cu in the presence of bicarbonate (see SI, Section 12).

Employing BIMe as a model NHC on copper, a solvent screen led to solvent-dependent XPS spectra and etching behavior (see Fig. 4 and S18, SI).

In nonpolar organic solvents like tetrahydrofuran (THF) and *n*-hexane, little to no surface functionalization and no etching was observed, as the NHC precursor was poorly dissolved. In polar aprotic solvents like dichloromethane (CH_2_Cl_2_), dichloroethane (C_2_H_4_Cl_2_), and acetonitrile (MeCN), XPS shows two distinct N 1s peaks after 3 h of modification, and the 100 nm thin Cu substrates were dissolved within two days of modification, yielding an etching rate of 
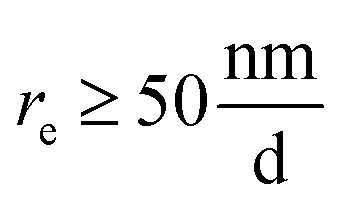
. In protic solvents like methanol (MeOH) or ethanol (EtOH), one broad N 1s peak was found after 3 h, two distinct N 1s peaks were found after 3 d (see Fig. S18) and no etching was observed within weeks.

This suggests alcohols slow down the surface functionalization, probably due to competitive O–M interactions at metallic binding sites. The attractive O–M interaction between solvent and metal is supported by doubled O 1s XPS signal intensities in the case of surface modification in MeOH for 3 h, compared to either modification in MeOH for 3 d or to modification in non-alcoholic solvents (see SI, Sections 4.6 and 4.9). These solvent–surface interactions may also cause the inhibition of Cu etching in alcohols, as will be discussed later on.

Negative control experiments without NHC precursor using various solvents, inorganic bicarbonate, or benzimidazolium iodide linked the etching to the required presence of activated NHC precursors in solution (see SI, Section 12).

**Fig. 5:**
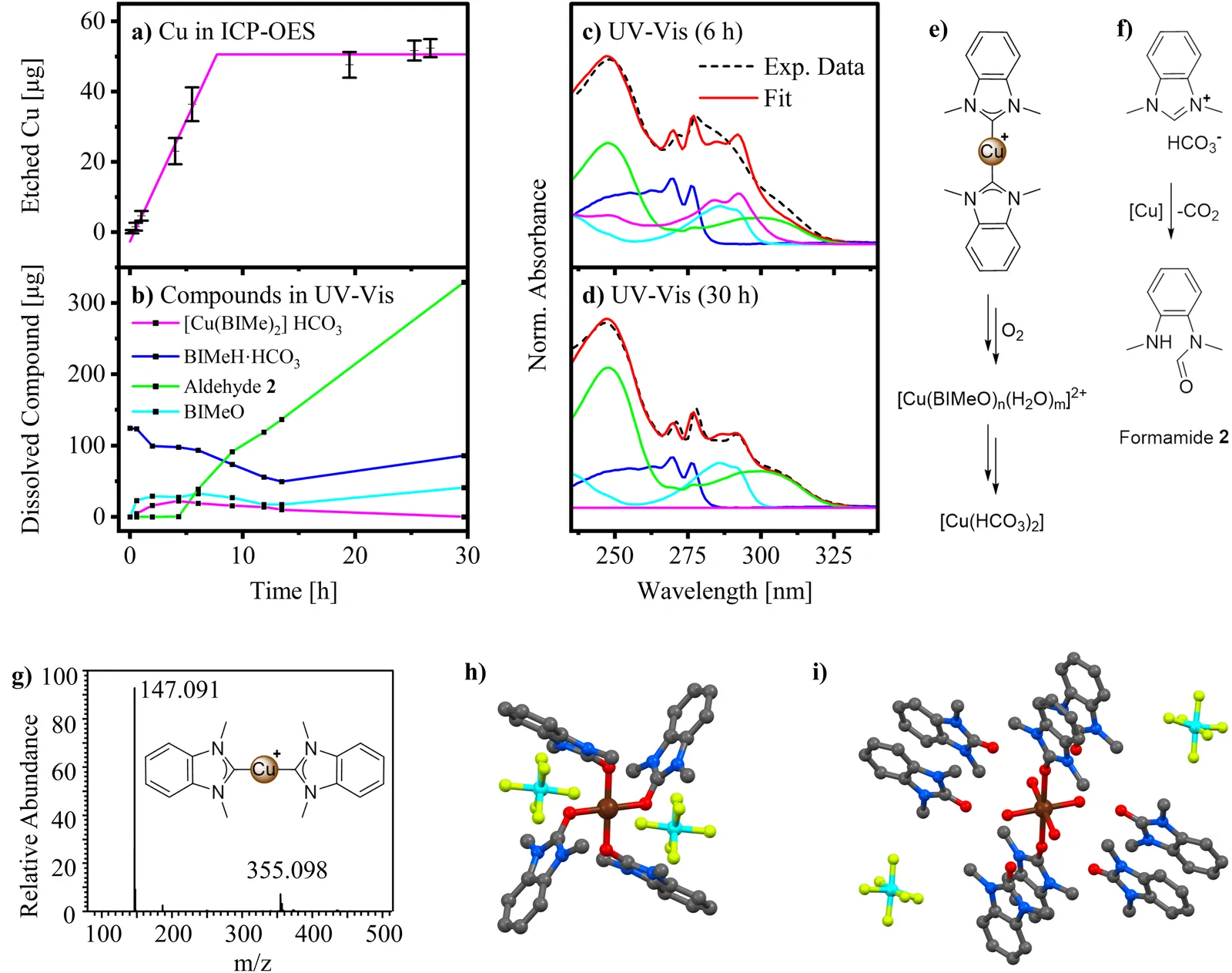
(a) The dissolved copper content determined by ICP-OES as a function of deposition time using 5 mg BIMeH·HCO_3_ in 2 ml MeCN. (b) Time dependent concentration profile of the etching solution analyzed by UV-vis. (c and d) Show exemplary linear deconvolution of the UV-vis spectra taken at a given modification time (6 and 30 h) into their component spectra. (e) Plausible [Cu(BIMe)_2_]^+^ decomposition towards BIMeO (urea analogue) and Cu^II^ salts. (f) Plausible decomposition of BIMeH·HCO_3_ towards Formamide 2 in presence of Cu ions (formal hydration of BIMe). (g) ESI-MS spectrum of the etching solution with only two dominant peaks: BIMeH^+^ and [Cu(BIMe)_2_]^+^. (h and i) X-ray crystal structures CS1 and CS2 obtained from [Cu(BIMe)_2_]PF_6_ solution upon oxidative decomposition under air. Elements are represented using the following color code: Cu brown, O red, C grey, N blue, F green, P cyan. Hydrogen atoms are not drawn for clarity.^77^

The phenomenon of etching enabled liquid-phase analysis as indirect insight into on-surface processes and its involved species. Mass spectrometry (MS, nanospray ESI) of the liquid phase revealed the presence of [Cu^I^(NHC)_2_]^+^ upon surface modification in CH_2_Cl_2_ or MeCN, whilst surface modification in MeOH did not yield any Cu–NHC complexes. Depending on the etching parameters (deposition time, temperature, and air exposure), the formed copper complexes were found to catalyze hydration of imidazolium cations to the respective formamide,^73^ and to decompose under air to the respective urea derivative (BIMeO) and copper(ii) *via* oxygen insertion (see SI, Section 12 and 14).^74–76^ Therefore, our findings partially agree with Baddeley, Crudden, Horton *et al.*’s report about the formation of [Cu(NHC)_2_]^+^, formamide and urea derivatives during etching.^46^ However, we found the pathways leading to formamide and urea derivatives to be consecutive reactions of Cu etching without any impact on the etching rate or mechanism (see Fig. 5e and f). This is supported by the observation that both pathways can be avoided by short etching times at 60 °C (see Fig. 5g and S19). Also, both pathways were found to occur in solution in the absence of any Cu^0^, when recrystallizing [Cu(BIMe)_2_]PF_6_ in the presence of benzimidazolium bicarbonate under ambient conditions. Here, [Cu^II^(Urea)_*n*_](PF_6_)_2_ crystals were isolated (see Fig. 5h and i).

After synthesis and analysis of the species found in ESI, UV-vis was utilized for quantitative analysis of the etching solution (see Fig. 5b–d). Spectral deconvolution revealed the continuous formation of [Cu(NHC)_2_]^+^ during the first few hours of the reaction, followed by hydration of excess imidazolium salt even beyond the duration of etching. Inductively coupled plasma optical emission spectrometry (ICP-OES) analysis tracked the Cu content of the etching solution as a function of time, indicating pseudo-zeroth-order kinetics thanks to precursor excess and constant Cu surface area (see Fig. 5a).

As complexes were formed, monolayer self-assembly had to be distinguished from a possible multilayer or even intercalate formation.^78^ The N 1s XPS spectra shown in Fig S18b and S19h rank among the highest N 1s peak intensities of functionalized and washed Cu surfaces reported in this work. Based on the Cu 2p and the N 1s integrals, coverages of 4.4 NHCs nm^−2^ in MeOH and 3.3 NHCs nm^−2^ in CH_2_Cl_2_ were calculated (see SI, Section 5). Within uncertainties regarding attenuation lengths,^79,80^ our coverage estimations agree with literature reports of BIMe monolayers^58,81^ as well as with our DFT simulations (see SI, Section 15). Combined with a root mean square roughness of only *S*_q_ = 3.1 nm observed *via* atomic force microscopy (AFM, see Fig. S67), the calculated coverages suggest the presence of a chemisorbed NHC (sub-)monolayer on copper and rule out multilayer or stacked island formation. Notably, the surface roughness was even lower using PVD instead of liquid-phase deposition, as observed *via* white light interferometry (WLI, *S*_q_ = 0.7 nm, see Fig. S68).

Sticking to BIMe as a model NHC on Cu in CH_2_Cl_2_, deposition time and BIMeH·HCO_3_ concentration did not have a significant influence on the peak ratio (see SI, Section 4.3). However, elongated deposition times generally resulted in sharper, more intense N 1s peaks. Also, higher BIMeH·HCO_3_ concentrations increased the etching rate. This concentration dependency, combined with functionalized surfaces being robust against organic solvents, underlines the relevance of NHC precursor excess for etching. Thus, low to medium NHC concentrations (*c* ≤ 2 mM) combined with long deposition times (overnight or longer) were found to be beneficial for surface functionalization. This observation was not surprising and agrees with common functionalization protocols applied in the literature.^42^

**Fig. 6:**
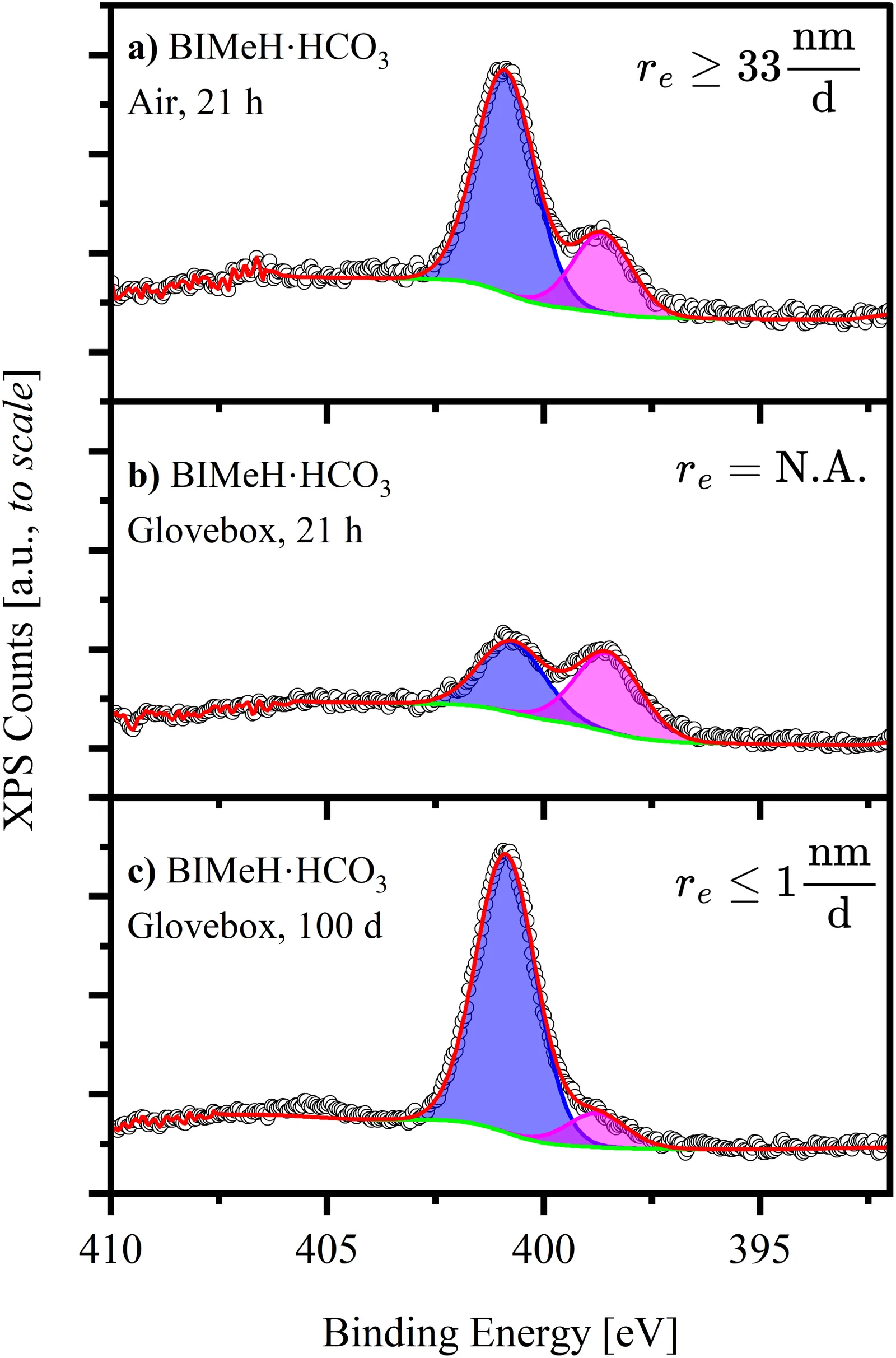
N 1s XPS spectra of BIMe on copper. (a) Cu substrate and 2 mg BIMeH·HCO_3_ in 1 ml CH_2_Cl_2_ under ambient conditions for 21 h. (b) Cu substrate and 2 mg BIMeH·HCO_3_ in 1 ml CH_2_Cl_2_ in a glovebox for 21 h. (c) Same as (b) but after 100 days of deposition time. Other element and survey spectra can be found in the SI, Section 4.7 including detailed deposition procedures.

Avoiding oxygen by working with freeze-pump-thawed solvents in a glovebox (H_2_O and O_2_ levels below 0.5 ppm), led to decreased N 1s signal intensities at a given deposition time of 21 h (see Fig. 6b). Especially the 400.7 eV intensity was weakened compared to Cu modification under ambient conditions. Supposedly, this was caused by the lack of humidity, as traces of water increase functionalization speed by improving NHC precursor solubility and activity.^42,58^ At a deposition time of 100 d in the glovebox, the peak at 400.7 eV became dominant, even suppressing the peak at 398.8 eV (see Fig. 6c). As etching was inhibited in the glovebox for at least 100 d, oxygen seems to be crucial for the oxidative Cu^0^ etching. The necessity of oxygen for this phase transfer matches a novel literature report of oxygen being crucial for NHC ligand exchange on Au NPs, which presumably includes a reversible Au^I^ complex formation.^56,82^

Furthermore, based on the N 1s peak ratio achieved under extended modification time, we conclude that the 400.7 eV peak originates from a thermodynamically favored surface functionalization state under maximization of adsorption energy (AE) per area—as expected for NHC SAM formation with upright binding configuration.

Notably, if the copper substrate had a native oxide layer due to oxygen exposure prior to its oxygen-free functionalization inside the glovebox, [Cu(NHC)_2_]^+^ ions were found *via* ESI-MS in the functionalization solution, indicating the removal of native oxides without consecutive Cu^0^ etching. This statement agrees with the removal of CuO_*x*_ features in Cu 2p spectra between 935 and 950 eV and a clear decrease in O 1s signal intensity upon NHC functionalization (see Fig. S46 and S47). The removal of native oxide upon NHC functionalization is consistent with previous work.^46–48^ As a consequence, Baddeley, Crudden, Horton *et al.* reported NHCs to reduce Cu oxides and to form a protective monolayer on Cu^0^.^46^ However, no evidence of CuO_*x*_ reduction was observed throughout our work. In contrast, NHCs were observed to activate copper for aerial oxidation and to complex the formed Cu^I^ and Cu^II^ species, since the etching of metallic Cu was clearly caused by the presence of NHCs (see Fig. 5 and SI, Section 12). We consequently propose the following equations to describe NHCs dissolving copper oxides and one redox equation to describe the Cu^0^ etching:

1 2(BIMeH^+^HCO_3_^−^) + Cu^II^O → 2BIMe + H_2_O + Cu^II^(HCO_3_)_2_

2 4(BIMeH^+^HCO_3_^−^) + Cu^I^_2_O → 2[Cu^I^(BIMe)_2_]^+^ + 2HCO_3_^−^ + 3H_2_O + 2CO_2_

3
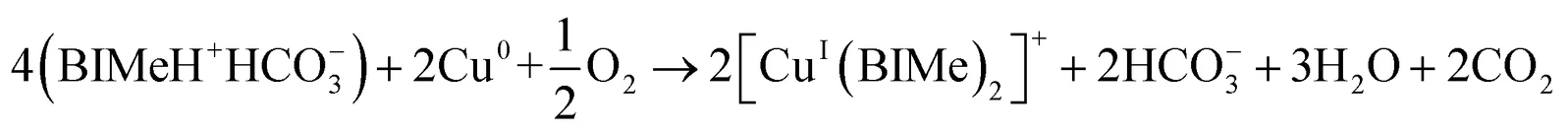


Underlining the oxidative character of the Cu etching, cyclic voltammetry (CV) experiments (see SI, Section 10) showed the following: (i) Negative voltages are beneficial for copper surface functionalization.^35,43,83^ (ii) Positive voltages lead to a loss of NHC monolayer due to etching. (iii) External voltages do not influence the N 1s peak ratio. (iv) The oxidation potential needed to dissolve copper decreases in the presence of dissolved NHC precursors. Consequently, a potential interconversion between the two species responsible for the 398.8 eV and the 400.7 eV N 1s peak, respectively, proceeds redox-neutral. Also, the finding of NHCs activating metallic copper for oxidation agrees with previous observations of BIMe accelerating thiourea-based gold etching at low SAM coverage and inhibiting the same process at high SAM coverage.^44^ Whereas addition of a chemical reductant neither influenced the N 1s peak ratio nor signal intensity, treatment of a functionalized surface with a chemical oxidant decreased the overall signal intensity. Further, adding the oxidant during functionalization, the signal at lower N 1s BEs vanished in favor of the signal at higher N 1s BEs (see SI, Section 10).

Moving from room temperature to 60 °C increased the etching rate *r*_e_ of IMe and BIMe on Cu in CH_2_Cl_2_ by two orders of magnitude (see Fig. S19). Remarkably, not only the etching, but also the functionalization was accelerated by elevated temperature. This acceleration enables copper functionalization with NHCs within several minutes. Both the solubility and the activity of the NHC precursor are expected to benefit from heat. *Vice versa*, cooling the process to −18 °C was found to slow down functionalization without any sign of etching for at least two weeks (see SI, Section 4.2). Not only heat, but also light exposure was found to influence this sensitive system of liquid-phase functionalization and etching: irradiation accelerates etching, whilst darkening slows down etching. This way, light exclusion enables elongated deposition times without slowing down the functionalization. The enhanced deposition time in the dark shifts the peak ratio in favor of the 400.7 eV N 1s peak (see Fig. S19h). This observation reinforces the results of the glovebox experiments discussed above. In both cases, etching is slowed down or even inhibited, resulting in a predominant 400.7 eV N 1s XPS signal at elongated deposition times.

**Fig. 7:**
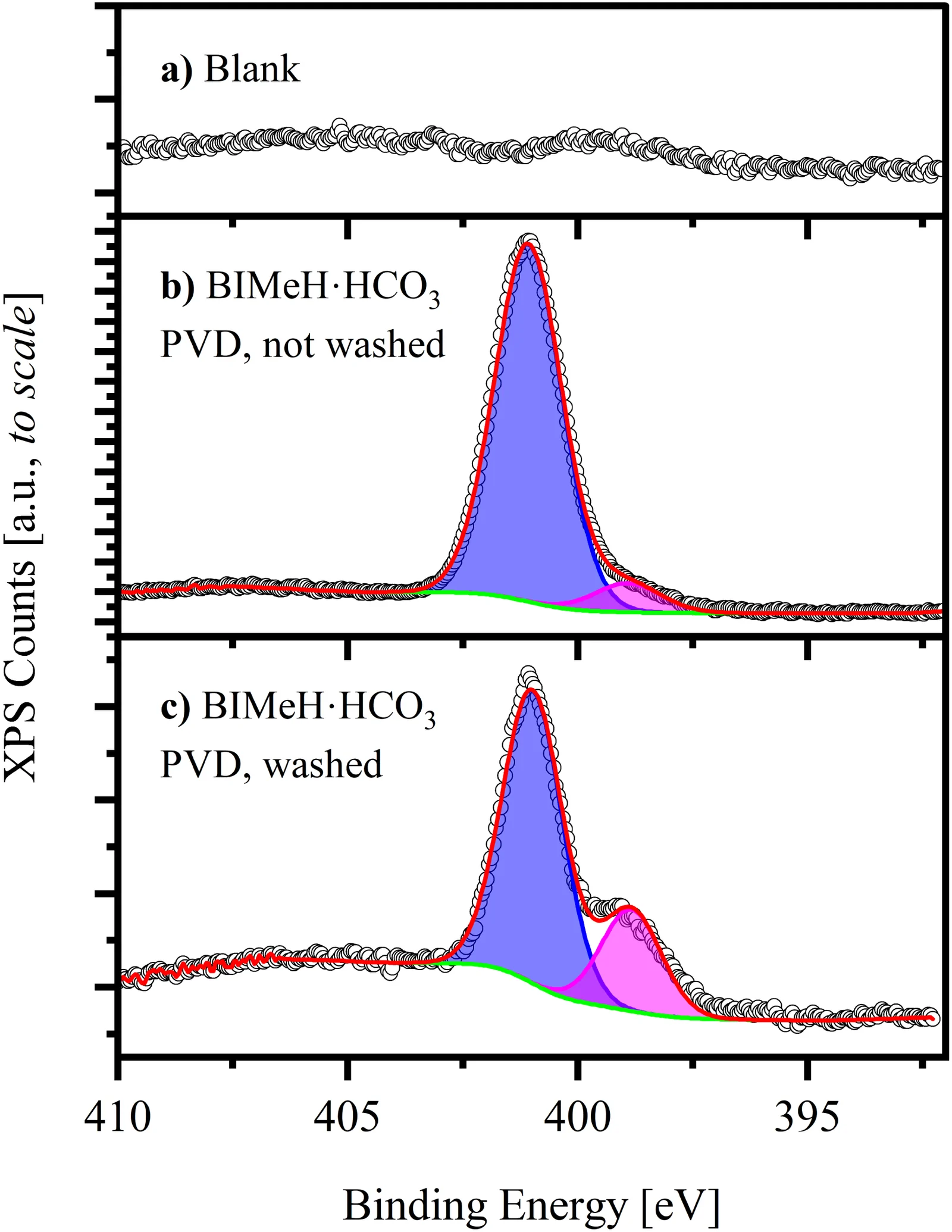
N 1s XPS spectra of copper surfaces (a) before and (b) after PVD using BIMeH·HCO_3_ as NHC precursor. (c) Shows the same surface as (b) but with an additional washing step after PVD. Other element and survey spectra can be found in the SI, Section 4.8, including deposition procedures.

Based on our temperature-dependent findings, the results of PVD should be revisited. As PVD requires heat (around 100 °C), and as our PVD chamber works at low but still significant O_2_-partial pressures of around 1 mbar, one could expect signs of Cu^0^ → Cu^I^ oxidation. Upon elongated deposition time (1 h instead of 15 min), the vapor-phase-modified substrates were analyzed before and after washing with organic solvents. Without any washing, the N 1s XPS signal surpassed typical monolayer intensities and other N to Cu ratios presented in this work by far (see Fig. 7b and SI, Section 4.8). After washing, two N 1s signals were found with typical peak ratio and intensities as known from NHC monolayers presented in this work (see Fig. 7c). This suggests the NHC monolayer gets covered underneath a washable physisorbate^78^ during PVD, whose N 1s BE overlaps with chemisorbed NHCs at around 400.7 eV. Analysis of the washing solution revealed the formation of [Cu(BIMe)_2_]^+^ complexes (see Fig. S99), which agrees with the powder XPS of the same compound (see Fig. 3d) and therefore confirms the Cu^0^ → Cu^I^ oxidation even during PVD.

Finally, the effects of molecular NHC design were studied. Switching from BIMeH·HCO_3_ to IMe·CO_2_ as precursor showed no clear influence on etching rate and surface functionalization and therefore extended our observations to imidazoles and to CO_2_ adducts. When increasing the steric bulk of the wingtip group at the heterocycle, the etching rate decreased stepwise. Specifically, it decreased by a factor of two from methyl in BIMe to iso-propyl in BIiPr, and by a factor of ten to diisopropylphenyl in IPr (see SI, Section 12.4). Bulky wingtip design therefore offers a convenient handle to suppress etching if desired.

### Unifying mechanism

**Fig. 8:**
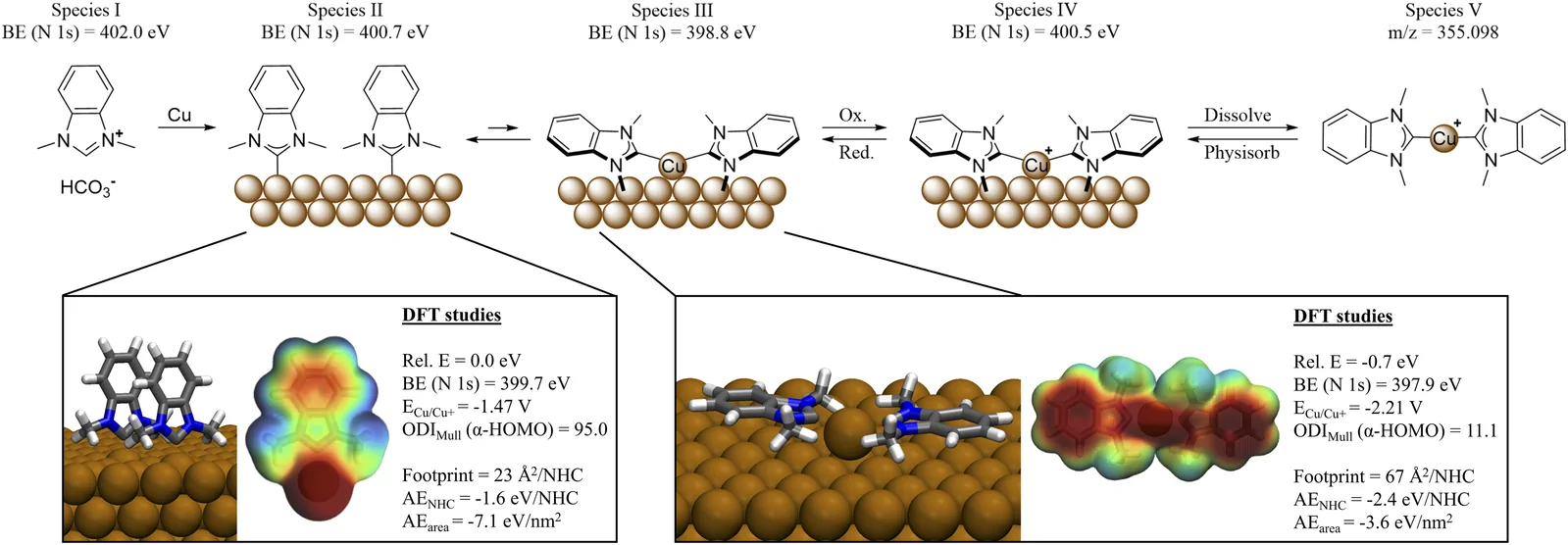
Proposed mechanism for Cu etching mediated by NHCs. All five species are observed experimentally. DFT results (see SI, Section 15) of species II and III are depicted: optimized on-surface binding geometries, electrostatic potential maps (EPM, red depicts electron-rich and blue depicts electron-poor regions), relative energies of both geometries (Rel. E), theoretical N 1s BEs, redox potentials (*E*_Cu^0^/Cu^I^_), orbital delocalization indices (ODI_Mull_, following Multiwfn, see Table S11) according to Mulliken population analysis, molecular footprints and adsorption energies per NHC (AE_NHC_) and per surface area (AE_area_).

Based on the entirety of our experiments, we propose the following Cu^0^ etching mechanism (see Fig. 8) consisting of four steps: surface functionalization with NHCs, on-surface bis(NHC) Cu^0^ complex formation, its oxidation to physisorbed Cu^I^ complexes, and finally its desorption and solvation.

The initial Cu functionalization is governed by precursor solubility and competition over binding sites. Non-polar solvents inhibit functionalization as the precursor salt precipitates. In polar aprotic solvents (CH_2_Cl_2_, C_2_H_4_Cl_2_, MeCN), which still resemble precursor suspensions due to partial solubility, the functionalization rate is enhanced. Alcohols fully dissolve the precursor but compete with NHCs for binding sites on the oxyphilic copper surface. This competition slows down the SAM formation from hours (CH_2_Cl_2_) to days (MeOH). Heating, in turn, speeds up the surface functionalization by increasing the precursor solubility, activating the precursor thermally, and driving the binding site competition towards the thermodynamically favored NHC chemisorption. The chemisorbed NHCs (here called species II) are observed at a BE of 400.7(2) eV in N 1s XPS spectra. For comparison, a powder sample of imidazolium salt appears at BEs as high as 402.5(2) eV, since the formal coordination of the NHC to a proton significantly lowers the electron density within the heterocycle. In other words, species II donates less electron density to the neutral copper surface^84^ than species I to the hard Lewis acid H^+^.

As a second step, we cautiously propose the formation of neutral bis(NHC) adatom complexes and assign them the observed N 1s BE of 398.8 eV. Based on the data, we are confident that the 398.8 eV N 1s peak corresponds to a transient, sterically demanding, electron-rich species activated for Cu^0^ oxidation due to its low N 1s BE. The transient character and the steric demand of species III are supported by experiments at elongated deposition times under inert conditions, which favor species II at the cost of species III. Also, recent STM studies^9,57,66–68^ reported the formation of bis(NHC) adatom complexes on coinage metals. On Au, this binding geometry was connected to a shift of 0.6 eV towards lower N 1s BEs compared to an upright binding geometry.^57^ The N 1s BE shift of 0.6 eV on Au could possibly be overlooked as signal broadening depending on the XPS resolution (see Fig. 3). The experimentally observed, larger shift towards lower BEs in the case of bis(NHC) adatom complexes on copper is supported by simulated N 1s BEs (1.8 eV, see SI, Section 15). The lower BE of species III compared to species II translates into a higher electron density at the nitrogen atoms. The increased local electron density is reflected in computed EPMs as well as natural population analysis (NPA). The ease of oxidation of species III compared to the upright-bound species II is further supported by DFT calculations yielding a redox potential shift of more than −0.7 V between both geometries (see Fig. S102). Based on its relative energy (Rel. E) and its high AE per NHC, the formation of the flat-lying geometry is energetically favored, enabling the etching sequence. However, thanks to a smaller molecular footprint on the surface, species II shows the higher AE per surface area and is therefore expected to predominate at high monolayer coverages, as experimentally observed under elongated modification times under inert conditions.

In addition, the electrostatic potential map (EPM, Fig. S103), natural population analysis (NPA, Table S10), and unrestricted Mulliken analysis (Table S11) consistently indicate a high degree of electron delocalization across the bis(NHC) adatom complex. These findings align with a literature report on a neutral mononuclear copper complex stabilized by two cyclic (alkyl)(amino) carbenes (CAACs), [Cu(CAAC)_2_].^85^ In that report, the formal Cu^0^ oxidation state was described as having a d^10^ configuration (*i.e.*, Cu^I^-like), with the unpaired electron being delocalized over both ligands, showing high carbene carbon and little copper participation in the singly occupied molecular orbital (SOMO).^85^ This matches our Mulliken orbital composition analysis of the α-HOMO (Table S11).

Thirdly, oxidation of the activated Cu^0^ adatoms was observed upon PVD, forming physisorbed [Cu(NHC)_2_]^+^ complexes (species IV). The charged complexes can be easily rinsed off the surface using polar organic solvents. In liquid-phase deposition, species IV was not observed, as the aerial oxidation is directly followed by desorption and solvation of the cationic complex (species V). Remarkably, the absence of oxygen in polar aprotic solvents as well as working under ambient conditions in alcoholic solvents avoid any aerial oxidation towards species IV. Presumably, protic alcohols deactivate species III upon coordination. Contrarily, elevated temperatures, additional oxidants, and application of positive voltages promote formation of species IV and its subsequent desorption. Light was also found to play a role in this step—possibly due to photo-excited electron–hole separation. The desorbed species V was detected with ESI-MS and UV/Vis and was synthesized to successfully match its powder XPS with the physisorbed species IV.

## Conclusions

Exploring the novel phenomenon of NHCs etching Cu^0^ substrates, this work establishes a unified mechanistic framework with experimental control, enabling either the formation of a dense NHC SAM or NHC-mediated copper etching.

SAM formation with minimized or even avoided etching can be achieved by working in alcoholic solvents or by working under oxygen-free conditions, under exclusion of light, by cooling, by working with low NHC concentrations (*e.g.*, *c* ≤ 1 mM) at elongated deposition times, or by using sterically demanding NHC wingtip groups. This knowledge about controlling phase transition in the presence of coordinating NHC ligands is crucial for thin-layer substrate functionalization as well as for heterogeneous catalysis.

In turn, NHC-mediated Cu etching can be easily achieved by working in polar aprotic solvents, gentle heating, and by working with small, sterically non-demanding or even electron-deficient NHCs.

Harnessing our experimental insight into the dynamic and sensitive interplay between functionalization and etching, we present an etching mechanism driven by the formation of bis(NHC) Cu^I^ complexes [Cu(NHC)_2_]^+^. A previously unidentified, albeit frequently reported N 1s XPS peak with a binding energy of 398.8(2) eV is cautiously interpreted as two NHCs bound to the same Cu^0^ adatom. In accordance with DFT simulations, this adatom complex represents the transient intermediate for metal etching. Here, the NHCs activate the Cu^0^ adatom for oxidation towards physisorbed [Cu(NHC)_2_]^+^, which is easily desorbed and dissolved. Matching ultra-high-vacuum STM studies,^57^ we report the controlled formation of bis(NHC) adatom complexes on a metal surface under ambient conditions, which could prove valuable for the design of heterogeneous catalysts.^82^

## Author contributions

All authors have approved the final version of the manuscript. A. N. and F. G. conceived the project. A. N. and H. S. S. carried out chemical synthesis, surface modifications and XPS measurements. L. S. carried out and automated data analysis. J. P. acquired MS data and optimized MS methods. A. C. T. and N. D. carried out theoretical calculations. C. G. D. carried out single-crystal X-ray diffraction data collection and structure refinement. L. S., A. J. and B. J. R. contributed to writing the manuscript (review and editing). J. H. carried out WLI measurements. S. M. L. carried out AFM measurements. M. H., A. K. and U. K. carried out ICP-OES measurements. F. S. carried out EPR measurements. A. N., H. S. S. and F. G. prepared the first draft of the manuscript. F. G. supervised the project.

## Conflicts of interest

There are no conflicts to declare.

## Supplementary Material

SC-OLF-D6SC04254F-s001

SC-OLF-D6SC04254F-s002

SC-OLF-D6SC04254F-s003

## Data Availability

CCDC 2532664 and 2532665 contain the supplementary crystallographic data for this paper.^86*a,b*^ The authors declare that the data supporting the findings of this study are available within the paper and the supplementary information (SI), as well as from the authors upon request. Supplementary information is available. See DOI: https://doi.org/10.1039/d6sc04254f.
