# Supplementary material for: N-Heterocyclic carbenes on copper: a dynamic and sensitive interplay between surface functionalization and etching
Source: Chem Sci. 2026 Aug 26;17(41):19838–48. doi: 10.1039/d6sc04254f (PMC13539560; doi:10.1039/d6sc04254f)
Supplement: Supplementary file 1 [file SC-017-D6SC04254F-s001.pdf]

This journal is © The Royal Society of Chemistry 2026

VASP optimized coordinates:
Species from **Figure S97 a)**

```
234
Generated with con2xyz
Cu   10.22973634280144   0.67131586652722   5.08728658449724
Cu   1.28348144762368   8.80137133087048   0.90370000000011
Cu   1.28568227792491   10.27631524173066   3.01640541105490
Cu   10.22981644228912   2.15983268780656   0.90370000000011
Cu   10.25367713906027   3.63121665598981   3.05766441944757
Cu   8.94294027149689   2.89699992853354   5.05772052873164
Cu   0.00653585769462   9.53560796468316   5.12239500734852
Cu   1.28495118830118   2.89701725840631   5.13073104414892
Cu   8.95154948948265   4.37350180863156   0.90265000000002
Cu   8.96156322821266   5.87197426168445   3.06773326229103
Cu   7.67411529528458   5.09406642094461   5.07392863737401
Cu   -5.10580841645604   15.44273288040492   0.90125000000008
Cu   0.00582796479926   11.01539463875492   0.90335000000000
Cu   7.67348702667015   6.58787930357520   0.90335000000000
Cu   7.70340403776502   8.04977449517052   3.06762787313566
Cu   6.39625500860128   7.32431556874938   5.08018112161294
Cu   0.00703805924353   12.48982868842775   3.01572412246952
Cu   -1.27400508295449   11.75151616846587   5.12195880203463
Cu   6.39562905385170   8.80119423734088   0.90335000000000
Cu   6.40211566103832   10.26884138580457   3.02236275917359
Cu   5.12496256267352   9.53157818956718   5.13183798733175
Cu   -5.10733917289367   16.91690257963065   3.01358261490843
Cu   -6.38440334125068   16.17833324154532   5.12870344962460
Cu   5.11715761105111   11.01486335816588   0.90265000000002
Cu   5.12147031411723   12.48752608406211   3.01684566327710
Cu   3.84442516413683   11.74942305818474   5.13250049342506
Cu   -1.27243898800736   13.22906375957992   0.90370000000011
Cu   -1.27134193087295   14.70164206510444   3.01351164584996
Cu   3.83889065824457   13.22888666605020   0.90370000000011
Cu   3.84080700697012   14.70366200881822   3.01778603124254
Cu   2.56466830188514   13.96416232439691   5.13266674919369
Cu   -2.55232204668666   13.96360096189153   5.12243044067647
Cu   2.56093044042913   15.44237869334548   0.90404999999997
Cu   2.55861706002907   16.92149793835652   3.02053493774517
Cu   1.28373594489880   16.17983061508311   5.13056639059167
Cu   -2.55019471582868   15.44255578687520   0.90370000000011
Cu   1.28307246761067   17.65569362711116   0.90404999999997
Cu   11.51272295283955   1.45425010280318   3.07522347250431
Cu   12.78754327152880   0.68233064198616   5.13925413404534
Cu   -2.54932932976529   16.91012821624843   3.00302839763375
Cu   -3.83062150567348   16.17827666763858   5.12630867479039
Cu   12.78563463291059   2.15965559427684   0.90370000000011
Cu   12.76470440601140   3.62235763219273   3.04489897341414
Cu   11.50768816772866   2.88599702500076   5.71639567977101
Cu   1.28317471264508   4.37385599569088   0.90544999999992
Cu   11.50757217009817   4.37367890216116   0.90125000000008
Cu   11.49838461883963   5.84220663907703   2.99663936397280
Cu   10.23868755901768   5.10341768041652   5.13821068570160
Cu   -3.82825717864111   17.65657909475952   0.90335000000000
Cu   6.39331227577935   1.42155033840939   3.00033706635323
Cu   10.22899848230045   6.58929605181247   0.90335000000000
Cu   10.20732987325493   8.05184160076602   3.05142944435683
```

Cu	8.96108876223756	7.29726955096644	5.74373569355396
Cu	7.66442352756754	0.67400376089295	5.08592450578078
Cu	1.28340303115435	5.84675293168321	3.01609719996102
Cu	8.95134499947611	8.80119423734088	0.90440000000008
Cu	8.94373184083330	10.27528094225507	3.01568474400744
Cu	7.68316893503693	9.52380605889392	5.14619829680330
Cu	0.00477317995854	5.10987437597340	5.12994664720891
Cu	7.67379376167375	2.16000978133616	0.90370000000011
Cu	7.67502070161293	11.01362370345820	0.90404999999997
Cu	7.67148410629666	12.48589213992396	3.01897712829482
Cu	6.39809054165325	11.74508349909365	5.13771871145437
Cu	7.67613586630467	3.63640561940760	2.98583513830558
Cu	6.38928803165711	2.89109139337575	5.10856846877277
Cu	6.39501558385700	13.22853247899088	0.90055000000010
Cu	6.39553797902381	14.70095888603574	3.01457645534396
Cu	5.11889918944827	13.96132871715024	5.13206126216744
Cu	-3.82917738360190	13.22870957252048	0.90195000000005
Cu	-3.83182348650360	14.70283869088506	3.01433809775943
Cu	5.11674863105053	15.44220159981588	0.90370000000011
Cu	5.11423706173026	16.91329346154973	3.01935509336412
Cu	3.83963845413899	16.17564877782106	5.13729665754150
Cu	6.39583354385823	4.37350180863156	0.90404999999997
Cu	6.40124744742495	5.85131638748069	2.99772375263630
Cu	3.83940188321714	17.65569362711116	0.90370000000011
Cu	14.06015383001809	1.41039630488508	3.00617706464700
Cu	15.33854251317532	0.68065420799076	5.13124229186236
Cu	5.11035946175093	5.10157275311060	5.08641807466639
Cu	0.00531673982669	6.58752511651588	0.90370000000011
Cu	15.34196404851714	2.15930140721752	0.90299999999988
Cu	15.33852867865144	3.63377753333967	2.99391891752222
Cu	14.06428750915371	2.88236122567099	5.06490282229617
Cu	2.56322946161833	0.68350770660710	5.12939273186559
Cu	5.11664638607854	6.58699383592684	0.90229999999991
Cu	14.06349260571656	4.37226215392388	0.90089999999996
Cu	14.06180216372667	5.84731348928190	2.99069317536541
Cu	12.78081170437918	5.11728163876211	5.06957784293965
Cu	5.12120390837829	8.06461266864344	3.00219407030339
Cu	3.84262769706319	7.32099354616629	5.11757597507063
Cu	12.78543014290405	6.58982733240151	0.90159999999994
Cu	12.78527230912565	8.06126514726949	2.99248927381718
Cu	11.51283506218079	7.30938683297406	5.05840209673240
Cu	-6.38468883924485	17.65640200122992	0.90335000000000
Cu	3.83692323406308	1.41932851427783	3.01465277173247
Cu	11.50695870010339	8.80491320146391	0.90825000000006
Cu	11.50211555356373	10.27181560806785	3.00697369127509
Cu	10.22012797556006	9.53585829943891	5.09571310894561
Cu	3.83919739324809	8.80172551792992	0.90404999999997
Cu	3.84489566127484	10.27692060191224	3.01660714768072
Cu	10.23247481218681	11.01238404875052	0.90895000000003
Cu	10.22733986126108	12.48582825574090	3.01632571272943
Cu	8.94987457133336	11.74344723681972	5.13564681856651
Cu	2.56682097483000	9.53610239466270	5.12565872965627
Cu	5.11700944457077	0.68130938491946	5.12769639704876
Cu	8.95277642942183	13.22782410487224	0.90229999999991
Cu	8.94891791766529	14.70057307412338	3.01295189742663
Cu	7.67150278663849	13.96100106186488	5.13077714163677
Cu	-3.83258821368188	11.74872927230191	5.12851096496599

Cu	2.56113493043560	11.01504045169548	0.90404999999997
Cu	7.67205559668686	15.44290997393452	0.90159999999994
Cu	7.67002711053177	16.91515421154686	3.01480898948899
Cu	6.39332754559433	16.17467855753725	5.13101962192952
Cu	2.56622296132313	12.49070143563499	3.01631901553803
Cu	1.28800338636299	11.75186566792159	5.12221508356140
Cu	6.39511782884163	17.65604781417048	0.90404999999997
Cu	16.61598185282914	1.42071649433575	3.01367428394877
Cu	17.89326338869094	0.68205065109919	5.12223944393324
Cu	2.56123717545758	2.16018687486588	0.90404999999997
Cu	-5.10910417461707	13.96336586292545	5.12807228592067
Cu	17.89808897412964	2.16000978133616	0.90404999999997
Cu	17.89727915874970	3.63564696607625	3.01328180319480
Cu	16.61636281299347	2.89834517301764	5.11974910961706
Cu	1.28358369260824	13.22888666605020	0.90335000000000
Cu	1.28713658798884	14.70404533842120	3.01638208974046
Cu	16.61992426632008	4.37314762157224	0.90335000000000
Cu	16.62577055750518	5.85218564049274	3.00016940744589
Cu	15.34433829467768	5.11326906334032	5.07912031846857
Cu	0.00697418819252	13.96557529255941	5.12516140560686
Cu	2.56360184189376	5.10852208418984	5.12769434696459
Cu	15.34237302849272	6.58734802298616	0.90019999999999
Cu	15.34469955526917	8.06297980846119	3.00647108278544
Cu	14.06946630307133	7.32394933784363	5.11012374344511
Cu	0.00516991782868	8.06064740520926	3.01611886595438
Cu	0.00552122979574	15.44255578687520	0.90370000000011
Cu	14.06400383068912	8.80084005028156	0.90159999999994
Cu	14.06828319085484	10.27826476700392	3.00356293323004
Cu	12.78894895066729	9.53741873983873	5.08619678248285
Cu	0.01026378804915	16.91624842877064	3.00381215226705
Cu	-1.27406471728344	16.17747647376347	5.12097178858466
Cu	12.78174932299860	11.01220695522093	0.90789999999995
Cu	12.78403594818209	12.48706595364928	3.01517949552454
Cu	11.50662235667912	11.74400170415131	5.12833334395356
Cu	5.11756659106419	2.16018687486588	0.90370000000011
Cu	11.50777666006714	13.22835538546116	0.90195000000005
Cu	11.50560696094608	14.70138591427050	3.01296384869798
Cu	10.22674485567173	13.95996172551247	5.12223356541078
Cu	-1.27274572301080	17.65587072064088	0.90335000000000
Cu	8.95804234561087	1.41999602606082	2.99583091025210
Cu	10.22910072727251	15.44273288040492	0.90125000000008
Cu	10.22715669834670	16.91561038805009	3.01498992289315
Cu	8.94705244031759	16.17473191846465	5.12348833071819
Cu	8.95062928447193	17.65604781417048	0.90370000000011
Cu	19.17356509641510	1.42080244024585	3.01513390210942
Cu	0.00293584380413	0.68362442705384	5.12236182708645
Cu	-1.27501087643786	7.32209665491987	5.12940079382328
Cu	0.00511224984508	2.16018687486588	0.90404999999997
Cu	0.00576052452628	3.63370173681350	3.01805792940198
Cu	-1.27538719687275	2.89766038553317	5.12334510722429
Cu	2.56133942044221	6.58770221004548	0.90370000000011
Cu	2.56176576966200	8.06368553290084	3.01275907905398
Cu	-1.27284796797029	4.37403308922048	0.90370000000011
Cu	-1.27083534807609	5.84849141845865	3.01800501945111
Cu	-2.55550480960487	5.11228683952438	5.12785308981835
Cu	-1.27203000800663	8.80172551792992	0.90299999999988
Cu	-1.27292176748267	10.27586144536226	3.01425233526166

Cu	-2.54968349081861	6.58663964886752	0.90125000000008
Cu	-2.54915729672843	8.06268216495646	3.01506838451784
Cu	-3.83011245056246	7.32257210243262	5.12758847871082
Cu	-2.55458155542977	9.53508353969745	5.12722774634695
Cu	5.11657429351721	3.63101429090631	2.99910086702006
Cu	-3.82846166861030	8.80154842440020	0.90089999999996
Cu	-3.82816110089490	10.27679906365902	3.01485213313744
Cu	-5.10896318109769	9.53533966052518	5.12772745675405
Cu	3.83925706834846	2.89514906715194	5.12877871969618
Cu	2.56199287916724	3.63427415195822	3.01551197631216
Cu	-5.10948923633635	11.01397789051752	0.90299999999988
Cu	-5.10789204344701	12.49046766738160	3.01671664783271
Cu	-6.38627093537535	11.74534829958244	5.12923063100991
Cu	1.28477795153052	7.32292593483370	5.12385340657152
Cu	3.83929963825765	4.37421018275020	0.90370000000011
Cu	-6.38632475918468	13.22729282428320	0.90195000000005
Cu	-6.38705172807159	14.70372552894858	3.01478028779228
Cu	-7.66433660669761	13.96147295492305	5.12216998271605
Cu	3.83684702580162	5.85199750996932	3.00370593573588
Cu	-7.66305803703581	15.44290997393452	0.90019999999999
Cu	-7.66334077172400	16.91584238578357	3.01617277649647
Cu	-8.94188680191163	16.17545717971704	5.12167996039876
Cu	-2.54988798082509	11.01574882581424	0.90125000000008
Cu	-2.55319140721920	12.48956641326422	3.01470713593600
Cu	-8.94163172483352	17.65711037534856	0.90229999999991
Cu	1.28252067490564	1.42172196043382	3.01780103963486
N	8.02531449894741	6.74060800833731	8.59855215068792
N	10.01421554357927	7.63931652824910	8.59544847643038
N	10.34527514154494	2.78102669218397	8.54282593743918
N	12.32441680915871	3.69994063884435	8.54699960460570
H	6.08923520362599	6.94831748266896	7.76560531045468
H	6.90176519423863	5.39098256070072	7.43560846182019
H	11.28956930112280	8.29932578999942	11.13801686750695
H	6.69662308334216	6.21791949203311	11.14782928517518
H	10.09969265593769	7.82558118066105	13.26831190132975
H	7.84503713614176	6.80314067145174	13.27294645220176
H	6.25536016527987	5.72596067617721	9.06264043768596
H	11.74776597203188	7.60939151961608	7.40273815061335
H	11.14289970461214	9.24542931113342	7.79767209130367
H	11.96082000639927	8.25702894213824	9.04848457880356
H	8.33616214358713	2.70428221245106	7.85983195609760
H	9.38010525406707	1.34047466465286	7.36635698510964
H	13.36240101142775	4.85044722399481	11.01864167591281
H	8.78152900817019	2.73289416644180	11.00125211742631
H	11.98344563021291	4.74976763059321	13.09129947337974
H	9.73742249861199	3.71431341780590	13.08264574529237
H	8.81573076459365	1.44762945456276	9.05124232844402
H	14.06392534395211	3.49217688688927	7.39533905857998
H	13.67145066351292	5.17948549082515	7.83415199661966
H	14.32017898672397	4.04529570156897	9.06756848667191
C	9.01691821761742	7.19296862494057	7.75432293533568
C	8.37111548704720	6.92271814501852	9.93893174536602
C	9.65189211636577	7.50232075519652	9.93674696761731
C	9.62680766006375	7.57207260516387	12.32019989112127
C	6.74404176443990	6.17328752408863	8.19051453483161
C	10.30320556119260	7.83908157887991	11.12857035875798

```
C   8.34409169556848   6.98997047827626   12.32278563369432
C   7.69315640731125   6.65546577703584   11.13374778703461
C   11.28554484187343   8.22671501317265   8.18625082057952
C   11.41271201933136   3.07646513175382   7.72108850673233
C   10.55546779369249   3.24500609070716   9.84125631551067
C   11.82994827330608   3.83560628598148   9.84474466618693
C   11.59520805806381   4.32864497308571   12.16462899693738
C   9.14614731652514   2.03244415437872   8.18330415917062
C   12.37566130208224   4.39026237993220   11.00734697767857
C   10.31636234807199   3.73816903843923   12.16024578846782
C   9.77159819404024   3.18614358032271   10.99828879616641
C   13.66942940228120   4.13753853079346   8.18909368798417
```

Species from **Figure S97 b)**

```
234
Generated with con2xyz
Cu   10.22960353395594   0.66912051743587   5.09150479945521
Cu   1.28348144762368   8.80137133087048   0.90370000000011
Cu   1.29261289465688   10.27455774917227   3.01941900755696
Cu   10.22981644228912   2.15983268780656   0.90370000000011
Cu   10.25642791783212   3.62471165828554   3.05596217585195
Cu   8.94696734606824   2.90186376248413   5.06788282940512
Cu   0.00250467472907   9.53867521577704   5.13096445824432
Cu   1.28618552712949   2.89918212381143   5.13292716673612
Cu   8.95154948948265   4.37350180863156   0.90265000000002
Cu   8.94455987099217   5.85795280856768   2.99344360472933
Cu   7.67694157891174   5.11966706764322   5.13857377386816
Cu   -5.10580841645604   15.44273288040492   0.90125000000008
Cu   0.00582796479926   11.01539463875492   0.90335000000000
Cu   7.67348702667015   6.58787930357520   0.90335000000000
Cu   8.95448550962954   7.31908894389213   5.04384479365056
Cu   6.40288952785061   7.33085736684458   5.12316278837161
Cu   0.00905453697742   12.48719210561307   3.01636935366783
Cu   -1.27426755434229   11.75073451662214   5.12745775269281
Cu   6.39562905385170   8.80119423734088   0.90335000000000
Cu   6.41102845169063   10.25304987189875   3.00789334953266
Cu   5.12705254519596   9.53340107476134   5.14232735928298
Cu   -5.10665423511725   16.92318051738173   3.01521241809177
Cu   -6.38605752155749   16.18287758104111   5.12950852990227
Cu   5.11715761105111   11.01486335816588   0.90265000000002
Cu   5.11749238217561   12.48707327734724   3.01298221681797
Cu   3.84223076176122   11.75431770237281   5.13584129138254
Cu   -1.27243898800736   13.22906375957992   0.90370000000011
Cu   -1.27169947174191   14.70142167820302   3.01335970789398
Cu   3.83889065824457   13.22888666605020   0.90370000000011
Cu   3.84015065750540   14.70163499285886   3.01770532026172
Cu   2.56008150115282   13.96694115587941   5.13781932444568
Cu   -2.55175535594592   13.96382437600608   5.11961233864785
Cu   2.56093044042913   15.44237869334548   0.90404999999997
Cu   2.55714903886697   16.92206733089717   3.01951187343304
Cu   1.28571372855192   16.17960778650219   5.13176188707955
Cu   -2.55019471582868   15.44255578687520   0.90370000000011
Cu   1.28307246761067   17.65569362711116   0.90404999999997
Cu   11.51443394877215   1.45495909353637   3.07940320322228
Cu   12.79044903027473   0.68255040775847   5.14299385594932
```

Cu	-2.54900658440773	16.91616568887387	3.00416527351586
Cu	-3.83095722726755	16.18011077026641	5.12700898829915
Cu	12.78563463291059	2.15965559427684	0.90370000000011
Cu	12.75962937251878	3.63264016138959	3.05062907937700
Cu	11.50469689974433	2.88723422969665	5.71009659483406
Cu	1.28317471264508	4.37385599569088	0.90544999999992
Cu	11.50757217009817	4.37367890216116	0.90125000000008
Cu	11.48324855519991	5.85998074680885	3.00534393317644
Cu	10.23159181539185	5.10922943785284	5.11676000547294
Cu	-3.82825717864111	17.65657909475952	0.90335000000000
Cu	6.39518858228958	1.43472483503512	3.00760877286134
Cu	10.22899848230045	6.58929605181247	0.90335000000000
Cu	10.20395440638465	8.06092675146172	2.99019974554471
Cu	7.79318240828724	8.00667677511216	7.05777420728821
Cu	7.66466319976986	0.67649584774444	5.09065743201359
Cu	1.29669566212287	5.85073310970839	3.01886128479202
Cu	8.95134499947611	8.80119423734088	0.90440000000008
Cu	8.93245707225573	10.24711411303018	3.00710116590498
Cu	7.66955991674650	9.52929286294739	5.11475419559380
Cu	0.00760609166653	5.11021813858092	5.12938313919072
Cu	7.67379376167375	2.16000978133616	0.90370000000011
Cu	7.67522519160704	11.01362370345820	0.90404999999997
Cu	7.67337815166162	12.46647471491591	3.01423038753894
Cu	6.39613450695061	11.74555114231434	5.11382103497870
Cu	7.67230779204615	3.66017494571921	3.00796663378706
Cu	6.39171968235359	2.90020464087847	5.12483133349692
Cu	6.39501558385700	13.22853247899088	0.90055000000010
Cu	6.39861185180435	14.69075388391963	3.01665834938826
Cu	5.11888271634050	13.96092843472467	5.13140513772838
Cu	-3.82917738360190	13.22870957252048	0.90195000000005
Cu	-3.83118981928924	14.70273193649487	3.01458403663237
Cu	5.11674863105053	15.44220159981588	0.90370000000011
Cu	5.11595748267342	16.91066888294607	3.01702801457229
Cu	3.84179593146206	16.17582936748277	5.13266982830611
Cu	6.39583354385823	4.37350180863156	0.90404999999997
Cu	6.41637361334126	5.88348257468140	3.00914838575159
Cu	3.83940188321714	17.65569362711116	0.90370000000011
Cu	14.05758239667670	1.41403266324659	3.00946143656341
Cu	15.34141573699608	0.68037378943900	5.12980318838034
Cu	5.12694602443283	5.11530866442328	5.11198582667853
Cu	0.00531673982669	6.58752511651588	0.90370000000011
Cu	15.34196404851714	2.15930140721752	0.90299999999988
Cu	15.33537820568017	3.63788375270584	2.99502833464765
Cu	14.06464316511878	2.88533447837401	5.06672309421469
Cu	2.56286749527210	0.68772850622542	5.13166117638045
Cu	5.11664638607854	6.58699383592684	0.90229999999991
Cu	14.06349260571656	4.37226215392388	0.90089999999996
Cu	14.05132638224048	5.85132052654128	2.99820565374996
Cu	12.77468422930807	5.12294270192901	5.07872975069825
Cu	5.15229776924814	8.06298298276885	3.00827851345685
Cu	3.84884996576690	7.32644590488039	5.10847530148821
Cu	12.78543014290405	6.58982733240151	0.90159999999994
Cu	12.78733518880463	8.06473815485707	3.00924487559506
Cu	11.50566451136488	7.32670337670661	5.10624005111626
Cu	-6.38468883924485	17.65640200122992	0.90335000000000
Cu	3.84023230853310	1.42531262706877	3.01858296165978
Cu	11.50695870010339	8.80491320146391	0.90825000000006

Cu	11.48967418092082	10.26484056966163	3.02358347686641
Cu	10.21966366346423	9.52946625875376	5.12314301646814
Cu	3.83919739324809	8.80172551792992	0.90404999999997
Cu	3.86212836883397	10.26554956076314	3.02483883409938
Cu	10.23247481218681	11.01238404875052	0.90895000000003
Cu	10.23018535490891	12.48453706852250	3.01365650933527
Cu	8.95086506944946	11.74147497173842	5.11311180774812
Cu	2.56336319097902	9.53657030993686	5.13125051380517
Cu	5.11749831334812	0.68354872606321	5.13683452814715
Cu	8.95277642942183	13.22782410487224	0.90229999999991
Cu	8.94753996444877	14.69221332885615	3.01662277082512
Cu	7.67253935067805	13.95939317071950	5.13037620365064
Cu	-3.83145971319397	11.74799346317812	5.13099109819791
Cu	2.56113493043560	11.01504045169548	0.90404999999997
Cu	7.67205559668686	15.44290997393452	0.90159999999994
Cu	7.67160592277051	16.91054628780059	3.01306570835272
Cu	6.39578209757462	16.17624809581542	5.12762427110685
Cu	2.56910644001859	12.48456683515520	3.01923374585827
Cu	1.28435230080382	11.75345672868456	5.13018287793957
Cu	6.39511782884163	17.65604781417048	0.90404999999997
Cu	16.61820269256641	1.42119990389460	3.01209551422413
Cu	17.89694776128976	0.68154174024354	5.11968307859013
Cu	2.56123717545758	2.16018687486588	0.90404999999997
Cu	-5.10907408143652	13.96533567620369	5.12757817132883
Cu	17.89808897412964	2.16000978133616	0.90404999999997
Cu	17.89942109845175	3.63616688352109	3.01174451812556
Cu	16.61881902787924	2.89934464742589	5.11332029867824
Cu	1.28378818260220	13.22888666605020	0.90335000000000
Cu	1.28801611134760	14.70249032672857	3.01604285064641
Cu	16.61992426632008	4.37314762157224	0.90335000000000
Cu	16.62301456515078	5.85467238248930	3.00345537755963
Cu	15.34339784910443	5.11444840781673	5.08221574374331
Cu	0.00713459200262	13.96612489253668	5.12878065837170
Cu	2.57008116150478	5.11268174864340	5.13162175466663
Cu	15.34237302849272	6.58734802298616	0.90019999999999
Cu	15.34082437722640	8.06248142675811	3.01455672916603
Cu	14.06098134760294	7.32571180185712	5.12820976284100
Cu	0.00892162614916	8.06237567596488	3.01807865889267
Cu	0.00552122979574	15.44255578687520	0.90370000000011
Cu	14.06400383068912	8.80084005028156	0.90159999999994
Cu	14.05612881124553	10.27343763309358	3.02010677030548
Cu	12.78424810351117	9.53309593039298	5.13767720277768
Cu	0.00993872694355	16.92071339232898	3.00495541668021
Cu	-1.27327532332786	16.17740606416976	5.12370114957428
Cu	12.78174932299860	11.01220695522093	0.90789999999995
Cu	12.77910769479352	12.48369431569406	3.02052156179839
Cu	11.50457148029643	11.74587627354883	5.13649615150474
Cu	5.11756659106419	2.16018687486588	0.90370000000011
Cu	11.50777666006714	13.22835538546116	0.90195000000005
Cu	11.50527536626507	14.70086562844309	3.01628830497342
Cu	10.22699145270581	13.95896219821975	5.12489337751663
Cu	-1.27274572301080	17.65587072064088	0.90335000000000
Cu	8.95497431055958	1.42855074916293	2.99983295669617
Cu	10.22910072727251	15.44273288040492	0.90125000000008
Cu	10.22828153201610	16.91363826892309	3.01657834771412
Cu	8.94876549076412	16.17788888174765	5.12497928645949
Cu	8.95062928447193	17.65604781417048	0.90370000000011

Cu	19.17538343401029	1.42208521233732	3.01444446162362
Cu	0.00557475268957	0.68487748143341	5.13050182405698
Cu	-1.27345370552068	7.32191135837362	5.12797284307072
Cu	0.00511224984508	2.16018687486588	0.90404999999997
Cu	0.01265128628195	3.63726184036615	3.01682685342176
Cu	-1.27078686303766	2.89915061422155	5.12138083802683
Cu	2.56133942044221	6.58770221004548	0.90370000000011
Cu	2.56567891682945	8.06561324563953	3.01271337938985
Cu	-1.27284796797029	4.37403308922048	0.90370000000011
Cu	-1.26610649982836	5.85031285725936	3.01801559963213
Cu	-2.55269019987231	5.11293610900148	5.12906804507301
Cu	-1.27203000800663	8.80172551792992	0.90299999999988
Cu	-1.27094417434543	10.27425993025359	3.01635250851782
Cu	-2.54968349081861	6.58663964886752	0.90125000000008
Cu	-2.55010560331834	8.06399053667341	3.01558628723403
Cu	-3.83177069192845	7.32142404730991	5.13066915436214
Cu	-2.55387549790632	9.53356269769300	5.13235868297196
Cu	5.11966579297757	3.63922546785927	3.00871371757807
Cu	-3.82846166861030	8.80154842440020	0.90089999999996
Cu	-3.83370663988565	10.27361530973723	3.01663313882478
Cu	-5.10630575751762	9.53613976489323	5.13841101326180
Cu	3.84418720940569	2.90247084385630	5.13364027756484
Cu	2.56961915715451	3.64269724472659	3.01978370493266
Cu	-5.10948923633635	11.01397789051752	0.90299999999988
Cu	-5.11197382531966	12.48592667940081	3.01726486870516
Cu	-6.38703392265349	11.74706259366047	5.13494664171720
Cu	1.28897111631006	7.32458372315828	5.12649065840594
Cu	3.83929963825765	4.37421018275020	0.90370000000011
Cu	-6.38632475918468	13.22729282428320	0.90195000000005
Cu	-6.38979438628710	14.70308250628824	3.01478904854919
Cu	-7.66218222550009	13.96525279086419	5.13038142567475
Cu	3.86068949186447	5.85992567372959	3.01287248100072
Cu	-7.66305803703581	15.44290997393452	0.90019999999999
Cu	-7.66340631794452	16.91901215644871	3.01535773780949
Cu	-8.94309629836821	16.17968933788143	5.12449813012977
Cu	-2.54988798082509	11.01574882581424	0.90125000000008
Cu	-2.55372472893442	12.48926453537294	3.01539100160388
Cu	-8.94163172483352	17.65711037534856	0.90229999999991
Cu	1.28717900528974	1.42592824056385	3.02029835085251
N	7.34450352249128	7.16141481043556	9.85824310879248
N	9.25801587470672	8.19124885212133	9.62541010837175
N	10.34925381908614	2.75913255446739	8.54177669355749
N	12.32294088756192	3.68842632758935	8.54642002938800
H	5.25755717361097	7.06777966496267	10.15360814917849
H	5.82942878334883	6.71658265510448	8.49305493208666
H	10.99981565192673	8.51911981691597	11.93127648803791
H	6.57131246459403	6.15363713153461	12.48021091437986
H	10.27046614079687	7.64760688903296	14.15566162064814
H	8.10227151877794	6.49115993790589	14.42400767373208
H	6.04754312596676	5.49936076240405	9.78577881895595
H	10.17124351617876	9.10581470346079	7.98406523945458
H	10.54140381763472	9.86410791401828	9.56222903072669
H	11.30284417964995	8.30141666916383	9.10908048417597
H	8.32203938302528	2.66511938270807	7.92270535488614
H	9.37852540169189	1.34533202619150	7.34038732331164
H	13.32386737159677	4.90816284675495	10.99938385871977
H	8.75748780996288	2.75836253762928	10.98030609053687

H	11.91411551059671	4.86277175676995	13.05480814755499
H	9.67565351704582	3.81289120709757	13.04564083580358
H	8.86750953280417	1.37086124682133	9.04533789683182
H	14.05663355413449	3.49675258737528	7.38574334074046
H	13.67458454253994	5.17678331210750	7.86479496440768
H	14.32205548647261	4.01018560409386	9.06917275086485
C	8.13865735485970	7.79726766967486	8.93928512483064
C	7.94380118443469	7.15453276670016	11.11814061595457
C	9.17911435599877	7.81719873606944	10.96678265292437
C	9.62772468567704	7.51432753977782	13.28610476491304
C	6.03913934987374	6.57503494538025	9.55890550823437
C	10.04558266804498	8.00688275383890	12.04614806453689
C	8.39245769306615	6.85433225334644	13.43866578856918
C	7.52754421207620	6.65988740924080	12.35696920722312
C	10.38911111524528	8.90965868483704	9.03924028900227
C	11.42028910129812	3.04916438601830	7.72269470156810
C	10.54321590310641	3.25222363332937	9.83116910637939
C	11.81393618469094	3.85029716867179	9.83502882767293
C	11.54257942224768	4.41064444184208	12.13597754777899
C	9.15564655821664	1.99824455663466	8.19146268051118
C	12.34018183148934	4.44179146138716	10.98854198821206
C	10.26774068517836	3.81131624005798	12.13148008804590
C	9.74356681684584	3.22038667202669	10.97864963819533
C	13.66914094233235	4.12685376700408	8.19475586402263

Species from **Figure S97 c)**

234

Generated with con2xyz

Cu	10.22754724442588	0.66307549775268	5.08299690465969
Cu	1.28348144762368	8.80137133087048	0.90370000000011
Cu	1.29001882843034	10.27452026959007	3.01517442643402
Cu	10.22981644228912	2.15983268780656	0.90370000000011
Cu	10.25477696920518	3.62798370989486	3.06156149184442
Cu	8.94463352190443	2.89737151194105	5.07138942302667
Cu	0.00585250752713	9.53846090735608	5.13189332734788
Cu	1.28512956029246	2.89782435460703	5.13413470770868
Cu	8.95154948948265	4.37350180863156	0.90265000000002
Cu	8.93655804149669	5.87373628441135	3.02490139126967
Cu	7.67559889769867	5.10874209436540	5.14861118452323
Cu	-5.10580841645604	15.44273288040492	0.90125000000008
Cu	0.00582796479926	11.01539463875492	0.90335000000000
Cu	7.67348702667015	6.58787930357520	0.90335000000000
Cu	8.94681717925912	7.30868996449220	5.20117747242183
Cu	6.41168693963092	7.30661872968650	5.13921134388615
Cu	0.00834352054341	12.48665603441088	3.01676339886725
Cu	-1.27152628509955	11.74865255598683	5.12808256957522
Cu	6.39562905385170	8.80119423734088	0.90335000000000
Cu	6.41172023137697	10.25722330314467	2.98132433529502
Cu	5.13139411055015	9.53201427196956	5.08148862664528
Cu	-5.10594038726139	16.92134993654480	3.01538198850971
Cu	-6.38665172074737	16.18011921759182	5.13033487565432
Cu	5.11715761105111	11.01486335816588	0.90265000000002
Cu	5.11272320577894	12.49244395581464	3.00597684187206
Cu	3.84473852598180	11.75145687515944	5.12685739776492
Cu	-1.27243898800736	13.22906375957992	0.90370000000011

Cu	-1.27154685332694	14.69890274249500	3.01337561728186
Cu	3.83889065824457	13.22888666605020	0.90370000000011
Cu	3.83663247892111	14.70557568060461	3.01669921434391
Cu	2.55956608247970	13.96583355843334	5.13597765497920
Cu	-2.55127758405181	13.96126317757988	5.12093667354148
Cu	2.56093044042913	15.44237869334548	0.90404999999997
Cu	2.55488248232179	16.92444645115364	3.01956382789429
Cu	1.28671185231338	16.17687699283164	5.13445680173640
Cu	-2.55019471582868	15.44255578687520	0.90370000000011
Cu	1.28307246761067	17.65569362711116	0.90404999999997
Cu	11.51390818760828	1.45838146632344	3.07953728843663
Cu	12.79301035534024	0.67643797911953	5.14391008025683
Cu	-2.54849079949955	16.91371385138810	3.00334304311717
Cu	-3.83144360435518	16.17581186251957	5.12770918957573
Cu	12.78563463291059	2.15965559427684	0.90370000000011
Cu	12.75865892057911	3.63370207146276	3.05024733202010
Cu	11.50633906381553	2.88250310137348	5.67999211097134
Cu	1.28317471264508	4.37385599569088	0.90544999999992
Cu	11.50757217009817	4.37367890216116	0.90125000000008
Cu	11.47830284607559	5.86168123018146	3.00985069544619
Cu	10.23144638354775	5.10701344427503	5.12707591118842
Cu	-3.82825717864111	17.65657909475952	0.90335000000000
Cu	6.39544223516706	1.43261469583539	3.00489089575398
Cu	10.22899848230045	6.58929605181247	0.90335000000000
Cu	10.17706769968109	8.05308266157360	3.02417334877306
Cu	7.62553178542923	8.23449612095219	7.11282751120091
Cu	7.66138493853320	0.67167314656276	5.08531855017890
Cu	1.29604485396436	5.84969958973204	3.01827312289733
Cu	8.95134499947611	8.80119423734088	0.90440000000008
Cu	8.94376894855393	10.25544757311813	2.98383719074614
Cu	7.68119693689908	9.52034865474064	5.02850365527302
Cu	0.00827794639638	5.11051843312886	5.12890694975345
Cu	7.67379376167375	2.16000978133616	0.90370000000011
Cu	7.67502070161293	11.01362370345820	0.90404999999997
Cu	7.67259026859656	12.47505360763472	2.99702510362356
Cu	6.39941179189373	11.74405434737709	5.08098045509053
Cu	7.67419865466326	3.66183535606832	3.01079563873894
Cu	6.38953108805152	2.89248493389775	5.12250408285961
Cu	6.39501558385700	13.22853247899088	0.90055000000010
Cu	6.39686870777428	14.69395173394748	3.01613105360917
Cu	5.11837097368600	13.96053195654477	5.12543527112342
Cu	-3.82917738360190	13.22870957252048	0.90195000000005
Cu	-3.83007292750086	14.70010063254733	3.01447363707484
Cu	5.11674863105053	15.44220159981588	0.90370000000011
Cu	5.11504808642443	16.91220648526951	3.01644724208430
Cu	3.84243051811226	16.17468906485124	5.13549017090552
Cu	6.39583354385823	4.37350180863156	0.90404999999997
Cu	6.41333438696529	5.87380067019595	3.00932585301298
Cu	3.83940188321714	17.65569362711116	0.90370000000011
Cu	14.05713171393419	1.41423702443333	3.00941076604793
Cu	15.34273253135209	0.67784530876717	5.13219275361738
Cu	5.12322542765772	5.10695979883633	5.11167543095100
Cu	0.00531673982669	6.58752511651588	0.90370000000011
Cu	15.34196404851714	2.15930140721752	0.90299999999988
Cu	15.33588520651733	3.63809828622721	2.99303360666843
Cu	14.06916139553229	2.88344474263132	5.06173432644221
Cu	2.56079163400170	0.68420399115942	5.13255090757454

Cu	5.11664638607854	6.58699383592684	0.90229999999991
Cu	14.06349260571656	4.37226215392388	0.90089999999996
Cu	14.04956099713787	5.85077309408107	2.99691134398770
Cu	12.77561598255331	5.12295121751148	5.07689755562633
Cu	5.16605456229638	8.05472486582645	3.00496857961125
Cu	3.85256220436883	7.32260011061244	5.09195419530625
Cu	12.78543014290405	6.58982733240151	0.90159999999994
Cu	12.77731990353658	8.06140708515311	3.01269067063129
Cu	11.49999702529403	7.32339379771957	5.12009610800585
Cu	-6.38468883924485	17.65640200122992	0.90335000000000
Cu	3.84017969244467	1.42354010546447	3.01849362452267
Cu	11.50695870010339	8.80491320146391	0.90825000000006
Cu	11.48950822044525	10.25954606769414	3.02148341626721
Cu	10.22284675987658	9.52504411575760	5.12156457605413
Cu	3.83919739324809	8.80172551792992	0.90404999999997
Cu	3.85855595176044	10.26640770300189	3.00624596749411
Cu	10.23247481218681	11.01238404875052	0.90895000000003
Cu	10.23927250118141	12.49234726219921	2.99956062183290
Cu	8.95544548009073	11.74563862525665	5.06556576202739
Cu	2.56550409693769	9.53696864993854	5.11458888924287
Cu	5.11578667951230	0.67935297569490	5.13561900935346
Cu	8.95277642942183	13.22782410487224	0.90229999999991
Cu	8.94994914614826	14.69544876247025	3.01497212230047
Cu	7.67380438467671	13.95671317166789	5.12597537665111
Cu	-3.83042983570821	11.74605161551789	5.13077804893399
Cu	2.56113493043560	11.01504045169548	0.90404999999997
Cu	7.67205559668686	15.44290997393452	0.90159999999994
Cu	7.67200569397032	16.91172902259725	3.01285925036195
Cu	6.39627226895257	16.17592459609604	5.12738371966087
Cu	2.56649682195109	12.48577103164040	3.01779823915867
Cu	1.28757843427749	11.75123864131286	5.13240710289490
Cu	6.39511782884163	17.65604781417048	0.90404999999997
Cu	16.61837213239517	1.42098293338478	3.01283252734148
Cu	17.89678770717005	0.67978448863403	5.12158384532412
Cu	2.56123717545758	2.16018687486588	0.90404999999997
Cu	-5.10865474255421	13.96321430820861	5.12861952810094
Cu	17.89808897412964	2.16000978133616	0.90404999999997
Cu	17.89990480684789	3.63606959322462	3.01165895988905
Cu	16.62118131377239	2.89887902732334	5.11239250302277
Cu	1.28358369260824	13.22888666605020	0.90335000000000
Cu	1.28651489537445	14.70312312718294	3.01449620512254
Cu	16.61992426632008	4.37314762157224	0.90335000000000
Cu	16.62278548895317	5.85492807212634	3.00254375836997
Cu	15.34614373092503	5.11520393743935	5.07754030540432
Cu	0.00954687765213	13.96349049975610	5.13055585668432
Cu	2.56929110104739	5.11135197738534	5.13024347710538
Cu	15.34237302849272	6.58734802298616	0.90019999999999
Cu	15.33787038158404	8.06175696693635	3.01383115080489
Cu	14.06160163277914	7.32487727586878	5.12432787374212
Cu	0.00846199811265	8.06217681935251	3.01746195146218
Cu	0.00552122979574	15.44255578687520	0.90370000000011
Cu	14.06400383068912	8.80084005028156	0.90159999999994
Cu	14.05528664189073	10.26997252934253	3.01977222153042
Cu	12.78450798786336	9.53216308880273	5.13673295792733
Cu	0.00950579743748	16.91897885122220	3.00377693726960
Cu	-1.27339354995898	16.17269460314976	5.12157784662184
Cu	12.78174932299860	11.01220695522093	0.90789999999995

Cu	12.78419586015908	12.48290325150145	3.01654389923257
Cu	11.50688264821575	11.74744153268053	5.12193967140408
Cu	5.11756659106419	2.16018687486588	0.90370000000011
Cu	11.50777666006714	13.22835538546116	0.90195000000005
Cu	11.50927017365180	14.70426855891860	3.01462538743261
Cu	10.22738246071768	13.95800756410100	5.12152573425873
Cu	-1.27274572301080	17.65587072064088	0.90335000000000
Cu	8.95516037199958	1.42923203086226	2.99853712803846
Cu	10.22910072727251	15.44273288040492	0.90125000000008
Cu	10.22857578187975	16.91445762740703	3.01699954483753
Cu	8.94779038879278	16.17522245481507	5.12848936126703
Cu	8.95062928447193	17.65604781417048	0.90370000000011
Cu	19.17556030229316	1.42208444926989	3.01565151305255
Cu	0.00473550611072	0.68276609907350	5.12983957295277
Cu	-1.27077342202837	7.32202188268892	5.12763482694357
Cu	0.00511224984508	2.16018687486588	0.90404999999997
Cu	0.01230143631181	3.63663258782258	3.01684744172563
Cu	-1.27081215272736	2.89947409532160	5.12263464146432
Cu	2.56133942044221	6.58770221004548	0.90370000000011
Cu	2.56757871798707	8.06387693227936	3.00515153725719
Cu	-1.27284796797029	4.37403308922048	0.90370000000011
Cu	-1.26557064511565	5.85036498678645	3.01798033232859
Cu	-2.55126549307784	5.11267163490725	5.13049545066010
Cu	-1.27203000800663	8.80172551792992	0.90299999999988
Cu	-1.27152127963852	10.27329246372364	3.01654274700159
Cu	-2.54968349081861	6.58663964886752	0.90125000000008
Cu	-2.55114489257091	8.06354870661624	3.01488187790852
Cu	-3.82977327724930	7.32150704919643	5.12969748576879
Cu	-2.55201435004925	9.53269144499272	5.13253377425078
Cu	5.11880683475153	3.63484160679503	3.00965521126483
Cu	-3.82846166861030	8.80154842440020	0.90089999999996
Cu	-3.83357513682402	10.27235257118036	3.01690074130535
Cu	-5.10524690273466	9.53484412406840	5.13766198396897
Cu	3.84067136108107	2.89662898304994	5.13338828584601
Cu	2.56821886035381	3.63987581342246	3.02024641149278
Cu	-5.10948923633635	11.01397789051752	0.90299999999988
Cu	-5.11059353904830	12.48391056677674	3.01745359839756
Cu	-6.38629957760280	11.74571304171157	5.13363488290923
Cu	1.29371993377291	7.32539686785569	5.12373241046097
Cu	3.83929963825765	4.37421018275020	0.90370000000011
Cu	-6.38632475918468	13.22729282428320	0.90195000000005
Cu	-6.38785951567752	14.70187297099857	3.01463057349314
Cu	-7.66187548533777	13.96360746632825	5.12934221093065
Cu	3.85966204017576	5.85468809364428	3.00883883693430
Cu	-7.66305803703581	15.44290997393452	0.90019999999999
Cu	-7.66268391203167	16.91943410176861	3.01639243167038
Cu	-8.94459875690214	16.17717639070663	5.12599138266951
Cu	-2.54988798082509	11.01574882581424	0.90125000000008
Cu	-2.55337928147013	12.48720501202407	3.01641349871810
Cu	-8.94163172483352	17.65711037534856	0.90229999999991
Cu	1.28639630118410	1.42460388227170	3.02067380391173
N	5.75147849520307	9.95860220411031	8.54214211166994
N	7.77427131900146	10.78481207061598	8.53264012722927
N	10.47745685431294	2.48664594891995	8.53439170059242
N	12.42893752694142	3.46244519427418	8.53779506159196
H	3.92345270475273	9.25781812031645	7.74973368590218
H	5.02397655463583	8.00839390247511	8.39760193218162

```
H   8.14677778137326   13.66924251393575   8.67140602988007
H   3.46727043919383   11.75901387848939   8.68978834988502
H   6.14444113755807   15.16008106720474   8.72089349827495
H   3.85465163477679   14.22517569904794   8.72882134985844
H   4.11785191775062   9.07034612438433   9.52492889587034
H   9.65905803000093   9.90562629205064   8.36357606859965
H   9.55904840177589   11.56953832855237   7.72087872023177
H   9.57306907988214   11.29575844670898   9.49515553004291
H   8.54119790547407   2.50411541705096   7.67368365519757
H   9.48648920075073   1.00495365512489   7.41812748329034
H   13.58385442236898   4.34102685302040   11.07217964006137
H   9.07532816501770   2.08242963645018   11.06332062057512
H   12.34149342402225   3.94602895293009   13.18900093563532
H   10.12851636439046   2.83721996096316   13.18455128109739
H   8.79069799259295   1.34922189529986   9.01845662049545
H   14.20194790666431   3.37288493378769   7.41162304613276
H   13.58212368313863   5.02880554724237   7.69548201611827
H   14.35732538353028   4.10904122886401   9.02305129643317
C   7.07321631861591   9.61117084151543   8.38328915652537
C   5.60663081084684   11.33959742421922   8.67147259005120
C   6.91172304967611   11.87272566884290   8.66545730235481
C   6.01189387351810   14.07813235895707   8.73170604258514
C   4.63400487218170   9.01911375740942   8.55532101650904
C   7.14069892804033   13.25215041651929   8.70376226905662
C   4.70660117295877   13.54516312057363   8.73630202235391
C   4.47808357723757   12.16503729046145   8.71416750283918
C   9.22913186874062   10.89887941540107   8.52891985989631
C   11.48488160330412   2.91421829513922   7.69682343772192
C   10.76114399393886   2.78298102538855   9.86834729676300
C   12.01762730200354   3.41175535371327   9.87064767318924
C   11.90942177358904   3.62215014786834   12.24330285015238
C   9.25265294537954   1.80284025208711   8.13518994866786
C   12.61515972994908   3.84553572305198   11.05933461105753
C   10.64975413032075   2.99115505096212   12.24085018373034
C   10.05209850806690   2.56188396884327   11.05428590295916
C   13.71205910521837   4.03161919994761   8.14145161224874
```

Species from **Figure S97 d)**

```
234
Generated with con2xyz
Cu   10.22868310745960   0.68210151038097   5.13603362604442
Cu   1.28348144762368   8.80137133087048   0.90370000000011
Cu   1.28618358548897   10.27694122925838   3.01402260386554
Cu   10.22981644228912   2.15983268780656   0.90370000000011
Cu   10.22711319043871   3.63445454185888   2.99867173802472
Cu   8.94890918618233   2.89983365012614   5.10736769260792
Cu   0.00374315542355   9.54163442834902   5.13965125309924
Cu   1.28549253705952   2.89997931175686   5.13313472303485
Cu   8.95154948948265   4.37350180863156   0.90265000000002
Cu   8.93026564776408   5.89422503151029   3.01003480249837
Cu   7.66792462477840   5.11669357074617   5.08304659325597
Cu   -5.10580841645604   15.44273288040492   0.90125000000008
Cu   0.00582796479926   11.01539463875492   0.90335000000000
Cu   7.67348702667015   6.58787930357520   0.90335000000000
Cu   8.92900493950832   7.33013637804817   5.20422689352959
Cu   6.40980487748457   7.33541751166284   5.15961532098322
```

Cu	0.00662755534031	12.48918026799318	3.01894677332300
Cu	-1.27361803213745	11.75114365315121	5.13296140773617
Cu	6.39562905385170	8.80119423734088	0.90335000000000
Cu	6.42711669335552	10.23138281752486	3.01539712899724
Cu	5.11843876523526	9.53353070975626	5.07386520319874
Cu	-5.10981624426461	16.91877113622861	3.01886196101622
Cu	-6.39023539299284	16.18011348989139	5.13676392143786
Cu	5.11715761105111	11.01486335816588	0.90265000000002
Cu	5.11522301405277	12.48667197227952	3.00391973933951
Cu	3.83794386719527	11.75359630730384	5.11750785132909
Cu	-1.27243898800736	13.22906375957992	0.90370000000011
Cu	-1.27478694289273	14.70256683205186	3.01806846321622
Cu	3.83889065824457	13.22888666605020	0.90370000000011
Cu	3.83719592947707	14.70418168228374	3.01783089877487
Cu	2.55567900811462	13.96857839362137	5.13454351156068
Cu	-2.55300006008306	13.96633015802070	5.12675122592632
Cu	2.56093044042913	15.44237869334548	0.90404999999997
Cu	2.56020263634038	16.91921582597506	3.01825278654486
Cu	1.28468350355835	16.18270384231596	5.13890842475725
Cu	-2.55019471582868	15.44255578687520	0.90370000000011
Cu	1.28307246761067	17.65569362711116	0.90404999999997
Cu	11.50614312914514	1.42248879551714	3.02000645862966
Cu	12.78421874304067	0.68709035673228	5.13736858164064
Cu	-2.55203859014415	16.91814865034942	3.01762707283575
Cu	-3.83224606831343	16.18073256825455	5.13406603062241
Cu	12.78563463291059	2.15965559427684	0.90370000000011
Cu	12.77943652544277	3.64147170068337	3.01330747851591
Cu	11.50295066529679	2.90246177864673	5.12623101206270
Cu	1.28317471264508	4.37385599569088	0.90544999999992
Cu	11.50757217009817	4.37367890216116	0.90125000000008
Cu	11.49442567555066	5.85983323934159	2.99430844703398
Cu	10.22341806031726	5.11167870842912	5.06764878246195
Cu	-3.82825717864111	17.65657909475952	0.90335000000000
Cu	6.39624430837274	1.42481727845683	3.01204283392251
Cu	10.22899848230045	6.58929605181247	0.90335000000000
Cu	10.18548460923907	8.05522337702122	3.01885582861875
Cu	7.73615507914407	7.96136040219276	7.68364167555973
Cu	7.67037131924416	0.68354564273375	5.12946555165059
Cu	1.28824021031786	5.85264203211515	3.01978337166276
Cu	8.95134499947611	8.80119423734088	0.90440000000008
Cu	8.92273266157165	10.23089594918829	3.02303273241550
Cu	7.66870478443779	9.50957774952008	5.23087755868728
Cu	0.00744323000039	5.11389163637337	5.13295642679507
Cu	7.67379376167375	2.16000978133616	0.90370000000011
Cu	7.67522519160704	11.01362370345820	0.90404999999997
Cu	7.67185809176554	12.47176959227684	2.99247806217617
Cu	6.39604060856415	11.74296305227509	5.07542231313764
Cu	7.67226036407196	3.65704446968496	3.00346725689134
Cu	6.38905084654274	2.89515156241135	5.10927350179328
Cu	6.39501558385700	13.22853247899088	0.90055000000010
Cu	6.39639144778903	14.69495056590933	3.01608694228938
Cu	5.11425932503692	13.96434865827414	5.12070230218736
Cu	-3.82917738360190	13.22870957252048	0.90195000000005
Cu	-3.83044509159136	14.70222599159705	3.01759585540011
Cu	5.11674863105053	15.44220159981588	0.90370000000011
Cu	5.11640330972087	16.91533474591567	3.01791434921344
Cu	3.83828928111669	16.18143301532485	5.13248769707421

Cu	6.39583354385823	4.37350180863156	0.90404999999997
Cu	6.40898147586941	5.88690082431225	3.00199712754103
Cu	3.83940188321714	17.65569362711116	0.90370000000011
Cu	14.06109649338083	1.42375333572061	3.01996512709025
Cu	15.33857902313554	0.68420591153644	5.13588165354675
Cu	5.11678771527641	5.11625193171423	5.07009380636251
Cu	0.00531673982669	6.58752511651588	0.90370000000011
Cu	15.34196404851714	2.15930140721752	0.90299999999988
Cu	15.33662640356060	3.63893049505393	3.01630213864074
Cu	14.05842971672429	2.90085076902697	5.13349656958767
Cu	2.56049545489185	0.68577713822622	5.13533422132595
Cu	5.11664638607854	6.58699383592684	0.90229999999991
Cu	14.06349260571656	4.37226215392388	0.90089999999996
Cu	14.05769997205913	5.85391181037993	3.01244313430710
Cu	12.77863548277059	5.11472703682500	5.11572551683417
Cu	5.15134461767602	8.05435029782995	3.00286532081799
Cu	3.84624770785256	7.32640050554592	5.09527483620408
Cu	12.78543014290405	6.58982733240151	0.90159999999994
Cu	12.78297822597139	8.06694319624448	3.00776563376303
Cu	11.49714557754623	7.32817842485580	5.08850423010186
Cu	-6.38468883924485	17.65640200122992	0.90335000000000
Cu	3.83840699462618	1.42118966110510	3.01895337585127
Cu	11.50695870010339	8.80491320146391	0.90825000000006
Cu	11.48435382099714	10.26216680149184	3.02067735240091
Cu	10.21878397609589	9.52457786149564	5.12124617645415
Cu	3.83919739324809	8.80172551792992	0.90404999999997
Cu	3.85655482432307	10.26737218384823	3.00166118640402
Cu	10.23247481218681	11.01238404875052	0.90895000000003
Cu	10.23223350403769	12.48536314418664	2.99945175786655
Cu	8.94899369731094	11.74216393529636	5.06586625241968
Cu	2.55714645236595	9.53935150147392	5.11574935218716
Cu	5.11546225628952	0.68224196790127	5.13958974968828
Cu	8.95277642942183	13.22782410487224	0.90229999999991
Cu	8.95062146124013	14.69482618751730	3.01375202113393
Cu	7.67365112152596	13.95633117607657	5.11985770379916
Cu	-3.83149764589629	11.74996319195653	5.13291295698881
Cu	2.56113493043560	11.01504045169548	0.90404999999997
Cu	7.67205559668686	15.44290997393452	0.90159999999994
Cu	7.67419103127657	16.91254016191116	3.01543987547544
Cu	6.39481702967765	16.17896166955379	5.12672482973187
Cu	2.56369025882118	12.48747227292613	3.01549297816419
Cu	1.28334594534052	11.75265218098201	5.13628212655386
Cu	6.39511782884163	17.65604781417048	0.90404999999997
Cu	16.62059525968475	1.42276894664461	3.01768221300216
Cu	17.89675337335444	0.68101949459133	5.12809731174386
Cu	2.56123717545758	2.16018687486588	0.90404999999997
Cu	-5.11160023566376	13.96647002113163	5.13302652293116
Cu	17.89808897412964	2.16000978133616	0.90404999999997
Cu	17.89795932246980	3.63579120355400	3.01787928160250
Cu	16.61560500270533	2.89931071967174	5.12683353107272
Cu	1.28378818260220	13.22888666605020	0.90335000000000
Cu	1.28396754024100	14.70493910596321	3.01768379169181
Cu	16.61992426632008	4.37314762157224	0.90335000000000
Cu	16.61707752959117	5.85146009472290	3.01837582285864
Cu	15.33586924561223	5.11300501089999	5.13195542200902
Cu	0.00567665629597	13.96755922120846	5.13382554072847
Cu	2.56701460920279	5.11615658590198	5.13008629226706

Cu	15.34237302849272	6.58734802298616	0.90019999999999
Cu	15.33959333352293	8.06408631966885	3.01833340805636
Cu	14.05549499817403	7.32581552844504	5.13063993388331
Cu	0.00573241735790	8.06413633618407	3.02103870984586
Cu	0.00552122979574	15.44255578687520	0.90370000000011
Cu	14.06400383068912	8.80084005028156	0.90159999999994
Cu	14.05491077346697	10.27431036187124	3.02060890659941
Cu	12.78139241760318	9.53496573467707	5.13729595706566
Cu	0.00526843761243	16.91933264664403	3.01870229238090
Cu	-1.27309986287742	16.18124034105011	5.13354428316976
Cu	12.78174932299860	11.01220695522093	0.90789999999995
Cu	12.78139233577614	12.48490708268858	3.01674083026668
Cu	11.50386833161298	11.75104497774955	5.12107980003478
Cu	5.11756659106419	2.16018687486588	0.90370000000011
Cu	11.50777666006714	13.22835538546116	0.90195000000005
Cu	11.50785552807144	14.70231152025678	3.01598743728101
Cu	10.22656888209170	13.95839636132432	5.12549284936629
Cu	-1.27274572301080	17.65587072064088	0.90335000000000
Cu	8.94842106524429	1.42590469913191	3.01552430139074
Cu	10.22910072727251	15.44273288040492	0.90125000000008
Cu	10.22948817743953	16.91416724373341	3.01811490852378
Cu	8.94967297606726	16.17476256445765	5.13090522450561
Cu	8.95062928447193	17.65604781417048	0.90370000000011
Cu	19.17503889861479	1.42271889327838	3.01798977134867
Cu	0.00523410588034	0.68224225196341	5.13372045172099
Cu	-1.27142028379726	7.32507168702667	5.13481624867673
Cu	0.00511224984508	2.16018687486588	0.90404999999997
Cu	0.00872011587998	3.63855413420097	3.01909697902208
Cu	-1.27239498714115	2.89906877416660	5.12795667850221
Cu	2.56133942044221	6.58770221004548	0.90370000000011
Cu	2.56135678435606	8.06614096898747	3.00650453999969
Cu	-1.27284796797029	4.37403308922048	0.90370000000011
Cu	-1.27138871908286	5.85158864221715	3.02008745698882
Cu	-2.55280099972711	5.11335289003642	5.13451310130065
Cu	-1.27203000800663	8.80172551792992	0.90299999999988
Cu	-1.27264178684351	10.27454710156233	3.01872905707509
Cu	-2.54968349081861	6.58663964886752	0.90125000000008
Cu	-2.55197728370446	8.06292935983833	3.01835400272060
Cu	-3.83321933977437	7.32444512310368	5.13387438626683
Cu	-2.55266991417531	9.53604393995179	5.13656746768096
Cu	5.11686773701679	3.63386748262261	2.99963851759732
Cu	-3.82846166861030	8.80154842440020	0.90089999999996
Cu	-3.83284724825776	10.27393570777247	3.01854182398621
Cu	-5.10914980137418	9.53898466579466	5.13928181214656
Cu	3.84248134291088	2.90033069769832	5.13036945714828
Cu	2.56522895420522	3.64067047649928	3.01956683612206
Cu	-5.10948923633635	11.01397789051752	0.90299999999988
Cu	-5.11239599567077	12.48710880368501	3.01909063571707
Cu	-6.38963469230537	11.75064112766039	5.13665166954406
Cu	1.28929656280869	7.32818301493332	5.13405221515612
Cu	3.83929963825765	4.37421018275020	0.90370000000011
Cu	-6.38632475918468	13.22729282428320	0.90195000000005
Cu	-6.38945741457341	14.70226311746262	3.01796068770302
Cu	-7.66546180536491	13.96486806292607	5.13923161124362
Cu	3.84762083075286	5.85746679565274	2.99605975501601
Cu	-7.66305803703581	15.44290997393452	0.90019999999999
Cu	-7.66463489240795	16.91738616338088	3.01877860673793

```
Cu   -8.94538984379969   16.17612161936319   5.13434162707200
Cu   -2.54988798082509   11.01574882581424   0.90125000000008
Cu   -2.55319230802880   12.49004721795713   3.01757701340600
Cu   -8.94163172483352   17.65711037534856   0.90229999999991
Cu   1.28481165464430   1.42311872461218   3.01999921113051
N   5.79421816930748   10.09790930612887   8.39711918696021
N   7.83389489883175   10.87818864170554   8.38052007865605
N   7.39690219817769   5.08142782526206   8.35169656586146
N   9.50633459841031   5.64566109503579   8.39513445676091
H   3.91282152112505   9.43278816375610   7.70214115890499
H   5.01028217851526   8.16844334739793   8.31773644991980
H   8.26741639937392   13.75633180105564   8.56091378669496
H   3.55016904478495   11.94120348874557   8.58162077974753
H   6.29704350759211   15.28594217729442   8.65561342647697
H   3.98783647604502   14.39906017447456   8.66599514375441
H   4.18791900264895   9.26836621175125   9.46986226412452
H   9.70596718552421   9.98333640874369   8.15226915481601
H   9.66142069912263   11.71653158760001   7.74194218482817
H   9.60904324859183   11.20691301315803   9.46375355921031
H   5.59651703577571   5.21396335359830   9.44727638370337
H   5.61711643230417   6.06065712467129   7.85973473305145
H   11.53705571944866   3.57468144802013   8.68048985652168
H   6.65535563447878   2.27371320724920   8.61594797723460
H   10.83664496621888   1.18373769863203   8.83512614282523
H   8.44565561562488   0.54771088317564   8.80518833111939
H   5.49955910496401   4.29114923543710   7.90854829463263
H   10.50870599776689   7.46039890058027   8.15048694660328
H   11.11392699167133   6.44035687108548   9.49943299526770
H   11.49296878087050   6.01896342462317   7.79423406070984
C   7.10795235787557   9.71813626421802   8.24087994719149
C   5.68049267197885   11.48103991124205   8.53090790598480
C   6.99603186423925   11.98506895722820   8.52307306823331
C   6.14150754331319   14.20718757930878   8.64069384719765
C   4.65526230658328   9.19034672236520   8.47683210156303
C   7.25357482760214   13.35847437470294   8.58568223117564
C   4.82580011315217   13.70213294938997   8.64649187245461
C   4.56911369965321   12.32694251265833   8.59884109591815
C   9.28964324526166   10.95392903227988   8.44153268946153
C   8.24419064865675   6.15789921587010   8.21263819799485
C   8.10990839836925   3.89778156460984   8.55061948151972
C   9.47057517726369   4.26071139675177   8.57251572127894
C   10.07879722232748   1.96483184451097   8.77517462214940
C   5.94077146285645   5.16626913317253   8.40269686151540
C   10.48237255194373   3.30206465605516   8.68844375091757
C   8.71727216477281   1.60234106760435   8.75751188623076
C   7.70526390001266   2.56255729091486   8.65015019026987
C   10.73120921587640   6.43641130392852   8.46781186045516
```

Species **[Cu(BIMe)]**

```
22
Cu-NHC-2_1_complex_neutral_opt.log      Energy: -1316738.3384431
N         -0.09109       -1.07648        0.04078
H         -0.19920       -2.95967        0.94695
H         -1.64325       -2.42844        0.05146
H          2.42241        2.50546       -0.01459
```

```
C          -0.90981        0.00000        0.06028
C           1.23702       -0.69802        0.00945
C           1.23702        0.69802        0.00945
N          -0.09109        1.07649        0.04078
H           2.42241       -2.50546       -0.01459
C           3.60050        0.69827       -0.03890
H           4.54579        1.22730       -0.05757
H           4.54578       -1.22731       -0.05757
C          -0.55365       -2.44218        0.05269
H          -0.19933       -2.97302       -0.83370
C           2.41841        1.42231       -0.01484
C           3.60050       -0.69827       -0.03890
C           2.41841       -1.42231       -0.01484
C          -0.55364        2.44218        0.05269
H          -1.64325        2.42844        0.05146
H          -0.19919        2.95967        0.94695
H          -0.19933        2.97302       -0.83370
Cu         -2.88090       -0.00000       -0.04202
```

Species **[Cu(BIMe)]$^+$**

```
22
Cu-NHC-2_1_complex_positive.log Energy: -1316630.6036579
N           0.12037       -1.08275        0.00000
H           0.19266       -2.97266       -0.89029
H           1.64804       -2.48566       -0.00001
H          -2.39619        2.51021       -0.00000
C           0.91669        0.00000        0.00000
C          -1.20876       -0.69655       -0.00000
C          -1.20876        0.69655       -0.00000
N           0.12036        1.08275       -0.00000
H          -2.39619       -2.51021        0.00001
C          -3.56404        0.70034        0.00000
H          -4.51077        1.22616        0.00000
H          -4.51077       -1.22616        0.00001
C           0.55939       -2.45971       -0.00001
H           0.19267       -2.97268        0.89026
C          -2.38867        1.42755       -0.00000
C          -3.56404       -0.70034        0.00000
C          -2.38867       -1.42755        0.00000
C           0.55939        2.45971       -0.00000
H           1.64804        2.48566        0.00001
H           0.19267        2.97267       -0.89028
H           0.19266        2.97267        0.89027
Cu          2.82023       -0.00000        0.00000
```

Species **[Cu(BIMe)$_2$]**

```
43
Cu-NHC-2_2_complex_neutral.log  Energy: -1604224.9237036
N         -2.72753       -1.05489        0.24533
H         -2.38039       -3.04879       -0.31412
H         -1.23056       -2.33157        0.84172
H         -5.23603        2.45227       -0.51597
C         -1.88128        0.00001        0.00000
C         -4.04718       -0.68416        0.15121
C         -4.04718        0.68415       -0.15121
N         -2.72754        1.05490       -0.24533
H         -5.23602       -2.45229        0.51597
C         -6.42074        0.67912       -0.14468
H         -7.36518        1.19908       -0.25402
H         -7.36517       -1.19911        0.25403
C         -2.27976       -2.38305        0.54872
H         -2.85624       -2.79511        1.38047
C         -5.23025        1.39163       -0.29711
C         -6.42073       -0.67914        0.14469
C         -5.23024       -1.39164        0.29712
C         -2.27978        2.38306       -0.54873
H         -1.23057        2.33159       -0.84169
H         -2.85624        2.79509       -1.38050
H         -2.38045        3.04881        0.31410
N          2.72753       -1.05489       -0.24533
H          2.38041       -3.04880        0.31411
H          1.23056       -2.33157       -0.84171
H          5.23604        2.45227        0.51597
C          1.88128        0.00001       -0.00000
C          4.04718       -0.68416       -0.15121
C          4.04718        0.68415        0.15121
N          2.72754        1.05490        0.24533
H          5.23602       -2.45229       -0.51597
C          6.42074        0.67911        0.14469
H          7.36518        1.19908        0.25403
H          7.36517       -1.19911       -0.25402
C          2.27976       -2.38305       -0.54872
H          2.85623       -2.79510       -1.38049
C          5.23025        1.39163        0.29712
C          6.42073       -0.67914       -0.14468
C          5.23024       -1.39165       -0.29711
C          2.27978        2.38306        0.54872
H          1.23057        2.33160        0.84169
H          2.85624        2.79510        1.38050
H          2.38045        3.04881       -0.31410
Cu         0.00000        0.00001       -0.00000
```

Species **$[Cu(BIMe)_2]^+$**

```
43
Cu-NHC-2_2_complex_positive.log Energy: -1604150.0887378
N          -2.73731       -0.76276        0.76238
H          -2.65837       -2.72898        1.47016
H          -1.20522       -1.74516        1.74432
H          -5.25365        1.77404       -1.77314
C          -1.92686        0.00001       -0.00001
C          -4.06628       -0.49272        0.49247
C          -4.06629        0.49272       -0.49247
N          -2.73732        0.76277       -0.76238
H          -5.25362       -1.77406        1.77315
C          -6.42468        0.49481       -0.49456
H          -7.37093        0.86745       -0.86700
H          -7.37091       -0.86748        0.86704
C          -2.29374       -1.73446        1.73357
H          -2.65843       -1.47159        2.72822
C          -5.24687        1.00817       -1.00765
C          -6.42467       -0.49484        0.49458
C          -5.24685       -1.00819        1.00767
C          -2.29377        1.73446       -1.73358
H          -1.20525        1.74517       -1.74434
H          -2.65848        1.47160       -2.72822
H          -2.65840        2.72899       -1.47016
N           2.73732       -0.76241       -0.76274
H           2.65844       -2.72828       -1.47149
H           1.20524       -1.74438       -1.74511
H           5.25364        1.77321        1.77398
C           1.92686        0.00001       -0.00000
C           4.06629       -0.49249       -0.49270
C           4.06629        0.49249        0.49270
N           2.73732        0.76241        0.76273
H           5.25363       -1.77322       -1.77398
C           6.42468        0.49458        0.49481
H           7.37092        0.86704        0.86744
H           7.37092       -0.86707       -0.86742
C           2.29376       -1.73364       -1.73440
H           2.65840       -1.47027       -2.72893
C           5.24686        1.00770        1.00814
C           6.42468       -0.49460       -0.49480
C           5.24686       -1.00771       -1.00813
C           2.29376        1.73365        1.73439
H           1.20524        1.74440        1.74509
H           2.65839        1.47028        2.72892
H           2.65845        2.72829        1.47148
Cu         -0.00000        0.00001       -0.00001
```
