# Supplementary material for: N-Heterocyclic carbenes on copper: a dynamic and sensitive interplay between surface functionalization and etching
Source: Chem Sci. 2026 Aug 26;17(41):19838–48. doi: 10.1039/d6sc04254f (PMC13539560; doi:10.1039/d6sc04254f)
Supplement: Supplementary file 2 [file SC-017-D6SC04254F-s002.pdf]

This journal is © The Royal Society of Chemistry 2026

N-Heterocyclic Carbenes on Copper: A Dynamic and Sensitive Interplay between Surface Functionalization and Etching

Supporting Information

Table of Contents

1 Chemical Synthesis 3

1.1 BIMeH·HCO_3 3

1.2 IPr·CO_2 4

1.3 IPrH·HCO_3 4

1.4 BIiPrH·HCO_3 5

1.5 IMe·CO_2 5

1.6 Triazolium **1** 6

1.7 [Cu(BIMe)$_2$]PF_6 6

1.8 BIMeO 6

1.9 Aldehyde **2** 6

2 NMR Spectra 7

3 General Surface Modification Procedures 15

3.1 Cleaning as pretreatment 15

3.2.1 Liquid phase modification 15

3.2.2 Vapor phase modification 16

4 XPS Measurements 16

4.1 Solvent screening 18

4.2 Light and temperature dependency 19

4.3 Time and concentration dependency 20

4.4 Data associated with Figure 2 22

4.5 Data associated with Figure 3 35

4.6 Data associated with Figure 4 39

4.7 Data associated with Figure 6 45
4.8 Data associated with Figure 7 48
4.9 Data associated with Figure S18 51
4.10 Data associated with Figure S19 55
4.11 Repeated measurements of the same sample 65
5 XPS Surface Coverage Estimation 68
6 AFM Measurements 70
7 WLI Measurements 71
8 UV-Vis Measurements 72
9 ICP-OES Measurements 73
10 CV Experiments and Redox Active Additives 75
11 EPR Data 87
12 Control Experiments for Copper Etching 89
12.1 Organic solvents as etching reagent 89
12.2 Alkaline etching 89
12.3 Acidic etching 89
12.4 Etching with different NHCs 91
12.4.1 IPr 91
12.4.2 BIiPr 91
12.5 Etching of different metals 94
13 XRD Measurements 94
13.1.1 X-ray crystal structure analysis of **CS1** (glo10594) 94
13.1.2 Refinement details for **CS1** 95
13.2.1 X-ray crystal structure analysis of **CS2** (glo10604) 96
13.2.2 Refinement details for **CS2** 97
14 Mass Spectrometry of Etching Solutions 99
15 DFT Studies 104
15.1 Coordinates of optimized geometries 110
Supplementary References 130

1 Chemical Synthesis

The following reaction conditions apply for all reaction unless otherwise noted. All reactions were stirred using oven-dried PTFE-coated magnetic stir bars with a rotation speed of 750 rpm. Solids were weighed under ambient conditions, liquids handled with pipettes or syringes. Reaction temperatures are reported as the temperature of metal heating blocks or cooling baths surrounding the reaction vessel. The following solvents were purchased from ACROS Organics, Sigma-Aldrich and Fischer Scientific and used from a solvent purification system, which dried HPLC grade solvents over alumina under a positive pressure of Argon according to Grubb's procedure: tetrahydrofuran (THF), toluene, acetonitrile (MeCN) and dichloromethane (CH_2Cl_2). Other solvents were also purchased from ACROS Organics, Sigma-Aldrich and Fischer Scientific and stored over activated molecular sieves. Solvents for standard work-up or purification procedures were simply purified by distillation (*n*-pentane, *n*-hexane, CH_2Cl_2, EtOAc, Et_2O).

Starting materials were bought from commercial suppliers such as ABCR, Acros Organics, Alfa Aesar, BLD Pharm, Carbolution, Combi-Blocks, Fisher Scientific, Fluka, Fluorchem, Merck, Sigma-Aldrich, Strem Chemicals, or TCI and used as received. Air or moisture sensitive compounds were stored and handled in an argon filled glovebox with oxygen and water levels below 0.5 ppm.

Products were purified by flash column chromatography on Merck silica gel (40–63 mesh) utilizing a positive pressure. Suitable purification conditions were evaluated with thin-layer chromatography (tlc) on silica gel 60 F254 aluminum plates from Merck. Compounds were visualized by UV light with 254 nm. Solvents were removed using rotary evaporators under reduced pressure at 40 °C followed by high vacuum (below 1 mbar) at room temperature.

^{1}H-, ^{13}C-, ^{11}B, ^{19}F, and ^{31}P-NMR spectra were recorded at room temperature on a Avance II 400 MHz, Agilent DD2 500 MHz or Agilent DD2 600 MHz. Chemical shifts (δ) are given in ppm. Residual solvent signals were used as references for 1H- and 13C{1H}-NMR spectra: $CDCl_3$ (δ_H 7.26 ppm, δ_C 77.16 ppm), CD_3OD (δ_H 4.78 ppm, δ_C 49.15 ppm), CD_2Cl_2 (δ_H 5.32 ppm, δ_C 53.84 ppm), D_2O (δ_H 4.79 ppm). Spectra were analyzed with MestReNova from Mestrelab Research S.L. and multiplicity described with standard abbreviations.

ESI accurate masses were measured on a Orbitrap Exploris 120 (Thermo-Fisher Scientific, Bremen) with loop injection.

1.1 BIMeH·HCO_3

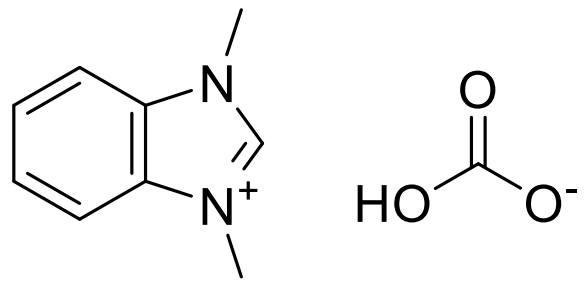

1,3-dimethyl-1*H*-benzo[*d*]imidazol-3-ium hydrogen carbonate

1,3-dimethyl-1H-benzo[d]imidazol-3-ium iodide (BIMeH·I) was synthesized according to known literature procedure.[1] 1,3-dimethyl-1H-benzo[d]imidazol-3-ium hydrogen carbonate (BIMeH·HCO_3) was synthesized via anion exchange according to adapted literature procedures.[1] A column was charged with ion exchanger resin Amberlyst A-26 to a length of roughly 30 – 35 cm. The column was then prepared by washing with KOH (aq., 1.0 M, 200 mL), followed by washing with H_2O until the column had a neutral pH value. NH_4HCO_3 (8 g/400 mL H_2O) were added until pH = 8, respectively. The column ran dry and flushed with MeOH for five column volumes. Close attention was paid for tight packing. BIMeH·I (1.2 g) was added in MeOH (50 mL) and left running slowly through the column (1 drop per second) with MeOH. Product flux was

tracked via thin layer chromatography. Product containing eluates were carefully evaporated at 30 °C and 80 mbar. (Note: At higher temperature or higher vacuum, quantitative BIMeH_2O formation was observed.) CH_2Cl_2 was added and product precipitated upon sonication. After filtration, the white powder was washed with CH_2Cl_2 (200 ml) and pentane (100 ml) and dried under reduced pressure (660 mg, 72 % yield).

^{1}H NMR (400 MHz, MeOD) δ = 7.94 – 7.90 (m, 2H), 7.73 – 7.69 (m, 2H), 4.12 (s, 6H).

^{13}C NMR (101 MHz, MeOD) δ = 161.37, 133.51, 128.15, 114.15, 33.67.

HRMS (ESI): m/z calculated for $[BIMeH]^+$ 147.0917, found 147.0921.

Pushrod (thermal evaporation coupled to EI): m/z calculated for $[BIMe]^{+\cdot}$ 146.1, found 146.1.

1.2 IPr·CO_2

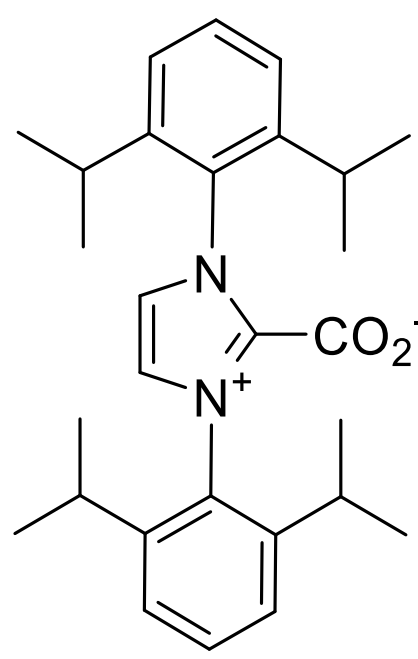

1,3-bis(2,6-diisopropylphenyl)-1*H*-imidazol-3-ium-2-carboxylate

1,3-bis(2,6-diisopropylphenyl)-1H-imidazol-3-ium chloride (IPrH·Cl) was synthesized according to a known literature procedure.[2] 1,3-(2,6-diisopropylphenyl)-1H-imidazol-3-ium-2-carboxylate (IPr·CO_2) was synthesized according to adapted literature procedures:[2] IPrH·Cl (12.4 g, 29.2 mmol, 1 eq.) and potassium *tert*-butoxide (4.8 g, 43 mmol, 1.5 eq.) were placed in a Schlenk flask under Argon. At -78 °C, 200 ml dry and degassed toluene were added. Whilst warming slowly to rt., the mixture was stirred for 2.5 hours. The mixture was filtered over Celite under Argon. The filter was washed with more toluene (two times 200 ml). Still under Argon, CO_2 was infiltrated into the yellow carbene solution and white precipitate was formed. After one hour, the white precipitate was filtered under ambient condition, washed with 100 ml toluene and 200 ml n-hexane, dried under reduced pressure and stored inside a glovebox fridge (10.2 g, 23.6 mmol, 80 % yield).

^{1}H NMR (400 MHz, CD_2Cl_2) δ = 7.53 (t, *J* = 7.8 Hz, 2H), 7.33 (d, *J* = 7.8 Hz, 4H), 7.15 (s, 2H), 2.52 (p, *J* = 6.9 Hz, 4H), 1.24 (dd, *J* = 24.1, 6.8 Hz, 24H).

^{13}C NMR (101 MHz, CD_2Cl_2) δ = 145.34, 132.96, 131.09, 124.56, 122.90, 29.57, 24.49, 23.56.

Pushrod (thermal evaporation coupled to EI): m/z calculated for $[IPr]^{+\cdot}$ 388.29, found 388.4.

1.3 IPrH·HCO_3

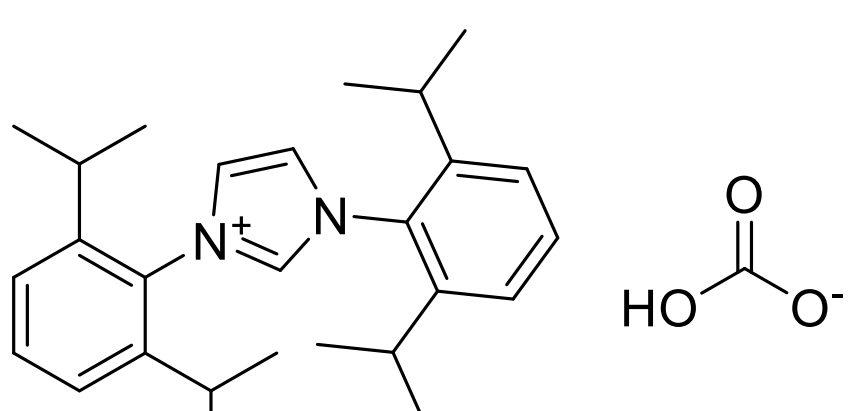

1,3-bis(2,6-diisopropylphenyl)-1*H*-imidazol-3-ium hydrogen carbonate

1,3-bis(2,6-diisopropylphenyl)-1H-imidazol-3-ium hydrogen carbonate (IPrH·HCO_3) was synthesized via anion exchange according to adapted literature procedures and similar to BIMeH·HCO_3 (120 mg, 58 % yield).[1]

^{1}H NMR (400 MHz, MeOD) δ = 8.26 (s, 2H), 7.66 (t, *J* = 7.9 Hz, 2H), 7.50 (d, *J* = 7.9 Hz, 4H), 2.46 (p, *J* = 6.8 Hz, 4H), 1.32 (d, *J* = 6.8 Hz, 12H), 1.24 (d, *J* = 7.0 Hz, 12H).

^{13}C NMR (101 MHz, MeOD) δ = 146.55, 133.39, 131.50, 127.53, 125.99, 30.42, 24.75, 23.84.

Pushrod (thermal evaporation coupled to EI): m/z calculated for $[IPr]^{+\cdot}$ 388.29, found 388.4.

HRMS (ESI): m/z calculated for $[IPrH]^{+}$ 389.2951, found 389.2916.

1.4 BIiPrH·HCO_3

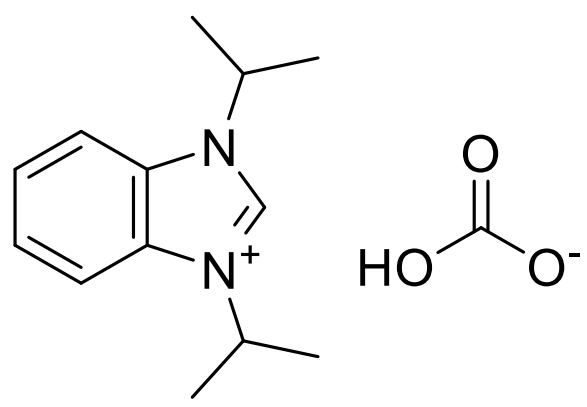

1,3-diisopropyl-1*H*-benzo[*d*]imidazol-3-ium hydrogen carbonate

1,3-diisopropyl-1H-benzo[d]imidazol-3-ium iodide (BIiPrH·I) was synthesized according to known literature procedure.[1] 1,3-diisopropyl-1H-benzo[d]imidazol-3-ium hydrogen carbonate (BIiPrH·HCO_3) was synthesized via anion exchange according to adapted literature procedures and similar to BIMeH·HCO_3 (55 mg, 69 % yield).[1]

^{1}H NMR (400 MHz, MeOD) δ = 8.02 (dd, *J* = 6.3, 3.2 Hz, 2H), 7.70 (dd, *J* = 6.4, 3.1 Hz, 2H), 5.06 (hept, *J* = 6.8 Hz, 2H), 1.72 (d, *J* = 6.8 Hz, 12H).

^{13}C NMR (101 MHz, MeOD) δ = 161.38, 132.55, 128.15, 114.97, 52.80, 22.11.

1.5 IMe·CO_2

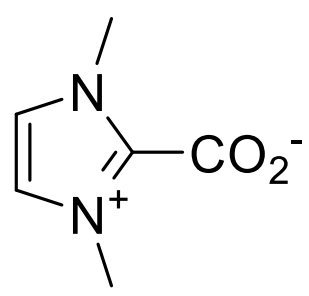

1,3-dimethyl-1*H*-imidazol-3-ium-2-carboxylate

1,3-dimethyl-1H-imidazolium iodide (IMeH·I) was synthesized according to a known literature procedure.[3,4] 1,3-dimethyl-1H-imidazol-3-ium-2-carboxylate (IMe·CO_2) was synthesized according to adapted literature procedures:[3,4] IMeH·I (10 g, 44.6 mmol, 1 eq.) and potassium *tert*-butoxide (6.0 g, 53.6 mmol, 1.2 eq.) were placed in a Schlenk flask under Argon. At -78 °C, 250 ml dry and degassed tetrahydrofuran (THF) were added. Whilst warming slowly to rt., the mixture was stirred for 2.5 hours. The mixture was filtered over Celite under Argon. The filter was washed with more THF (two times 250 ml). Still under Argon, CO_2 was infiltrated into the yellow carbene solution and white precipitate was formed. After one hour, the white precipitate was filtered under ambient condition, washed with 100 ml THF and 400 ml CH_2Cl_2, dried under reduced pressure and stored inside a glovebox fridge (5.3 g, 37.8 mmol, 85 % yield).

^{1}H NMR (400 MHz, D_2O) δ = 7.36 (s, 2H), 3.97 (s, 6H).

^{13}C NMR (101 MHz, D_2O) δ = 161.48, 158.29, 123.07, 36.76.

Pushrod (thermal evaporation coupled to EI): m/z calculated for $[IMe]^{+\cdot}$ 96.1, found 96.2.

HRMS (ESI): m/z calculated for $[IMeH]^{+}$ 97.0760, found 97.0760.

1.6 Triazolium **1**

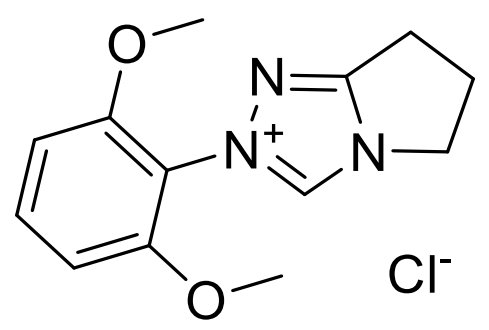

2-(2,6-dimethoxyphenyl)-6,7-dihydro-5*H*-pyrrolo[2,1-*c*][1,2,4]triazol-2-ium chloride

2-(2,6-dimethoxyphenyl)-6,7-dihydro-5H-pyrrolo[2,1-c][1,2,4] triazol-2-ium chloride (Triazolium**1**) was synthesized according to known literature procedure.[5]

^{1}H NMR (400 MHz, $CDCl_3$) δ = 11.18 (s, 1H), 7.47 (t, *J* = 8.6 Hz, 1H), 6.67 (d, *J* = 8.5 Hz, 2H), 4.89 (t, *J* = 7.4 Hz, 2H), 3.81 (s, 6H), 3.22 (t, *J* = 7.8 Hz, 2H), 2.87 (p, *J* = 7.7 Hz, 2H).

1.7 $[Cu(BIMe)_2]PF_6$

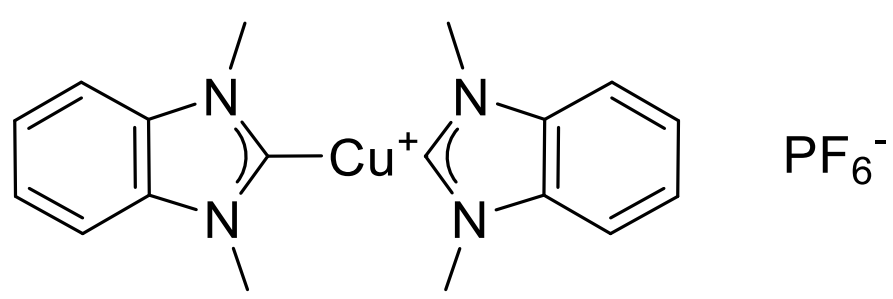

bis(1,3-dimethyl-2,3-dihydro-1*H*-benzo[*d*]imidazol-2-yl)copper(I) hexafluorophosphate(V)

bis(1,3-dimethyl-2,3-dihydro-1H benzo[d]imidazole-2-yl)copper(I) hexafluorophosphate(V) ($[Cu(BIMe)_2]PF_6$) was synthesized according to literature.[6,7]

^{1}H NMR (400 MHz, $CDCl_3$) δ = 7.10 (dd, *J* = 5.7, 3.2 Hz, 2H), 6.97 (dd, *J* = 5.8, 3.3 Hz, 2H), 3.42 (s, 6H).

^{13}C NMR (101 MHz, $CDCl_3$) δ = 154.68, 130.04, 121.20, 107.32, 27.16.

HRMS (ESI): m/z calculated for $[Cu(BIMe)_2]^+$ 355.0978, found 355.0977.

1.8 BIMeO

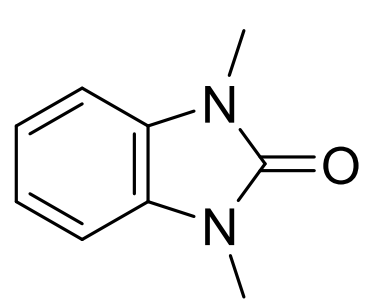

1,3-dimethyl-1,3-dihydro-2*H*-benzo[*d*]imidazol-2-one

1,3-dimethyl-1,3-dihydro-2H-benzo[d]imidazol-2-one (BIMeO) was synthesized according to literature.[8,9]

^{1}H NMR (400 MHz, $CDCl_3$) δ = 7.09 (dd, *J* = 5.7, 3.2 Hz, 2H), 6.96 (dd, *J* = 5.7, 3.2 Hz, 2H), 3.41 (s, 6H).

HRMS (ESI): m/z calculated for $[BIMeO+H]^+$ 163.0866, found 163.0866. m/z calculated for $[BIMeO+Na]^+$ 185.0685, found 185.0683.

1.9 Aldehyde **2**

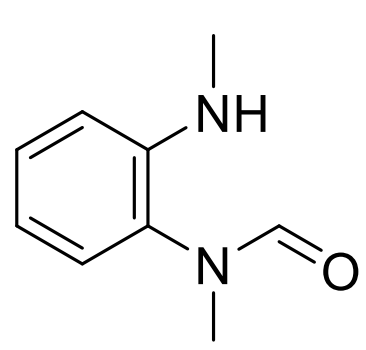

N-methyl-*N*-(2-(methylamino)phenyl)formamide

N-methyl-N-(2-(methylamino)phenyl)formamide ($BIMeH_2O$ or aldehyde **2**) was synthesized according to literature.[10]

^{1}H NMR (400 MHz, $CDCl_3$) δ = 8.05 (s, 1H), 7.26 – 7.15 (m, 1H), 6.93 (dd, *J* = 8.4, 1.3 Hz, 1H), 6.69 – 6.62 (m, 2H), 3.86 (s, 1H), 3.08 (d, *J* = 0.8 Hz, 3H), 2.79 (d, *J* = 5.3 Hz, 3H).

^{13}C NMR (101 MHz, $CDCl_3$) δ = 164.13, 145.81, 129.99, 128.45, 127.07, 116.87, 111.00, 32.23, 30.35.

HRMS (ESI): m/z calculated for [**2**+H]$^+$ 165.1022, found 165.1020. m/z calculated for [**2**+Na]$^+$ 187.0842, found 187.0839.

2 NMR Spectra

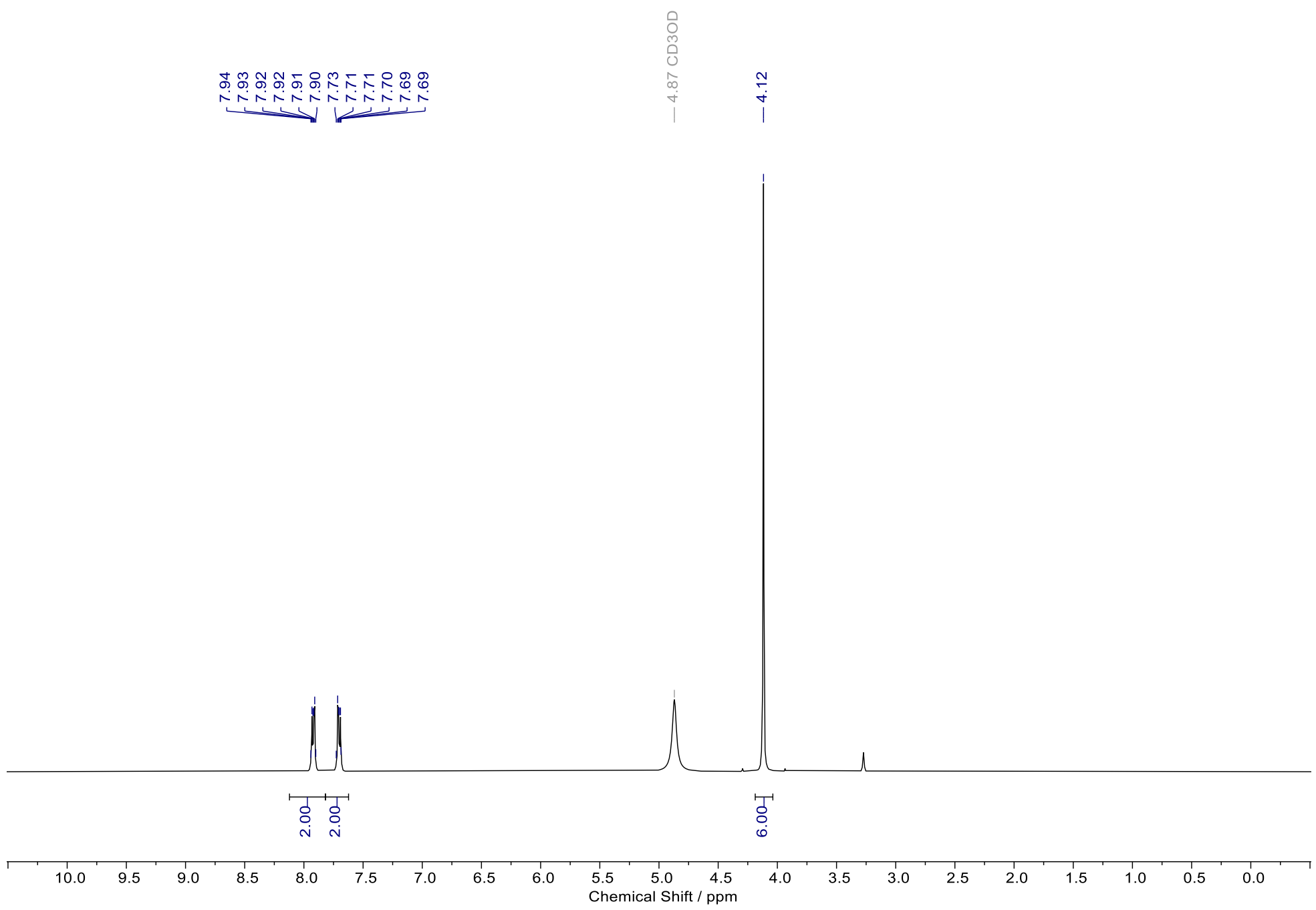

Figure S1: ^{1}H NMR (400 MHz, CD_3OD) spectrum of BIMeH·HCO_3.

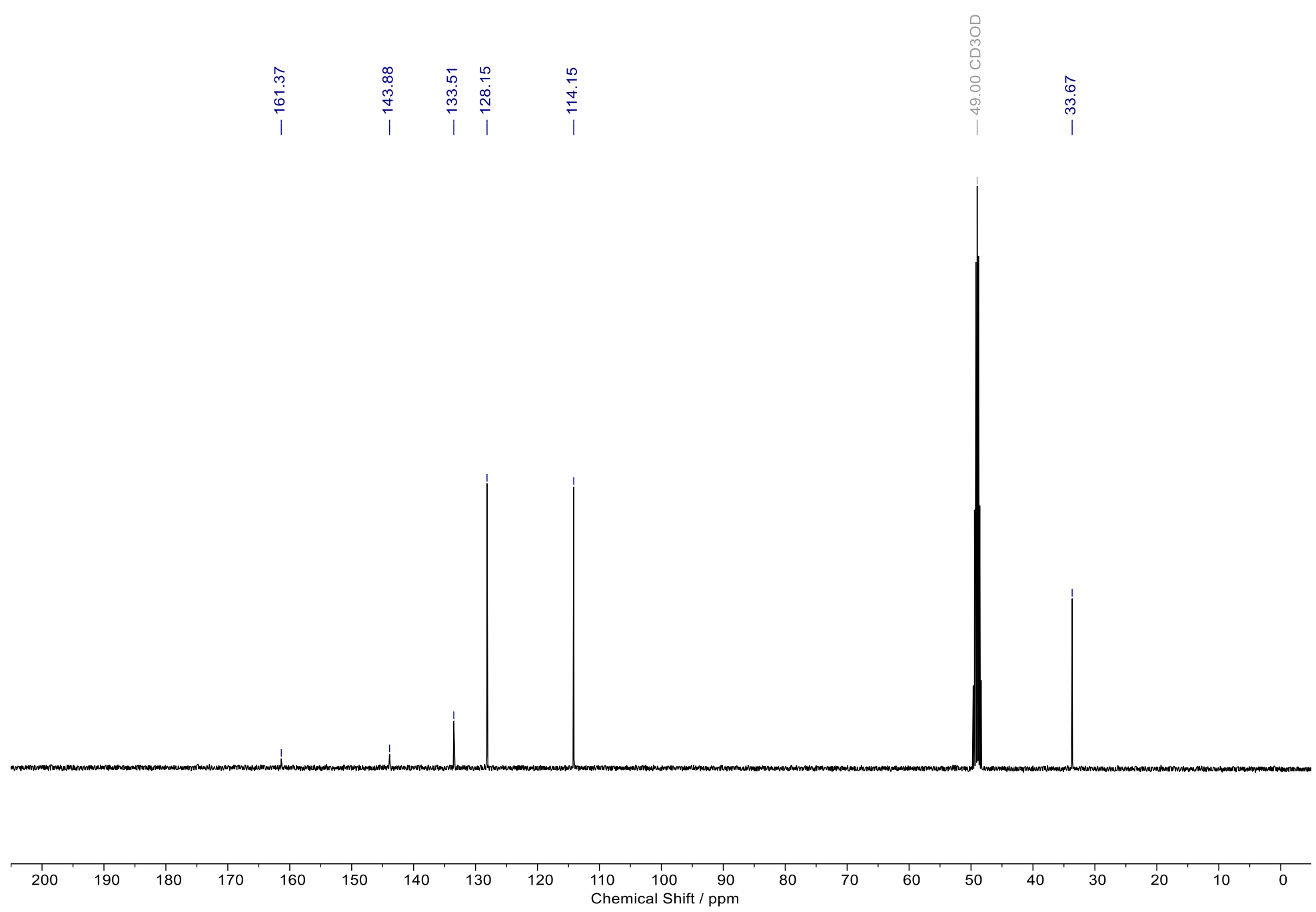

Figure S2: ^{13}C NMR (101 MHz, CD_3OD) spectrum of BIMeH·HCO_3.

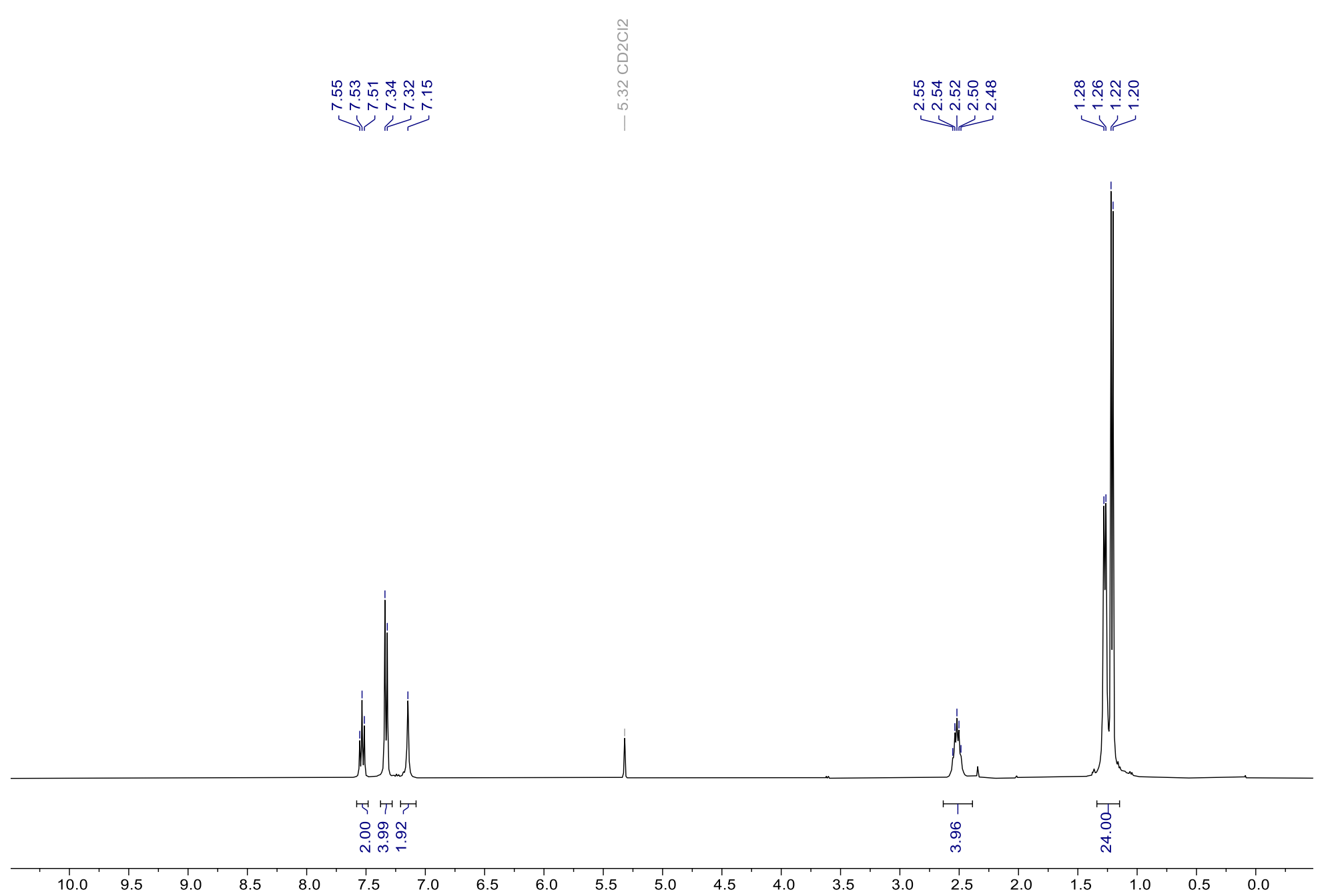

Figure S3: ^{1}H NMR (400 MHz, CD_2Cl_2) spectrum of IPr·CO_2.

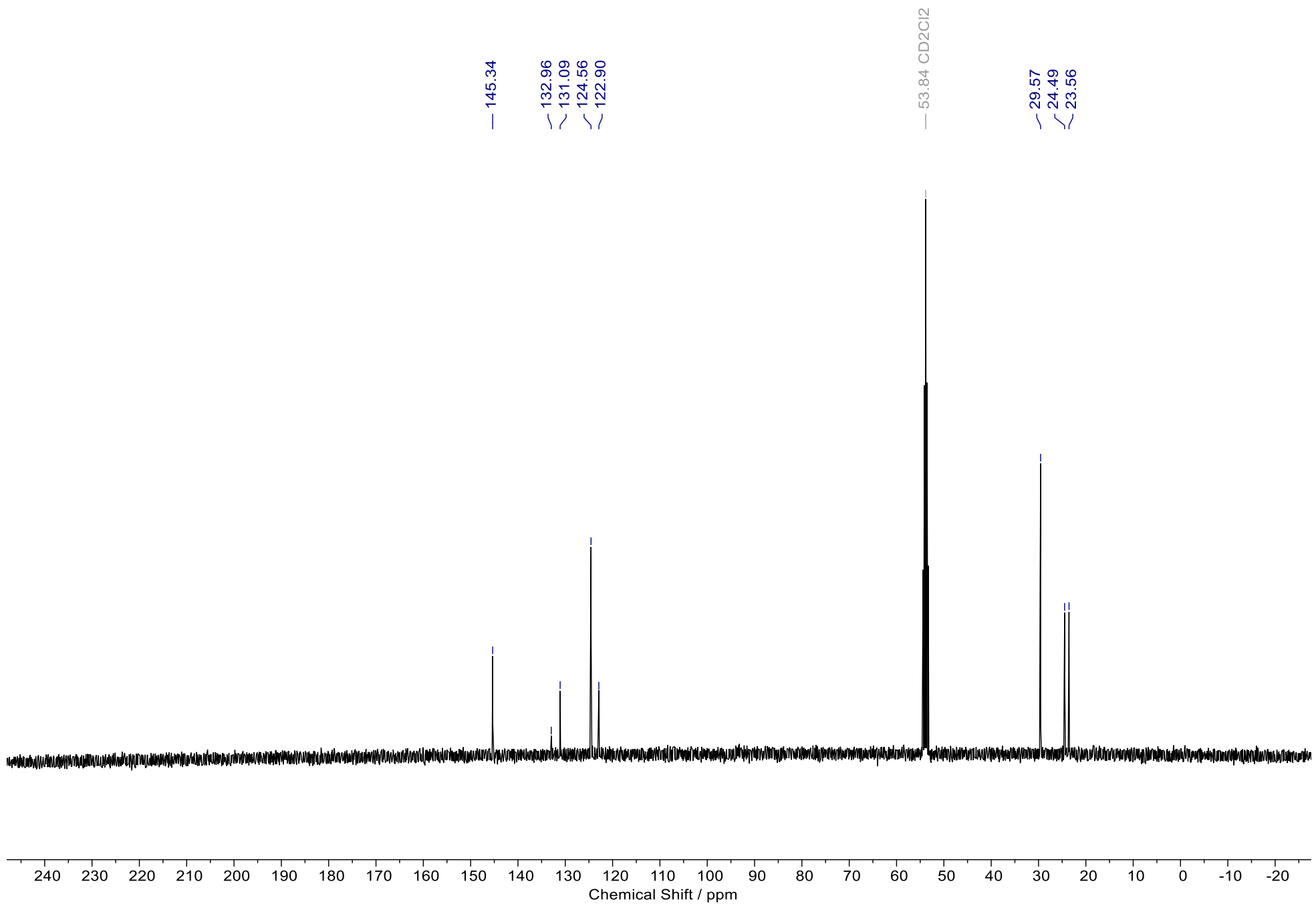

Figure S4: ^{13}C NMR (101 MHz, CD_2Cl_2) spectrum of IPr·CO_2.

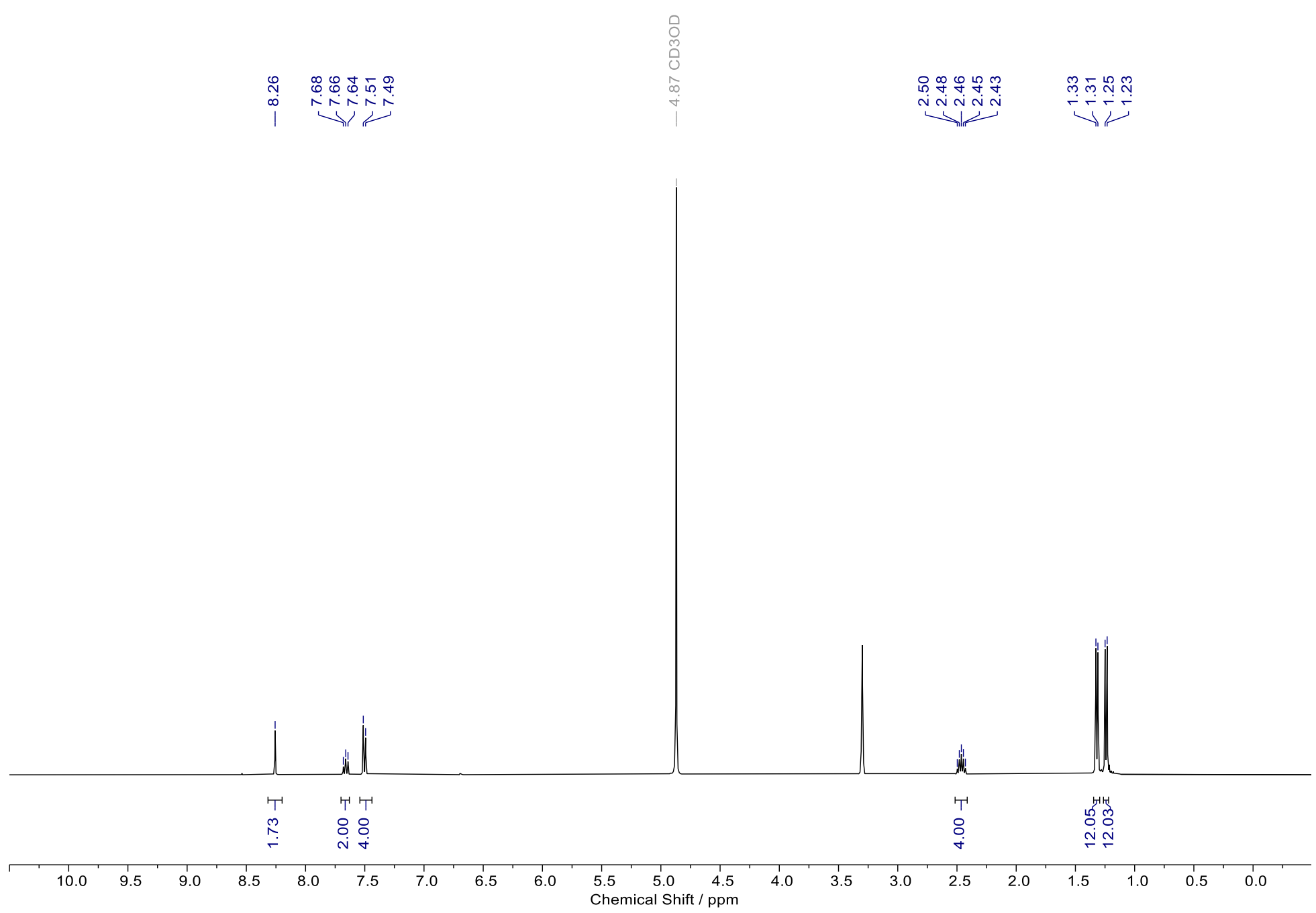

Figure S5: ^{1}H NMR (400 MHz, CD_3OD) spectrum of IPrH·HCO_3.

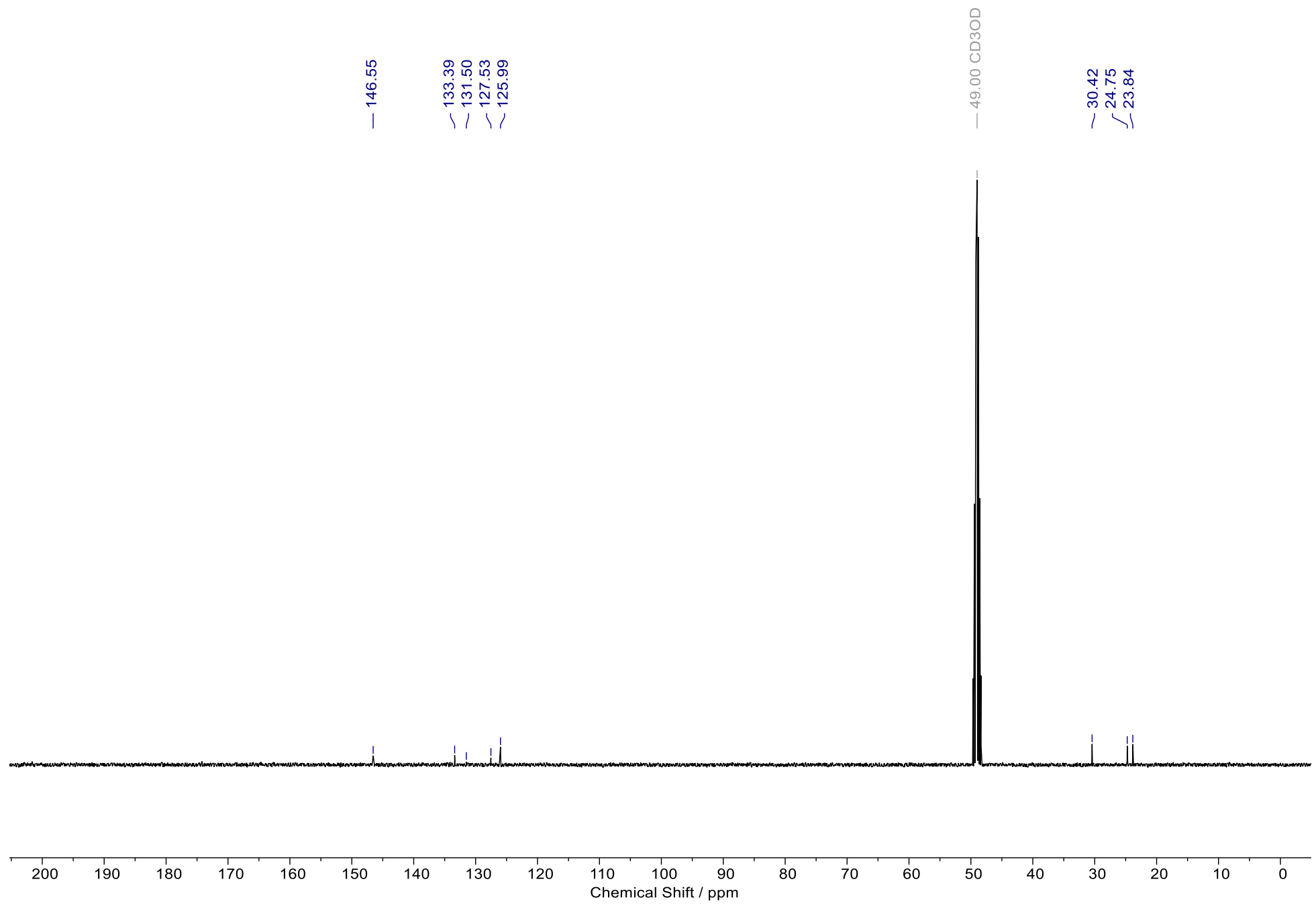

Figure S6: ^{13}C NMR (101 MHz, CD_3OD) spectrum of IPrH·HCO_3.

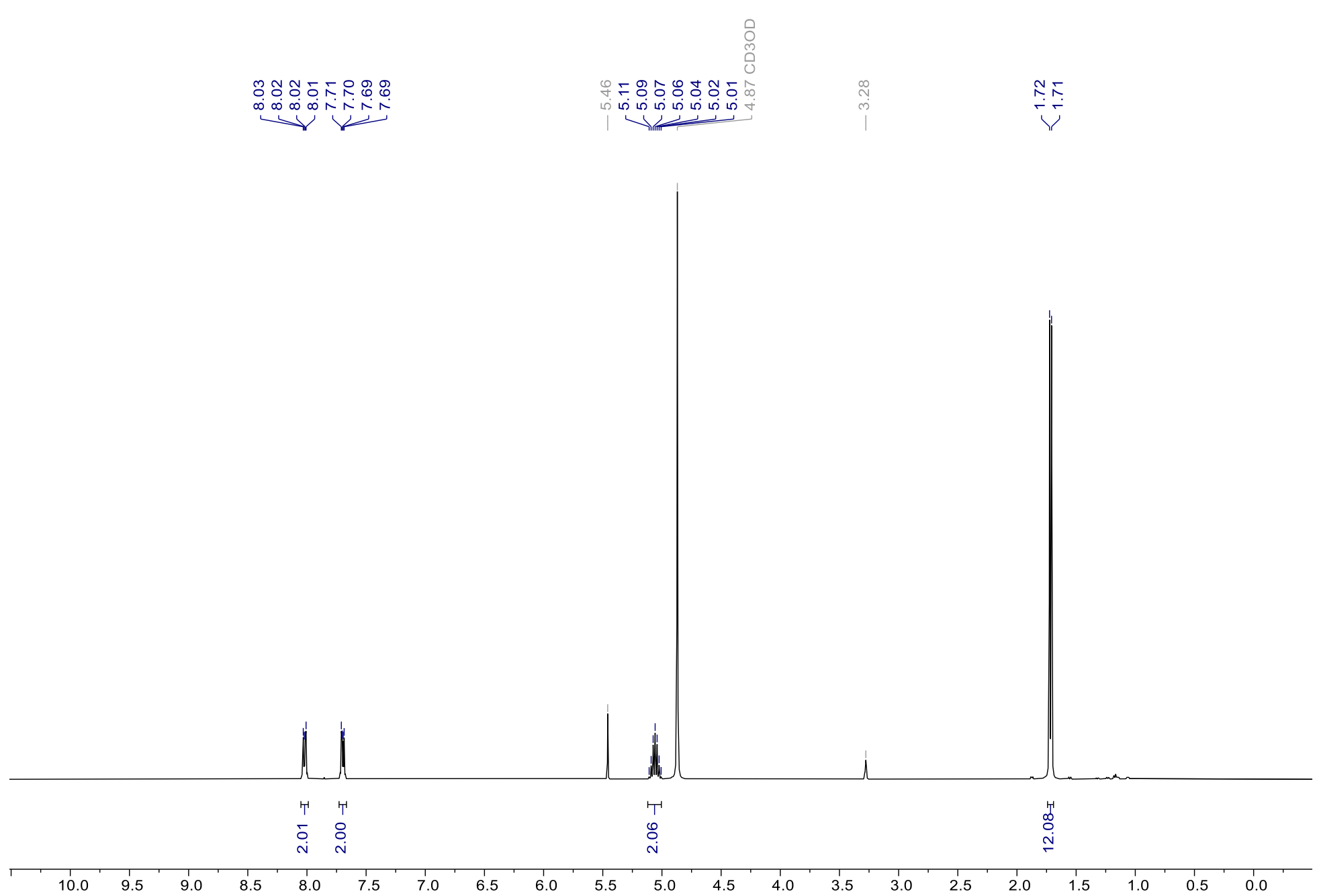

Figure S7: ^{1}H NMR (400 MHz, CD_3OD) spectrum of BIiPrH·HCO_3.

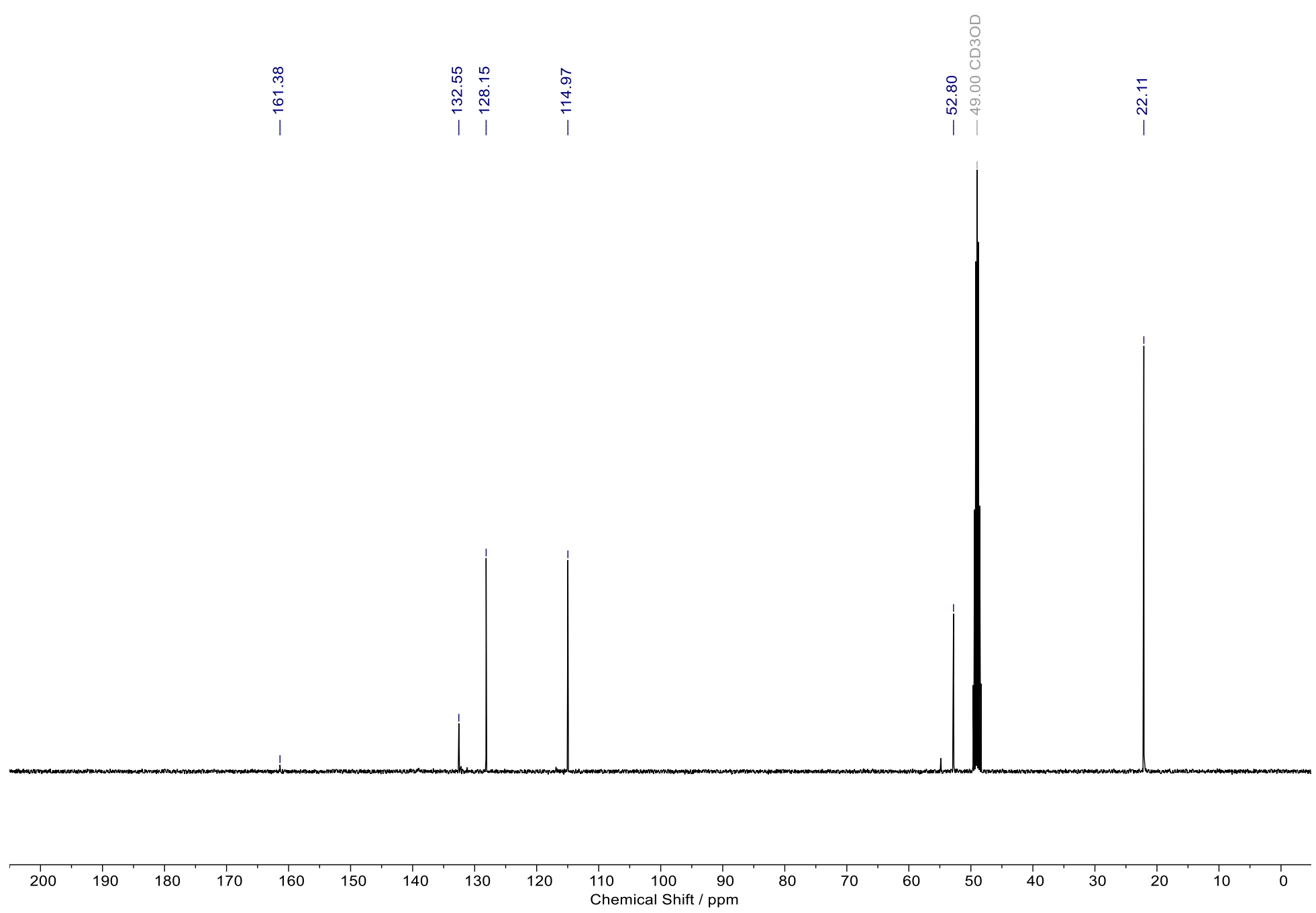

Figure S8: ^{13}C NMR (101 MHz, CD_3OD) spectrum of BIiPrH·HCO_3.

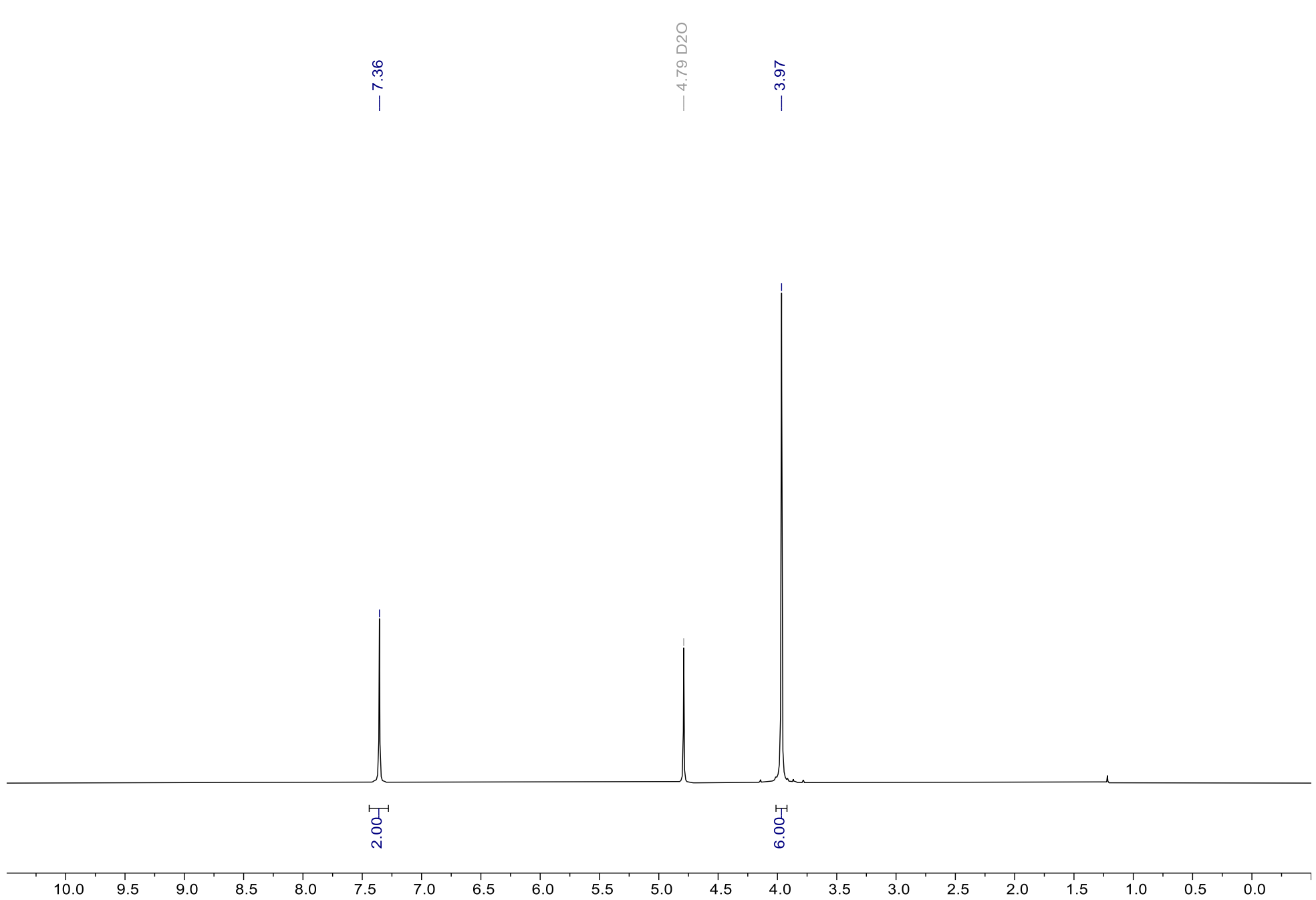

Figure S9: ^{1}H NMR (400 MHz, D_2O) spectrum of IMe·CO_2.

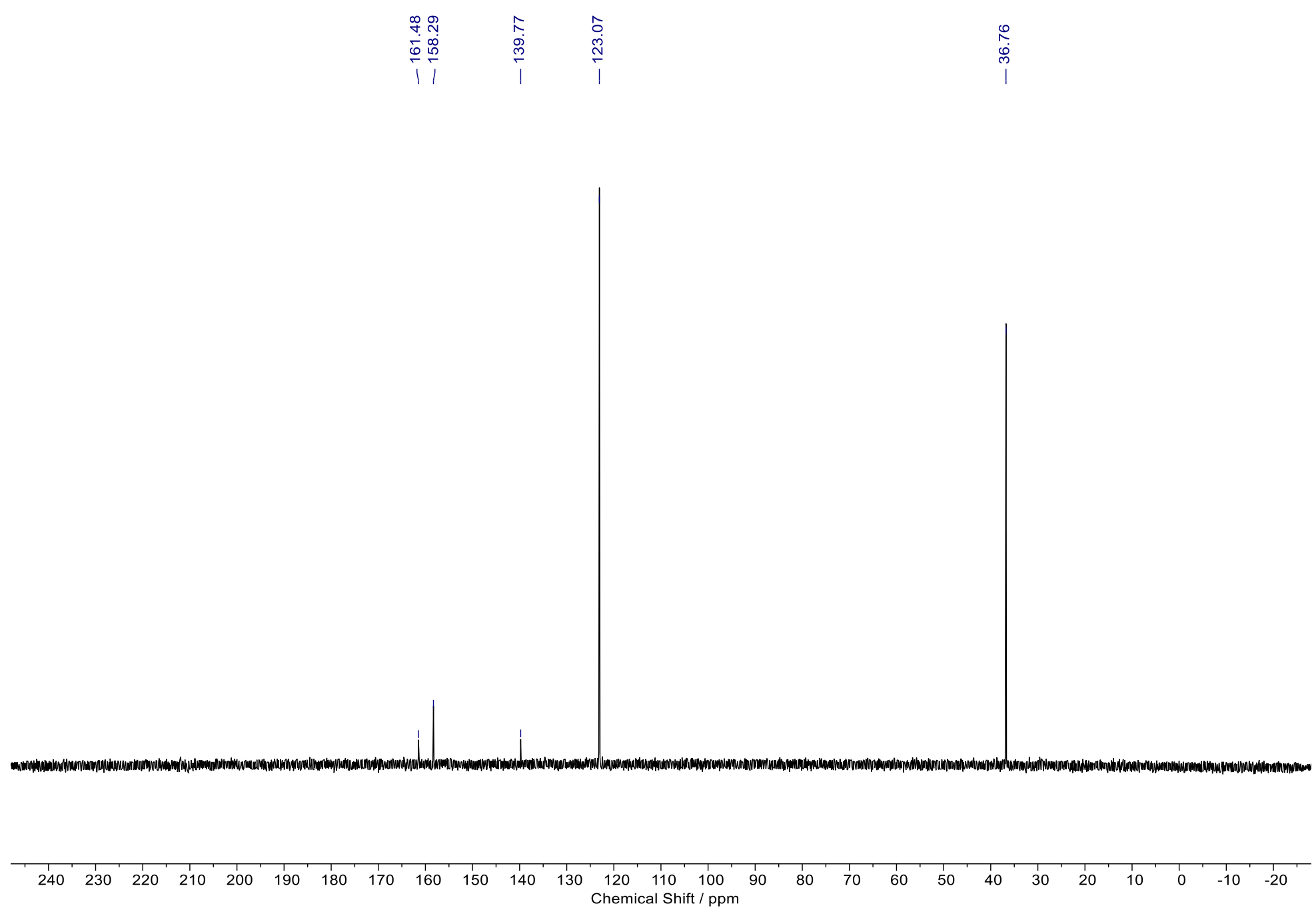

Figure S10: ^{13}C NMR (101 MHz, D_2O) spectrum of IMe·CO_2.

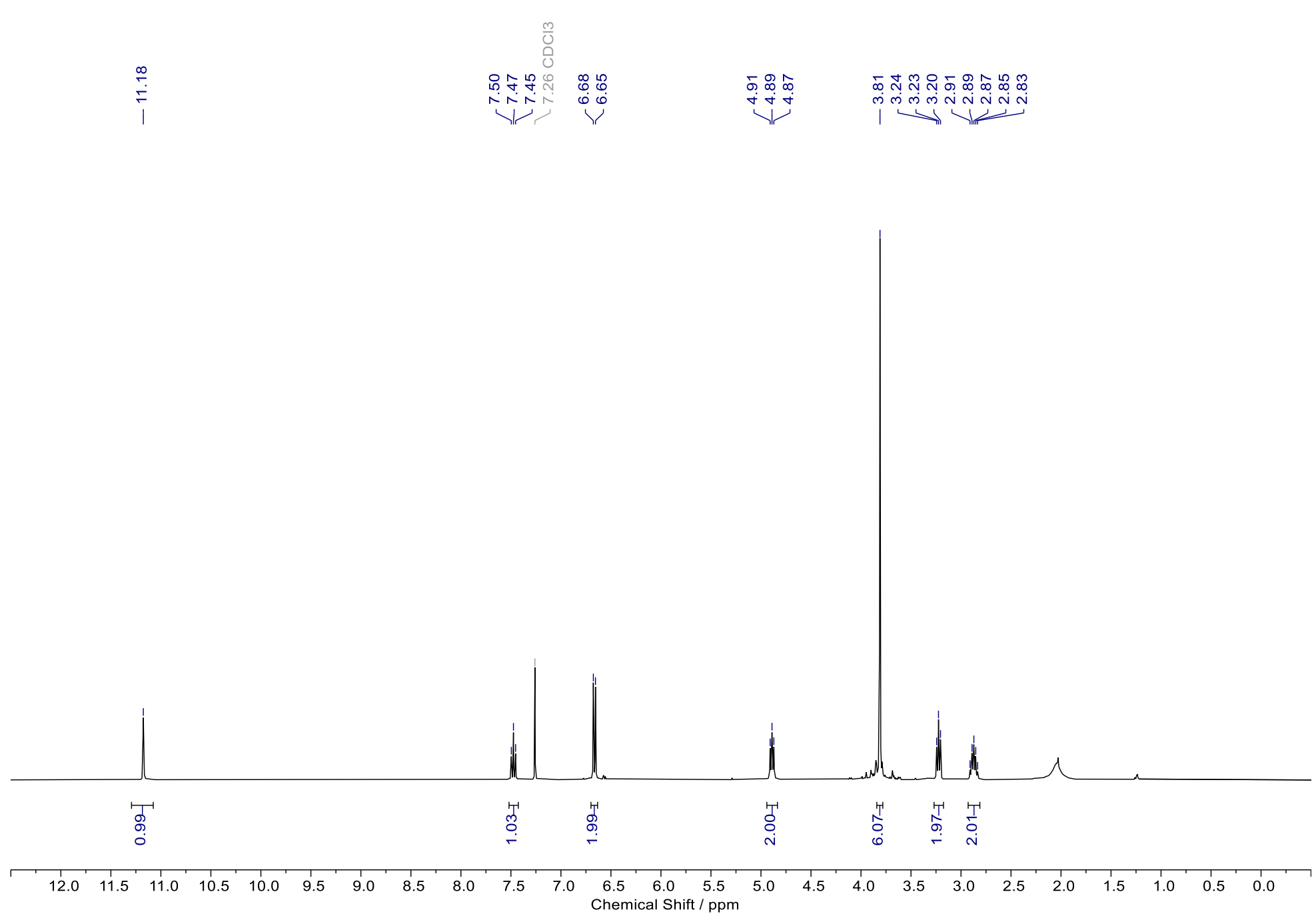

Figure S11: ^{1}H NMR (400 MHz, $CDCl_3$) spectrum of 2-(2,6-dimethoxyphenyl)-6,7-dihydro-5H-pyrrolo[2,1-c][1,2,4] triazol-2-ium chloride.

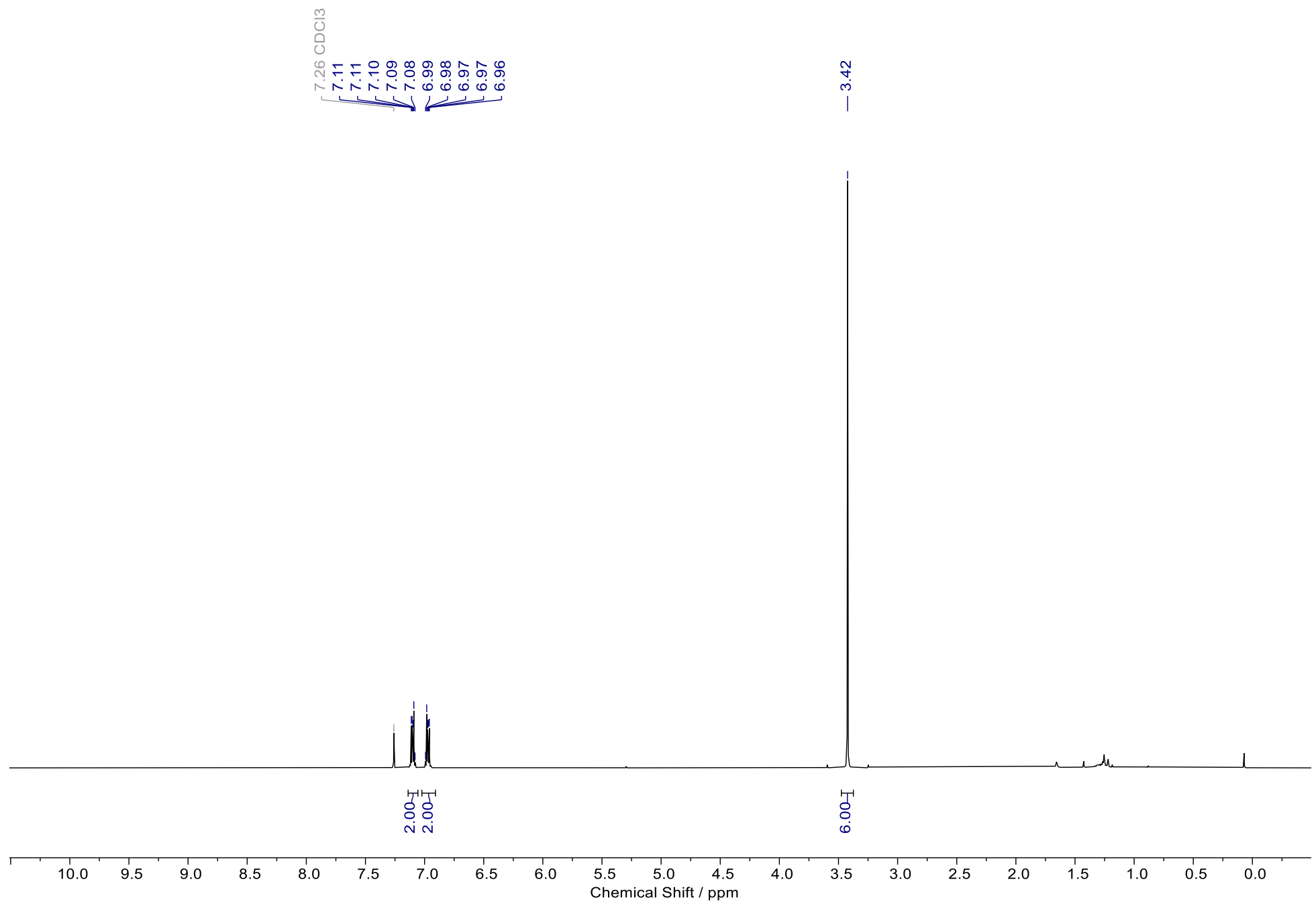

Figure S12: ^{1}H NMR (400 MHz, $CDCl_3$) spectrum of $[Cu(BIMe)_2]PF_6$.

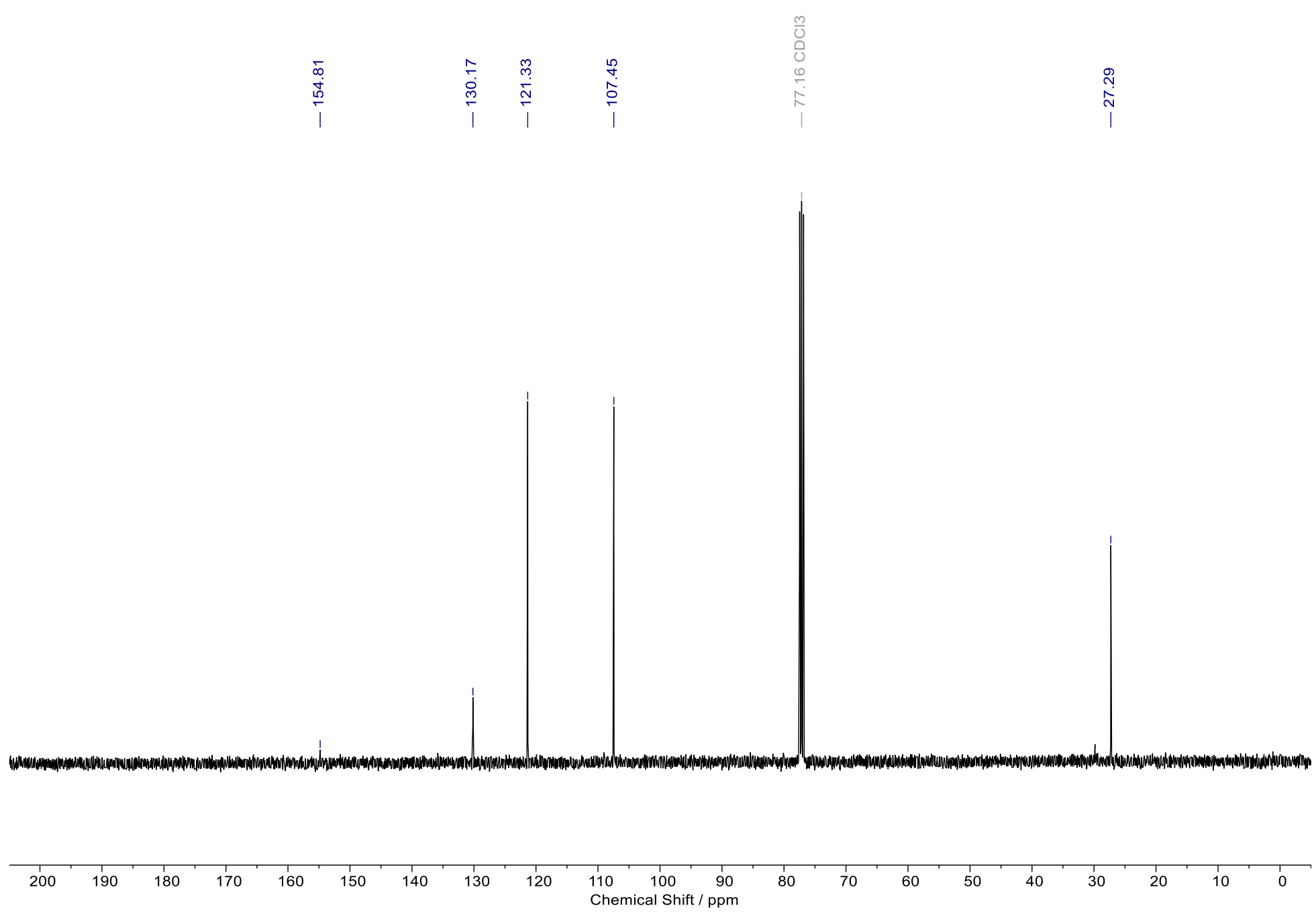

Figure S13: ^{13}C NMR (101 MHz,$CDCl_3$) spectrum of $[Cu(BIMe)_2]PF_6$.

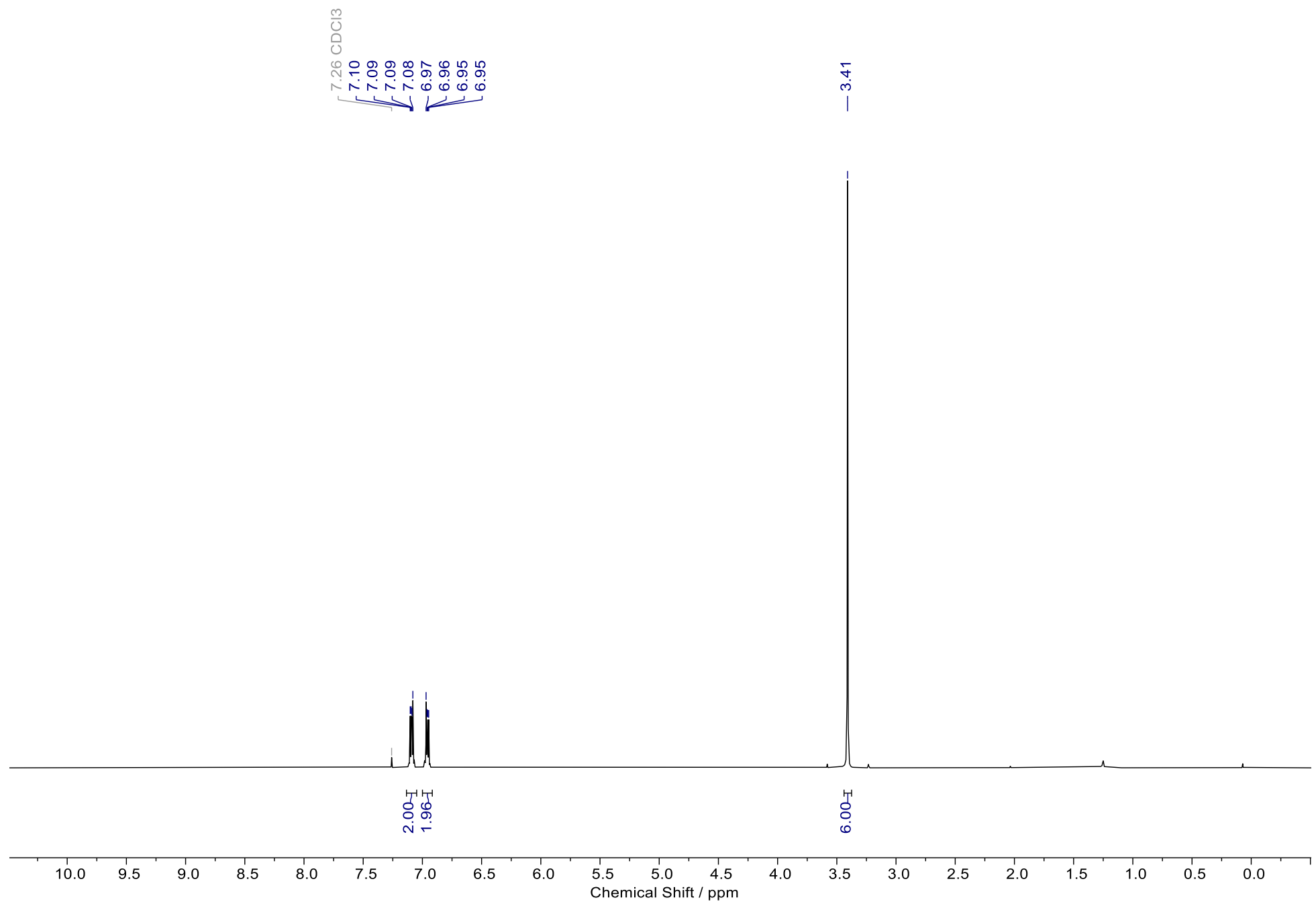

Figure S14: ^{1}H NMR (400 MHz, $CDCl_3$) spectrum of BIMeO.

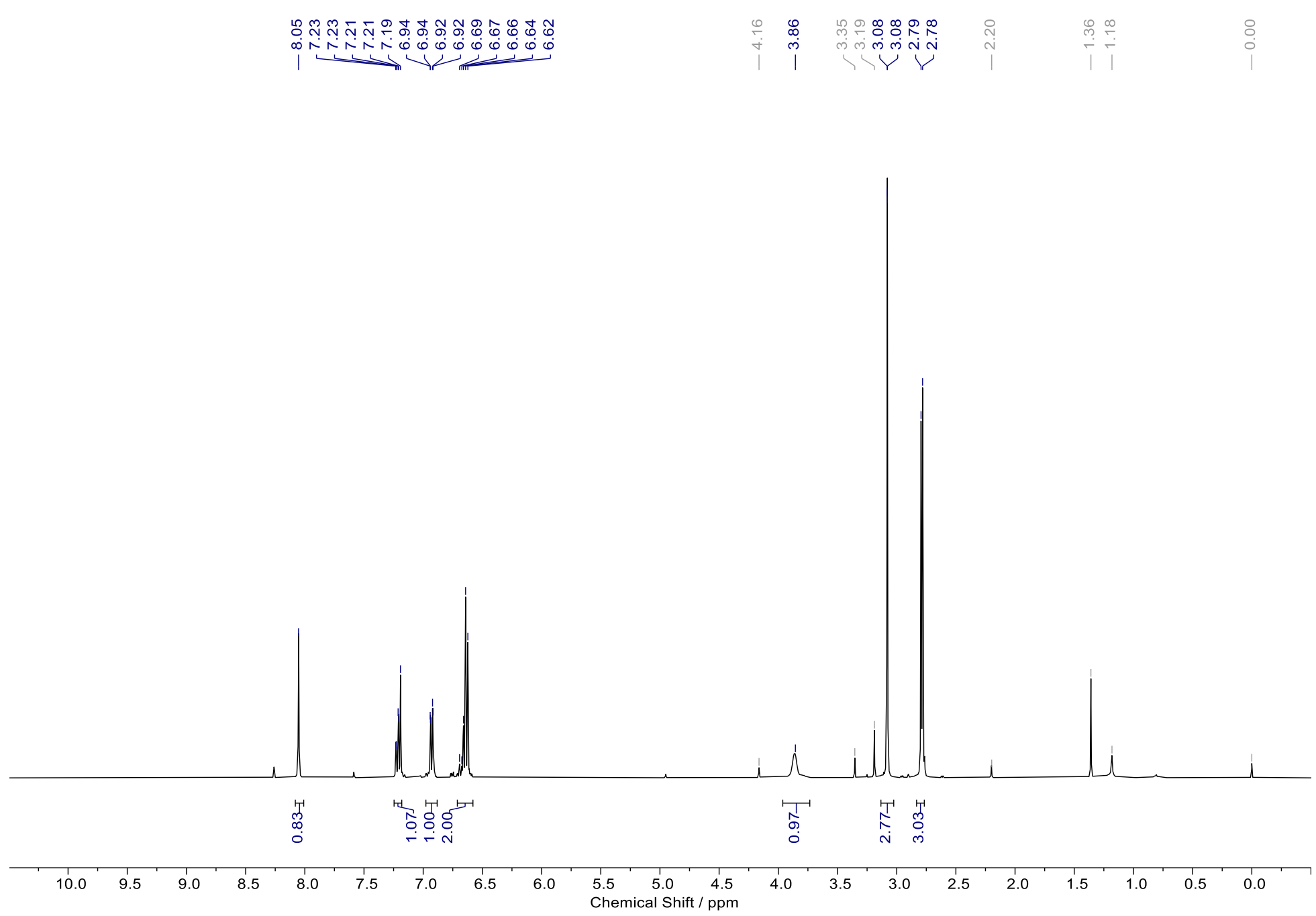

Figure S15: ^{1}H NMR (400 MHz, $CDCl_3$) spectrum of aldehyde **2**.

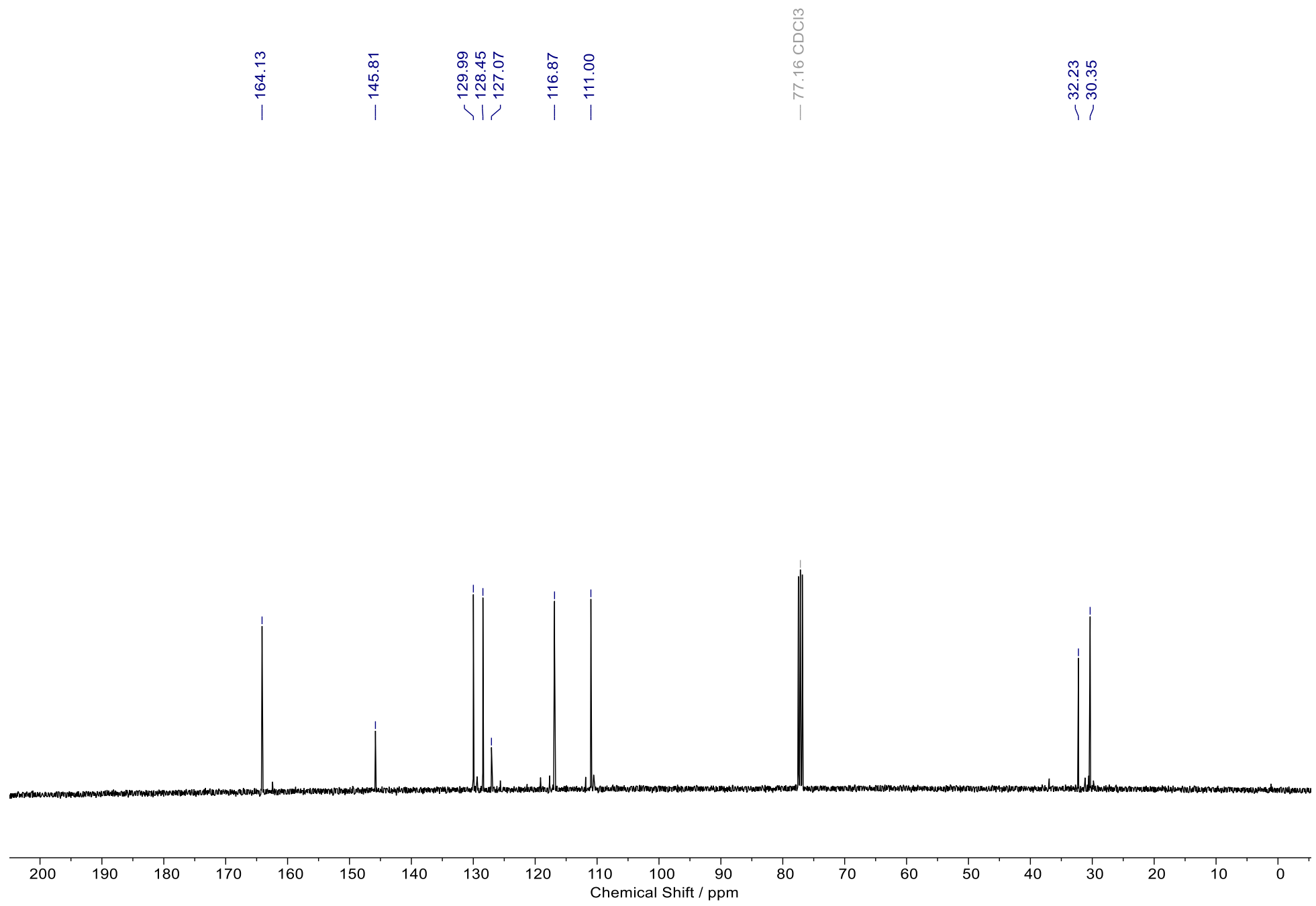

Figure S16: ^{13}C NMR (101 MHz, $CDCl_3$) spectrum of aldehyde **2**.

3 General Surface Modification Procedures

For surface modification, exclusively LC-MS grade solvents were used. Thin layer copper substrates were purchased from Angstrom Engineering: Borofloat 33 Microscopy slides (75x25x1 mm), Titanium adhesion layer (3 nm), copper top-layer (100 nm). Prior to use, the surfaces were cut into 5x5 mm^2 slides.

3.1 Cleaning as pretreatment

The flat metal surfaces were cleaned by heating them in 2 ml of MeOH to 50-60 °C for 1 h. Subsequently, they were rinsed with few ml of MeOH, dried under reduced pressure (1 mbar) and stored in glass vials under Argon.

3.2.1 Liquid phase modification

If not otherwise mentioned, the metal surface was placed in a glass vial (metal side facing up) and the NHC precursor solution (or suspension) was added (5 mg in 2 ml). The vial was sealed under ambient conditions and stored at room temperature for the indicated time of modification. Afterwards, the surface was rinsed thoroughly with iPrOH, dried under reduced pressure (1 mbar) and stored in glass vials under Argon.

3.2.2 Vapor phase modification

Physical vapor phase deposition was carried out with a house-made setup (see Figure S17). In order to clean the chamber, its valve was connected to a vacuum pump (0.1-1.0 mbar) whilst the setup was heated to T > 150 °C for one hour. After cooling down to room temperature, the chamber was flooded with Argon, 2 mg of the respective NHC precursor was loaded in the crucible and metal surfaces were placed on the sample stage. The chamber was flushed with Argon, evacuated (~0.1 mbar) and the valve was closed. Now, the setup was heated to 100 °C for 30 minutes (p < 5 mbar). During cooling down, vacuum was restored. At room temperature, the chamber was flushed with Argon to take out the sample, which was rinsed with MeOH, dried under reduced pressure (1 mbar) and stored in glass vials under Argon.

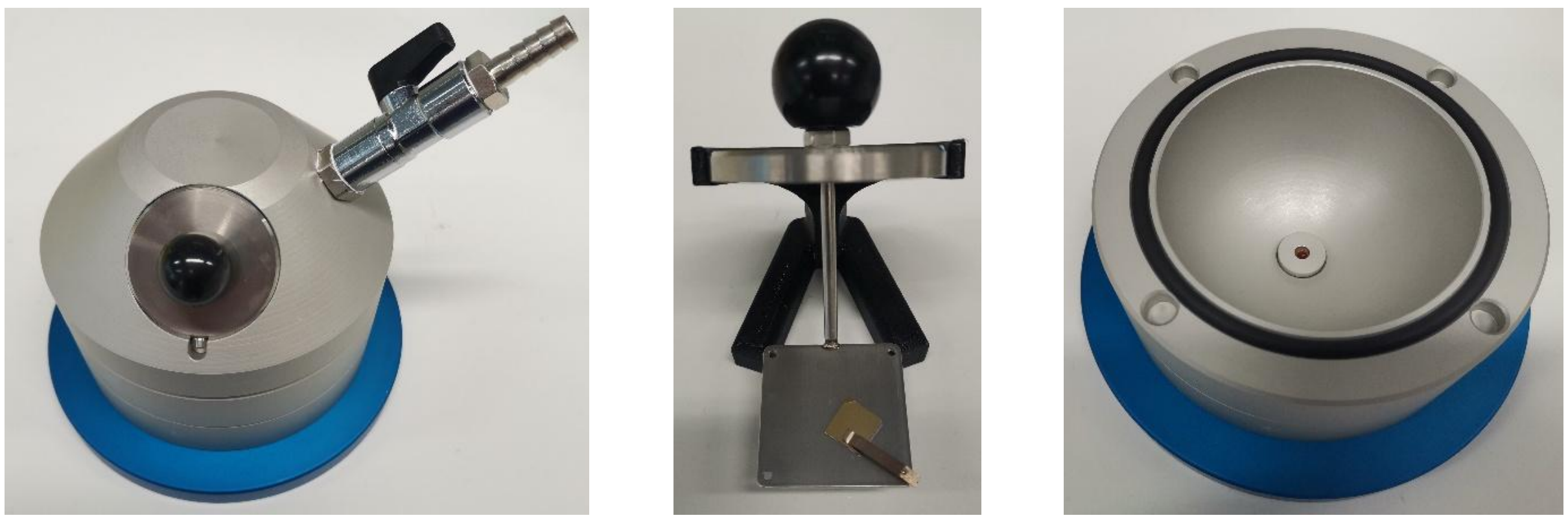

Figure S17: Physical vapor phase deposition chamber made from aluminum with Teflon sealings. The bottom is compatible with standard laboratory heating plates. It contains a sample stage, a small crucible and a vacuum/Argon valve.

4 XPS Measurements

Table S1: XPS measurement parameters.

	Pass energy [eV]	Dwell time [ms]	Step size [eV]	No. of scans
Survey	200	100	0.90	2
Au 4f	50	50	0.05	5
Au 4f (quant.)	100	100	0.05	10
Cu 2p	50	50	0.05	5
Cu 2p (quant.)	100	100	0.05	10
Pt 4f	50	50	0.05	5
Pt 4f (quant.)	100	100	0.05	10
N 1s	100	100	0.05	10
C 1s	50	50	0.05	5
O 1s	50	50	0.05	5

XPS measurements were performed on a Thermo Fisher Scientific K-Alpha instruments (Organisch-Chemisches Institut, Corrensstr. 36, 48149 Münster). All spectra were referenced to Cu $2p_{3/2}$ (932.8 eV), Pt $4f_{7/2}$ (71.2 eV) or Au $4f_{7/2}$ (84.0 eV), respectively. The spectra were

analyzed using Avantage 5.9925 (Thermo Fisher Scientific). Data visualization was done with Origin Pro 2023.

The XPS measurement parameters are shown in Table S1. An Al K_α X-ray source was used (12 kV filament, hv = 1486.7 eV), the spot size was 100 µm (72 W max.), X-ray incidence and take-off angle amount to 60° and 0° respectively (rel. to surface normal). For charge compensation metallic clips connected the samples to the grounded sample stage and an argon flood gun was used.

For each series of XPS measurements, also a blank metal surface was analyzed as reference. Only if the N 1s spectrum was found to be empty, the associated spectra of functionalized spectra are included and discussed. This way, signals due to e.g., contaminated solvents or glassware were omitted to be included in this work. Exemplary, one analysis of these non-functionalized surfaces is included (see Figure S47).

4.1 Solvent screening

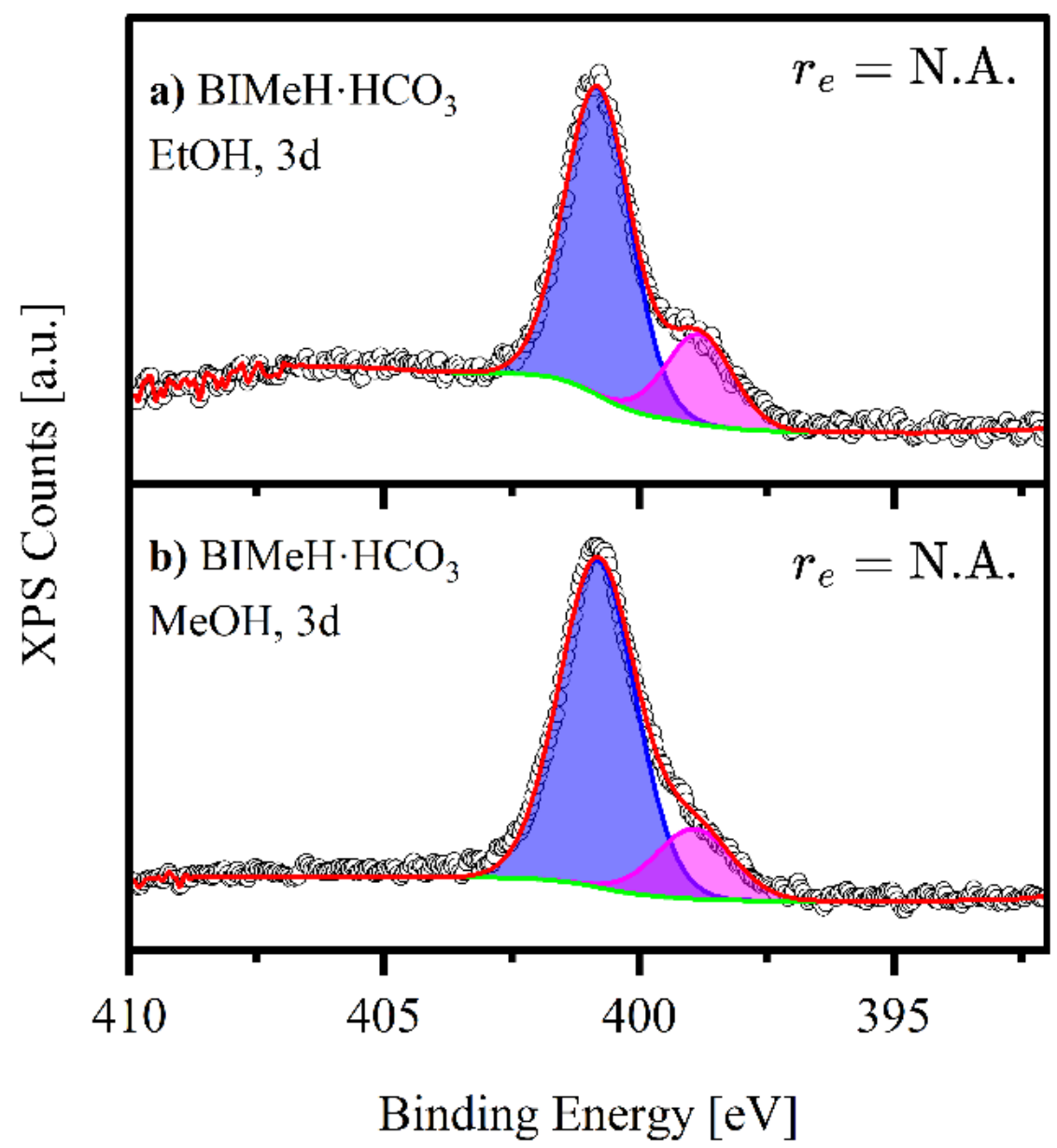

Figure S18: N 1s XPS spectra of BIMe on copper deposited from MeOH and EtOH after increased modification time. Other element and survey spectra can be found in Section 4.9 including detailed deposition procedures.

We observe a highly solvent dependent etching and functionalization behavior (see Figure 4): In non-polar organic solvents like THF (e) and n-hexane (f) little to no surface functionalization and no etching was observed, as the NHC precursor was poorly dissolved. In polar aprotic solvents like CH_2Cl_2 (c), $C_2H_4Cl_2$ (b) and MeCN (a) XPS shows two distinct N 1s peaks after 3 h of modification and the 100 nm thick Cu substrates were dissolved within two days of modification yielding an etching rate re of more than 50 nm per day. In case of the protic solvent MeOH, only one broad N 1s signal was found (or three overlapping signals – depending on interpretation).

In Figure S18, the deposition time in alcohol (MeOH and EtOH, respectively) was extended resulting in similar N 1s spectra as observed when using shorter deposition times in aprotic, polar solvents like CH_2Cl_2. Still, no sign of etching (fading of thin layer substrate or Cu ions in the solution based on ESI) was detected.

4.2 Light and temperature dependency

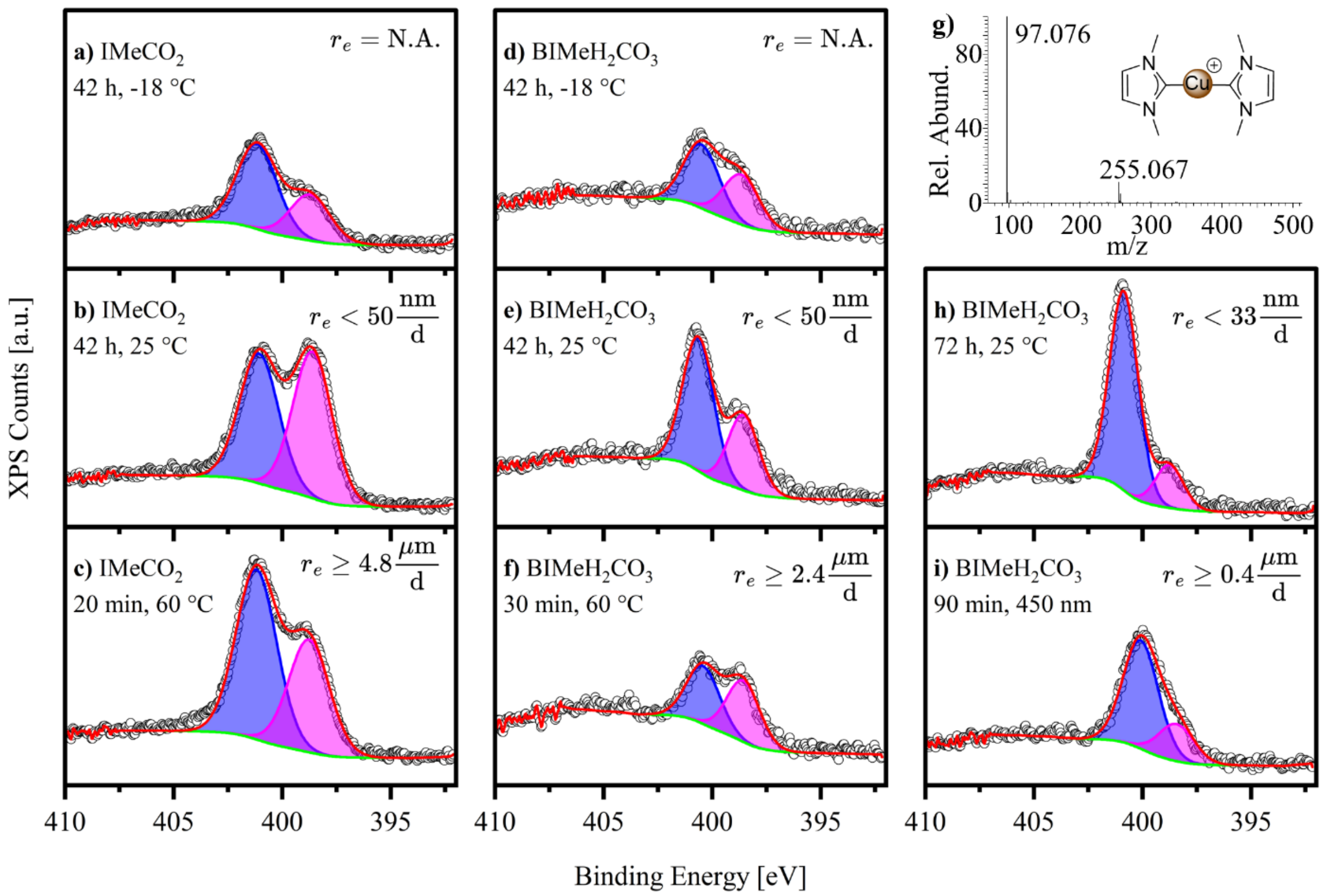

Figure S19: N 1s XPS spectra of IMe and BIMe on Cu deposited from CH_2Cl_2 at varying temperatures. All samples were modified in the dark except for sample i, which was irradiated with a 450 nm LED. g) shows the ESI of the etching solution obtained from the modification in c). Full ESI spectrum can be found in Figure S94. Other element and survey spectra can be found in section 4.10 including deposition procedures.

Looking at low temperature experiments in the freezer first (IMe in Figure S19 a, BIMe in d), cooling slows down the copper functionalization as reflected in comparatively low N 1s signal intensities. Further, cooling slows down any etching too. Working at room temperature, avoidance of light apparently slows down etching too and allows modification times of 42 h (IMe in Figure S19 b, BIMe in e) and even 72 h (Figure S19 h) without fully dissolving the 100 nm thin substrates. The elongated modification times generally increase N 1s signal intensities. In case of 72 h, the signal to noise ratio exceeds prior measurements and this entry was used to calculate the surface coverage to proof monolayer formation (see section 5). Also, the 400.7 eV seems to suppress the 398.8 eV peak after 72 h of modification (qualitatively similar to the experiment at elongated modification time without oxygen inside the glovebox, see Figure 6 and Figure S46).

Heating to 60 °C increased the etching rates roughly by a factor of 100 in case of IMe·CO_2 (c) and by a factor of 50 in case of BIMeH·HCO_3 (f). The etching solution of c was analyzed by HRMS (nano-spray ESI) and solely imidazolium salt and the bis(NHC) complex were found (g). Also, irradiation with a 450 nm LED led to an increased etching rate at room temperature by a factor of

around 10. Possibly, the common ground between both heat and light enhanced Cu etching could be the induction of an electron-hole separation within the metal.

Figure S19 g indicates that etching solutions do not contain any ionic or easily ionizable species except $[IMe+H]^+$ and $[Cu(IMe)_2]^+$ whilst the N 1s spectra of the Cu surfaces during etching with IMe show two peaks. Combined, this is a strong indication for both XPS peaks to originate from adsorbed IMe in different binding modes.

4.3 Time and concentration dependency

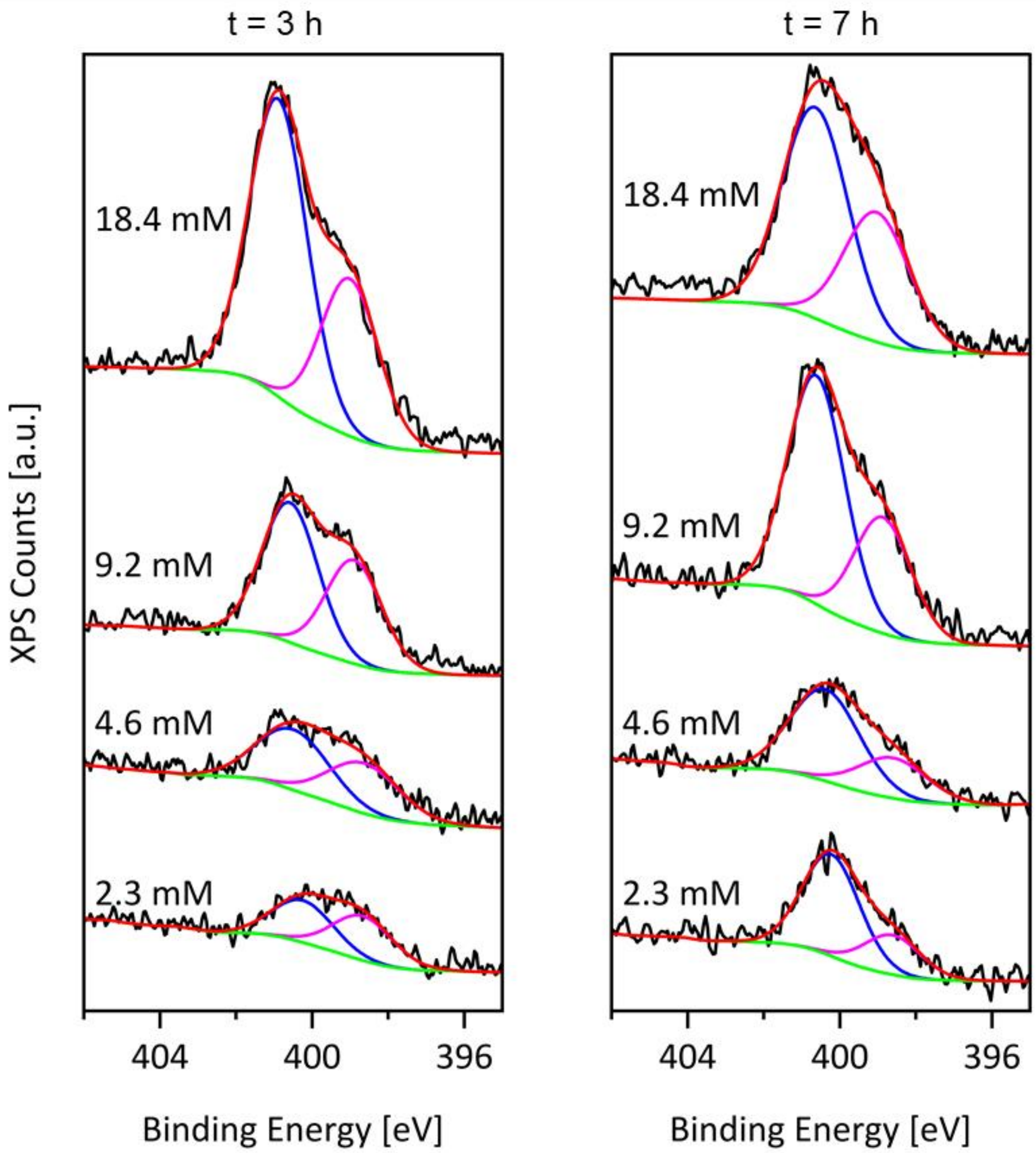

Figure S20: N 1s XPS spectra of copper surfaces modified with BIMe using different deposition times (left 3 h, right 7 h) and various BIMeH·HCO_3 concentrations between 2 and 20 mM. All depicted spectra use the same scale but with an individual and arbitrary offset.

As BIMeH·HCO_3 is only purely soluble in CH_2Cl_2, CH_2Cl_2 MeOH mixtures (2.0 to 0.1 ml) were used for concentration dependent experiments. It is clearly visible in Figure S20, that N 1s XPS signal intensities rise with increasing NHC precursor concentration at a given time. This indicates the speed of NHC monolayer formation to correlate with precursor concentration. At lower

concentrations, an increase in deposition time from 3 to 7 h tends to increase N 1s signal intensity too. In all cases we see two N 1s XPS peaks at around 400.7 and 398.8 eV. Here, we don't observe any significant influence of precursor concentration or deposition time (within the relatively short studied time window) on the observed peak ratio.

4.4 Data associated with Figure 2

Peaks					
**Name**	**Peak BE**	**FWHM eV**	**Area (P) CPS.eV**	**Atomic %**	**Q**
N1s	400.78	1.73	4790.74	73.25	Yes
N1s Scan A	398.71	1.73	1751.89	26.75	Yes

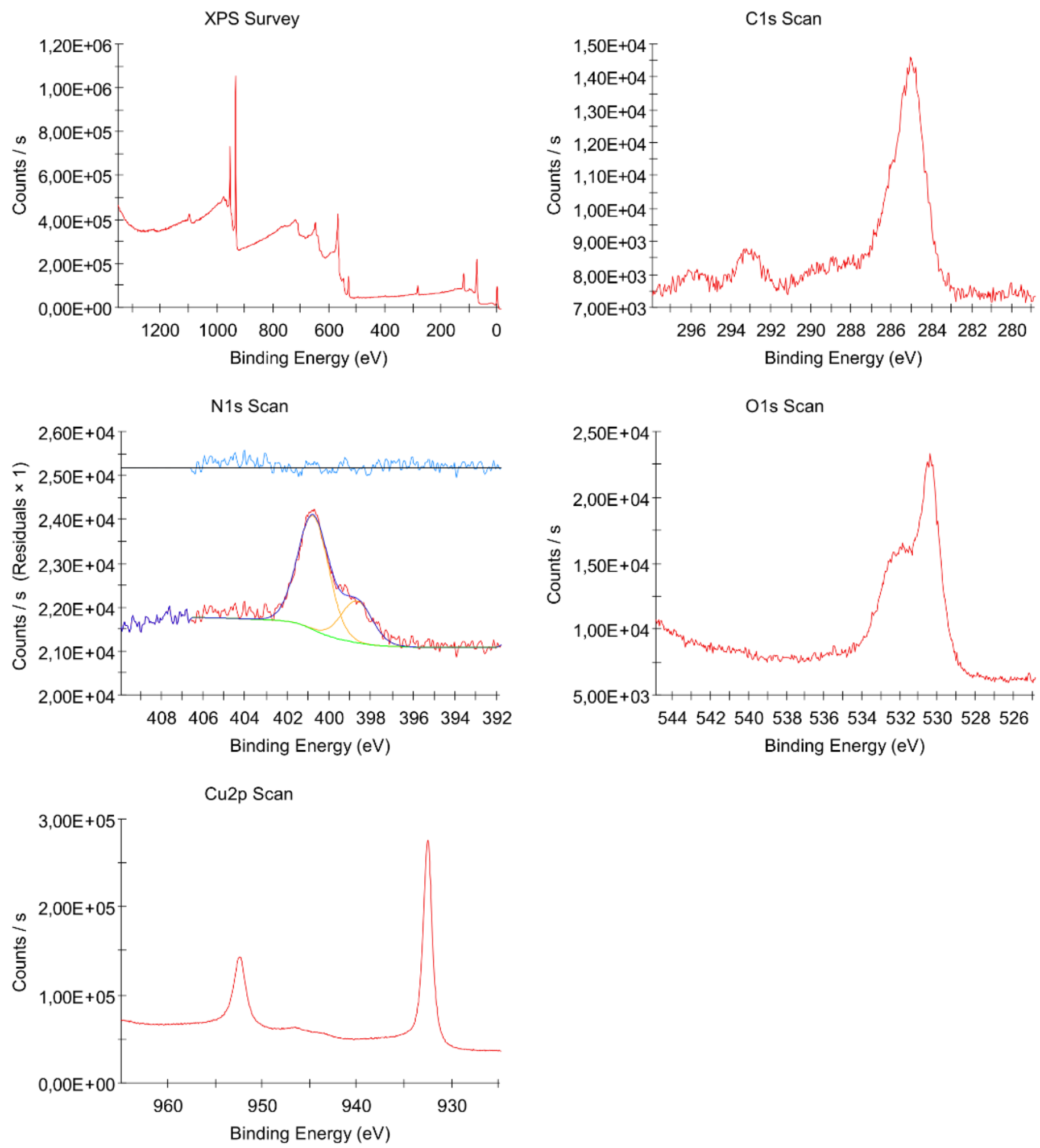

09.02.2026 11:11:00 O:\ANP\GLO_0123_ANP-AB-022\ANP-AB-022 DCM.vgp

Figure S21: Survey and element spectra accompanying the N 1s XPS spectrum displayed in Figure 2 a. Sample preparation details: Cu slide and 5 mg BIMeH·HCO_3 in 1 ml CH_2Cl_2 for 200 minutes. Washed with CH_2Cl_2, MeOH and again CH_2Cl_2.

Peaks					
Name	Peak BE	FWHM eV	Area (P) CPS.eV	Atomic %	Q
N1s	400.99	1.54	10368.72	74.23	Yes
N1s Scan A	398.83	1.54	3604.62	25.77	Yes

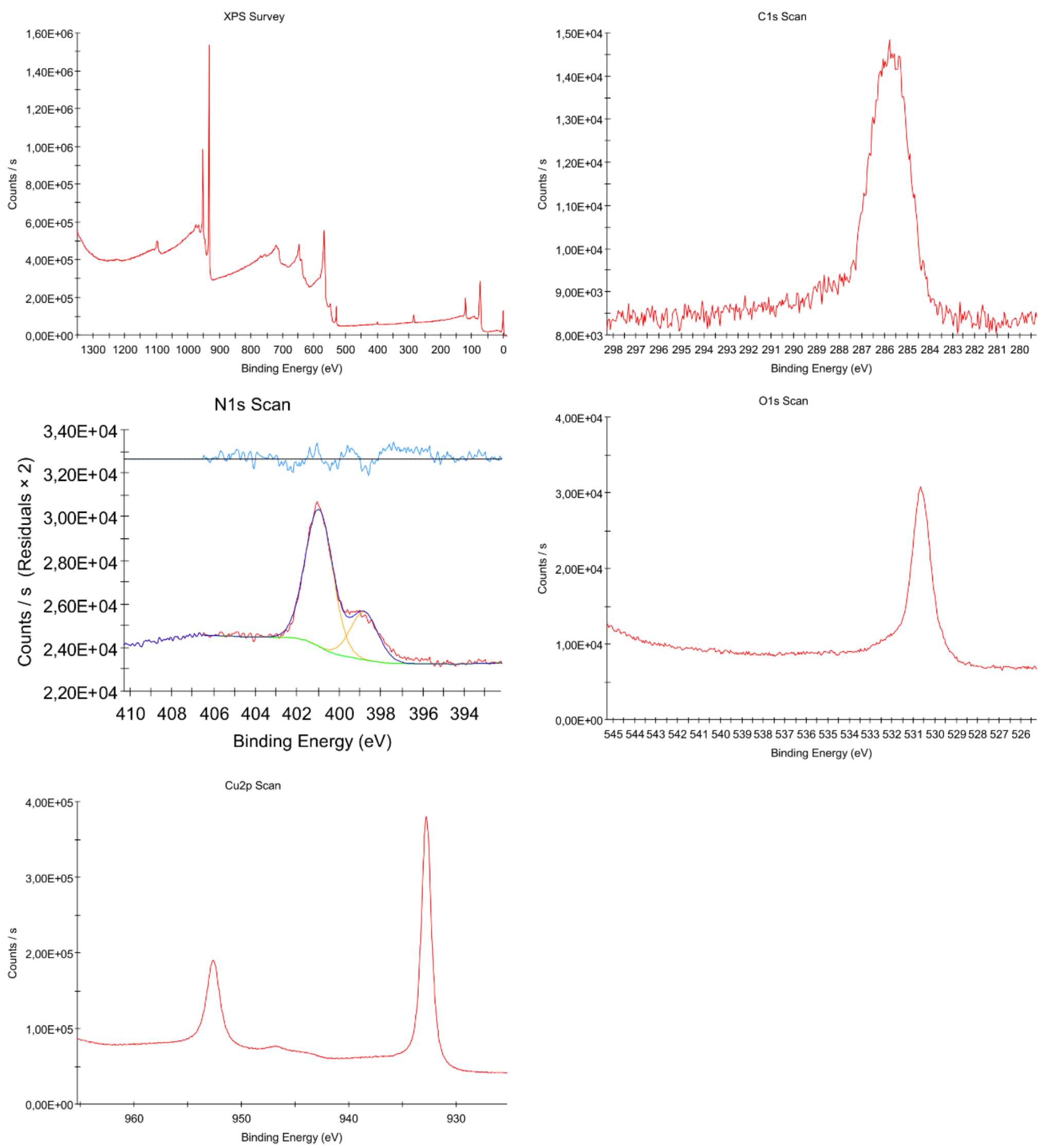

06.02.2026 14:19:59 O:\ANP\GLO_0321_ANP-AB-186_continuation\ANP-AB-186_03.vgp

Figure S22: Survey and element spectra accompanying the N 1s XPS spectrum displayed in Figure 2 b. Sample preparation details: PVD modification of Cu slide with $BIMeH\cdot HCO_3$ at 100 °C for 15 minutes. Washed with $EtOH_{abs}$ and acetone.

Peaks					
**Name**	**Peak BE**	**FWHM eV**	**Area (P) CPS.eV**	**Atomic %**	**Q**
N1s	400.46	2.09	7572.10	71.79	Yes
N1s Scan A	398.54	2.09	2979.28	28.21	Yes

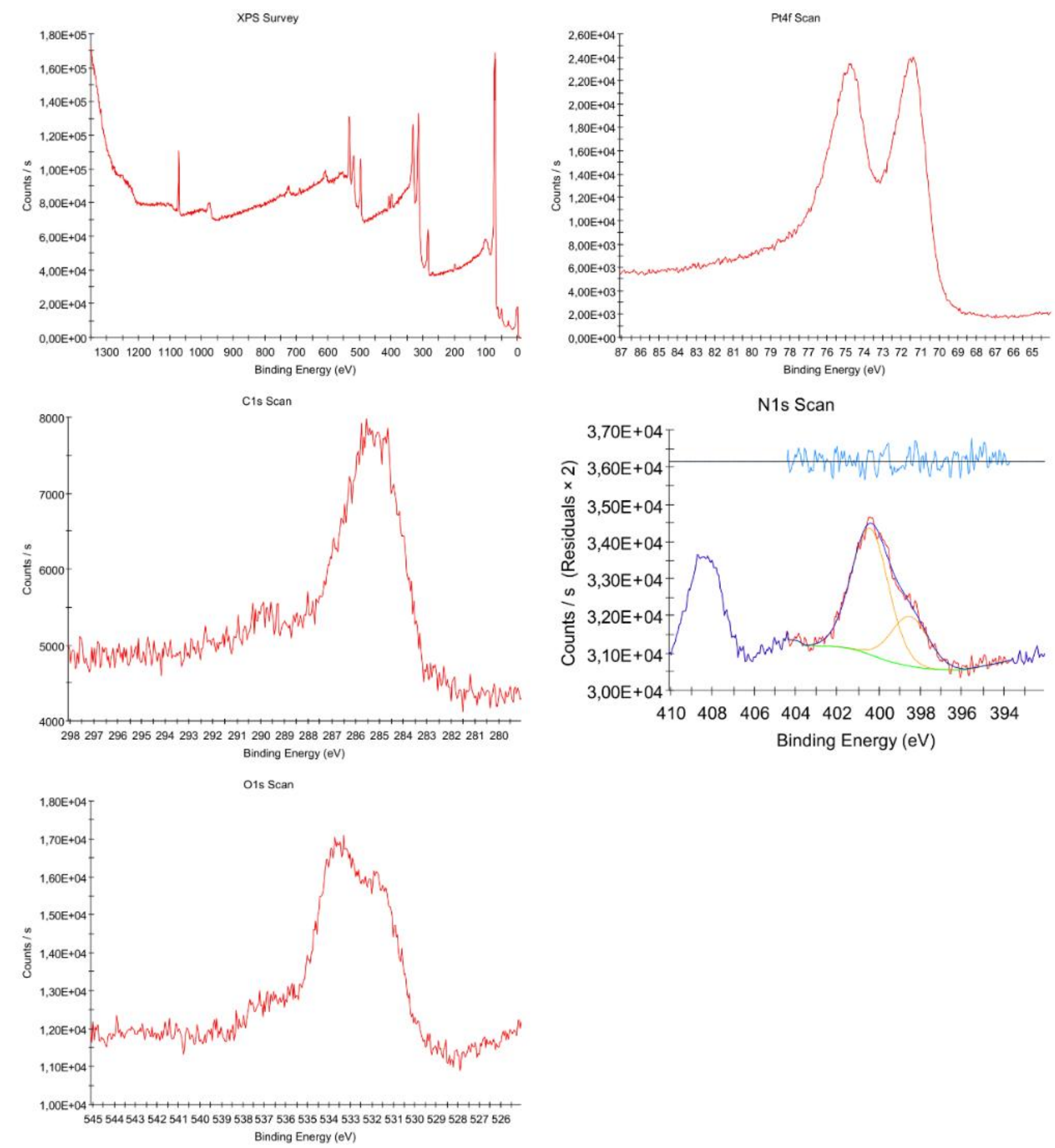

09.02.2026 12:18:00 O:\ANP\GLO_1119_ANP-AB-143\ANP-AB-143_B.vgp

Figure S23: Survey and element spectra accompanying the N 1s XPS spectrum displayed in Figure 2 c. Sample preparation details: Bottom-up nanoparticle synthesis using 20 mg of PtO_2 (1 eq, 0.088 mmol) and 6.5 mg (0.35 eq, 0.031 mmol) of BIMeH·HCO_3 in 3 ml THF, which was stirred at 750 rpm at rt. and 20 bar of H_2. After three days, the NPs were washed 4 times with THF and sonication and dried under reduced pressure.[11] Assumingly, the reduction with H_2 was not completed in the given time leading to oxygen content in the NPs and to an oxidized N species in the N 1s scan at 409 eV.

Peaks					
Name	Peak BE	FWHM eV	Area (P) CPS.eV	Atomic %	Q
N1s	400.34	2.42	15380.82	56.83	Yes
N1s Scan A	398.40	2.42	9175.98	33.86	Yes
N1s Scan B	403.14	2.42	2515.24	9.31	Yes

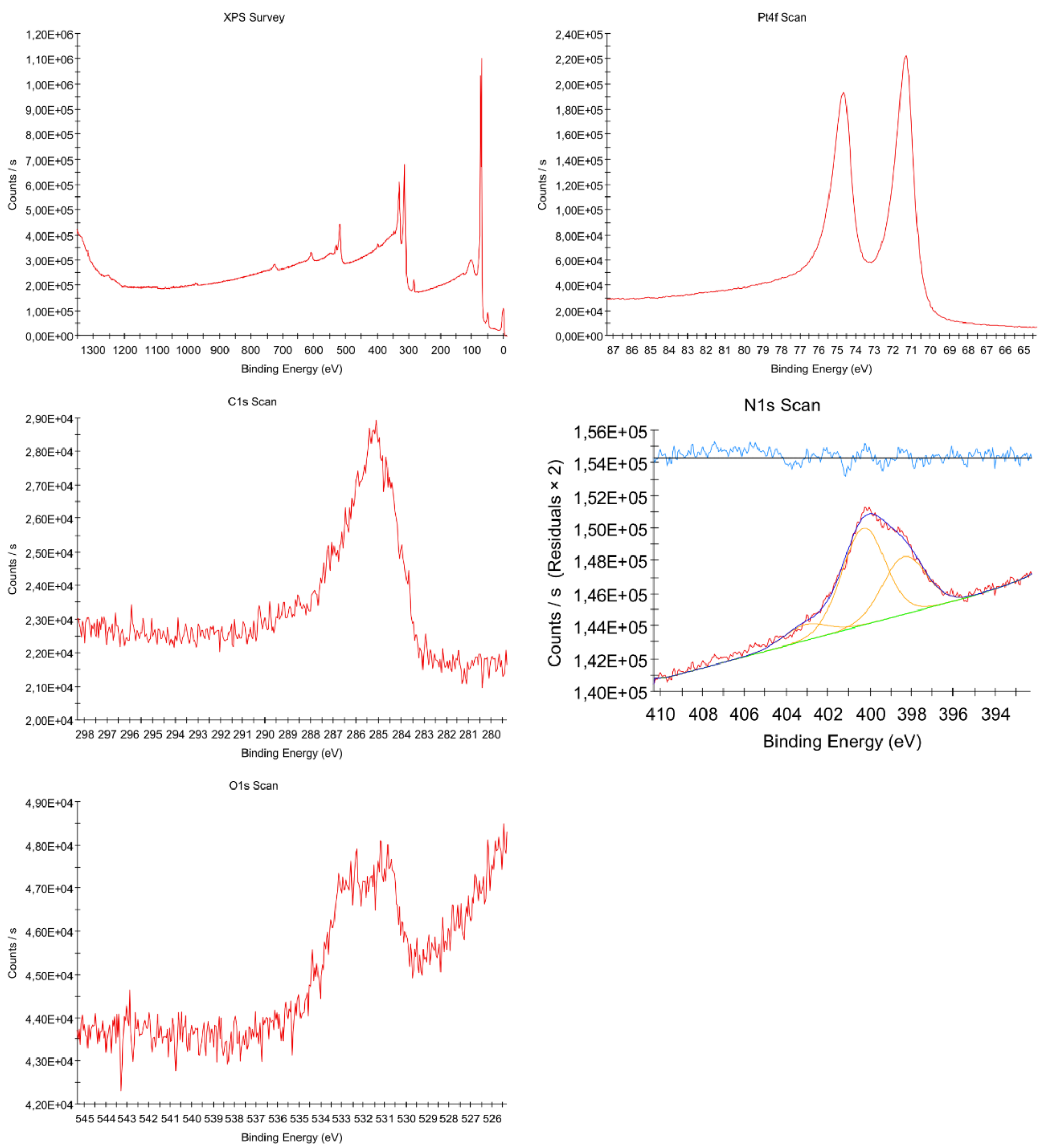

06.02.2026 14:24:18 O:\ANP\GLO_0321_ANP-AB-186_continuation\HSS-HB-554.vgp

Figure S24: Survey and element spectra accompanying the N 1s XPS spectrum displayed in Figure 2 d. Sample preparation details: PVD modification of Pt slide with BIMeH·HCO_3 at 100 °C for 15 minutes. Washed with $EtOH_{abs}$ and acetone.

Peaks					
Name	Peak BE	FWHM eV	Area (P) CPS.eV	Atomic %	Q
N1s	399.02	2.41	3006.64	58.56	Yes
N1s Scan A	400.82	2.41	2125.22	41.44	Yes

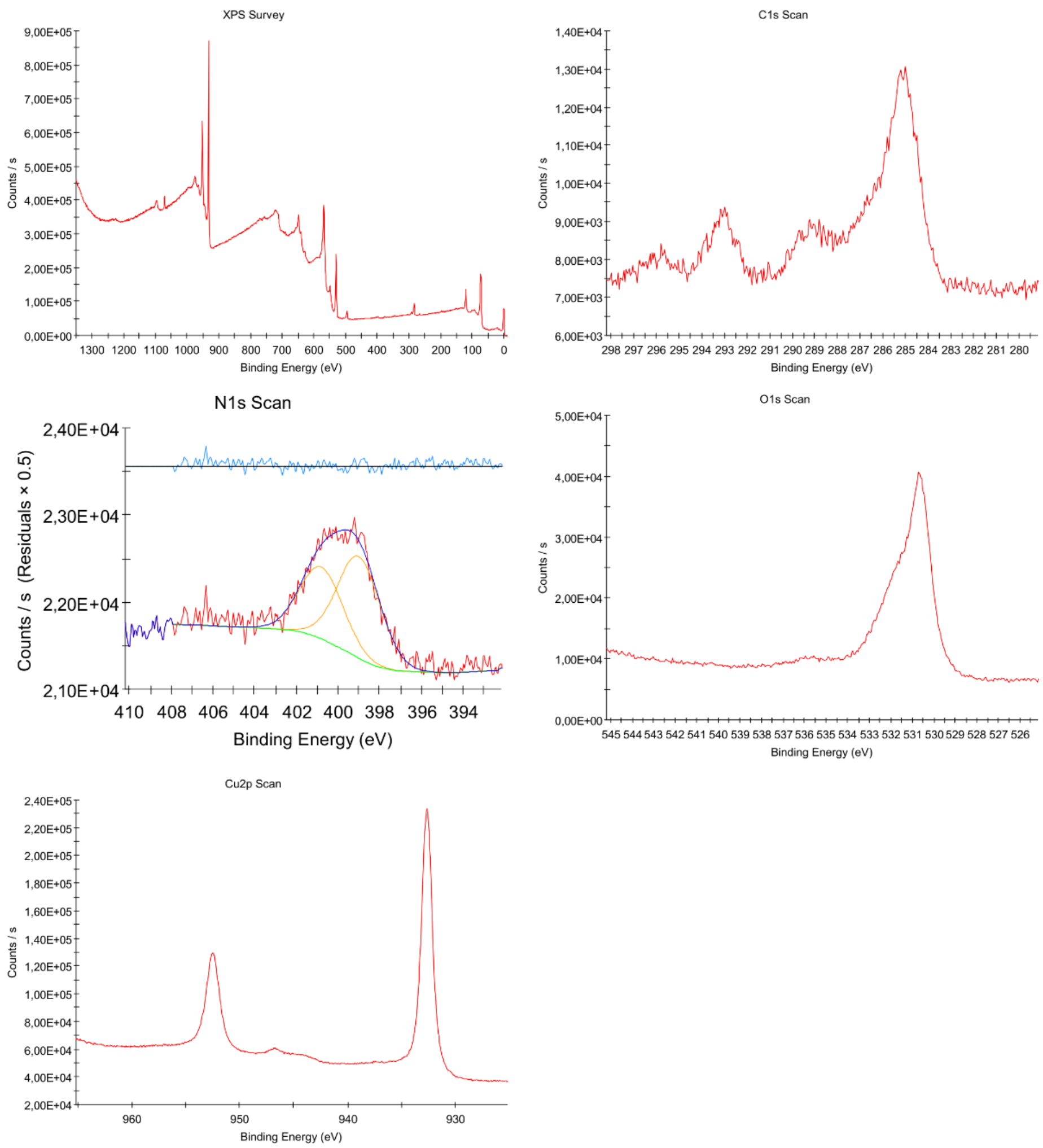

09.02.2026 12:19:15 O:\ANP\GLO_0730_ANP-AB-106\ANP-AB-106_2 IPr Cu.vgp

Figure S25: Survey and element spectra accompanying the N 1s XPS spectrum displayed in Figure 2 e. Sample preparation details: Cu slide and 5 mg IPrH·HCO_3 in 6 ml CH_2Cl_2 for 5 days. Washed with CH_2Cl_2, MeOH and again CH_2Cl_2.

Peaks					
**Name**	**Peak BE**	**FWHM eV**	**Area (P) CPS.eV**	**Atomic %**	**Q**
N1s	401.11	2.23	3874.63	59.44	Yes
N1s Scan A	398.89	2.23	2647.89	40.56	Yes

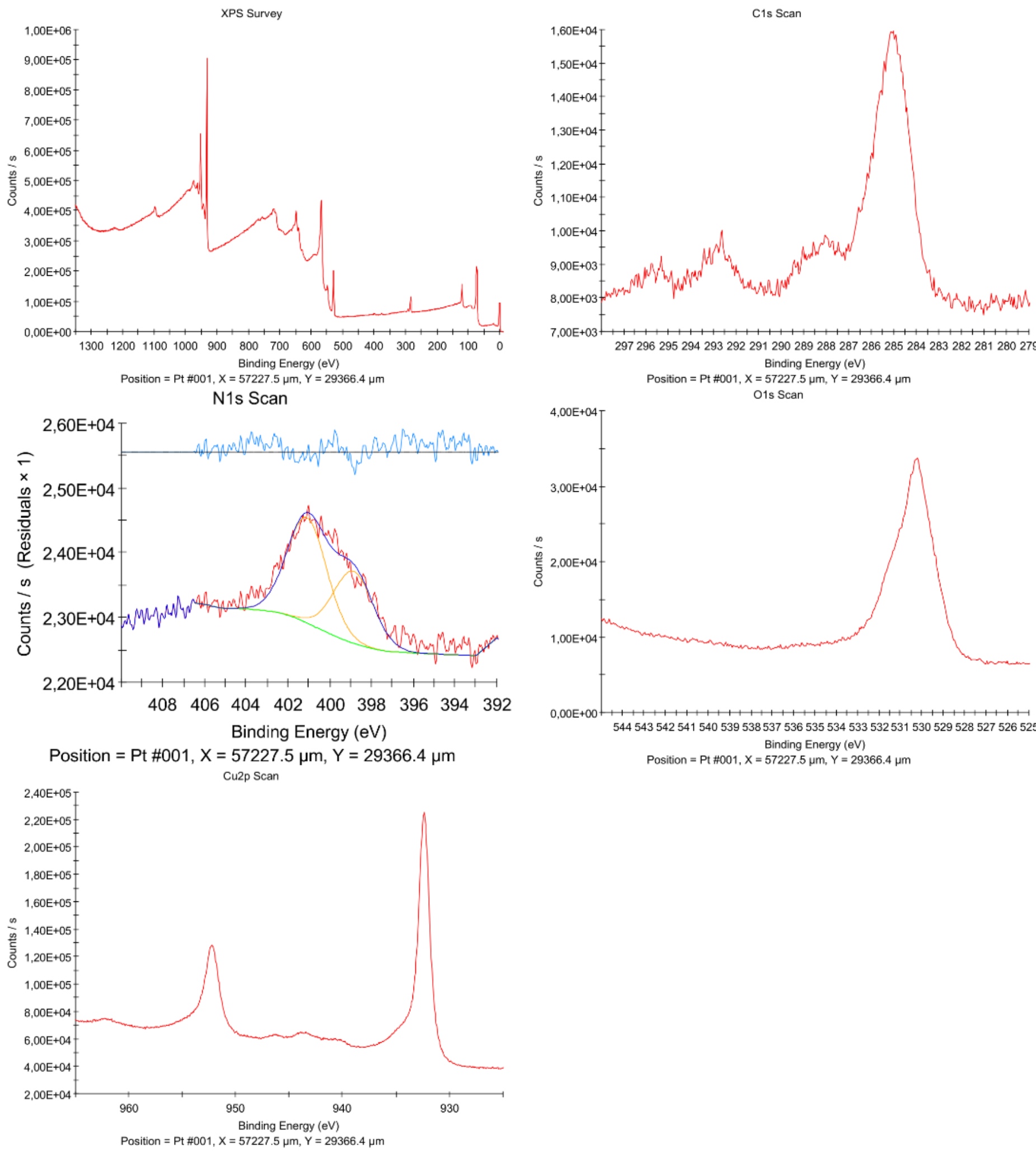

Figure S26: Survey and element spectra accompanying the N 1s XPS spectrum displayed in Figure 2 f. Sample preparation details: Cu slide and 8 mg $IPrCO_2$ in 1 ml MeOH at 60 °C for 90 minutes. Washed with iPrOH.

Peaks					
Name	Peak BE	FWHM eV	Area (P) CPS.eV	Atomic %	Q
N1s	398.92	1.79	4381.95	77.14	Yes
N1s Scan A	400.63	1.79	1297.14	22.86	Yes

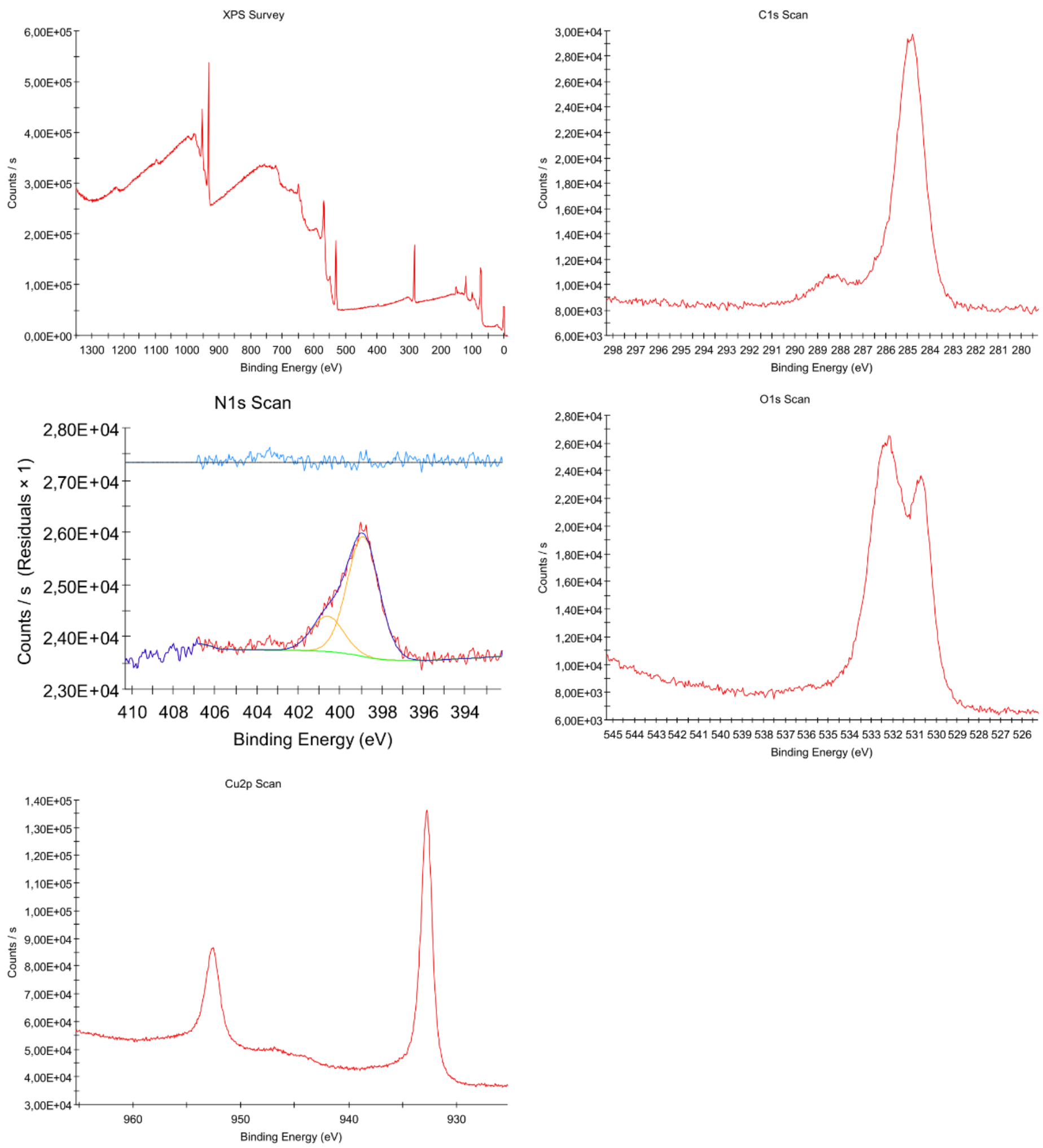

09.02.2026 12:26:24 O:\ANP\GLO_0416_ANP-AC-003_rewashed (1004_JGE-JB-109)\ANP-AC-003_rewashed_4.vgp

Figure S27: Survey and element spectra accompanying the N 1s XPS spectrum displayed in Figure 2 g. Sample preparation details: PVD modification of Cu slide with $IPrCO_2$ at 140 °C for 30 minutes. Washed with iPrOH.

Peaks					
Name	Peak BE	FWHM eV	Area (P) CPS.eV	Atomic %	Q
N1s	398.46	1.94	11843.35	45.84	Yes
N1s Scan A	400.25	1.94	10176.05	39.44	Yes
N1s Scan B	402.40	1.94	3794.12	14.73	Yes

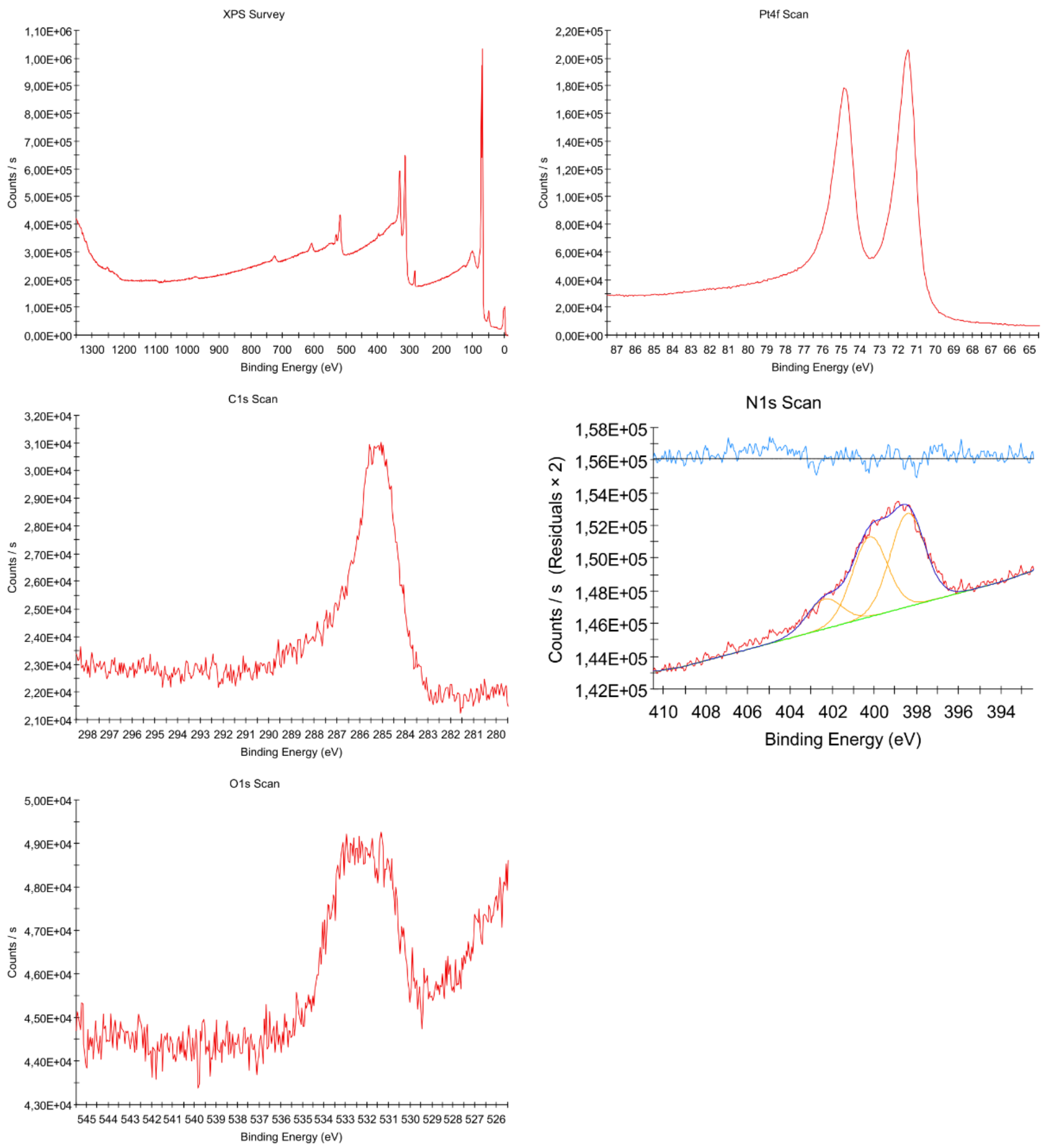

09.02.2026 12:25:27 O:\ANP\GLO_0416_ANP-AC-003_rewashed (1004_JGE-JB-109)\ANP-AC-003_rewashed_3.vgp

Figure S28: Survey and element spectra accompanying the N 1s XPS spectrum displayed in Figure 2 h. Sample preparation details: PVD modification of Pt slide with IPrCO$_2$ at 140 °C for 30 minutes. Washed with iPrOH.

Peaks					
Name	Peak BE	FWHM eV	Area (P) CPS.eV	Atomic %	Q
N1s	398.96	1.65	9505.95	87.13	Yes
N1s Scan A	400.80	1.65	1402.40	12.87	Yes

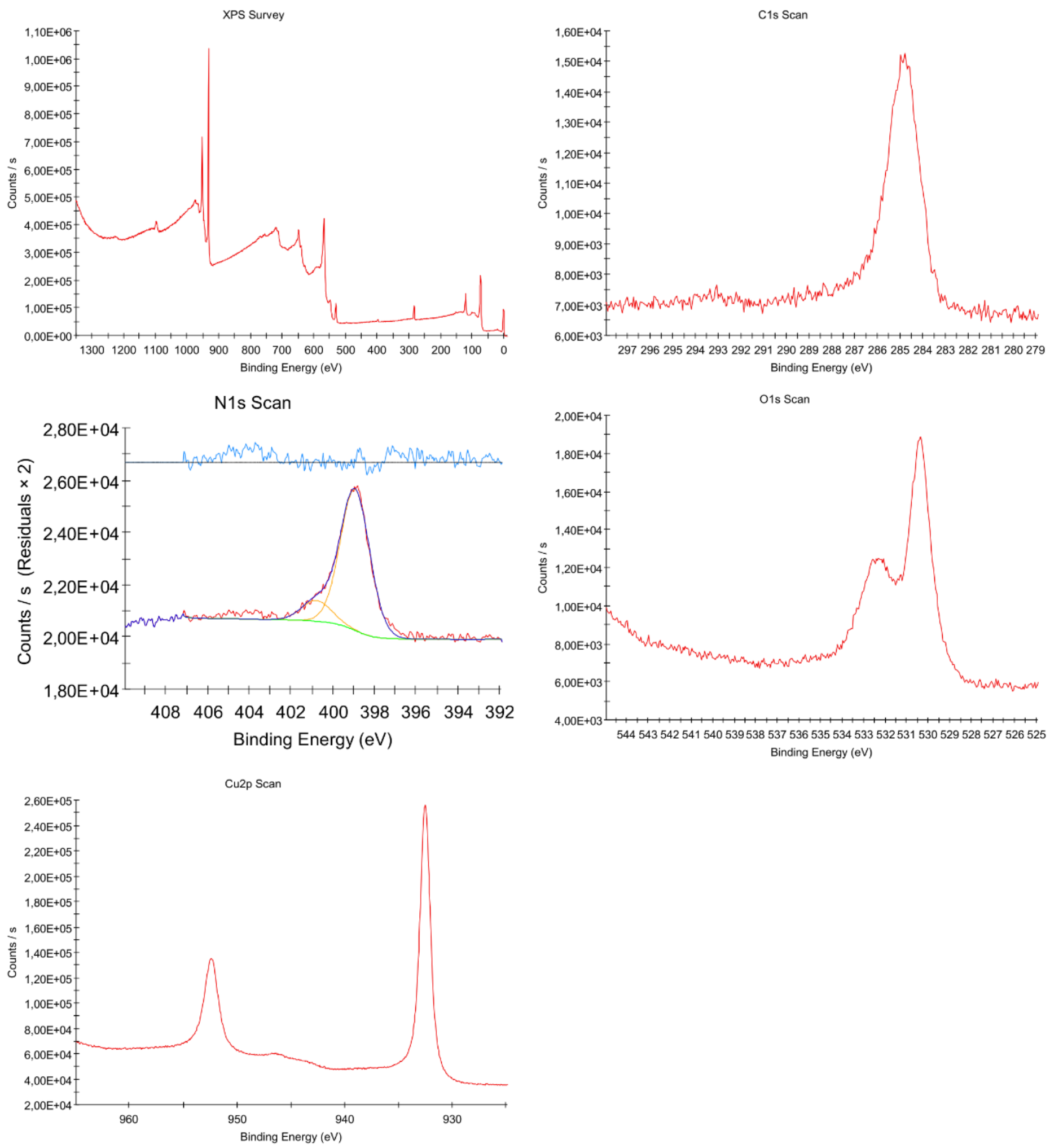

09.02.2026 12:45:36 O:\ANP\GLO_0128_ANP-AB-167\ANP-AB-167 13.vgp

Figure S29: Survey and element spectra accompanying the N 1s XPS spectrum displayed in Figure 2 i. Sample preparation details: Cu slide and 5 mg $BIiPrH\cdot HCO_3$ in 100 µl MeOH and 2 ml CH_2Cl_2 for 19 hours. Washed with CH_2Cl_2, MeOH and again CH_2Cl_2.

Peaks					
Name	Peak BE	FWHM eV	Area (P) CPS.eV	Atomic %	Q
N1s	398.73	2.04	14424.81	70.08	Yes
N1s Scan A	400.62	2.04	4758.01	23.15	Yes
N1s Scan B	402.53	2.04	1389.54	6.77	Yes

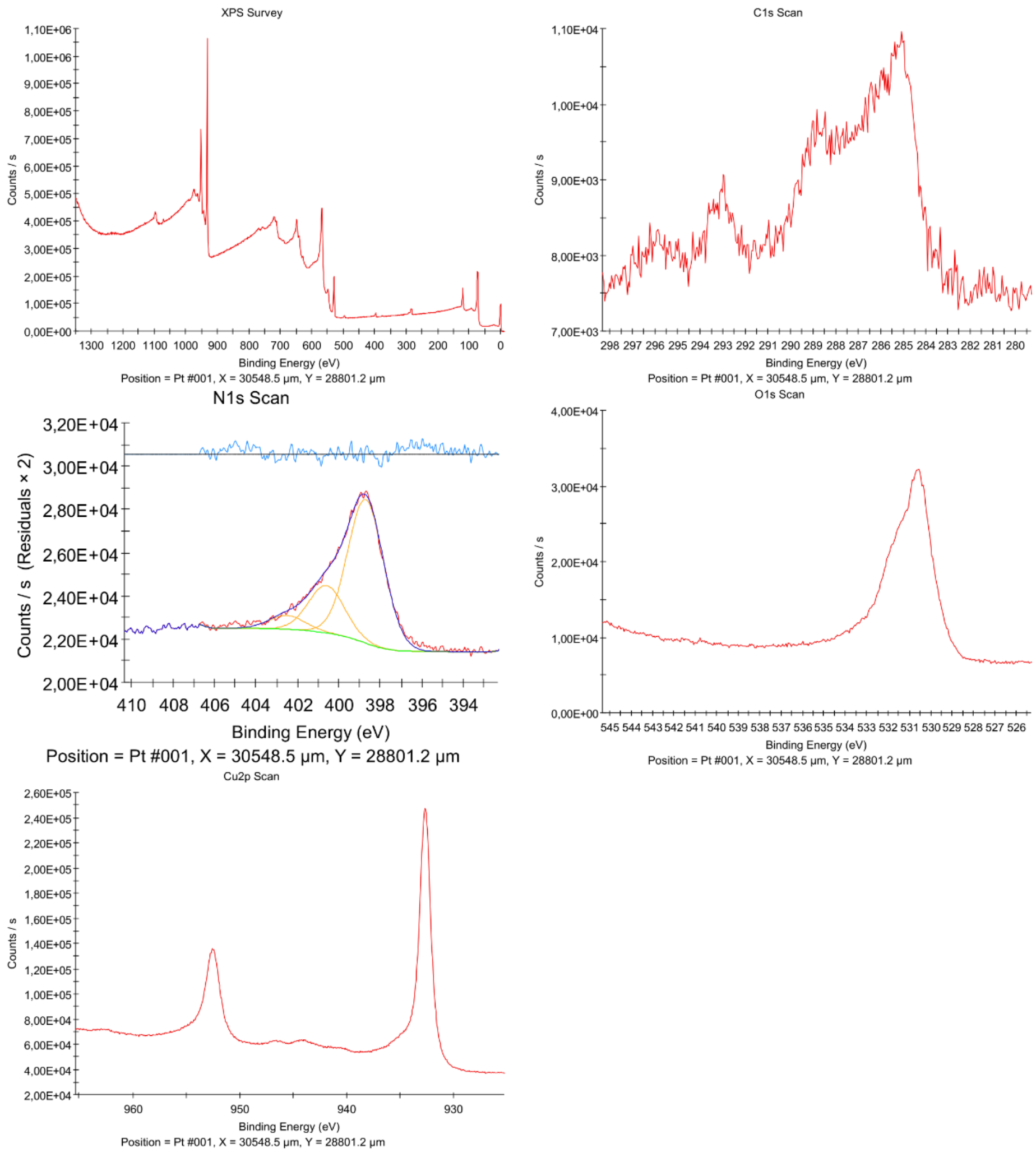

09.02.2026 12:31:10 O:\ANP\GLO_1104_ANP-AC-048\ANP-AC-048_4 IMeCO2 MeOH.vgp

Figure S30: Survey and element spectra accompanying the N 1s XPS spectrum displayed in Figure 2 j. Sample preparation details: Cu slide and 8 mg IMeCO$_2$ in 1 ml MeOH at 60 °C for 90 minutes. Washed with iPrOH.

Peaks					
Name	Peak BE	FWHM eV	Area (P) CPS.eV	Atomic %	Q
N1s	400.11	1.74	10555.52	66.70	Yes
N1s Scan A	398.83	1.74	5273.93	33.30	Yes

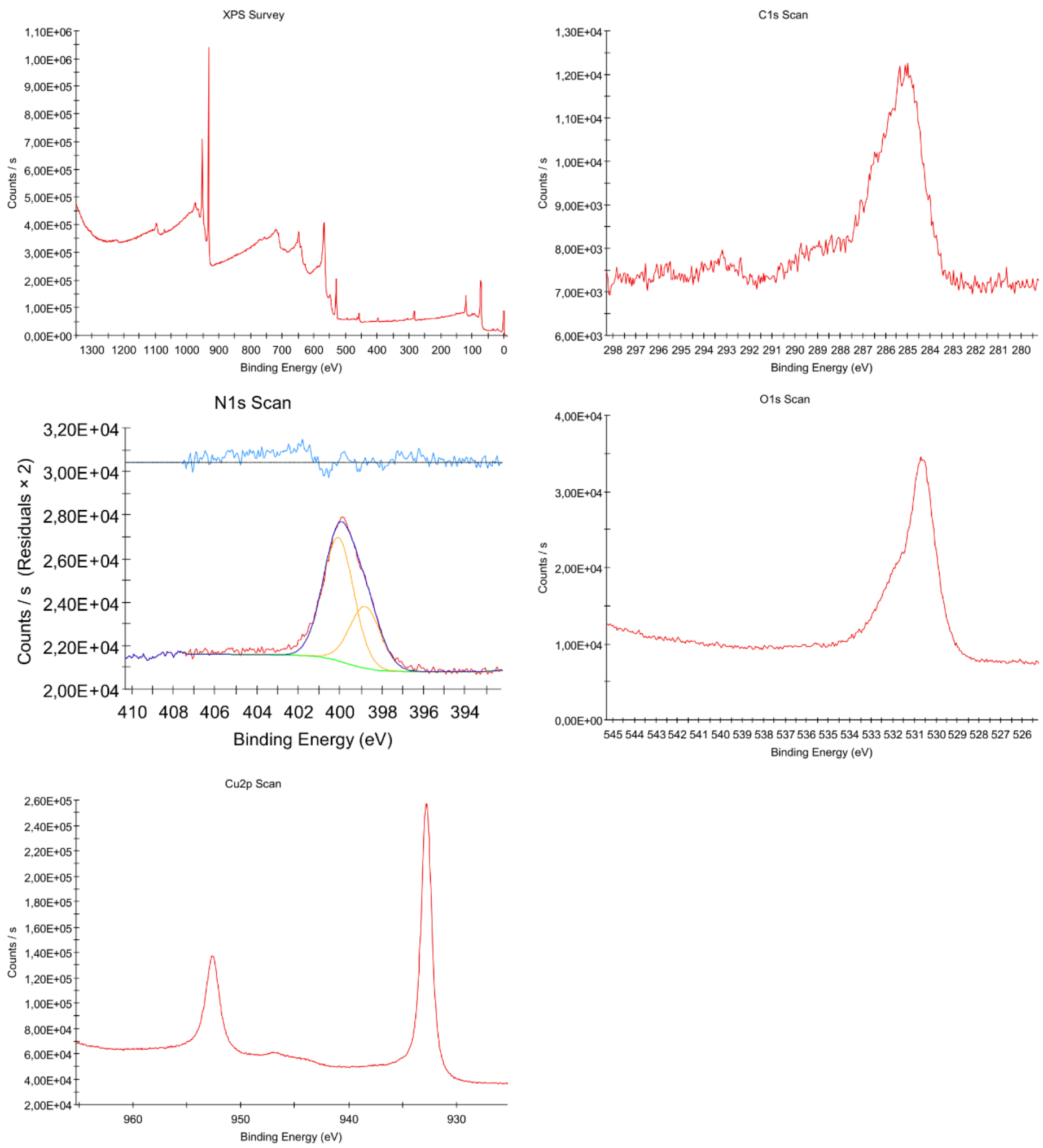

09.02.2026 12:46:32 O:\ANP\GLO_0412_ANP-AB-065\ANP-AB-065_3.vgp

Figure S31: Survey and element spectra accompanying the N 1s XPS spectrum displayed in Figure 2 k. Sample preparation details: Cu slide and 7 mg 2-(2,6-dimethoxyphenyl)-6,7-dihydro-5H-pyrrolo[2,1-c][1,2,4]triazol-2-ium chloride and 11 mg K_2CO_3 in 3 ml CH_2Cl_2 for 15 hours. Washed with H_2O, MeOH and again CH_2Cl_2.

Peaks					
Name	Peak BE	FWHM eV	Area (P) CPS.eV	Atomic %	Q
N1s	400.69	1.85	9874.22	72.98	Yes
N1s Scan A	398.83	1.85	3660.57	27.02	Yes

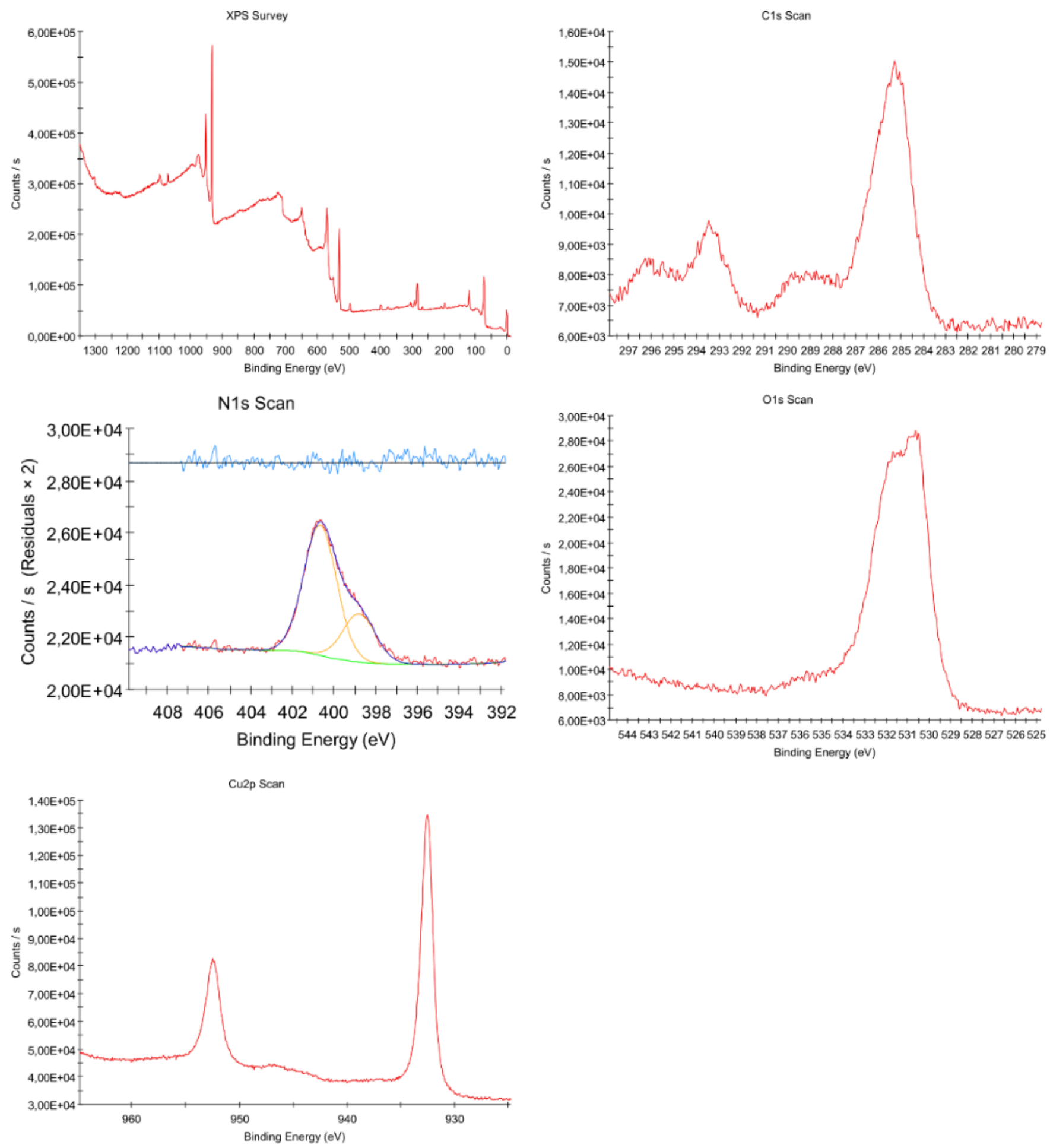

09.02.2026 12:48:02 O:\ANP\GLO_0730_ANP-AB-106\ANP-AB-109_negative repeat edge.vgp

Figure S32: Survey and element spectra accompanying the N 1s XPS spectrum displayed in Figure 2 l. Sample preparation details: Cu foil (100 µm thick) in 10 ml MeOH (20 mM of $LiClO_4$ and 1 mM BIMeH·HCO_3) under cyclic voltammetry conditions (0.1 V/s, +1 V / -1.2, 10 cycles, starting with positive semi-cycle and finishing with negative semi-cycle). Washed with H_2O, MeOH and CH_2Cl_2.

Peaks					
Name	Peak BE	FWHM eV	Area (P) CPS.eV	Atomic %	Q
N1s Scan A	400.51	2.42	2357.73	48.49	Yes
N1s Scan B	398.24	2.42	2508.26	51.51	Yes

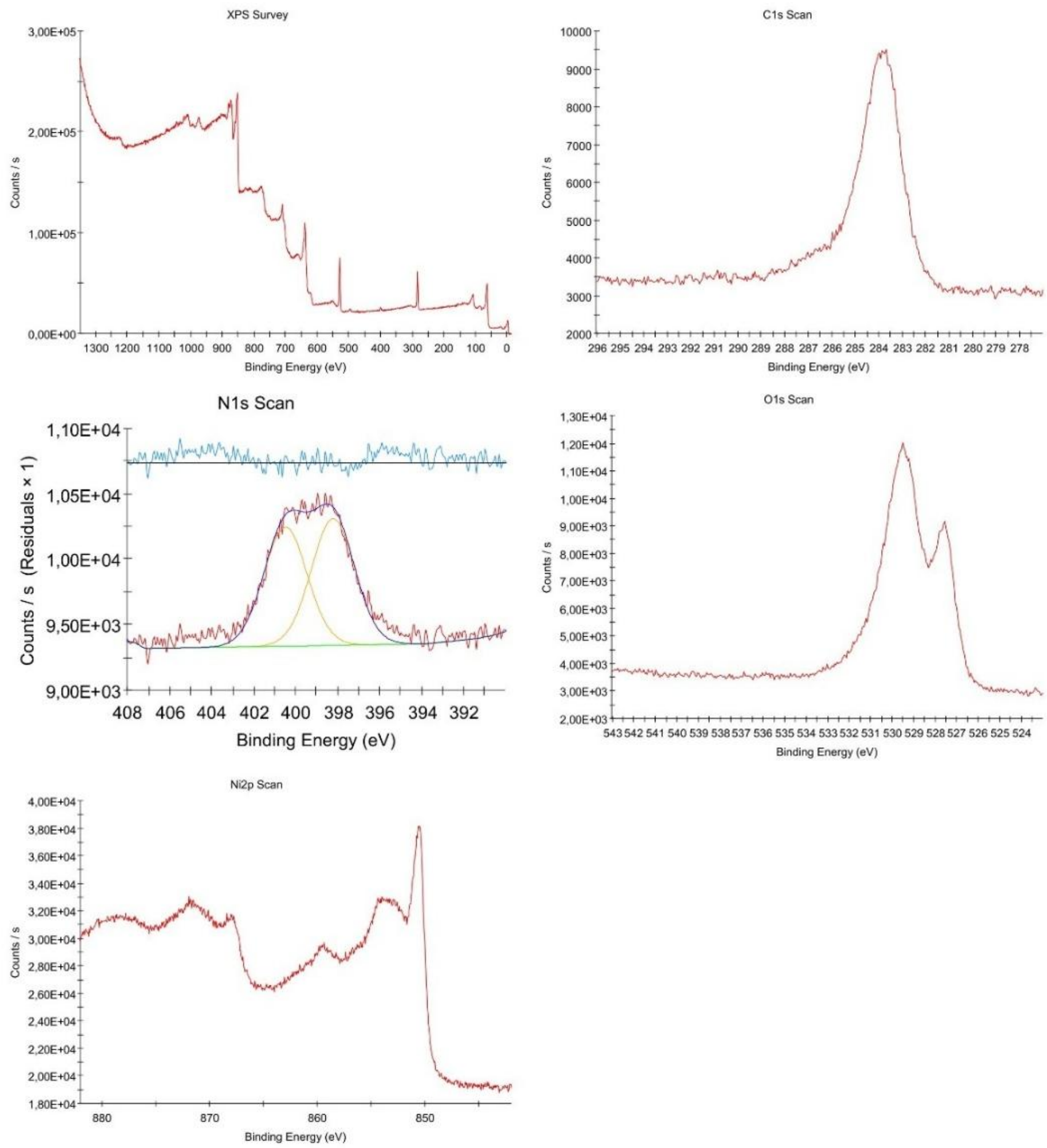

10.02.2026 14:38:33 L:\Gemeinsame Projekte - Common projects\ANP-HSS-LAS\Etching\Cu SI\HSS-HA-248.vgp

Figure S33: Survey and element XPS spectra of NHC modified Ni NPs. Sample preparation details: Bottom-up nanoparticle synthesis using 30 mg of Ni(COD)$_2$ (1 eq, 0.11 mmol) and 21.3 mg of IPrH·HCO$_3$ (0.5 eq, 0.055 mmol) in 1 ml THF. After stirring at 750 rpm (no magnetic stirring bar as the formed particles are magnetic) at 10 bar of H_2 pressure for 48 hours, volatiles were removed under reduced pressure. The residue was suspended in n-hexane. The NPs were collected over a magnet and washed again with n-hexane.[11]

4.5 Data associated with Figure 3

Peaks					
Name	Peak BE	FWHM eV	Area (P) CPS.eV	Atomic %	Q
N1s Scan A	400.57	2.03	14334.64	100.00	Yes

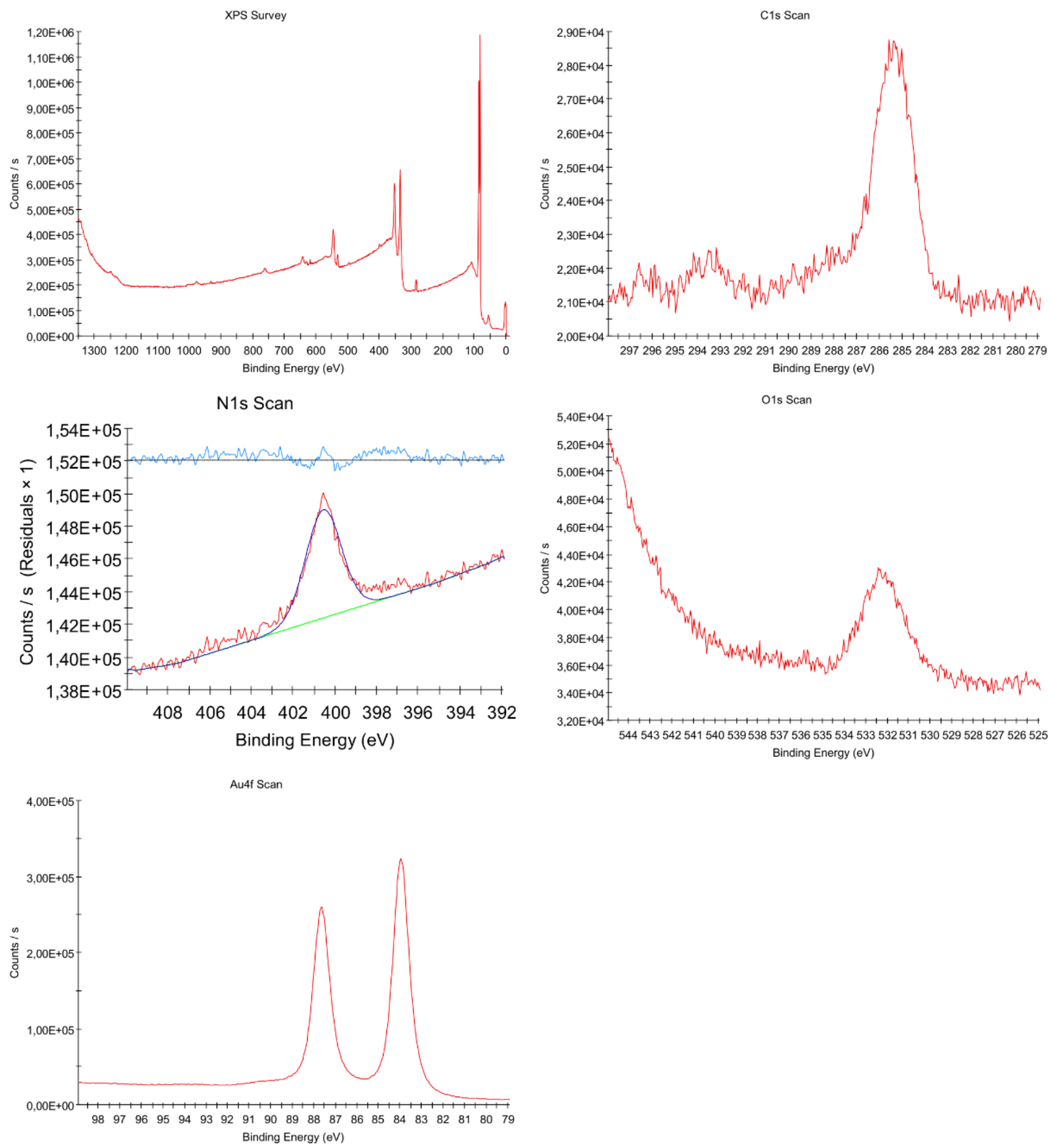

09.02.2026 12:20:03 O:\ANP\GLO_0730_ANP-AB-106\ANP-AB-106_5 BIMe Au.vgp

Figure S34: Survey and element spectra accompanying the N 1s XPS spectrum displayed in Figure 3 a. Sample preparation details: Au slide and 5 mg BIMeH·HCO_3 in 6 ml CH_2Cl_2 for 5 days. Washed with CH_2Cl_2, MeOH and again CH_2Cl_2.

Peaks					
Name	Peak BE	FWHM eV	Area (P) CPS.eV	Atomic %	Q
N1s	400.48	2.42	8906.76	100.00	Yes

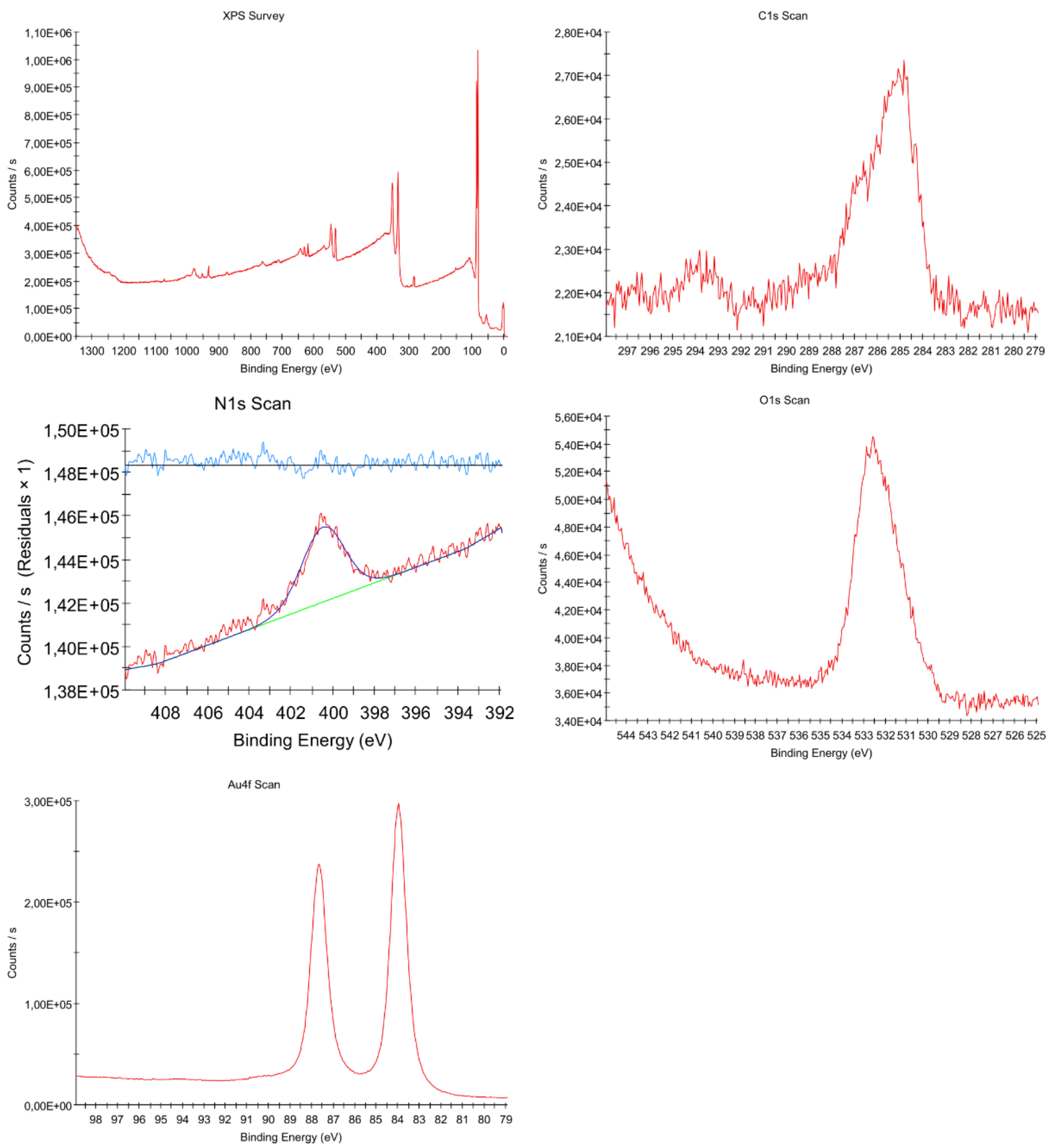

09.02.2026 12:21:22 O:\ANP\GLO_0807_ANP-AB-110\ANP-AB-110_5 BIMe Au again.vgp

Figure S35: Survey and element spectra accompanying the N 1s XPS spectrum displayed in Figure 3 b. Sample preparation details: Au slide and 5 mg BIMeH·HCO_3 in 6 ml MeOH for 5 days. Washed with CH_2Cl_2, MeOH and again CH_2Cl_2.

Peaks					
Name	Peak BE	FWHM eV	Area (P) CPS.eV	Atomic %	Q
N1s	400.74	1.74	12043.45	100.00	Yes

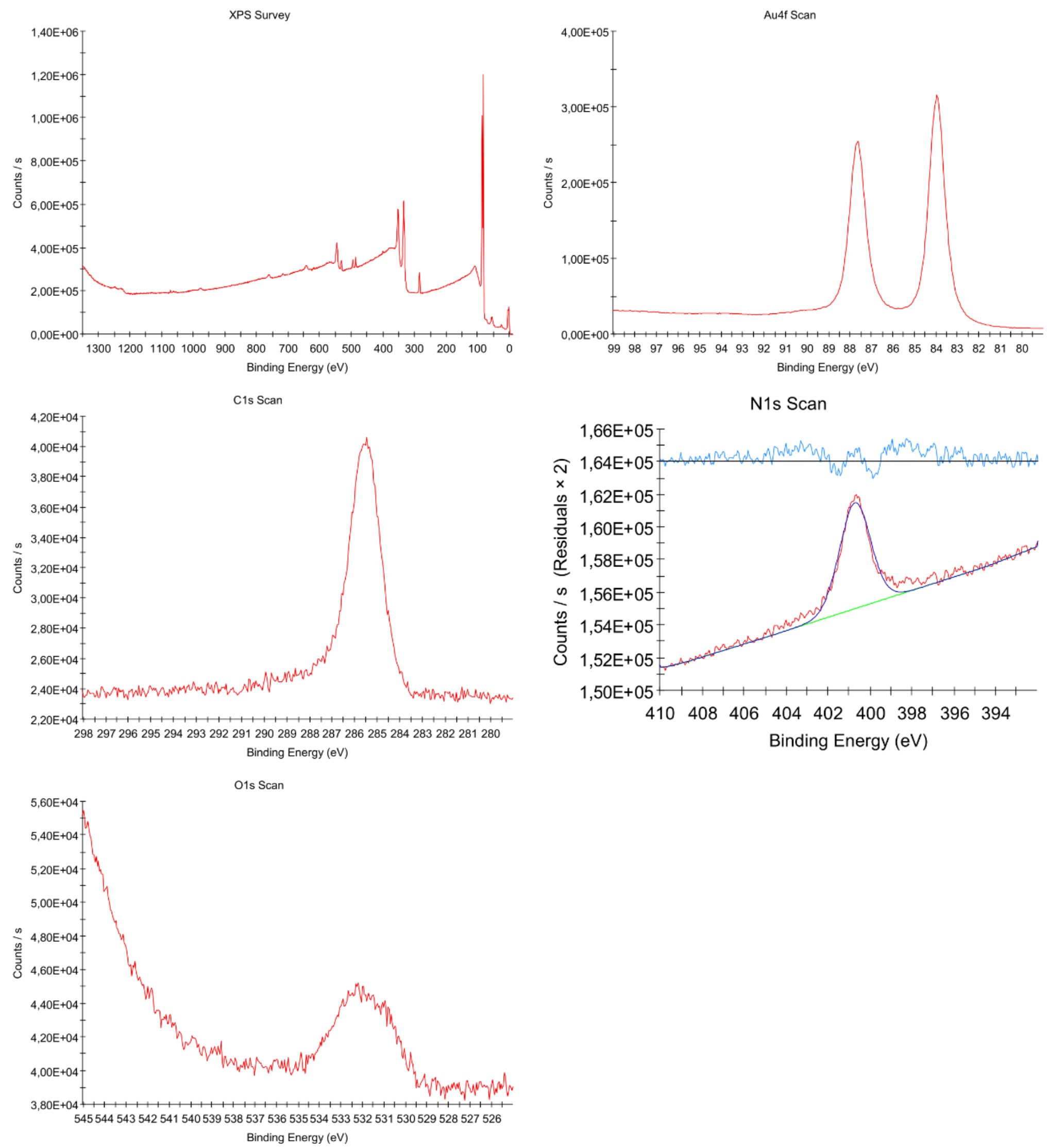

06.02.2026 14:25:05 O:\ANP\GLO_0321_ANP-AB-186_continuation\HSS-HB-553.vgp

Figure S36: Survey and element spectra accompanying the N 1s XPS spectrum displayed in Figure 3 c. Sample preparation details: PVD modification of Au slide with $BIMeH\cdot HCO_3$ at 100 °C for 15 minutes. Washed with $EtOH_{abs}$ and acetone.

Peaks					
Name	Peak BE	FWHM eV	Area (P) CPS.eV	Atomic %	Q
N1s	400.48	1.76	31812.22	100.00	Yes

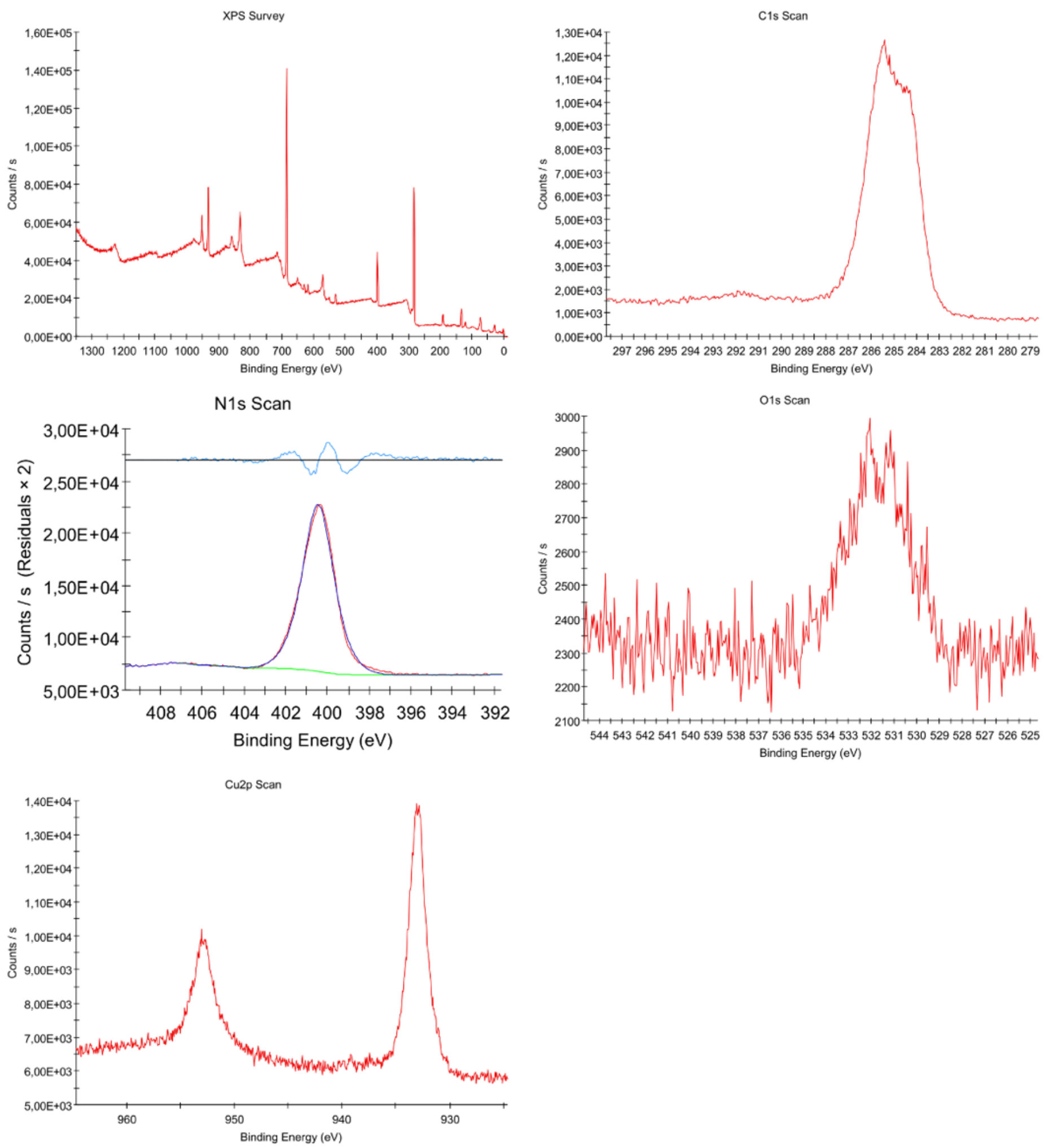

09.02.2026 12:23:20 O:\ANP\GLO_0703_ANP-AB103\ANP-AB-103-01.vgp

Figure S37: Survey and element spectra accompanying the N 1s XPS spectrum displayed in Figure 3 d. Sample preparation details: $[Cu(BIMe)_2]PF_6$ directly measured as powder XPS.

4.6 Data associated with Figure 4

Peaks					
Name	Peak BE	FWHM eV	Area (P) CPS.eV	Atomic %	Q
N1s	400.64	1.94	5052.23	54.49	Yes
N1s Scan A	398.48	1.94	4225.21	45.51	Yes

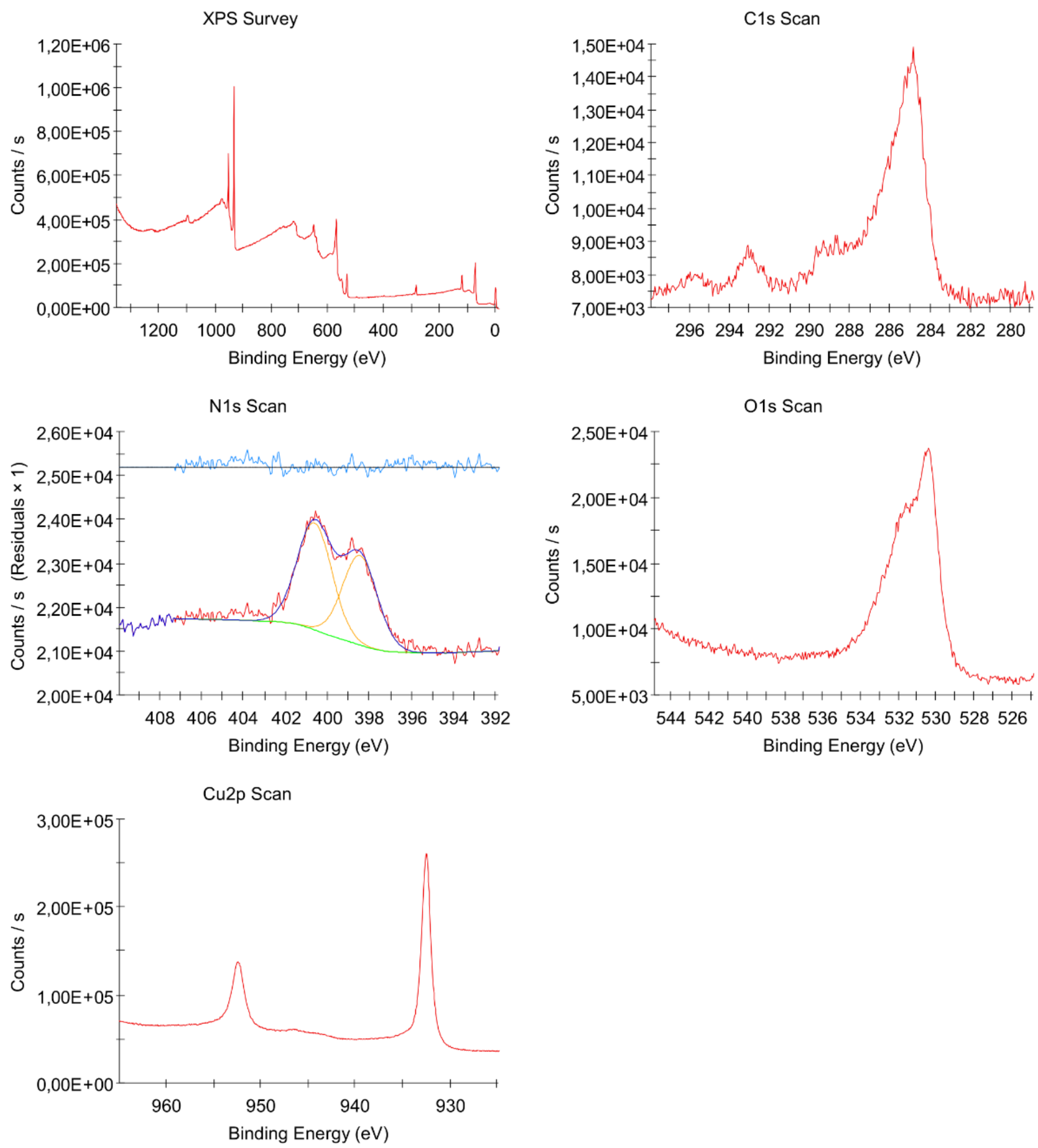

09.02.2026 11:06:51 O:\ANP\GLO_0123_ANP-AB-022\ANP-AB-022 ACN.vgp

Figure S38: Survey and element spectra accompanying the N 1s XPS spectrum displayed in Figure 4 a. Sample preparation details: Cu slide and 5 mg BIMeH·HCO_3 in 1 ml MeCN for 200 minutes. Washed with CH_2Cl_2, MeOH and again CH_2Cl_2.

Peaks					
Name	Peak BE	FWHM eV	Area (P) CPS.eV	Atomic %	Q
N1s	400.75	1.75	4092.49	61.78	Yes
N1s Scan A	398.68	1.75	2535.89	38.22	Yes

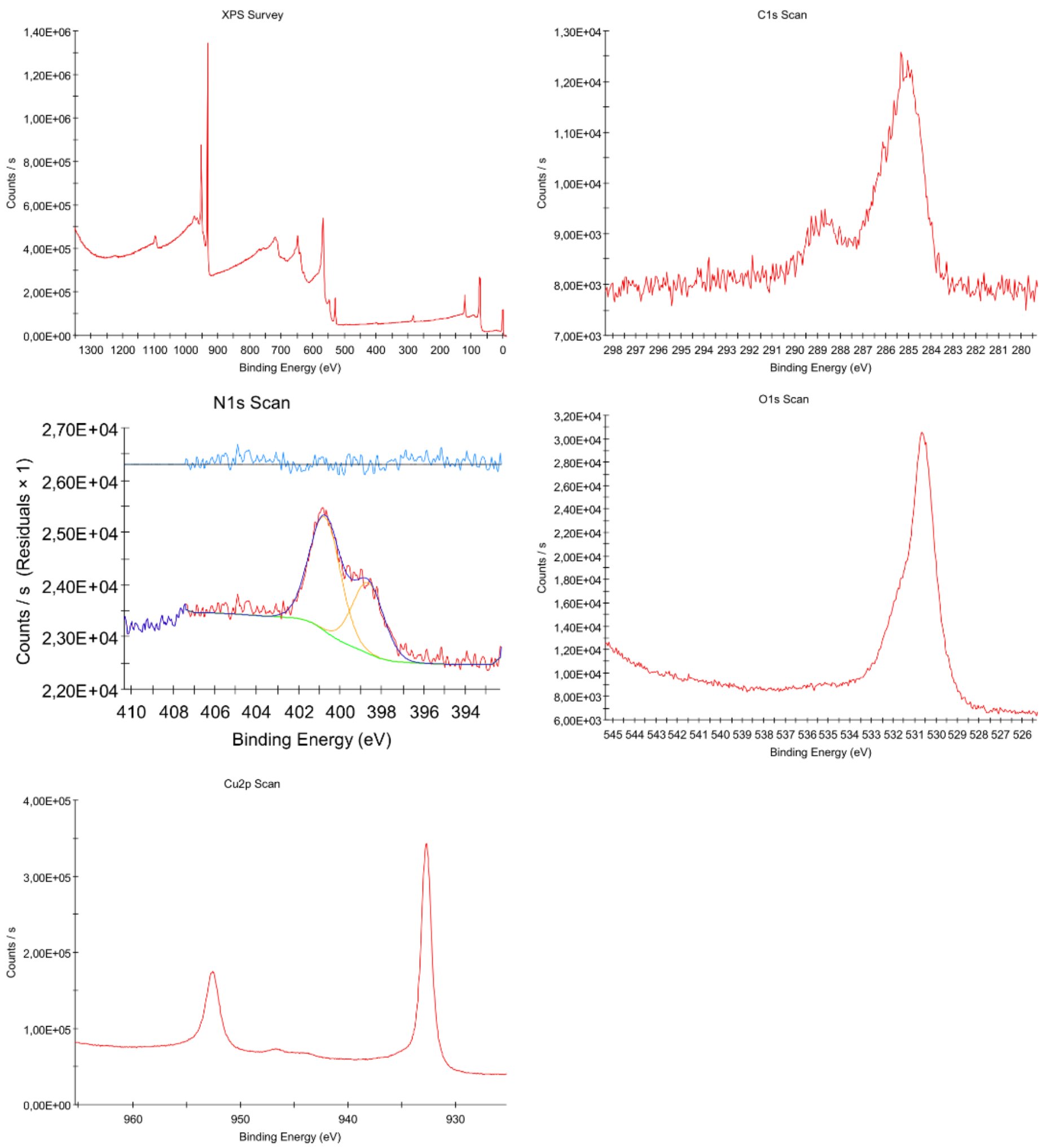

09.02.2026 13:32:50 O:\ANP\GLO_1016_HSS-HB-659 and ANP-AC-045\ANP-AC-045_3.vgp

Figure S39: Survey and element spectra accompanying the N 1s XPS spectrum displayed in Figure 4 b. Sample preparation details: Cu slide and 5 mg BIMeH·HCO_3 in 1 ml DCE for 200 minutes. Washed with CH_2Cl_2, MeOH and again CH_2Cl_2.

Peaks					
Name	Peak BE	FWHM eV	Area (P) CPS.eV	Atomic %	Q
N1s	400.78	1.73	4790.74	73.25	Yes
N1s Scan A	398.71	1.73	1751.89	26.75	Yes

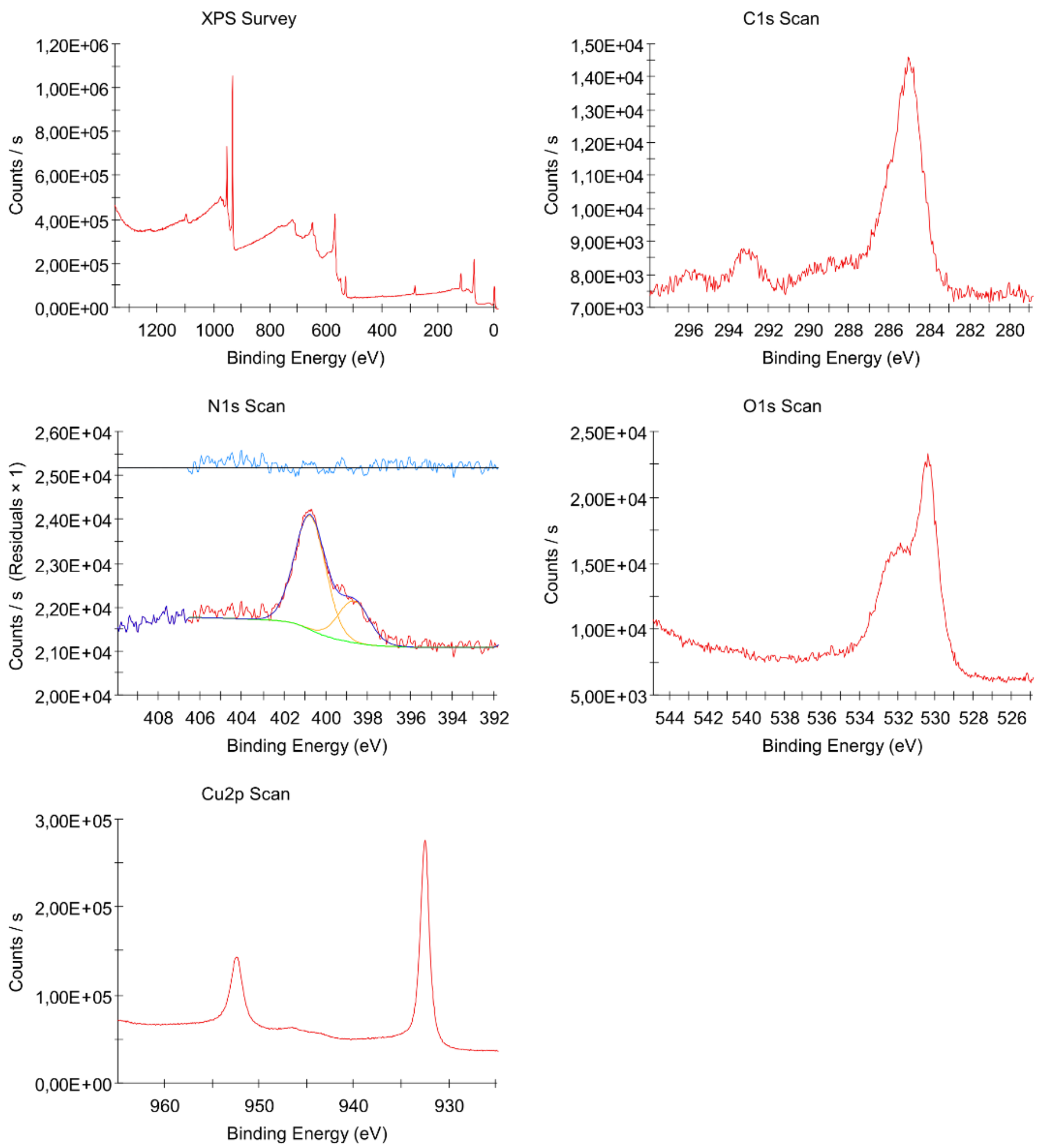

09.02.2026 11:11:00 O:\ANP\GLO_0123_ANP-AB-022\ANP-AB-022 DCM.vgp

Figure S40: Survey and element spectra accompanying the N 1s XPS spectrum displayed in Figure 4 c. Sample preparation details: Cu slide and 5 mg BIMeH·HCO_3 in 1 ml CH_2Cl_2 for 200 minutes. Washed with CH_2Cl_2, MeOH and again CH_2Cl_2.

Peaks					
Name	Peak BE	FWHM eV	Area (P) CPS.eV	Atomic %	Q
N1s	400.98	1.94	2850.44	60.93	Yes
N1s Scan A	402.93	1.94	942.76	20.18	Yes
N1s Scan B	399.16	1.94	884.97	18.89	Yes

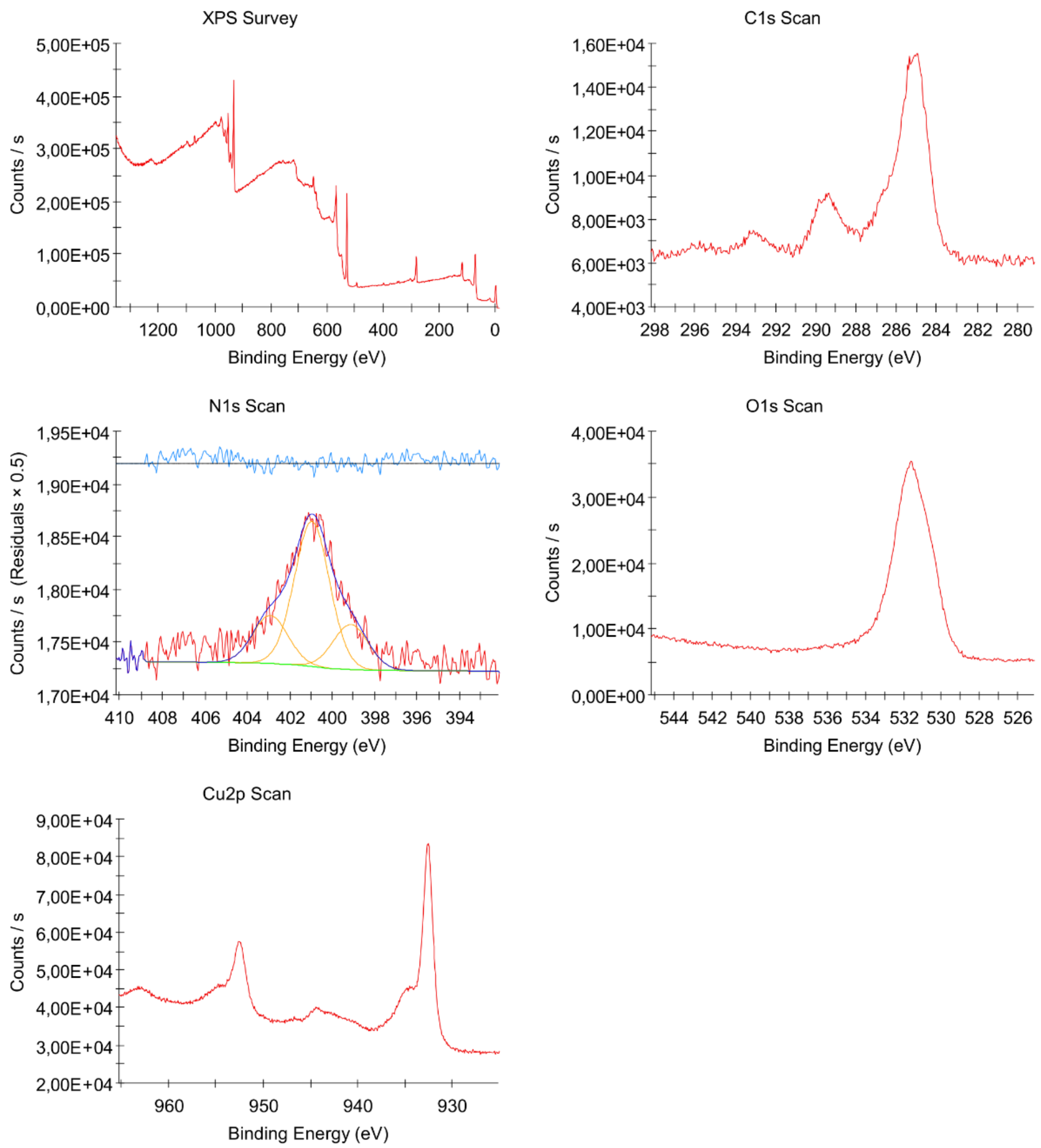

09.02.2026 11:23:17 O:\ANP\GLO_0123_ANP-AB-022\ANP-AB-022 MeOH.vgp

Figure S41: Survey and element spectra accompanying the N 1s XPS spectrum displayed in Figure 4 d. Sample preparation details: Cu slide and 5 mg BIMeH·HCO_3 in 1 ml MeOH for 200 minutes. Washed with CH_2Cl_2, MeOH and again CH_2Cl_2.

Peaks					
Name	Peak BE	FWHM eV	Area (P) CPS.eV	Atomic %	Q
N1s	401.12	1.94	1046.98	59.66	Yes
N1s Scan A	398.89	1.94	709.08	40.34	Yes

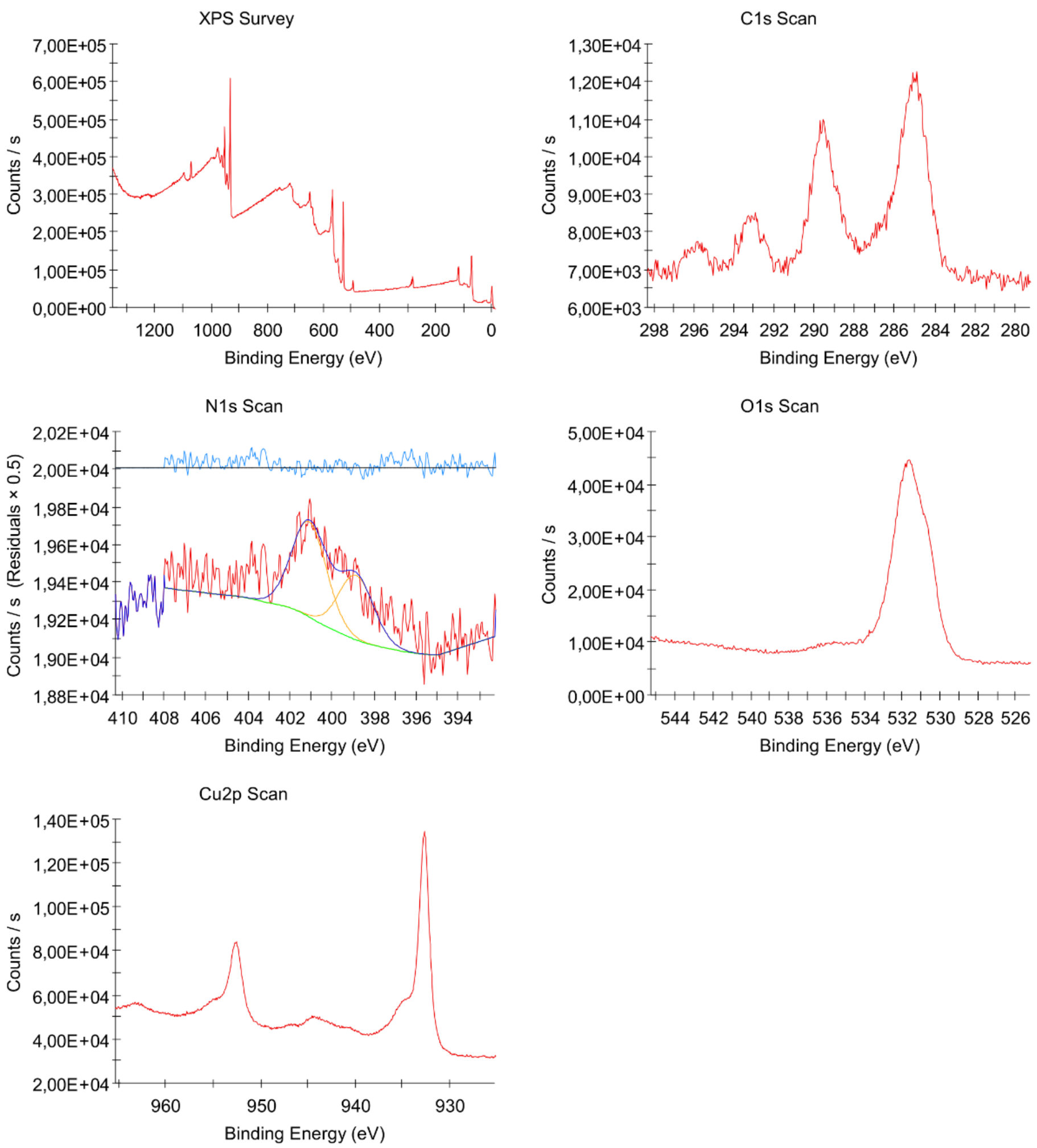

09.02.2026 11:17:01 O:\ANP\GLO_0123_ANP-AB-022\ANP-AB-022 THF.vgp

Figure S42: Survey and element spectra accompanying the N 1s XPS spectrum displayed in Figure 4 e. Sample preparation details: Cu slide and 5 mg BIMeH·HCO_3 in 1 ml THF for 200 minutes. Washed with CH_2Cl_2, MeOH and again CH_2Cl_2.

Peaks					
Name	Peak BE	FWHM eV	Area (P) CPS.eV	Atomic %	Q
N1s	398.86	1.94	946.21	55.29	Yes
N1s Scan A	400.53	1.94	764.10	44.71	Yes

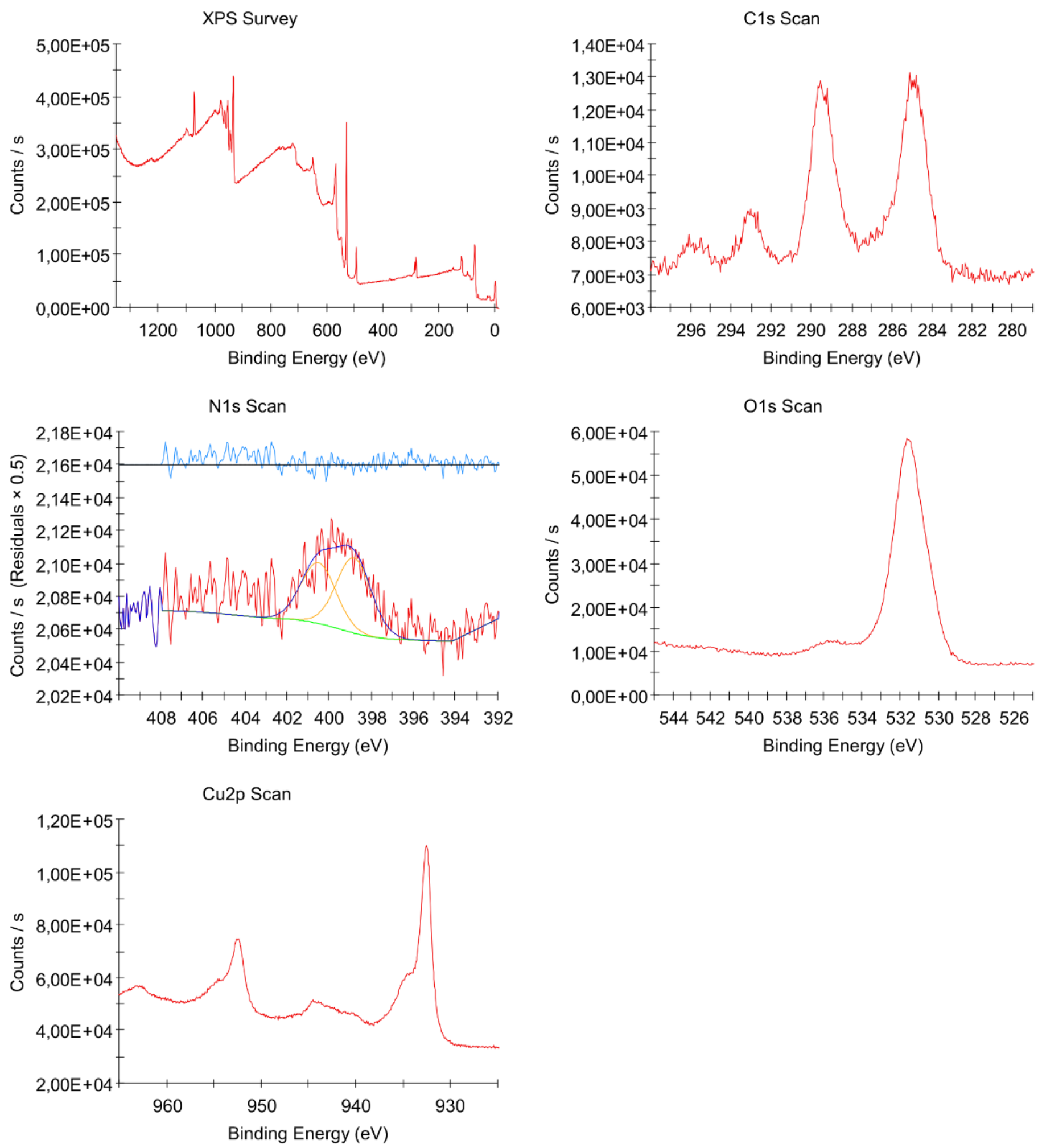

09.02.2026 11:13:50 O:\ANP\GLO_0123_ANP-AB-022\ANP-AB-022 hexane.vgp

Figure S43: Survey and element spectra accompanying the N 1s XPS spectrum displayed in Figure 4 f. Sample preparation details: Cu slide and 5 mg BIMeH·HCO_3 in 1 ml n-hexane for 200 minutes. Washed with CH_2Cl_2, MeOH and again CH_2Cl_2.

4.7 Data associated with Figure 6

Peaks					
Name	Peak BE	FWHM eV	Area (P) CPS.eV	Atomic %	Q
N1s	400.90	1.60	7733.16	73.77	Yes
N1s Scan A	398.64	1.60	2753.75	26.23	Yes

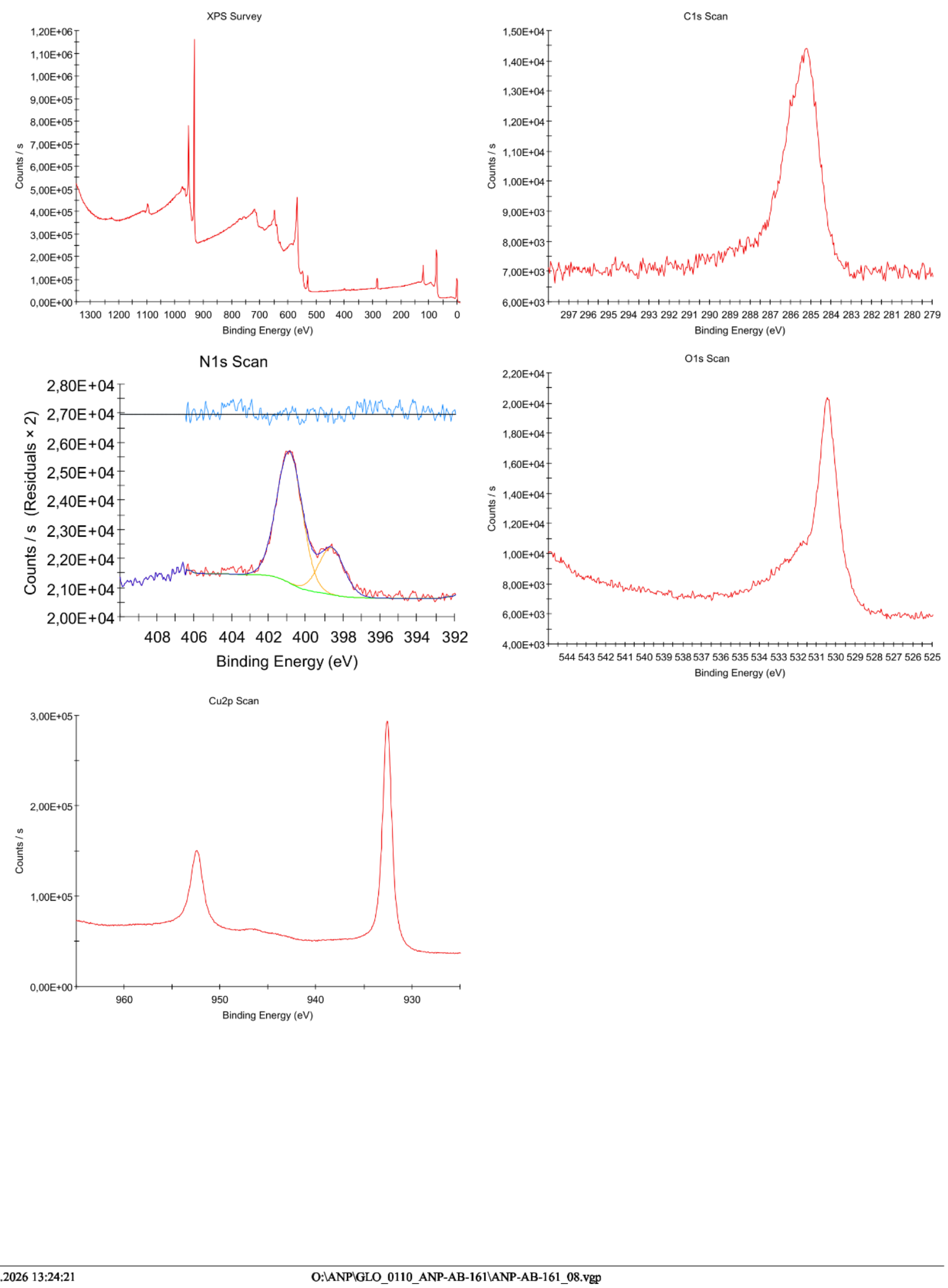

Figure S44: Survey and element spectra accompanying the N 1s XPS spectrum displayed in Figure 6 a. Sample preparation details: Cu slide and 2 mg BIMeH·HCO_3 in 1 ml CH_2Cl_2 for 21 hours. Washed with CH_2Cl_2, MeOH and again CH_2Cl_2.

Peaks					
**Name**	**Peak BE**	**FWHM eV**	**Area (P) CPS.eV**	**Atomic %**	**Q**
N1s	400.73	1.78	2730.05	47.26	Yes
N1s Scan A	398.50	1.78	3051.34	52.74	Yes

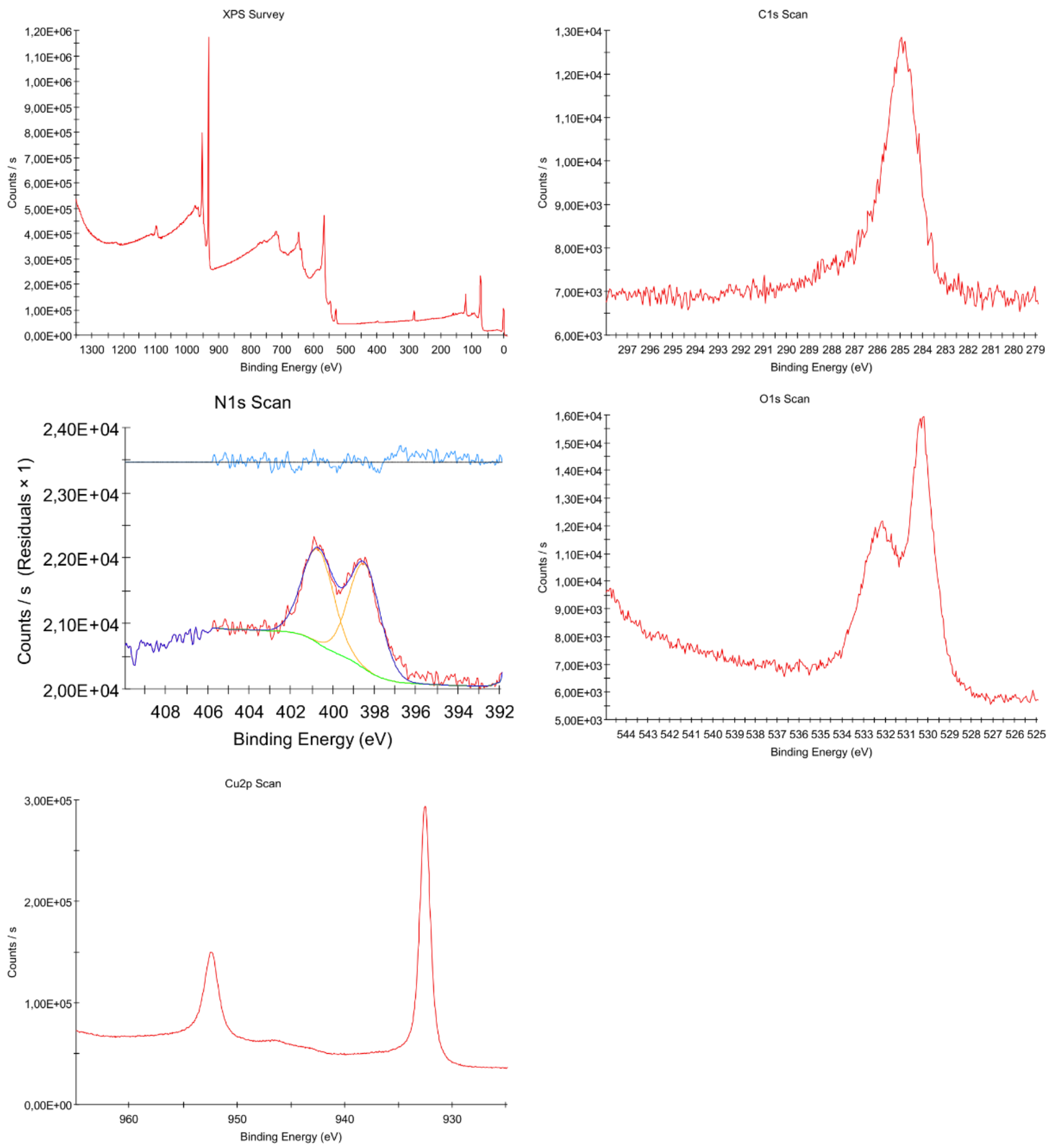

09.02.2026 13:22:27 O:\ANP\GLO_0110_ANP-AB-161\ANP-AB-161_02 control.vgp

Figure S45: Survey and element spectra accompanying the N 1s XPS spectrum displayed in Figure 6 b. Sample preparation details: Cu slide and 2 mg BIMeH·HCO_3 in 1 ml freeze-pump-thawed (3 cycles) CH_2Cl_2 for 21 hours inside a glovebox (O_2 and H_2O levels below 0.5 ppm). Washed with CH_2Cl_2, MeOH and again CH_2Cl_2.

Peaks					
Name	Peak BE	FWHM eV	Area (P) CPS.eV	Atomic %	Q
N1s	400.88	1.63	9822.67	89.14	Yes
N1s Scan A	398.78	1.63	1198.04	10.86	Yes

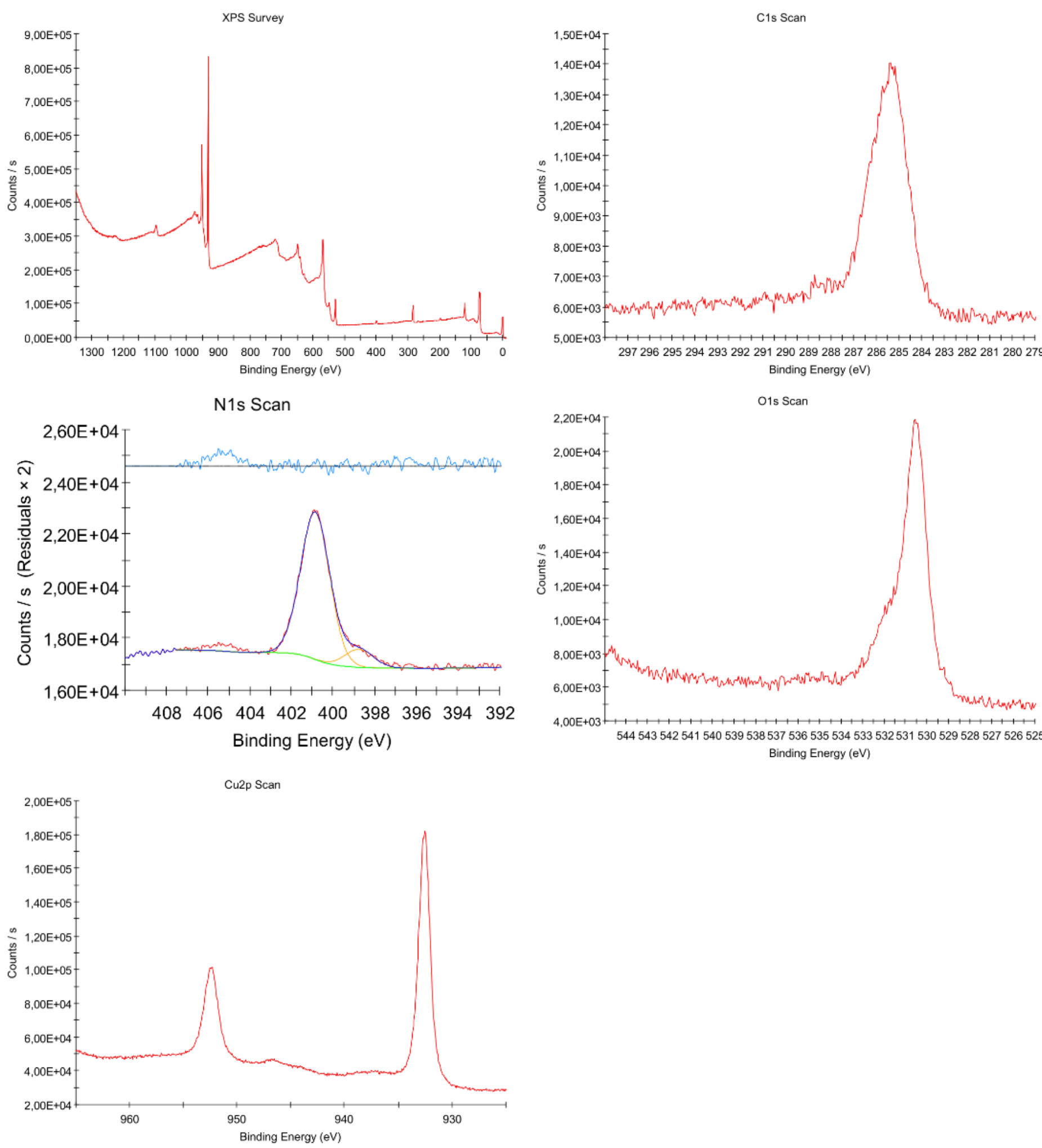

09.02.2026 13:29:14 O:\ANP\GLO_0923_ANP-AB-121_continuation\ANP-AB-093_8_inifinity.vgp

Figure S46: Survey and element spectra accompanying the N 1s XPS spectrum displayed in Figure 6 c. Sample preparation details: Cu slide and 0.5 mg BIMeH·HCO_3 in 0.5 ml freeze-pump-thawed (3 cycles) CH_2Cl_2 for 100 days inside a glovebox (O_2 and H_2O levels below 0.5 ppm). Washed with CH_2Cl_2, MeOH and again CH_2Cl_2.

4.8 Data associated with Figure 7

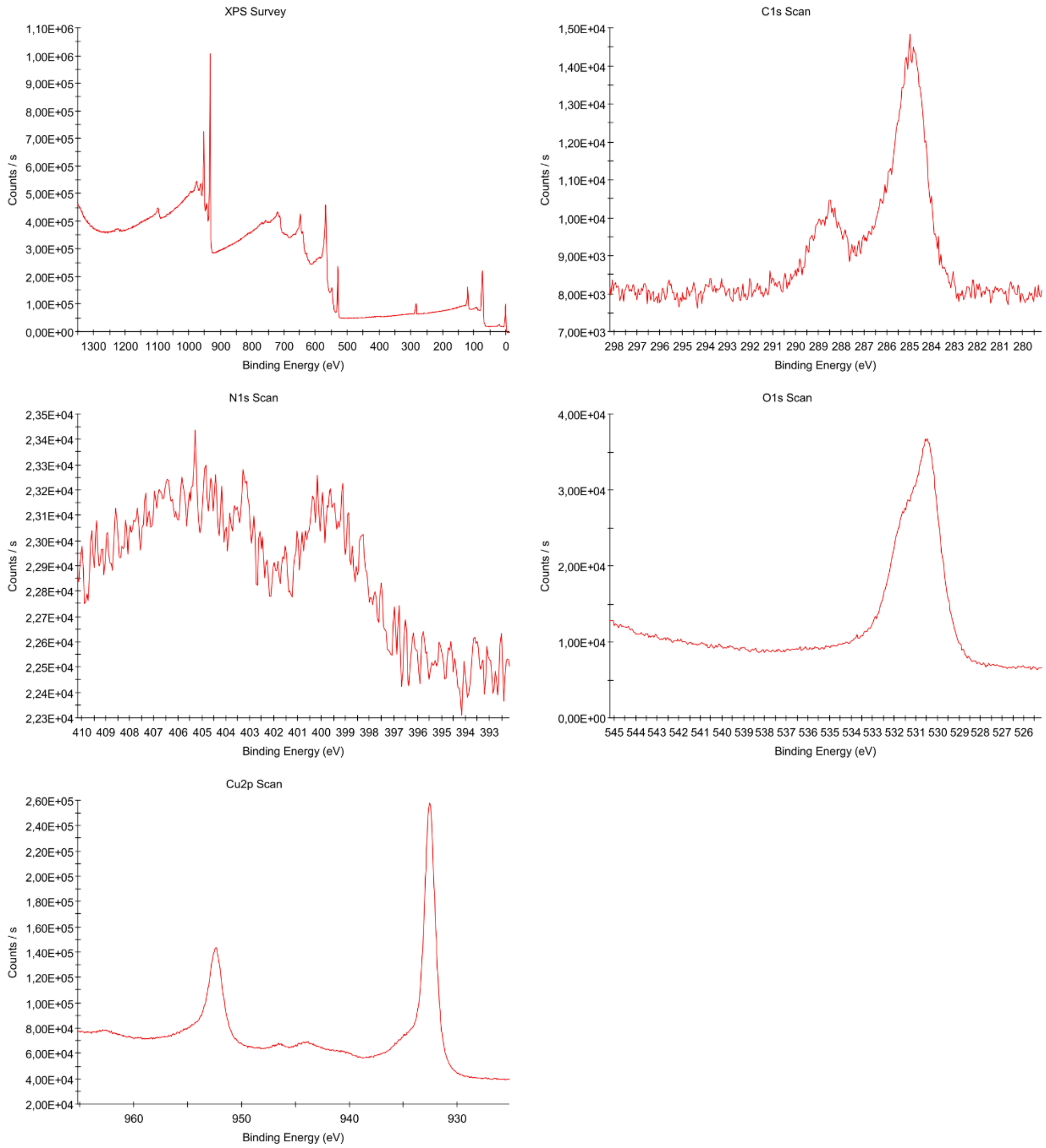

06.02.2026 14:33:39 O:\ANP\GLO_0321_ANP-AB-186_continuation2\ANP-AB-186_blankwashed.vgp

Figure S47: Survey and element spectra accompanying the N 1s XPS spectrum displayed in Figure 7 a. Sample preparation details: Cu slide was cleaned with 2 ml of $EtOH_{abs}$ at 60 °C for 1 h, rinsed with MeOH and dried under reduced pressure (1 mbar).

Peaks					
Name	Peak BE	FWHM eV	Area (P) CPS.eV	Atomic %	Q
N1s	401.08	1.72	43978.89	92.53	Yes
N1s Scan A	398.91	1.72	3554.24	7.47	Yes

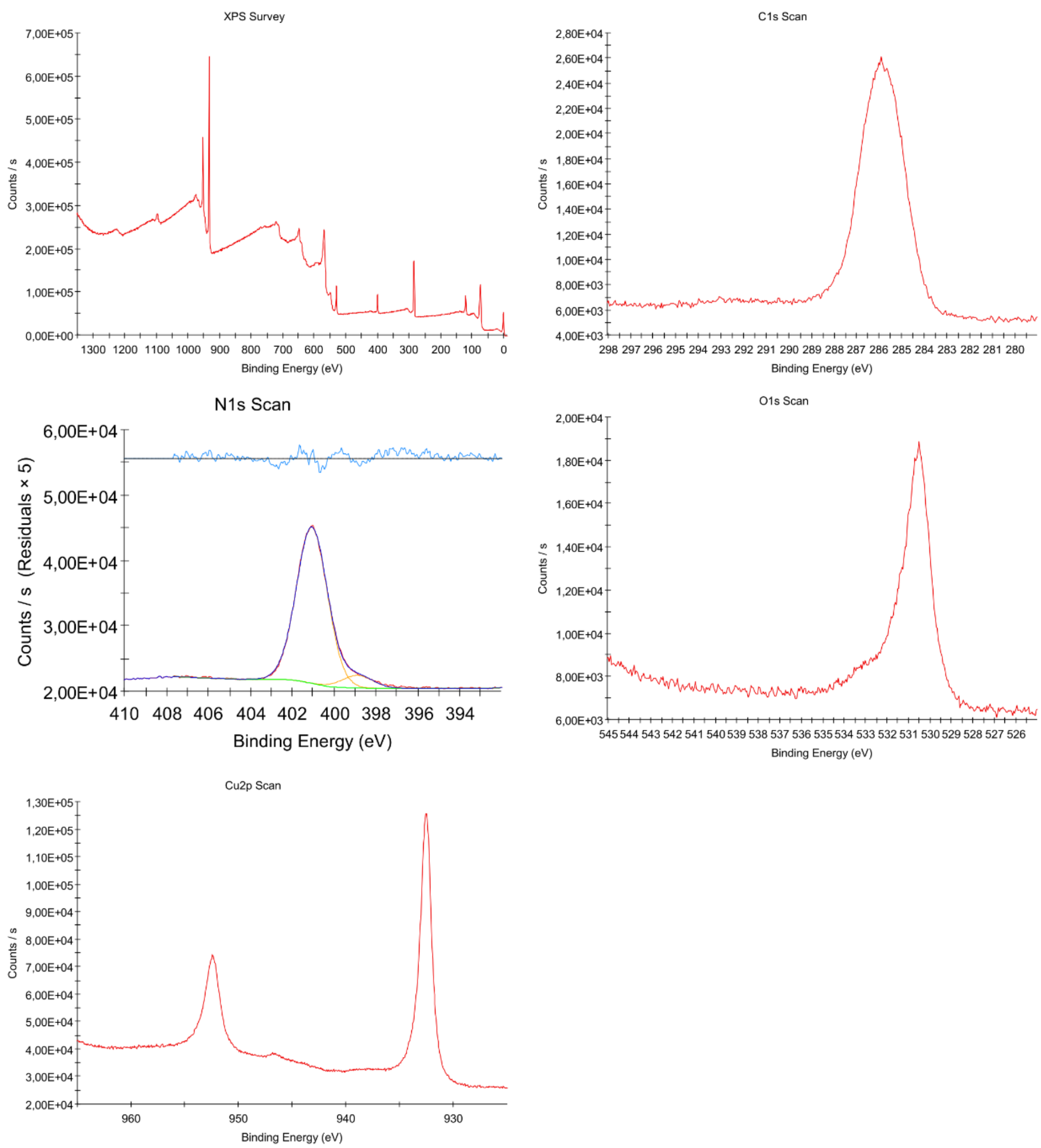

06.02.2026 14:22:51 O:\ANP\GLO_0321_ANP-AB-186_continuation2\ANP-AB-186_06.vgp

Figure S48: Survey and element spectra accompanying the N 1s XPS spectrum displayed in Figure 7 b. Sample preparation details: PVD modification of Cu slide with BIMeH·HCO_3 at 100 °C for 15 minutes.

Peaks					
Name	Peak BE	FWHM eV	Area (P) CPS.eV	Atomic %	Q
N1s	400.99	1.54	10368.72	74.23	Yes
N1s Scan A	398.83	1.54	3604.62	25.77	Yes

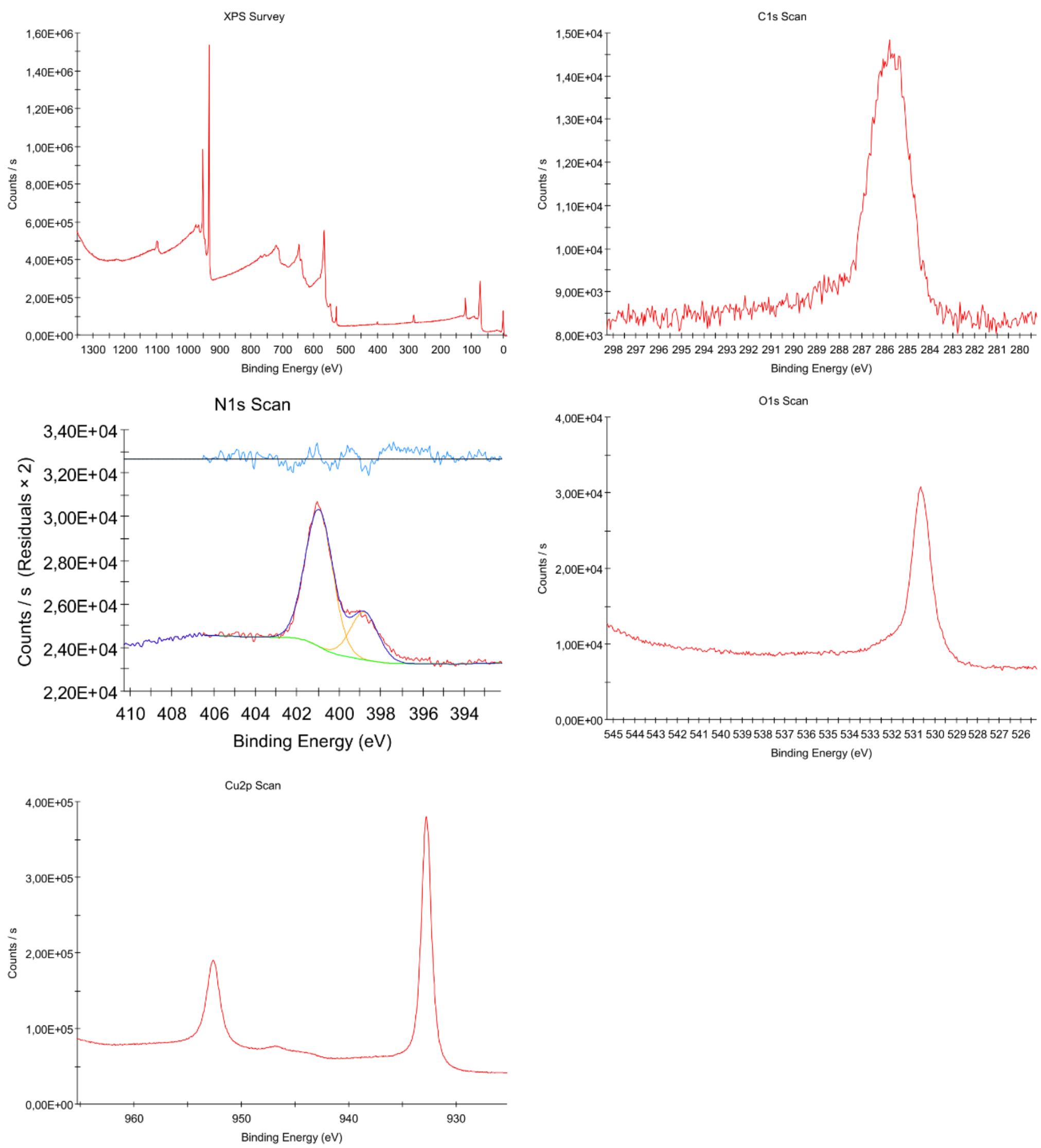

06.02.2026 14:19:59 O:\ANP\GLO_0321_ANP-AB-186_continuation\ANP-AB-186_03.vgp

Figure S49: Survey and element spectra accompanying the N 1s XPS spectrum displayed in Figure 7 c. Sample preparation details: PVD modification of Cu slide with BIMeH·HCO_3 at 100 °C for 15 minutes. Washed with $EtOH_{abs}$ and acetone.

4.9 Data associated with Figure S18

Peaks					
Name	Peak BE	FWHM eV	Area (P) CPS.eV	Atomic %	Q
N1s	400.81	1.54	7645.82	77.39	Yes
N1s Scan A	398.82	1.54	2237.08	22.61	Yes

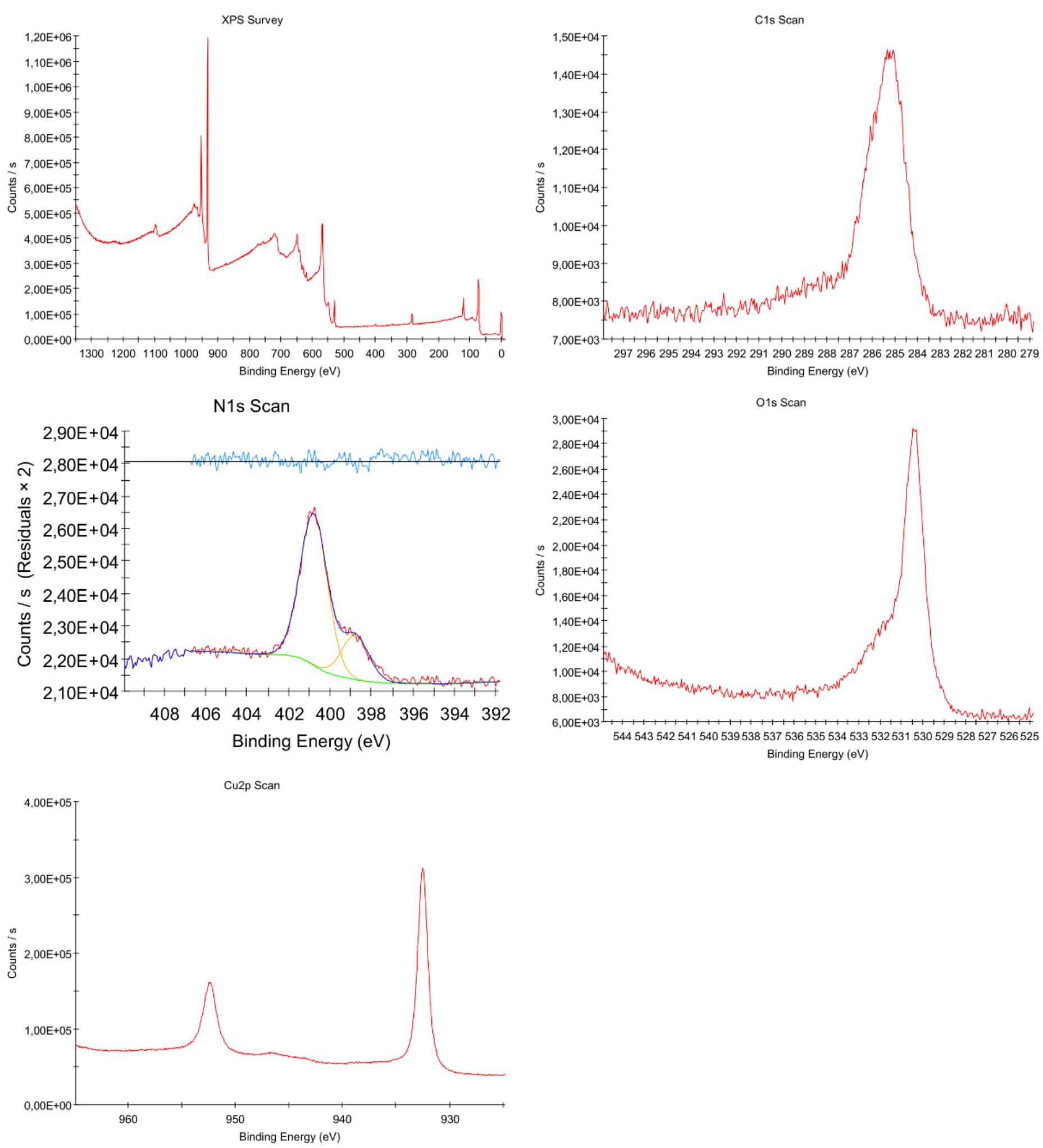

09.02.2026 12:51:50 O:\ANP\2023\2023_beforespec\GLO_1704_ANP-AA-104\ANP-AA-104-3.vgp

Figure S50: Survey and element spectra accompanying the N 1s XPS spectrum displayed in Figure S18 a. Sample preparation details: Cu slide and 8 mg BIMeH·HCO_3 in 2 ml $EtOH_{abs}$ for 3 days. Washed with $EtOH_{abs}$.

Peaks					
Name	Peak BE	FWHM eV	Area (P) CPS.eV	Atomic %	Q
C1s	286.52	1.75	11898.51	9.91	Yes
C1s Scan A	288.51	1.75	3615.20	3.02	Yes
C1s Scan B	285.29	1.75	13827.55	11.51	Yes
N1s	400.82	1.77	15996.10	8.58	Yes
N1s Scan A	398.91	1.77	3423.66	1.83	Yes
Cu2p3	932.57	1.40	722460.20	58.87	Yes
Cu2p1	952.38	1.45	434413.70	0.00	
Cu2p Scan A	946.65	4.85	110436.62	6.27	Yes

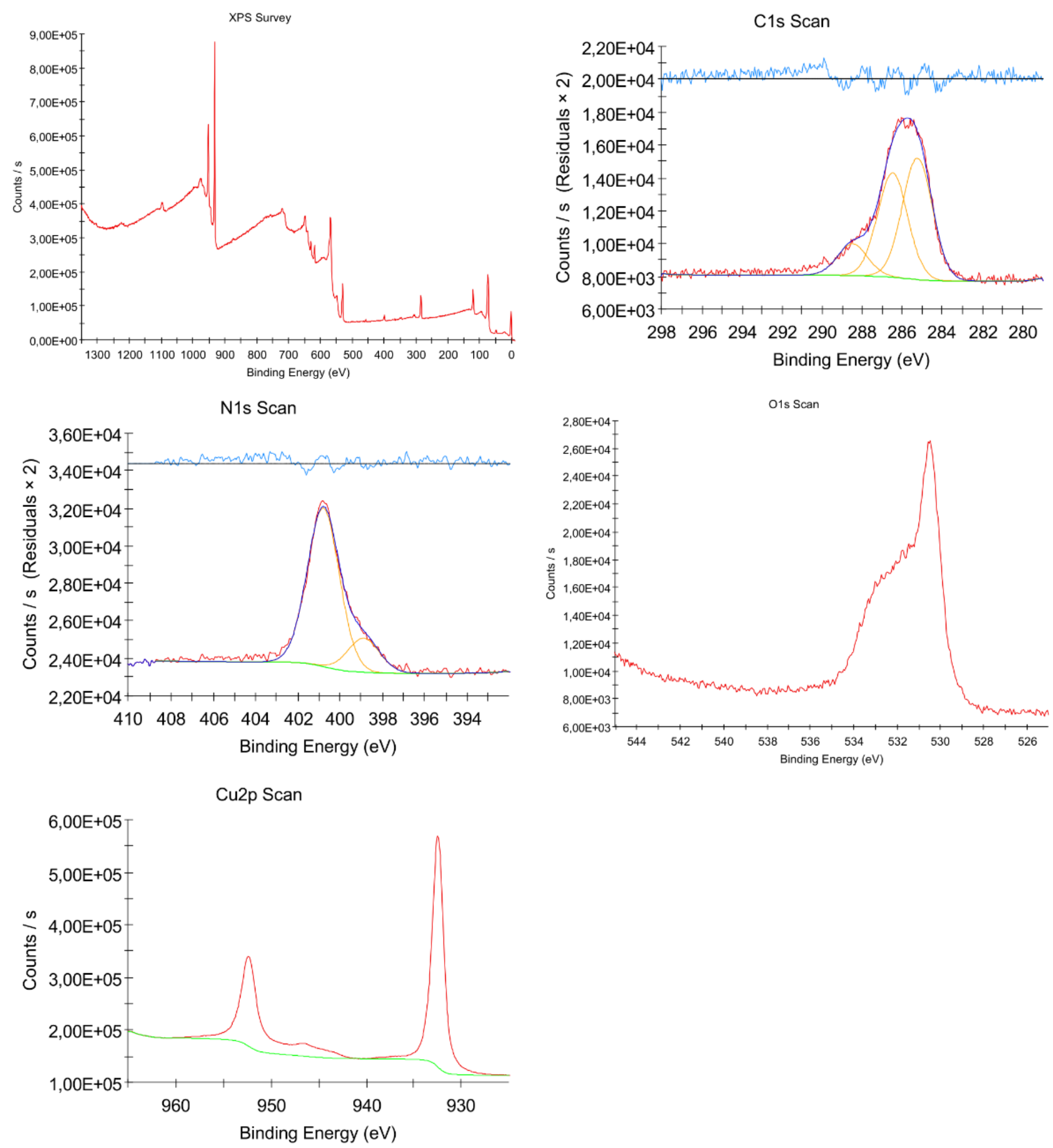

09.02.2026 12:54:25 O:\ANP\GLO_0818_ANP-AC-036\ANP-AC-036 3.vgp

Figure S51: Survey and element spectra accompanying the N 1s XPS spectrum displayed in Figure S18 b. Sample preparation details: Cu slide and 2 mg BIMeH·HCO_3 in 1 ml MeOH for 3 days. Washed with iPrOH.

Peaks

Name	Peak BE	FWHM eV	Area (P) CPS.eV	Atomic %	Q
N1s	400.75	1.88	11207.05	6.89	Yes
N1s Scan A	398.99	1.88	3307.31	2.03	Yes
C1s Scan A	285.07	1.44	14144.87	13.49	Yes
C1s Scan C	286.36	1.44	9137.19	8.72	Yes
Cu2p1 Scan B	952.63	1.43	310495.41	0.00	
Cu2p3 Scan B	932.79	1.43	673516.61	62.89	Yes
C1s Scan E	288.40	2.42	6265.49	5.99	Yes

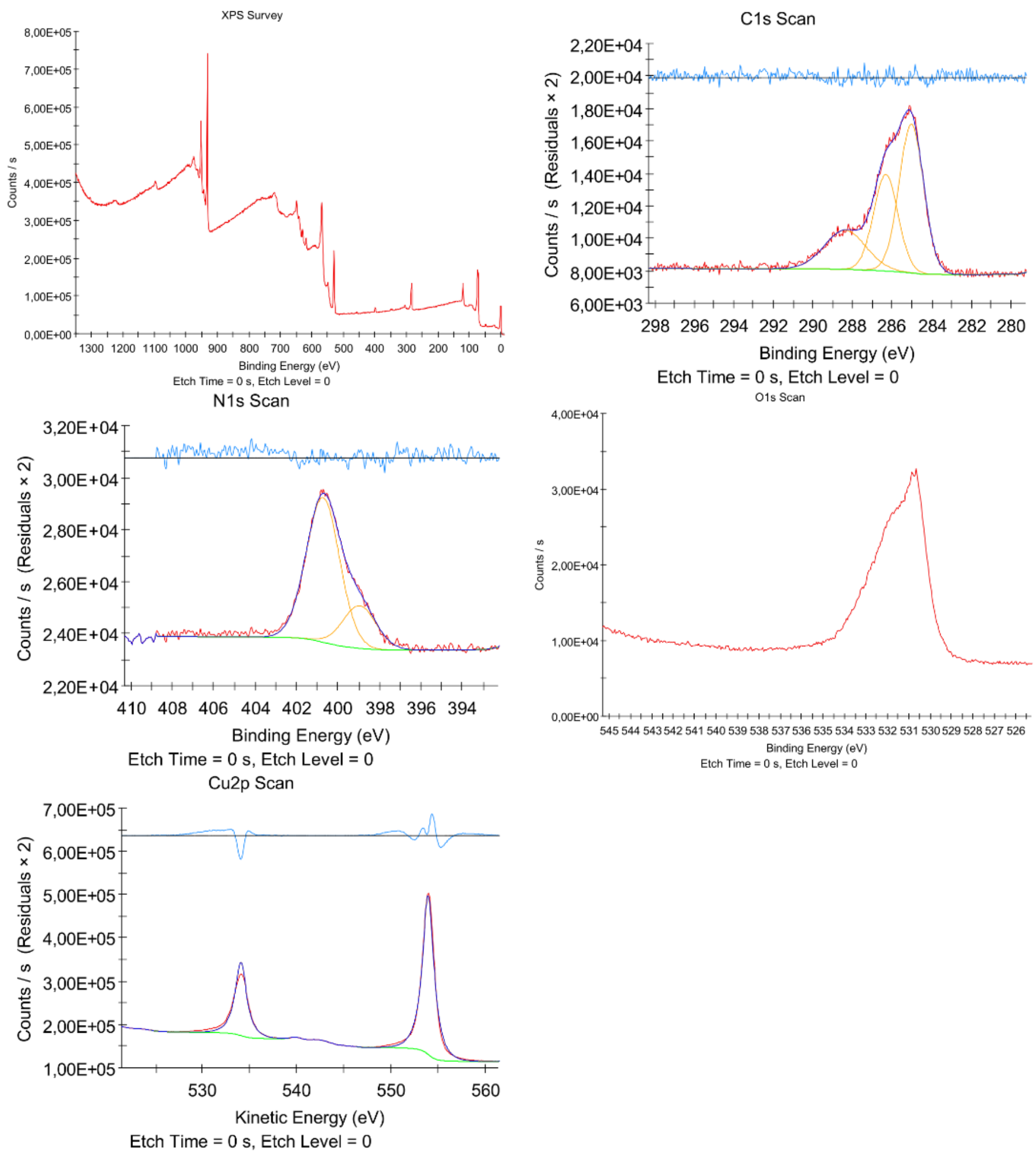

09.02.2026 12:57:10 O:\ANP\GLO_0829_ANP-AC-038\ANP-AC-036 3 iongun.vgp

Figure S52: Survey and element spectra accompanying the N 1s XPS spectrum displayed in Figure S18 b. Sample preparation details: Cu slide and 2 mg BIMeH·HCO_3 in 1 ml MeOH for 3 days. Washed with iPrOH. Measured directly before ion-gun sputtering.

Peaks

Name	Peak BE	FWHM eV	Area (P) CPS.eV	Atomic %	Q
N1s	400.75	2.42	1480.78	0.49	Yes
N1s Scan A	398.99	2.42	1364.55	0.45	Yes
C1s Scan A	285.09	2.42	1835.57	0.95	Yes
C1s Scan C	286.43	2.42	235.02	0.12	Yes
Cu2p1 Scan B	952.67	1.30	894561.09	0.00	
Cu2p3 Scan B	932.82	1.30	1940452.21	97.90	Yes
C1s Scan E	292.48	0.43	161.66	0.08	Yes

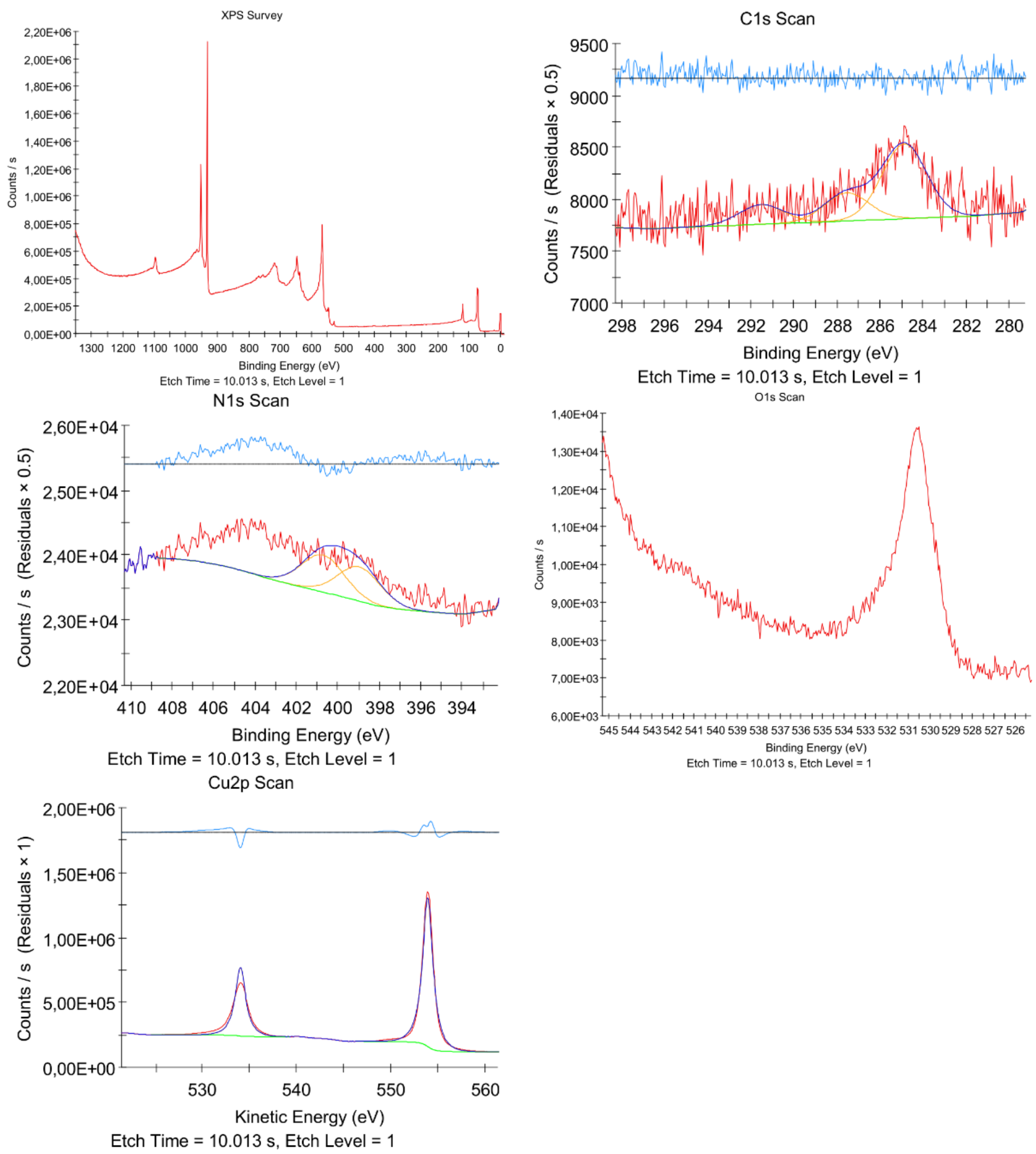

09.02.2026 12:57:54 O:\ANP\GLO_0829_ANP-AC-038\ANP-AC-036 3 iongun.vgp

Figure S53: Survey and element spectra accompanying the N 1s XPS spectrum displayed in Figure S18 b. Ion-gun sputtered sample with negligible N 1s and C 1s residues and Cu(0) characteristics.

4.10 Data associated with Figure S19

Peaks					
Name	Peak BE	FWHM eV	Area (P) CPS.eV	Atomic %	Q
N1s	401.16	2.17	7655.61	64.94	Yes
N1s Scan A	398.71	2.17	4139.92	35.06	Yes

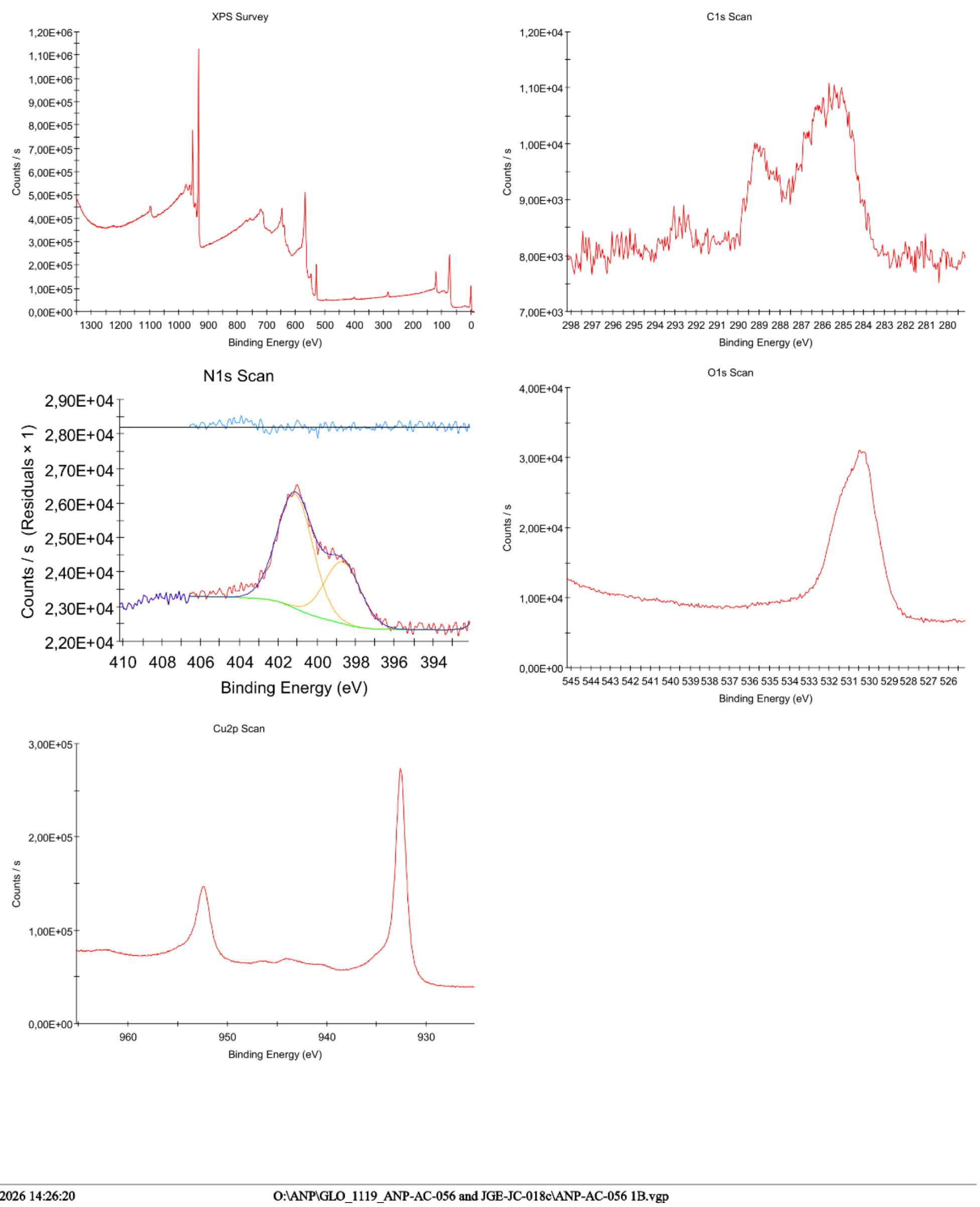

Figure S54: Survey and element spectra accompanying the N 1s XPS spectrum displayed in Figure S19 a. Sample preparation details: Cu slide and 5 mg $IMeCO_2$ in 1 ml CH_2Cl_2 at -18 °C for 42 hours and covered from light by aluminum foil. Washed with iPrOH.

Peaks					
Name	Peak BE	FWHM eV	Area (P) CPS.eV	Atomic %	Q
N1s	398.61	2.13	12982.46	53.03	Yes
N1s Scan A	401.06	2.13	11477.05	46.97	Yes

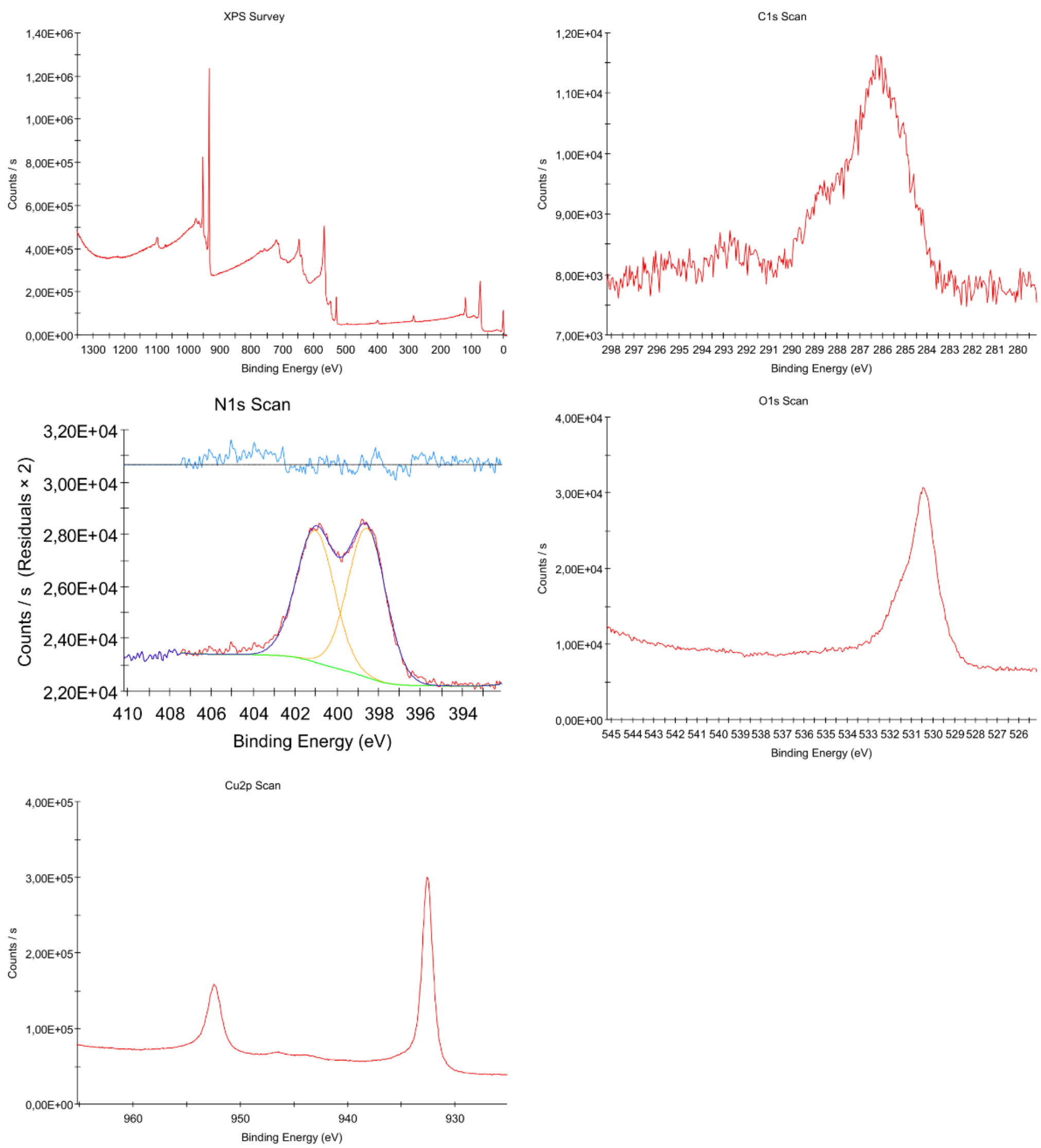

06.02.2026 14:26:49 O:\ANP\GLO_1119_ANP-AC-056 and JGE-JC-018c\ANP-AC-056 2B.vgp

Figure S55: Survey and element spectra accompanying the N 1s XPS spectrum displayed in Figure S19 b. Sample preparation details: Cu slide and 5 mg $IMeCO_2$ in 1 ml CH_2Cl_2 at 25 °C for 42 hours and covered from light by aluminum foil. Washed with iPrOH.

Peaks					
Name	Peak BE	FWHM eV	Area (P) CPS.eV	Atomic %	Q
N1s	401.19	2.21	15612.81	60.14	Yes
N1s Scan A	398.77	2.21	10366.95	39.86	Yes

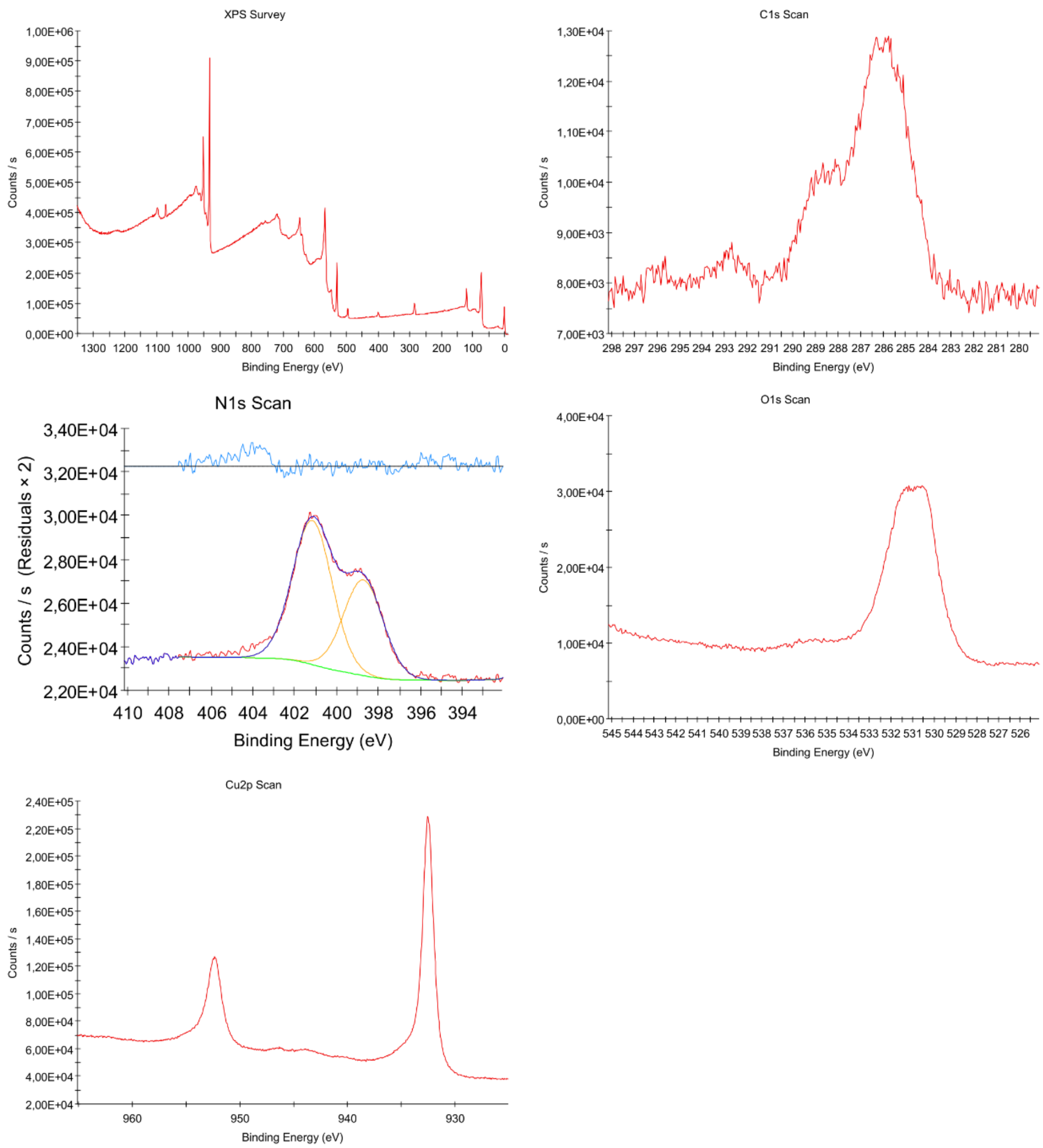

06.02.2026 14:35:45 O:\ANP\GLO_1110_ANP-AC-050\ANP-AC-050 09 A.vgp

Figure S56: Survey and element spectra accompanying the N 1s XPS spectrum displayed in Figure S19 c. Sample preparation details: Cu slide and 5 mg $IMeCO_2$ in 1 ml CH_2Cl_2 at 60 °C for 30 minutes. Washed with iPrOH.

Peaks					
Name	Peak BE	FWHM eV	Area (P) CPS.eV	Atomic %	Q
N1s	400.50	1.93	3060.49	56.04	Yes
N1s Scan A	398.66	1.93	2403.62	43.96	Yes

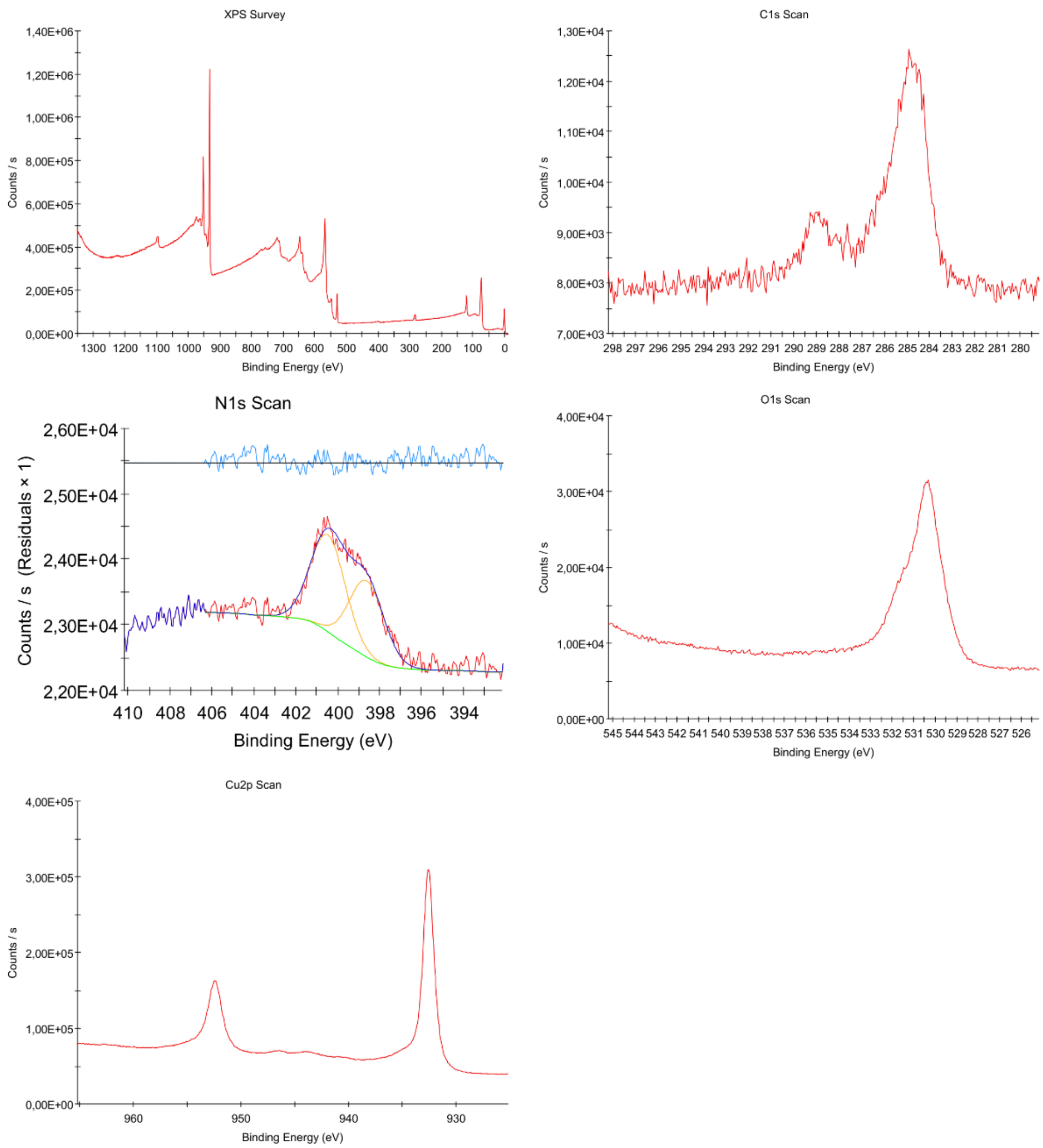

06.02.2026 14:28:03 O:\ANP\GLO_1119_ANP-AC-056 and JGE-JC-018c\ANP-AC-056 3A.vgp

Figure S57: Survey and element spectra accompanying the N 1s XPS spectrum displayed in Figure S19 d. Sample preparation details: Cu slide and 5 mg BIMeH·HCO_3 in 1 ml CH_2Cl_2 at -18 °C for 42 hours and covered from light by aluminum foil. Washed with iPrOH.

Peaks					
Name	Peak BE	FWHM eV	Area (P) CPS.eV	Atomic %	Q
N1s	400.65	1.68	5568.47	63.88	Yes
N1s Scan A	398.56	1.68	3153.03	36.12	Yes

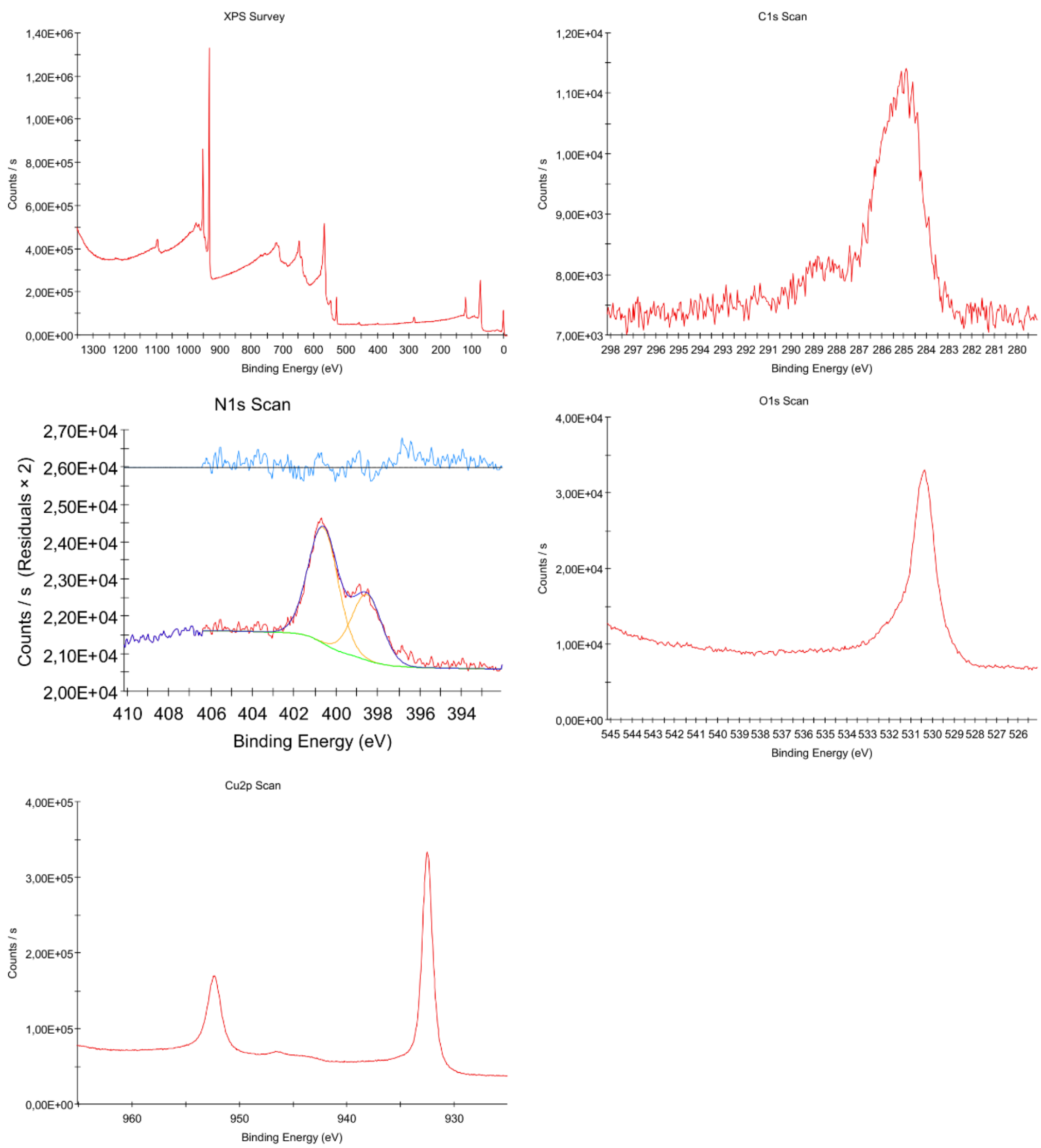

06.02.2026 14:28:45 O:\ANP\GLO_1119_ANP-AC-056 and JGE-JC-018c\ANP-AC-056 4B.vgp

Figure S58: Survey and element spectra accompanying the N 1s XPS spectrum displayed in Figure S19 e. Sample preparation details: Cu slide and 5 mg BIMeH·HCO_3 in 1 ml CH_2Cl_2 at 25 °C for 42 hours and covered from light by aluminum foil. Washed with iPrOH.

Peaks					
Name	Peak BE	FWHM eV	Area (P) CPS.eV	Atomic %	Q
N1s	398.56	1.80	2739.79	51.39	Yes
N1s Scan A	400.42	1.80	2588.06	48.61	Yes

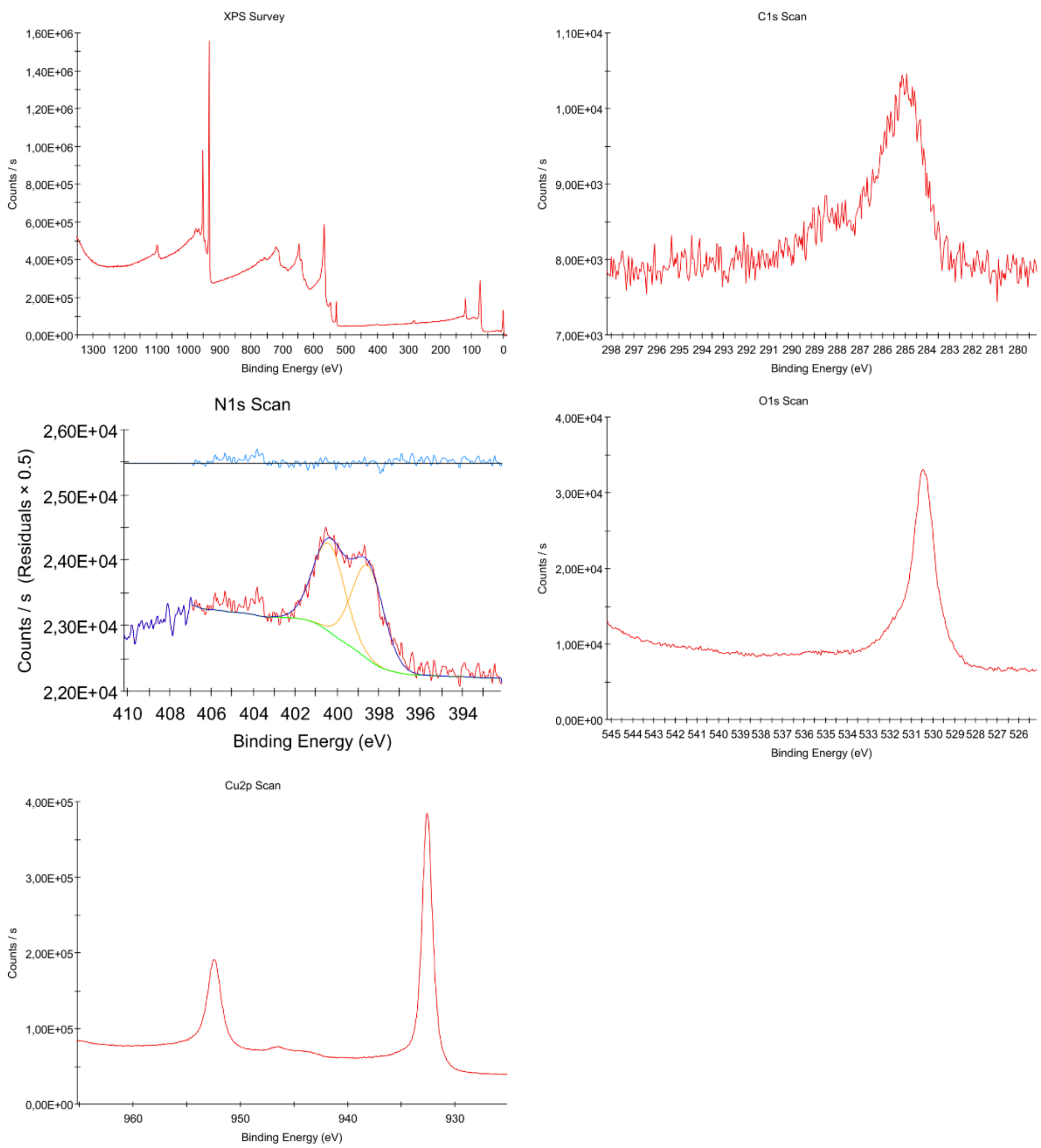

06.02.2026 14:38:28 O:\ANP\GLO_1105_ANP-AC-049\ANP-AC-049 1.vgp

Figure S59: Survey and element spectra accompanying the N 1s XPS spectrum displayed in Figure S19 f. Sample preparation details: Cu slide and 5 mg BIMeH·HCO_3 in 1 ml CH_2Cl_2 at 60 °C for 30 minutes. Washed with iPrOH.

Peaks					
**Name**	**Peak BE**	**FWHM eV**	**Area (P) CPS.eV**	**Atomic %**	**Q**
N1s	400.86	1.55	10228.50	4.86	Yes
N1s Scan A	398.76	1.55	2208.68	1.05	Yes
Cu2p3 Scan A	932.60	1.27	1303597.64	94.09	Yes
Cu2p1 Scan A	952.40	1.27	601023.39	0.00	

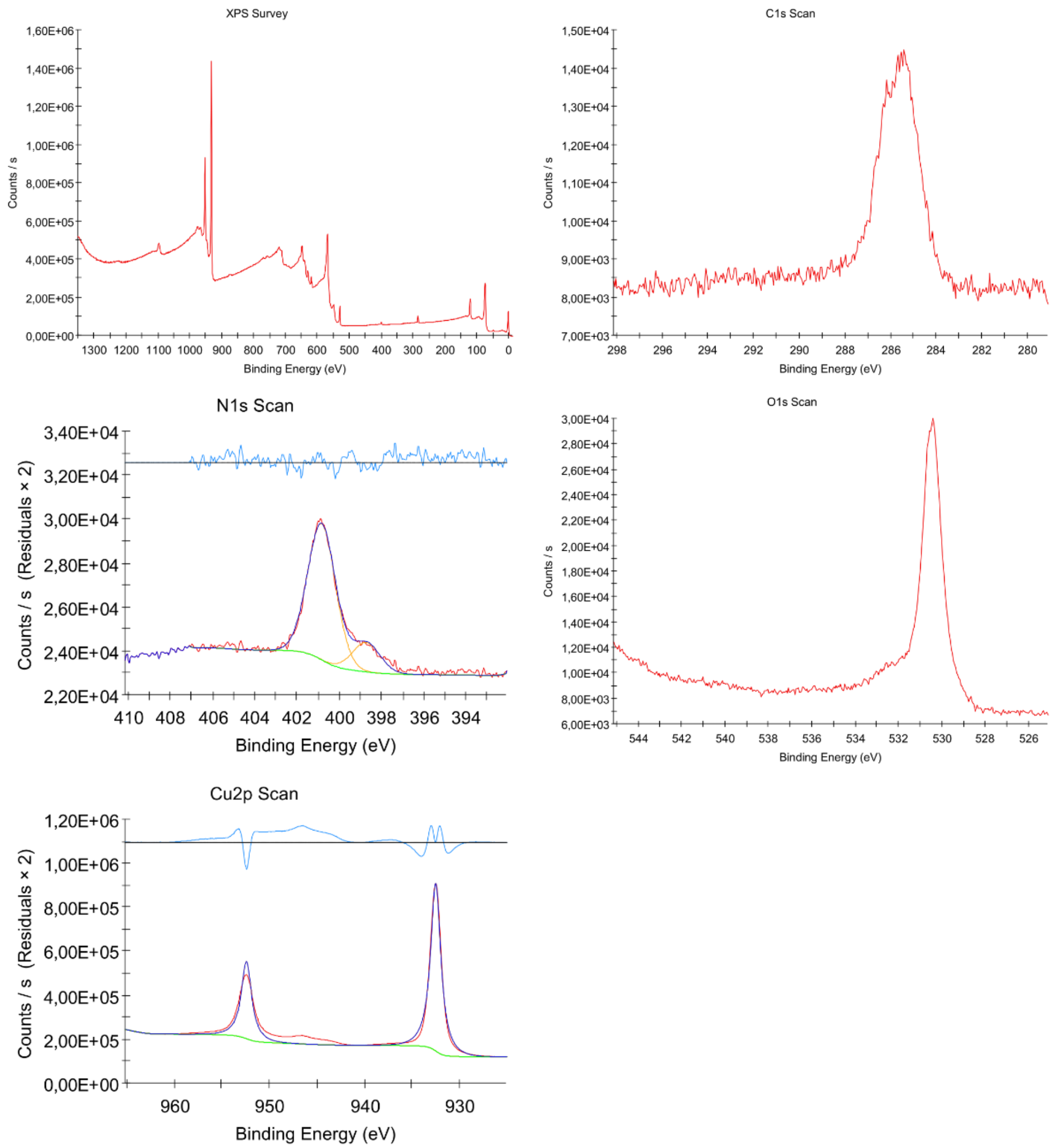

06.02.2026 14:45:06 O:\ANP\GLO_0818_ANP-AC-036\ANP-AC-036 6.vgp

Figure S60: Survey and element spectra accompanying the N 1s XPS spectrum displayed in Figure S19 h. Sample preparation details: Cu slide and 2 mg BIMeH·HCO_3 in 1 ml CH_2Cl_2 for 3 days covered from light by aluminum foil. Washed with iPrOH.

Quantified data at axis location :

Axis Label	**Current**	Min.	Max.	Units
Etch Time	**0.000**	0.000	10.013	s
Etch Level	**0**	0	1	

**Peaks**					
**Name**	**Peak BE**	**FWHM eV**	**Area (P) CPS.eV**	**Atomic %**	**Q**
Cu2p3 Scan A	932.62	1.80	810702.08	91.36	Yes
Cu2p1 Scan A	952.42	1.80	373735.64	0.00	
N1s	400.75	1.72	9097.85	6.75	Yes
N1s Scan A	398.79	1.72	2541.43	1.88	Yes

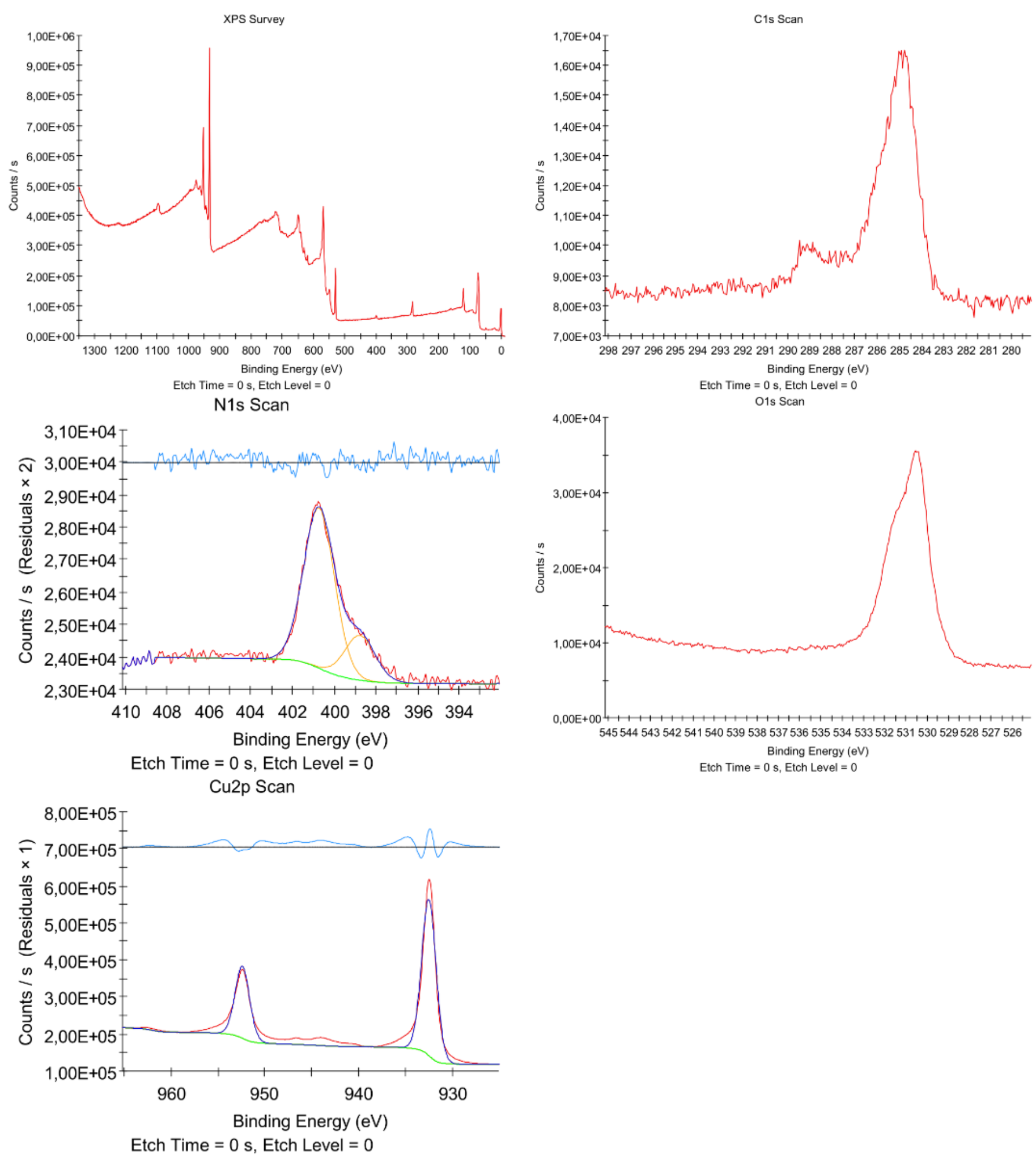

06.02.2026 14:57:08 O:\ANP\GLO_0829_ANP-AC-038\ANP-AC-036 6 iongun.vgp

Figure S61: Survey and element spectra accompanying the N 1s XPS spectrum displayed in Figure S19 h. Sample preparation details: Cu slide and 2 mg BIMeH·HCO_3 in 1 ml CH_2Cl_2 for 3 days covered from light by aluminum foil. Washed with iPrOH. Measured directly before ion-gun sputtering.

Quantified data at axis location :

Axis Label	**Current**	Min.	Max.	Units
Etch Time	**10.013**	0.000	10.013	s
Etch Level	**1**	0	1	

**Peaks**					
**Name**	**Peak BE**	**FWHM eV**	**Area (P) CPS.eV**	**Atomic %**	**Q**
Cu2p3 Scan A	932.67	1.29	2078004.27	98.82	Yes
Cu2p1 Scan A	952.48	1.29	958078.18	0.00	
N1s	403.98	2.42	2288.91	0.72	Yes
N1s Scan A	399.75	2.42	1465.54	0.46	Yes

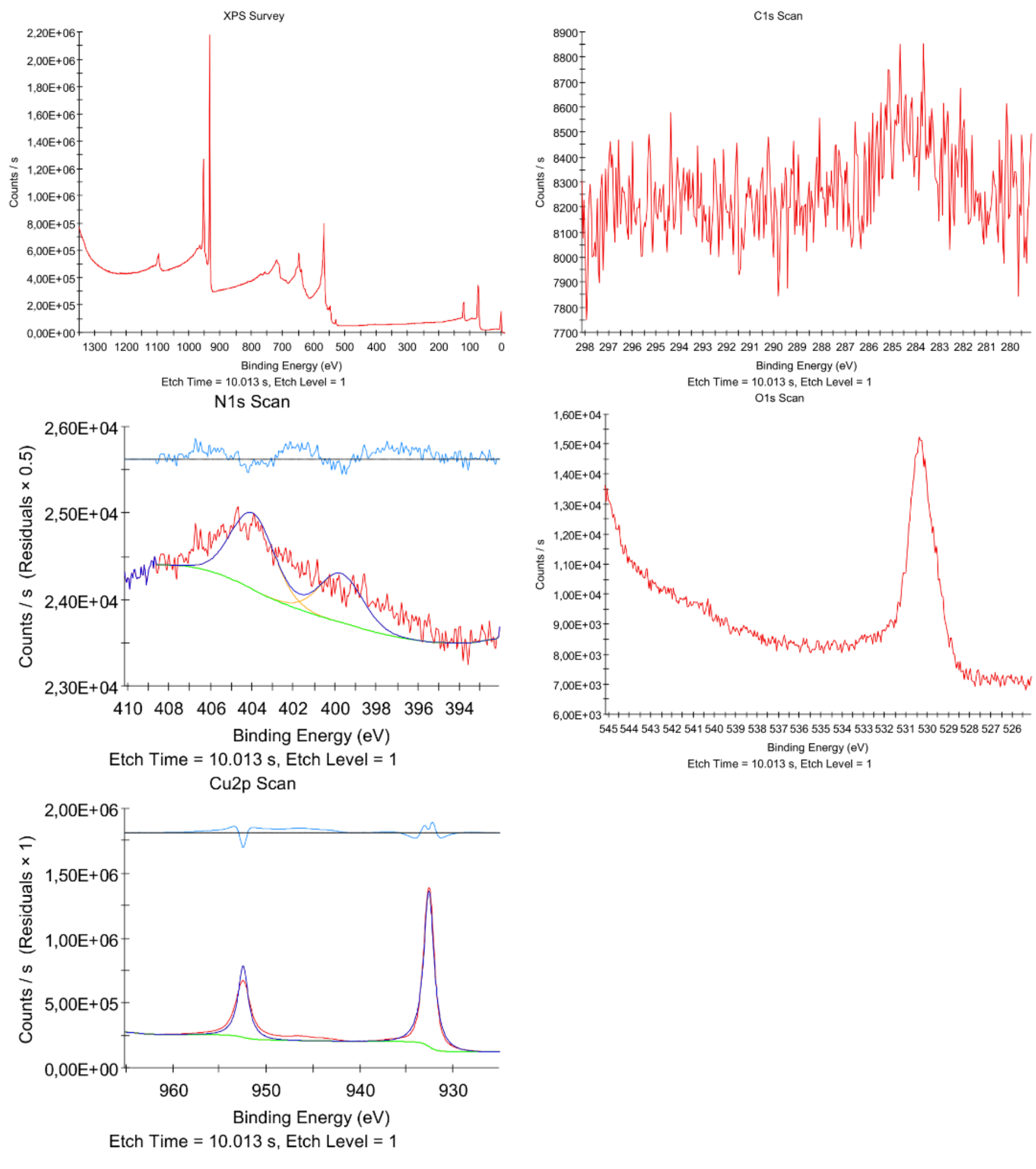

06.02.2026 14:57:38 O:\ANP\GLO_0829_ANP-AC-038\ANP-AC-036 6 iongun.vgp

Figure S62: Survey and element spectra accompanying the N 1s XPS spectrum displayed in Figure S19 h. Ion-gun sputtered sample with negligible N 1s and C 1s residues and Cu(0) characteristics.

Peaks					
Name	Peak BE	FWHM eV	Area (P) CPS.eV	Atomic %	Q
N1s	400.08	1.91	6890.28	3.82	Yes
N1s Scan A	398.44	1.91	2365.27	1.31	Yes
Cu2p3 Scan A	932.58	1.31	1125883.54	94.87	Yes
Cu2p1 Scan A	952.38	1.31	519078.88	0.00	

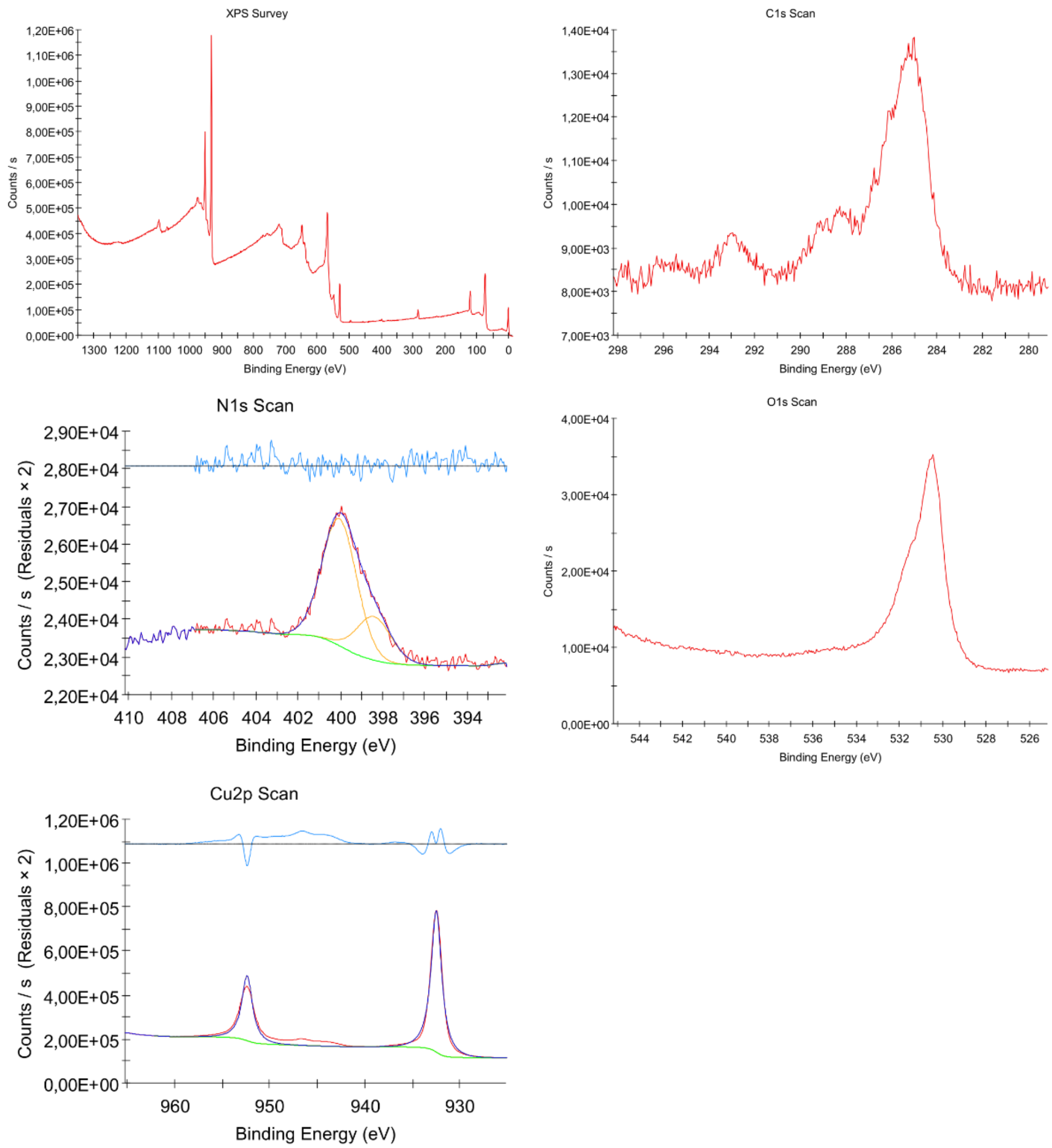

06.02.2026 14:42:32 O:\ANP\GLO_0818_ANP-AC-036\ANP-AC-036 5-1.vgp

Figure S63: Survey and element spectra accompanying the N 1s XPS spectrum displayed in Figure S19 i. Sample preparation details: Cu slide and 2 mg BIMeH·HCO_3 in 1 ml CH_2Cl_2 for 90 minutes at 450 nm LED irradiation. Washed with iPrOH.

4.11 Repeated measurements of the same sample

Peaks					
Name	Peak BE	FWHM eV	Area (P) CPS.eV	Atomic %	Q
N1s	400.86	1.64	6625.30	76.09	Yes
N1s Scan A	398.74	1.64	2085.37	23.91	Yes

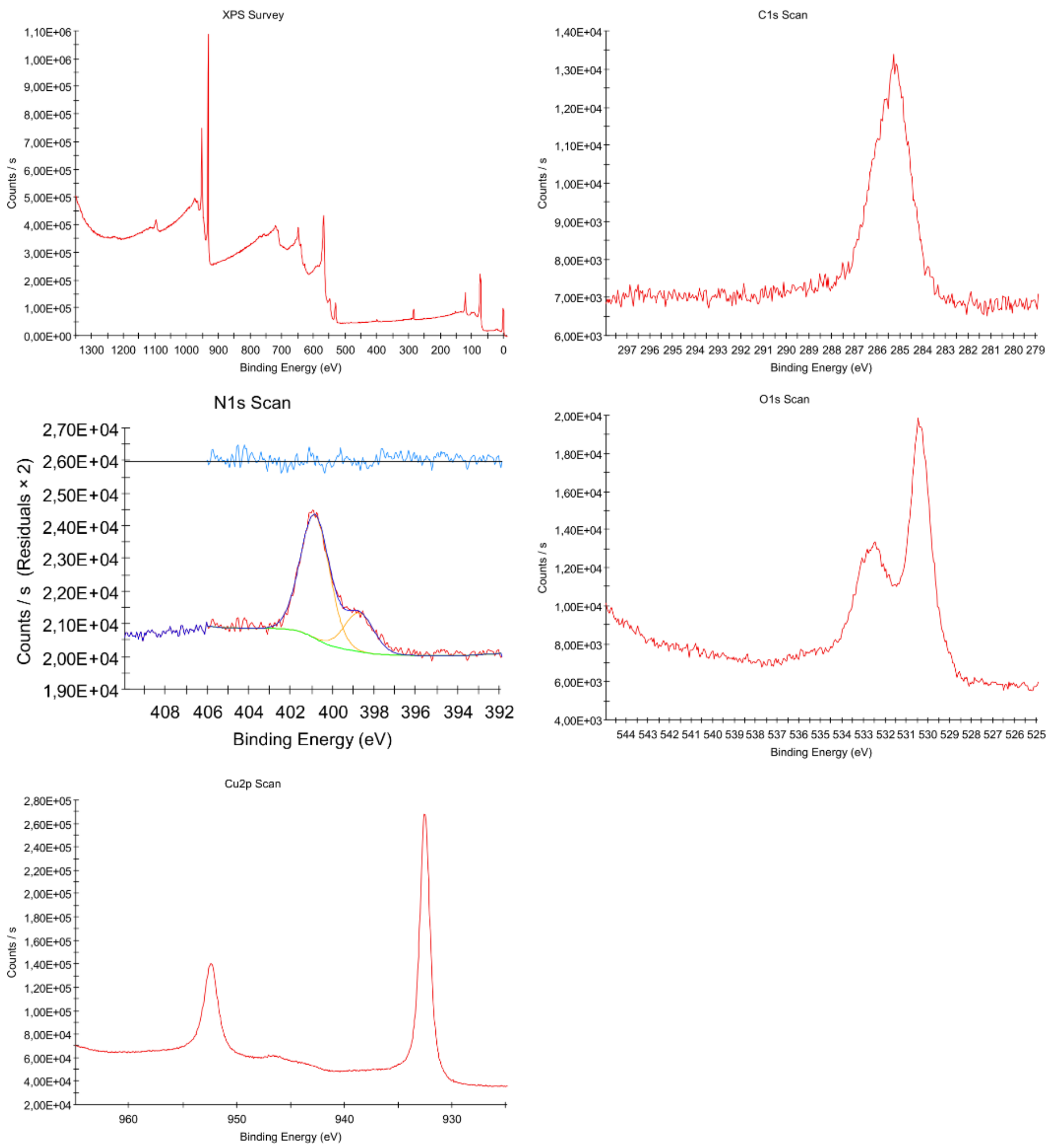

26.03.2026 13:07:10 O:\ANP\GLO_0117_ANP-AB-162\ANP-AB-162_04 control.vgp

Figure S64: Survey and element spectra of a Cu slide modified with BIMe. Sample preparation details: Cu slide and 4 mg BIMeH·HCO_3 in 2 ml CH_2Cl_2 and 100 µl MeOH for 3 h. Washed with MeOH and acetone.

Peaks					
Name	Peak BE	FWHM eV	Area (P) CPS.eV	Atomic %	Q
N1s	400.48	1.74	5984.27	74.61	Yes
N1s Scan A	398.82	1.74	2038.78	25.39	Yes

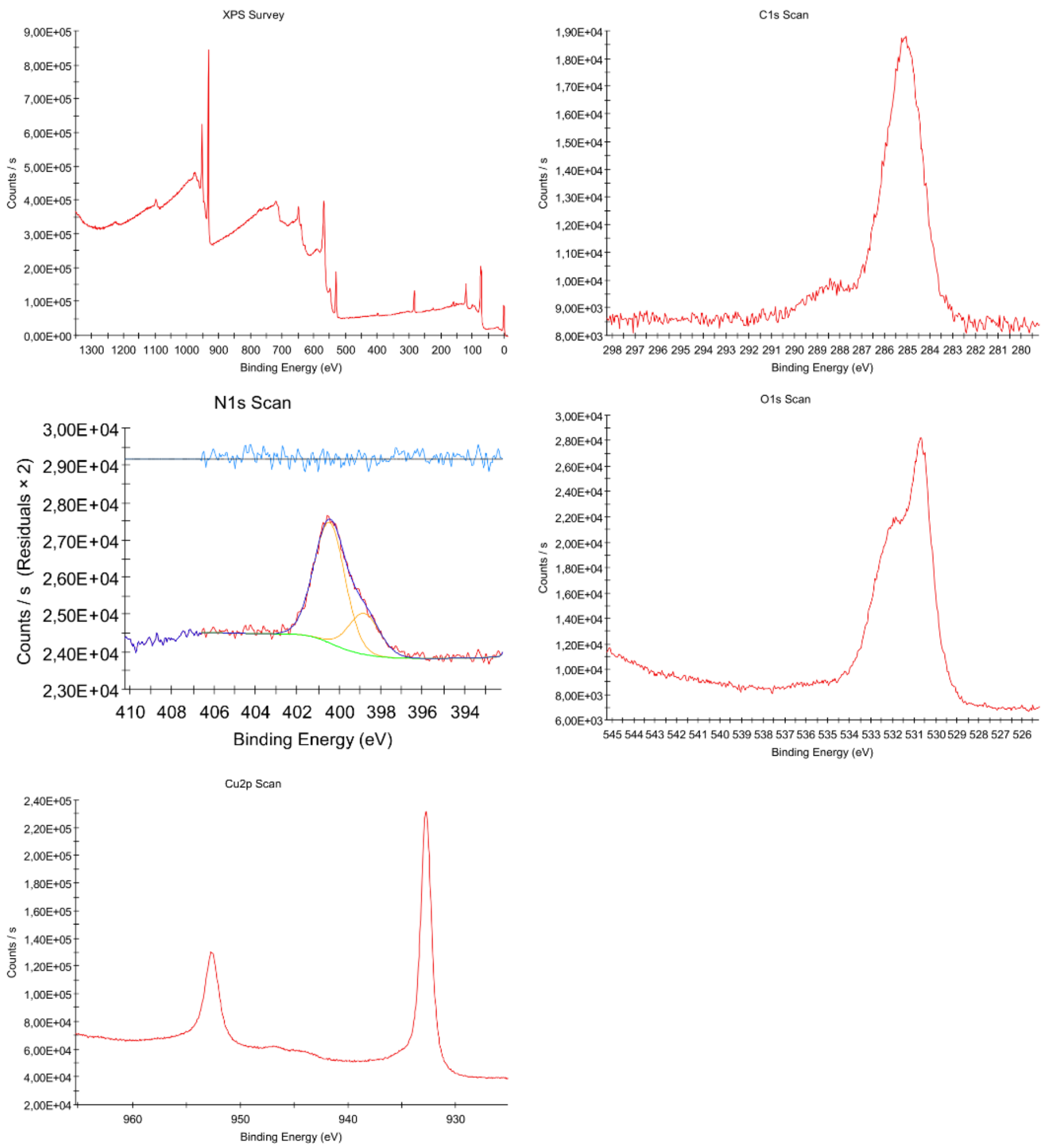

26.03.2026 13:08:14 O:\ANP\GLO_0117_ANP-AB-162\ANP-AB-162 04 6weekslater.vgp

Figure S65: Survey and element spectra of the same sample as depicted in Figure S64 but after 6 weeks of storage under air.

Peaks					
Name	Peak BE	FWHM eV	Area (P) CPS.eV	Atomic %	Q
N1s	400.53	1.81	5582.45	67.89	Yes
N1s Scan A	398.88	1.81	2643.83	32.11	Yes

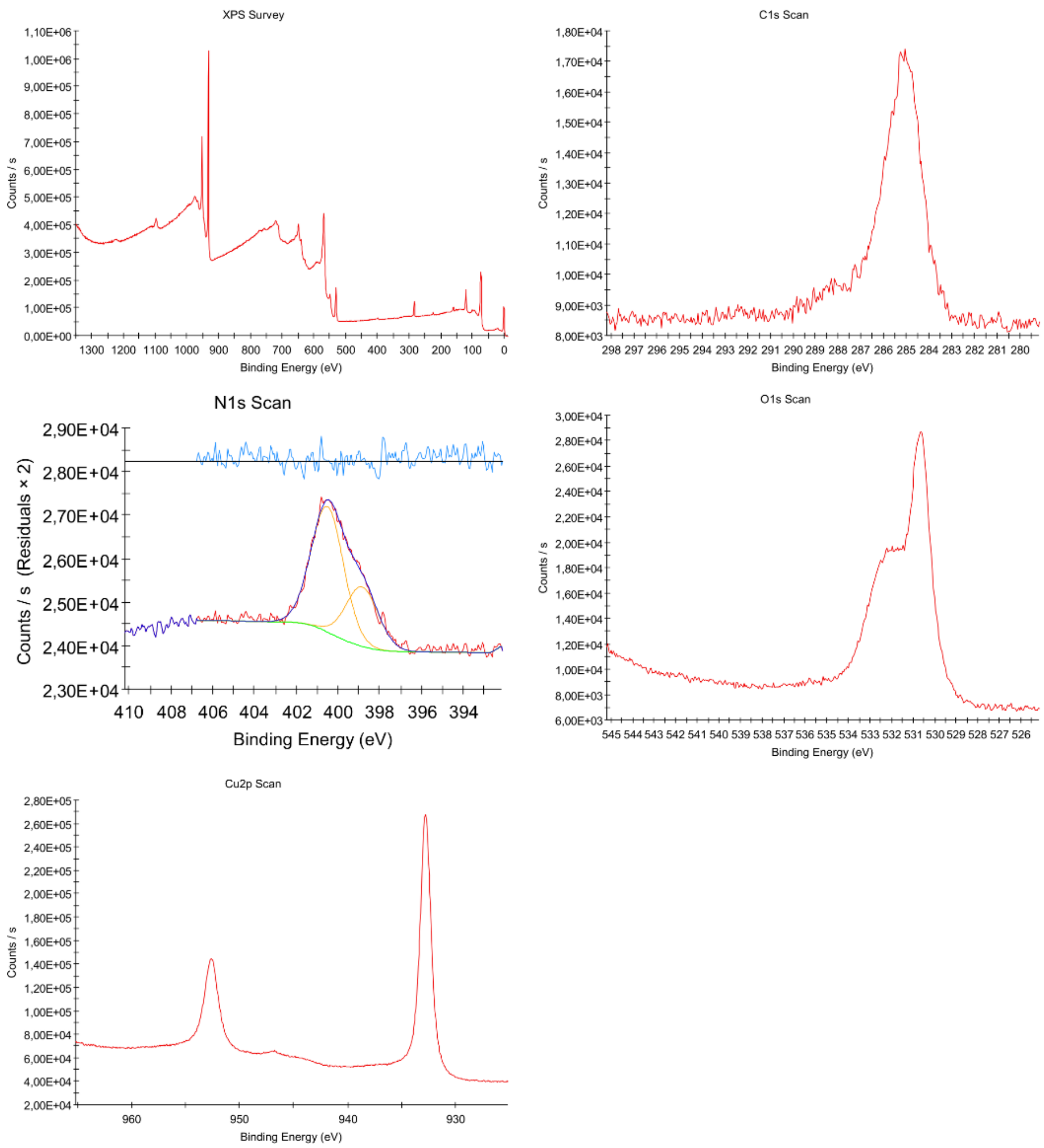

26.03.2026 13:09:16 O:\ANP\GLO_0117_ANP-AB-162\ANP-AB-162 04 6weekslater again.vgp

Figure S66: Survey and element spectra of the same sample as depicted in Figure S64and Figure S65. Remeasurement of the same spot as in Figure S65.

5 XPS Surface Coverage Estimation

To distinguish between monolayer assembly and multilayer formation, the surface coverage was estimated. Therefore, some samples with high N 1s signal intensities were remeasured with adapted measurement parameters and with an iongun sputter cycle.

The sample modification procedures and the respective XPS data can be found in Figure S52 and S53 for modification in MeOH and in Figure S61 and S62 for modification in CH_2Cl_2. The coverage was estimated based on the following equations:[1,12–14]

$$\frac{n_{NHC}}{A} = \frac{1}{2}\frac{I_{N1s}^{rel}}{I_{Cu2p3}^{rel}}\,\lambda_{Cu2p3}^{Cu}\,\frac{n_{Cu}}{V} \times e^{\frac{-d}{\lambda_{Cu2p3}^{org}}} \qquad (1)$$

$$\frac{I_{Cu2p3}}{I_{Cu2p3}^{0}} = e^{\frac{-d}{\lambda_{Cu2p3}^{org}}} \quad (2)$$

$$\frac{n_{NHC}}{A} = \frac{1}{2}\frac{I_{N1s}^{rel}}{I_{Cu2p3}^{rel}}\,\lambda_{Cu2p3}^{Cu}\,\frac{n_{Cu}}{V} \times \frac{I_{Cu2p3}}{I_{Cu2p3}^{0}} \qquad (3)$$

Table S2: Overview of XPS measured XPS parameters relevant for the coverage estimation.

MeOH	Area I [cps*eV]	Atomic % I^{rel}
N 1s 400.7 eV	11207.05	9.60
N 1s 398.8 eV	3307.31	2.82
Cu $2p_{3/2}$	673516.61	87.58
Cu $2p_{3/2}$ (0)	1940452.21	-

CH_2Cl_2	Area I [cps*eV]	Atomic % I^{rel}
N 1s 400.7 eV	9097.85	6.75
N 1s 398.8 eV	2541.43	1.88
Cu $2p_{3/2}$	810702.08	91.36
Cu $2p_{3/2}$ (0)	2078004.27	-

The resulting coverages are 4.4 nm^{-2} in MeOH and 3.3 nm^{-2} in CH_2Cl_2, respectively. Both values are in good agreement with previously reported NHC monolayer coverages. The slightly lower value obtained from surface modification in CH_2Cl_2 could be reasoned by the dynamic etching lowering the coverage at a given time, whereas MeOH suppresses etching due to O-Cu interactions of the solvent. Thus, MeOH slows down the functionalization due to competition over binding sites but enables higher coverages than CH_2Cl_2 at long modification times (3 days in this case).

Noteworthy, XPS determines the coverage as an averaged coverage across the spot size with a diameter of 100 µm. To distinguish between monolayer formation across the measured area and

multilayer island formation on elsewhere naked metal, the surface roughness needs to be determined.

6 AFM Measurements

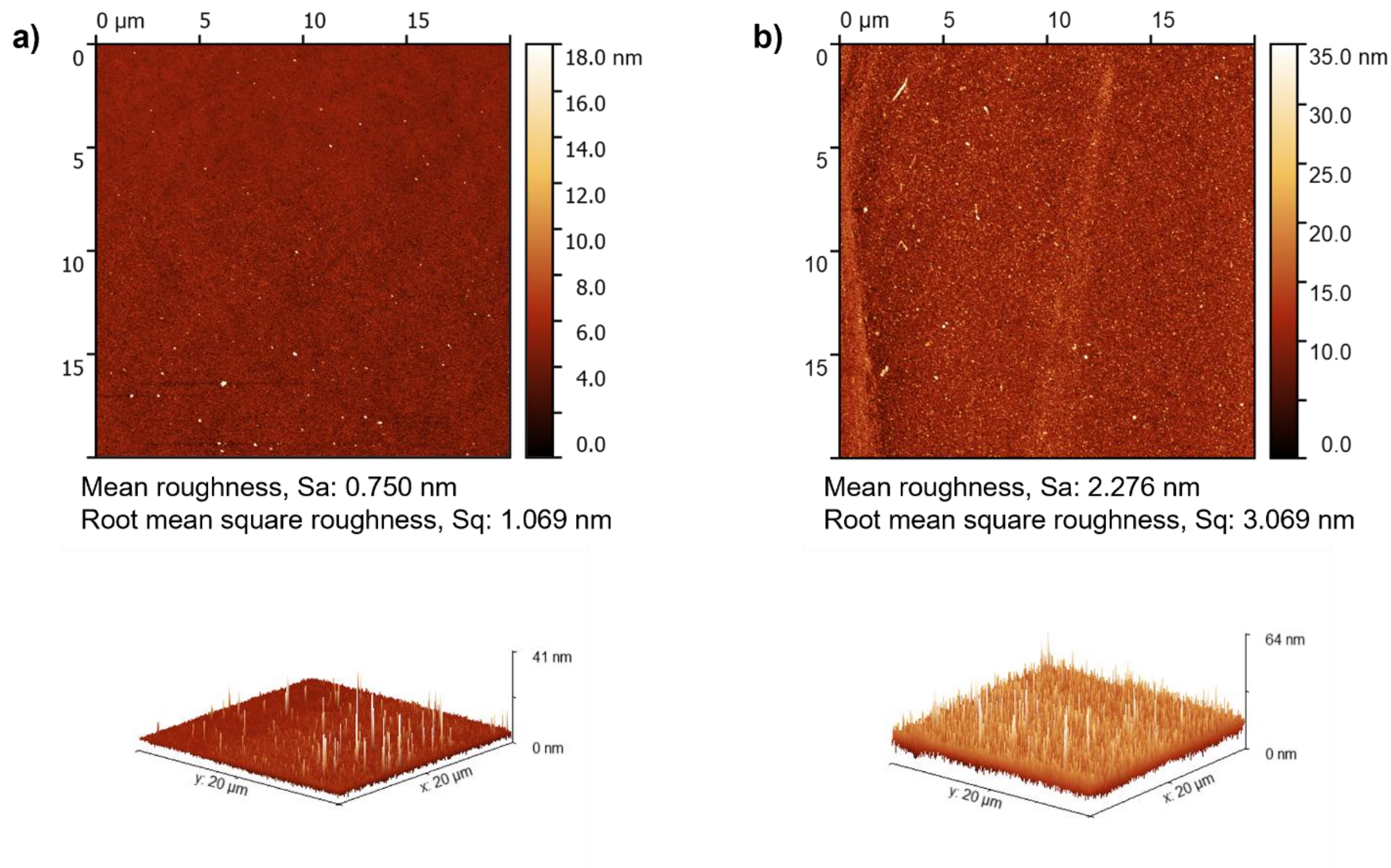

Figure S67: AFM images of a copper substrates placed in CH_2Cl_2 for one night, without (a) and with (b) 2 mg of BIMeH·HCO_3. Both were washed with MeOH. In both cases, height profiles are depicted as heap map (top) and as 3D map (bottom). Key AFM roughness parameters throughout the respective 3D map are noted.

Atomic force microscopy (AFM) allows precise images via a Bruker Icon atomic force microscope. The cantilevers which were used in this case were RTESPAW-300 (reflective coating, Oxford instruments, Abingdon, UK). The images were recorded in non-contact/tapping-mode and under ambient conditions. Data analysis of the recorded images was performed utilizing Gwyddion 2.66 (released 2024-05-24).

The roughness of the copper surfaces was determined by employing AFM measurements and analyzing the recorded images with the Gwyddion software. The roughness parameters, AFM images and the corresponding 3D-map can be found in Figure S67. The average roughness (Sa) describes the mean value of the sample roughness, while the root mean square (RMS) roughness (Sq) expresses the magnitude of change in roughness.[15]

7 WLI Measurements

White light interferometry (WLI) was performed with a VK-X3100 3D Surface profiler from Keyence. Measurements used a Mirau interference objective (10x Nikon CF IC EPI Plan DI, NA 0.30, working distance 7.4mm), giving a lateral resolution of 0.5 µm and a height resolution of 0.1 nm. Samples were aligned to normal incidence with a tilt stage before measurement by minimizing interference fringes.

The roughness of the copper surfaces was determined by employing WLI measurements and analyzing the recorded images with the Gwyddion 2.66 software.

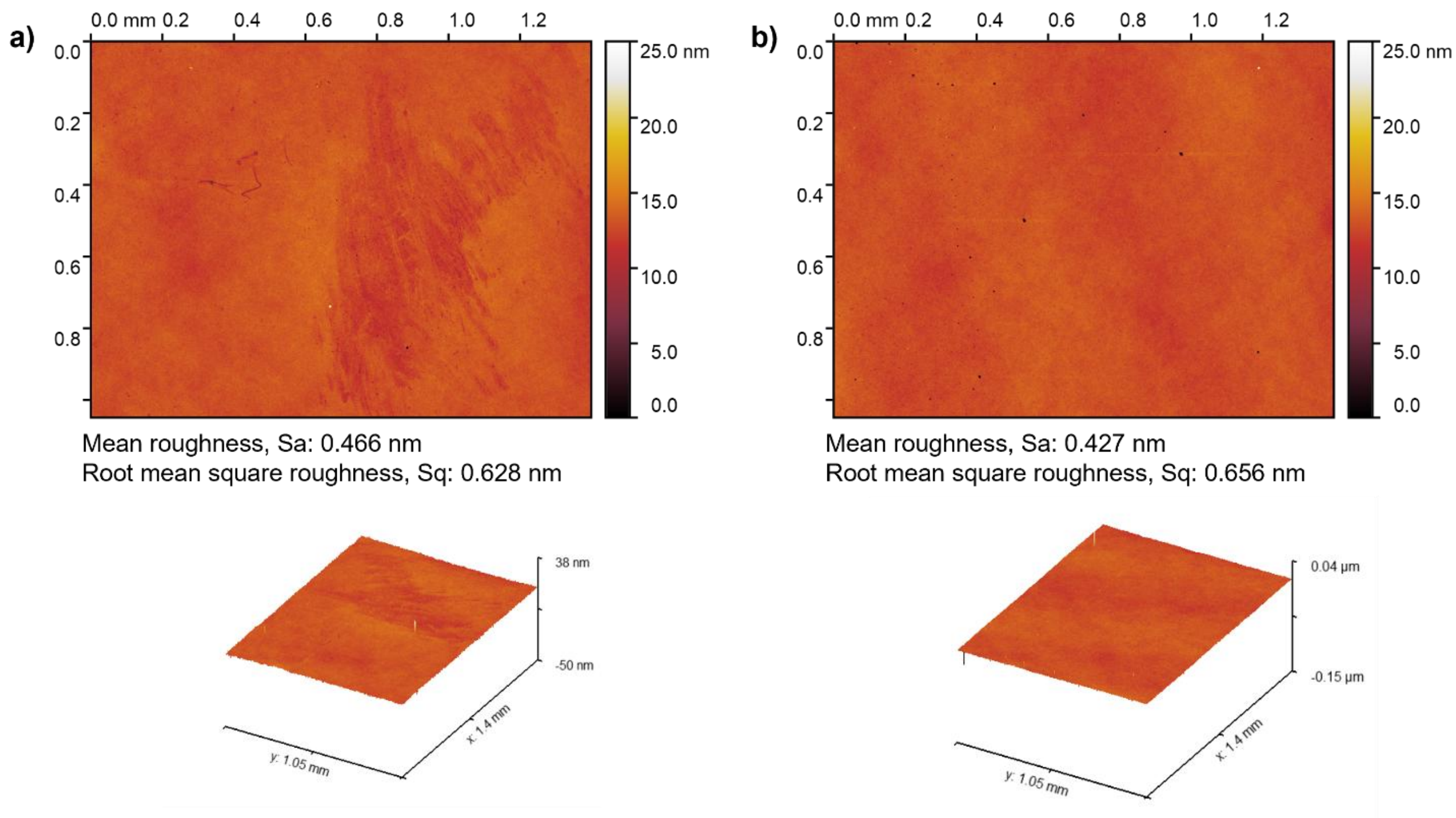

Figure S68: a) Washed but unfunctionalized blank copper surface. b) Copper surface after NHC functionalization according to the following procedure: PVD modification of Cu slide with IPr·CO_2 at 140 °C for 30 minutes. Washed with iPrOH. XPS spectra can be found in Figure S24.

Sa and Sq before modification (Figure S68a) are in good agreement with the results from AFM (Figure S67a) and indicate the surface area of the copper substrates to be very flat with Sq being around or even below 1 nm. Whilst our AFM measurements focus on an area of 15x15 μm^2, WLI shows low roughness for an area of 1x1 mm^2. After modification, both analysis methods still yield reasonable low roughness parameters and don't show any significant step-edges or other signs of organic island formation on the substrate. As we don't find elevated organic islands on the surface, the coverage observed in XPS is presumably evenly distributed throughout the surface giving reason to conclude monolayer formation.

Notably, Figure S67 b shows AFM of liquid phase modification of copper with BIMeH·HCO_3 in CH_2Cl_2, whereas Figure S68 b shows WLI of copper modified with IPr·CO_2 via PVD. This lowers comparability between both experiments in favor of generality. However, liquid phase modification with BIMeH·HCO_3 in CH_2Cl_2 (Sq = 3.1 nm) roughens the substrate more than vapor phase

modification with $IPrCO_2$ (Sq = 0.66 nm). This absence of roughening observed during PVD agrees with a former report of NHC smoothing metal surfaces during PVD as a consequence of microscopic restructuring.[16]

8 UV-Vis Measurements

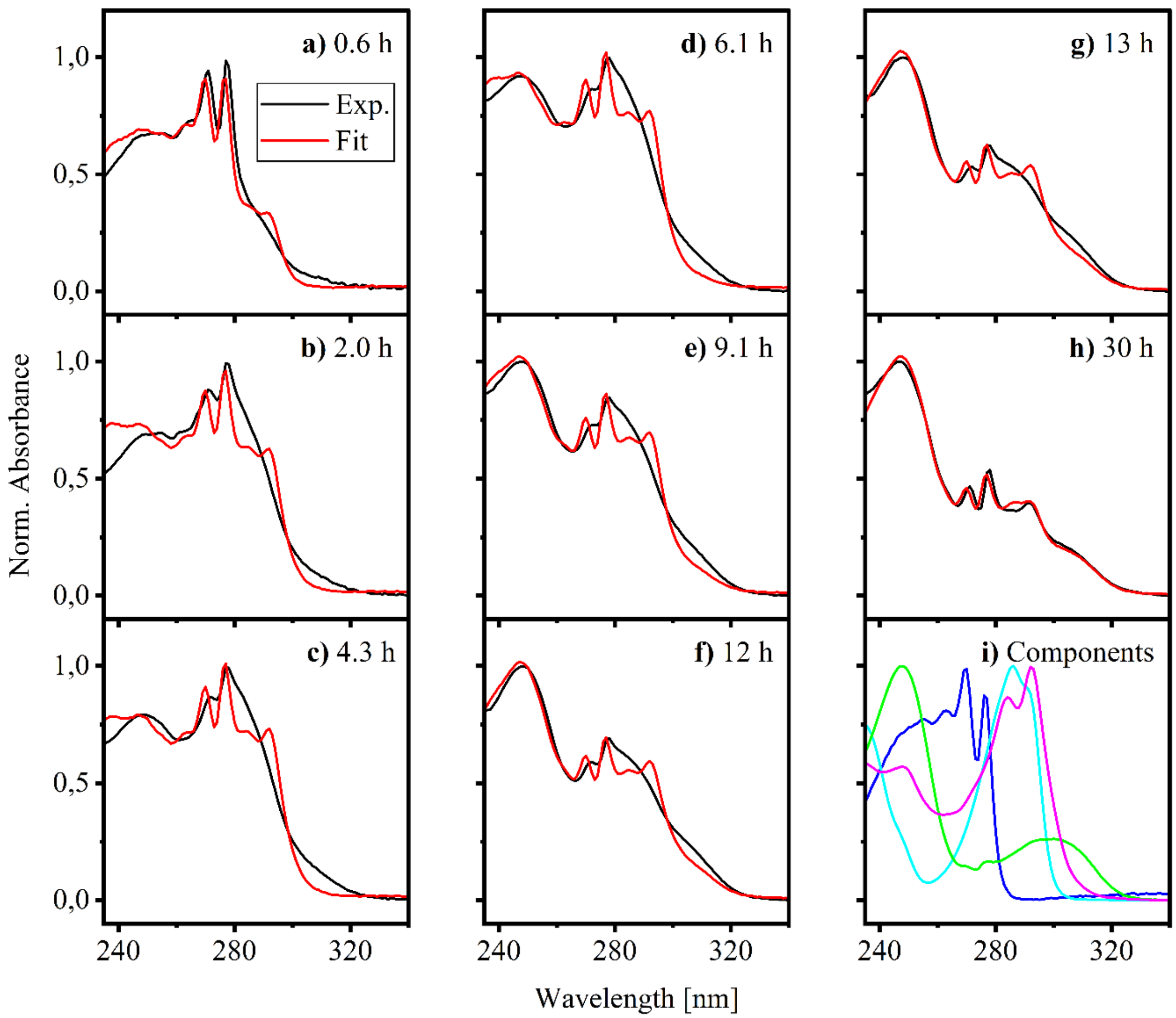

Figure S69: UV-Vis spectra of the etching solution after different time steps (black line, **a**-**h**) and their fits (red line, **a**-**h**) due to superposition of experimental component spectra (**i**). All intensities are normalized. Color code of **i**: $BIMeH{\cdot}HCO_3$ is blue, BIMeO is cyan, aldehyde **2** is green and $[Cu(BIMe)_2]PF_6$ is magenta.

First of all, five component UV-Vis spectra were measured as depicted in Figure S69 i. Also, 5 mg $BIMeH{\cdot}HCO_3$ and 5 ml CH_2Cl_2 were added to a custom build etching setup. The latter consists of a Teflon stage holding a copper substrate upside down at a height of 1 cm inside a glass vial. The mixture was stirred with a Teflon coated magnetic stirring bar at 750 rpm. At several timesteps, 100 µl samples were taken and diluted for at-line UV-Vis measurements (dilution factors of 10 for

early time steps up to 100 for the last time step). Linear superposition of the component spectra was applied to deconvolute the etching solution spectra.

The superposition of component spectra (Figure S69 i) as approach to fit the time dependent UV-Vis spectra (Figure S69 a-h) has one major drawback: Any interactions between individual components are neglected, despite knowing about BIMeO coordination to Cu-complexes based on XRD and despite the potential influence of intermolecular interactions on the absorption behavior of the mixture. This complicates the deconvolution of the time-dependent measurements especially between 275 nm and 295 nm and resembles the most significant deviation of experimental data and its fitted superposition. Secondly, excessive BIMeH·HCO_3 is present as partially dissolved solid in the reaction mixture and dissolves upon dilution of the sample taken for UV-Vis. Therefore, the amount of BIMeH·HCO_3 observed as a function of reaction time lacks information. However, all characteristic features (maxima, shoulders) of the spectra (Figure S69 a-h) of the reaction mixture can be retrieved in the component spectra (Figure S69 i). Also, the following trends are clear and in agreement with XRD, ESI and ICP-OES: A) BIMeO is formed in the very beginning of the etching, whilst native copper oxide gets dissolved by NHCs. B) $[Cu(BIMe)_2]^+$ is formed during the first few hours of etching and decomposes later on. C) BIMeO is also formed upon $[Cu(BIMe)_2]^+$ decomposition. D) The $Cu^{I/II}$ catalyzed hydration of BIMe forms a high amount of aldehyde **2** at an elongated reaction time dominating the 30 h spectrum (Figure S69 h).

Table S3: Amount of the four observed etching products in dependence of time as obtained from deconvolution of the UV-Vis spectra.

Time [h]	m(BIMeH·HCO_3) [µg]	m(BIMeO) [µg]	m(aldehyde **2**) [µg]	m($[Cu(BIMe)_2]PF_6$) [µg]
0.6	123.4	22.7	0.0	4.5
2.0	99.5	29.2	0.0	16.0
4.3	97.6	27.3	0.1	22.2
6.1	93.5	32.7	38.8	19.1
9.1	73.5	27.0	91.3	15.5
11.9	55.9	17.3	118.7	13.8
13.5	49.4	17.3	136.5	10.0
29.7	85.9	40.9	329.4	0.0

9 ICP-OES Measurements

Inductively coupled plasma optical emission spectroscopy (ICP-OES) was performed on an ArCos MVII ICP-OES (Spectro, Kleve, Germany), which was equipped with a crossflow spray chamber. For calibration, Cu solutions of 10 to 1000 µg/L were analyzed. At each studied emission line of Cu (219.226 nm, 219.958 nm, 224.700 nm, 324.754 nm and 327.396 nm) one calibration line was recorded. The plasma energy (RF-Power) amounted 1200 W. The argon cooling gas flow was 12 L/min, the auxiliary argon gas flow was 0.8 L/min and the nebulizer argon gas flow was 0.8 L/min. All samples were acidified with 2 % (vol.) HNO_3.

Time dependent samples of an etching solution were prepared according to Table S4. Knowing the taken sample volume, the Cu concentration c(Cu) in the etching solution was deducted. Multiplied by the left etching volume at a given time, this gave the amount of Cu inside dissolved in the etching solution Cu_{left} as function of time. Finally, the amounts of Cu removed from the etching solution Cu_{taken} were accumulated to Cu_{left} to obtain the total amount of etched Cu at a given time Cu_{etched}. Both experimental series show a qualitatively similar Cu-t trend. However, the

sample in the first series (Table S4) was larger than the second one (Table S5). As the substrate weight is directly proportional to the surface size and therefore the available amount of Cu, dividing Cu_{etched} by the substrate weight, gives quantitative similar trends. For visualization, both experimental series were averaged and plotted in Figure 6 a.

Table S4: Thin layer Cu substrate (m = 282.2 mg) and 5 mg BIMeH·HCO_3 in 2 ml MeCN. At the given timesteps volumes of 0.2 ml were taken and used for Cu quantification via ICP-OES.

Time [min]	Cu_{taken} [µg]	V_{taken} [ml]	V_{left} [ml]	c(Cu) [µg/ml]	Cu_{left} [µg]	Cu_{etched} [µg]
10	0.017	0.20	4.00	0.09	0.34	0.34
30	0.131	0.20	3.80	0.65	2.48	2.50
60	0.327	0.20	3.60	1.64	5.89	6.04
240	1.904	0.20	3.40	9.52	32.37	32.85
330	3.148	0.20	3.20	15.74	50.37	52.75
1170	3.425	0.20	3.00	17.13	51.38	56.91
1515	3.453	0.20	2.80	17.27	48.35	57.30
1600	3.432	0.20	2.60	17.16	44.61	57.02

Table S5: Thin layer Cu substrate (m = 229.3 mg) and 5 mg BIMeH·HCO_3 in 2 ml MeCN. At the given timesteps volumes of 0.2 ml were taken and used for Cu quantification via ICP-OES.

Time [min]	Cu_{taken} [µg]	V_{taken} [ml]	V_{left} [ml]	c(Cu) [µg/ml]	Cu_{left} [µg]	Cu_{etched} [µg]
10	0.000	0.20	4.00	0.00	0.00	0.00
30	0.037	0.20	3.80	0.19	0.70	0.70
60	0.181	0.20	3.60	0.91	3.27	3.30
240	0.768	0.20	3.40	3.84	13.05	13.27
330	1.197	0.20	3.20	5.99	19.15	20.14
1170	2.412	0.20	3.00	12.06	36.18	38.36
1515	2.965	0.20	2.80	14.83	41.51	46.11
1600	3.092	0.20	2.60	15.46	40.20	47.76

Table S6: Averaged kinetic series described in Table S4 and S5.

Time [min]	Cu_{etched} [µg]	SD(Cu_{etched}) [µg]
0	0.00	-
10	0.17	0.49
30	1.60	1.13
60	4.67	1.39
240	23.06	3.72
330	36.45	4.80
1170	47.63	3.62
1515	51.70	2.81
1600	52.39	2.56

10 CV Experiments and Redox Active Additives

Using electrodeposition, NHCs are known to form monolayers on Au and other metals at negative potentials (-1.0 V).[17] Here, we use cyclic voltammetry (CV) to investigate, whether the applied voltage influences the N 1s peak ratio and intensity. Ending with a negative semi-cycle (see Figure S32), two prominent peaks were observed (400.7 and 398.8 eV) with a peak ratio of 2.7. Ending with a positive semi-cycle (see Figure S70), the peak ratio stays almost unchanged at 2.9. However, the integration area of both peaks as well as the signal to noise ratio of the N 1s spectrum decreases. Thus, a negative voltage favors Cu functionalization and a positive voltage favors de-functionalization or etching. A voltage induced interconversion between both N 1s peaks was not observed.

Further, additions of several oxidants or reductants were tested during modification and post-modification. Cu was modified with IMe·CO_2 as NHC precursor analogue to the procedure and data ascribed in Figure S56. During this series of measurements, also reference samples without additives were measured (see Figure S71 and Figure S72). Already within these reference measurements, peak ratios between 1:3 and 3:1 were observed. Also it should be noted, that IMe was generally observed at slightly higher BEs than BIMe.[3,18]

Adding benzoquinone or acetone oxime during modification seems to suppress the N 1s signal at lower BEs yielding one dominant peak at around 401.5 eV (peak ratio 9:1, see Figure S73-S75).

A treatment of the functionalized Cu surface with acetone oxime as oxidizing agent did not result in any clear change of peak ratio or intensity (see Figure S76).

Looking again at BIMe on Cu, liquid phase modification followed by reducing treatments were investigated (Figure S77-S80). Treatments of the functionalized surface with H_2 gas was not found to clearly influence the peak ratio or intensity. A treatment with $NaBH_4$ was found to favor the lower of both N 1s peaks (399.3 eV) but could not suppress either peak.

In summary, both oxidizing or reducing treatments of functionalized surfaces did not yield any clear evidence of interconversion of removal of either surface bound species observed in XPS at 398.8 or 400.7 eV, respectively.

The addition of oxidizing agents like benzoquinone or acetone oxime clearly suppressed the N 1s peak at lower BEs in favor of a peak at 401.5 eV. This finding leaves room for interpretation. Either the bis(NHC) adatom complex got oxidized and therefore removed from equilibrium between both binding modes resulting in a dominant upright binding mode observed at a N 1s BE of 401.5 eV. Or the oxidizing agent and the NHC form an adduct which chemisorbs on the surface. This is rather improbable due to the similarity between the results obtained with two chemically quite different oxidizing agents. Also, no adducts between NHC and oxidizing agents have been found upon liquid phase analysis via ESI.

Peaks					
Name	Peak BE	FWHM eV	Area (P) CPS.eV	Atomic %	Q
N1s	400.77	2.42	3365.42	74.24	Yes
N1s Scan A	399.04	2.14	1169.36	25.76	Yes

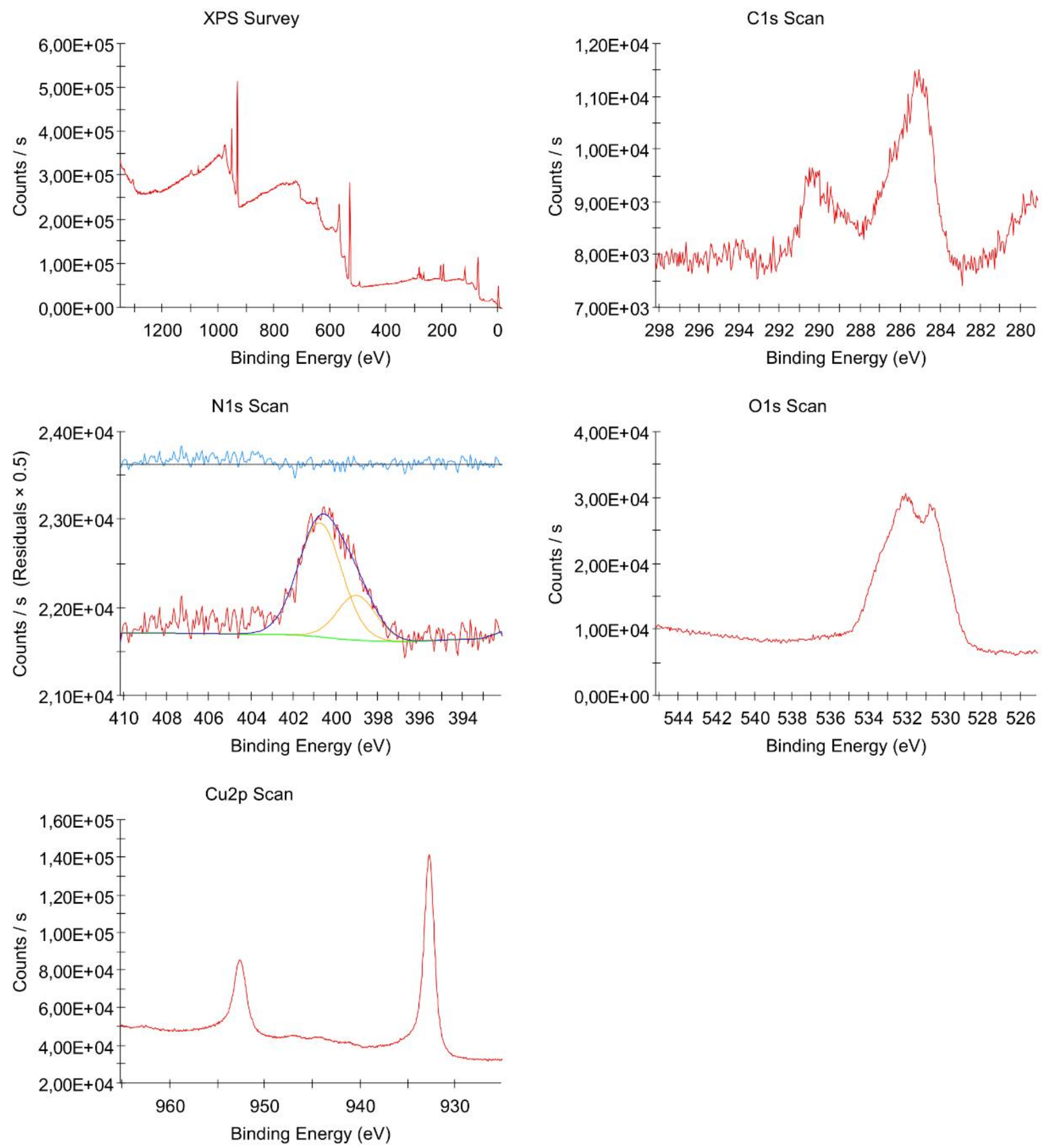

05.03.2026 17:57:31 Data Grid 3

Figure S70: Survey and element spectra accompanying the N 1s XPS spectrum displayed in Figure 2 l. Sample preparation details: Cu foil (100 µm thick) in 10 ml MeOH (20 mM of $LiClO_4$ and 1 mM BIMeH·HCO_3) under cyclic voltammetry conditions (0.1 V/s, +1 V / -1.2, 10 cycles, starting with positive semi-cycle and finishing with positive semi-cycle). Washed with H_2O, MeOH and CH_2Cl_2.

Peaks					
**Name**	**Peak BE**	**FWHM eV**	**Area (P) CPS.eV**	**Atomic %**	**Q**
N1s	401.21	2.13	19602.70	72.34	Yes
N1s Scan A	398.61	2.13	7510.20	27.66	Yes

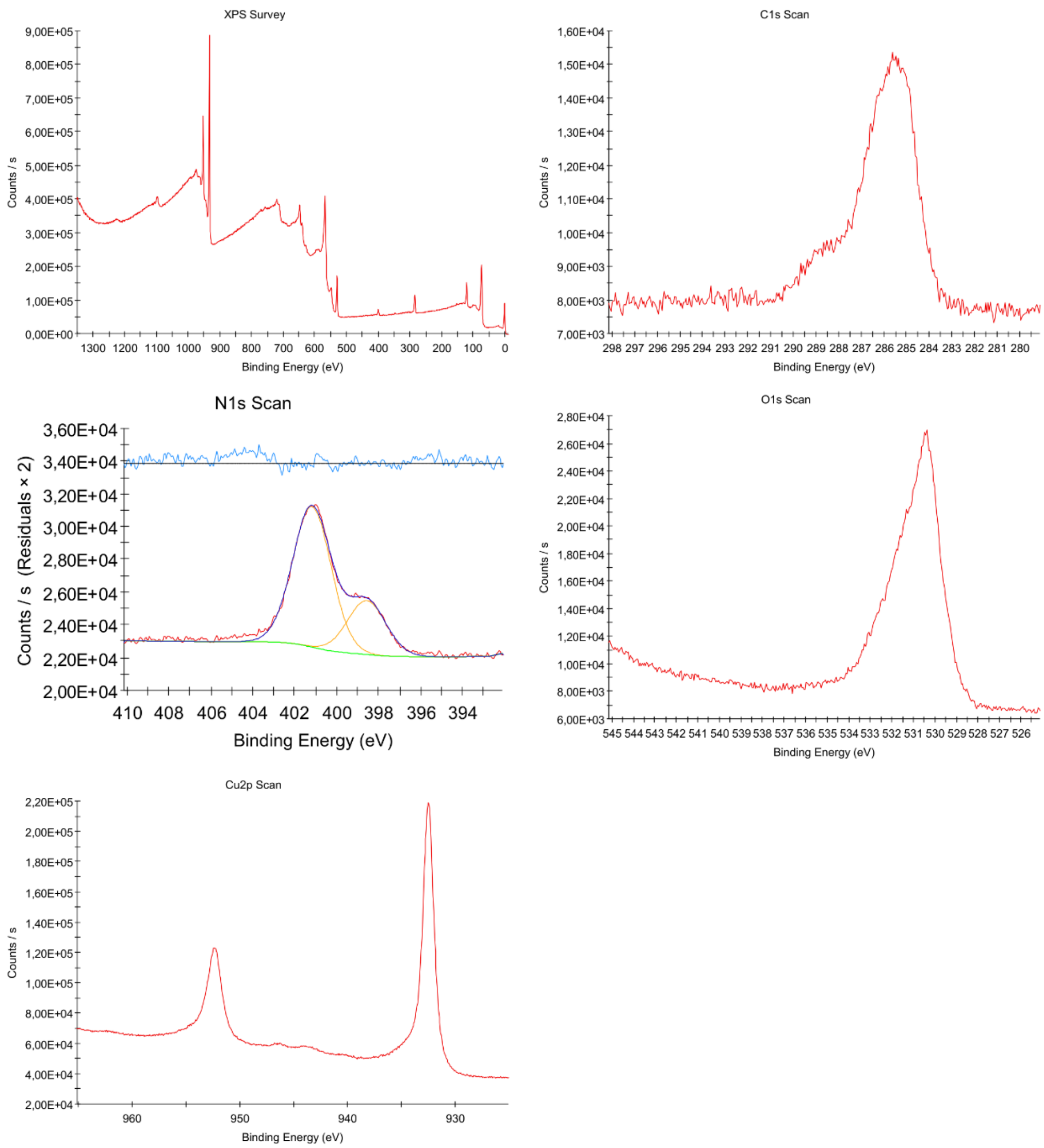

05.03.2026 17:40:27 O:\ANP\GLO_1111_ANP-AC-051\ANP-AC-051 02 A.vgp

Figure S71: Survey and element XPS spectra of a Cu substrate modified with IMe. Sample preparation details: Cu slide and 5 mg IMe·CO_2 in 1 ml CH_2Cl_2 at 60 °C for 30 minutes. Washed with iPrOH.

Peaks					
Name	Peak BE	FWHM eV	Area (P) CPS.eV	Atomic %	Q
N1s Scan A	399.20	2.07	17329.68	69.36	Yes
N1s Scan B	401.71	2.07	7642.75	30.64	Yes

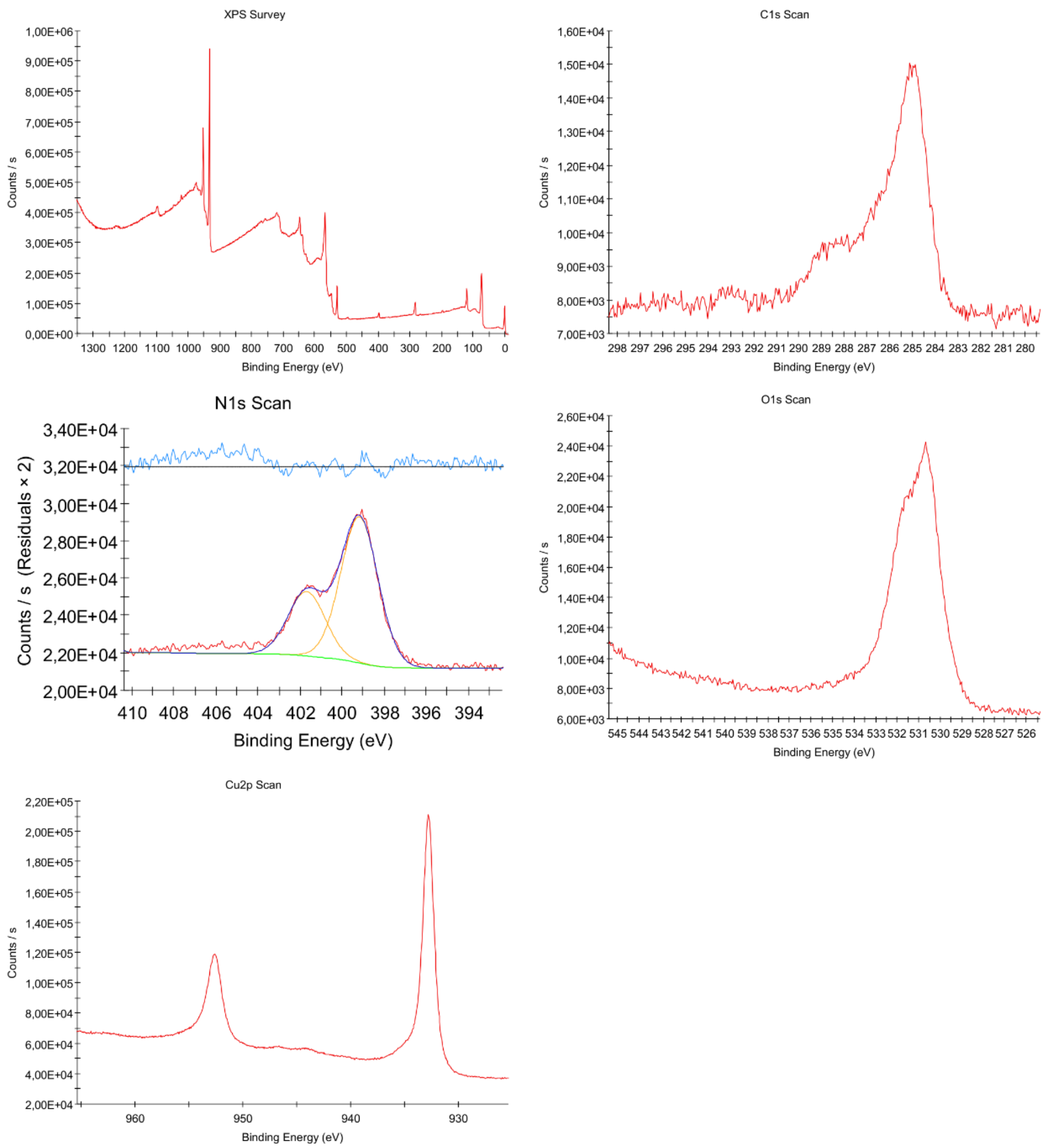

05.03.2026 17:52:45 O:\ANP\GLO_1114_ANP-AC-054 and SHE-SB-102\ANP-AC-054 01A.vgp

Figure S72: Survey and element XPS spectra of a Cu substrate modified with IMe. Sample preparation details: Cu slide and 5 mg IMe·CO_2 in 1 ml CH_2Cl_2 at 60 °C for 30 minutes. Washed with iPrOH.

Peaks					
Name	Peak BE	FWHM eV	Area (P) CPS.eV	Atomic %	Q
N1s Scan A	399.53	1.75	2938.28	10.51	Yes
N1s Scan B	401.83	1.75	24974.31	89.49	Yes

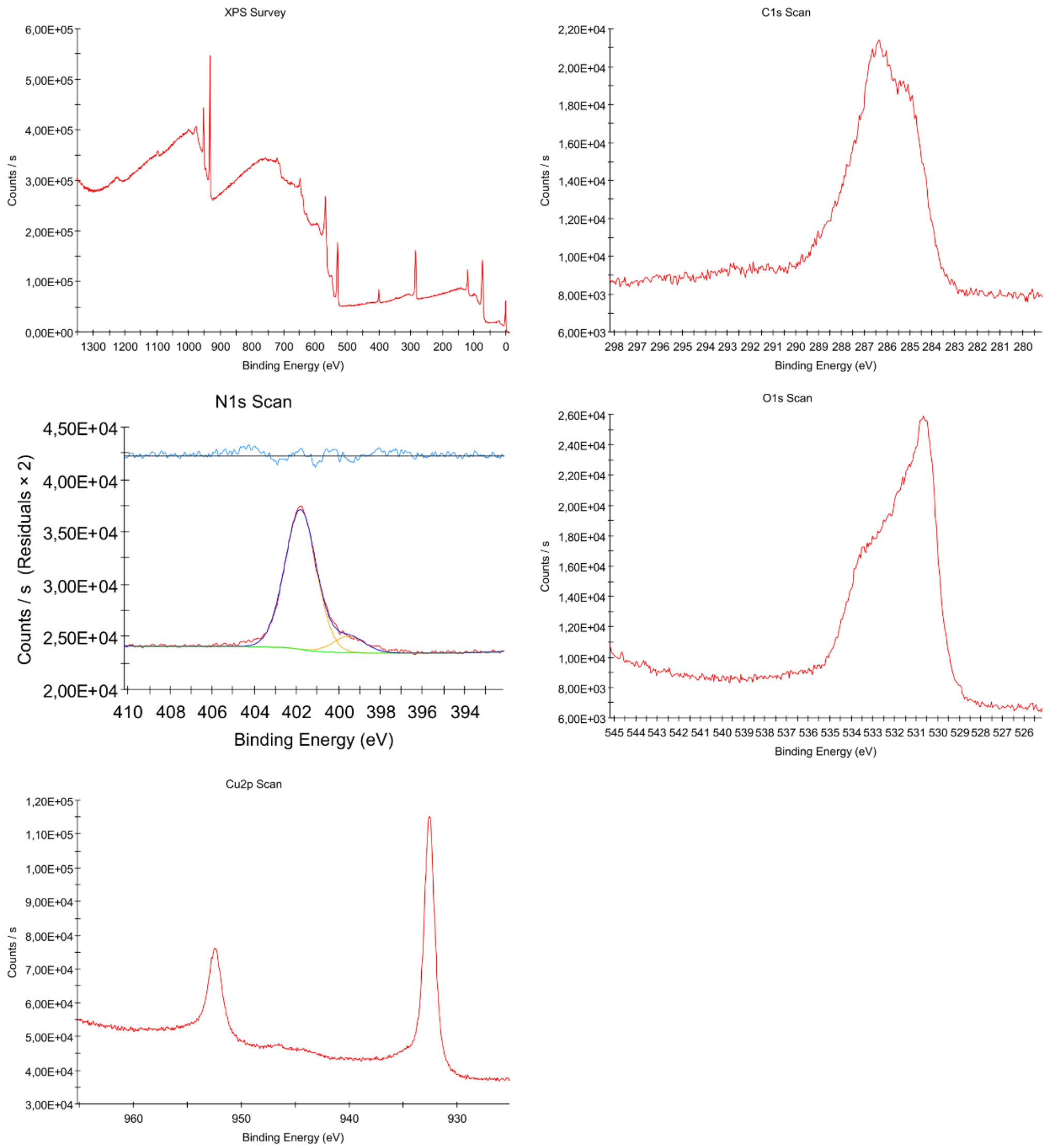

Figure S73: Survey and element XPS spectra of a Cu substrate modified with IMe in presence of benzoquinone. Sample preparation details: Cu slide, 5 mg IMe·CO_2 and 5 mg of benzoquinone in 1 ml CH_2Cl_2 at 60 °C for 30 minutes. Washed with iPrOH.

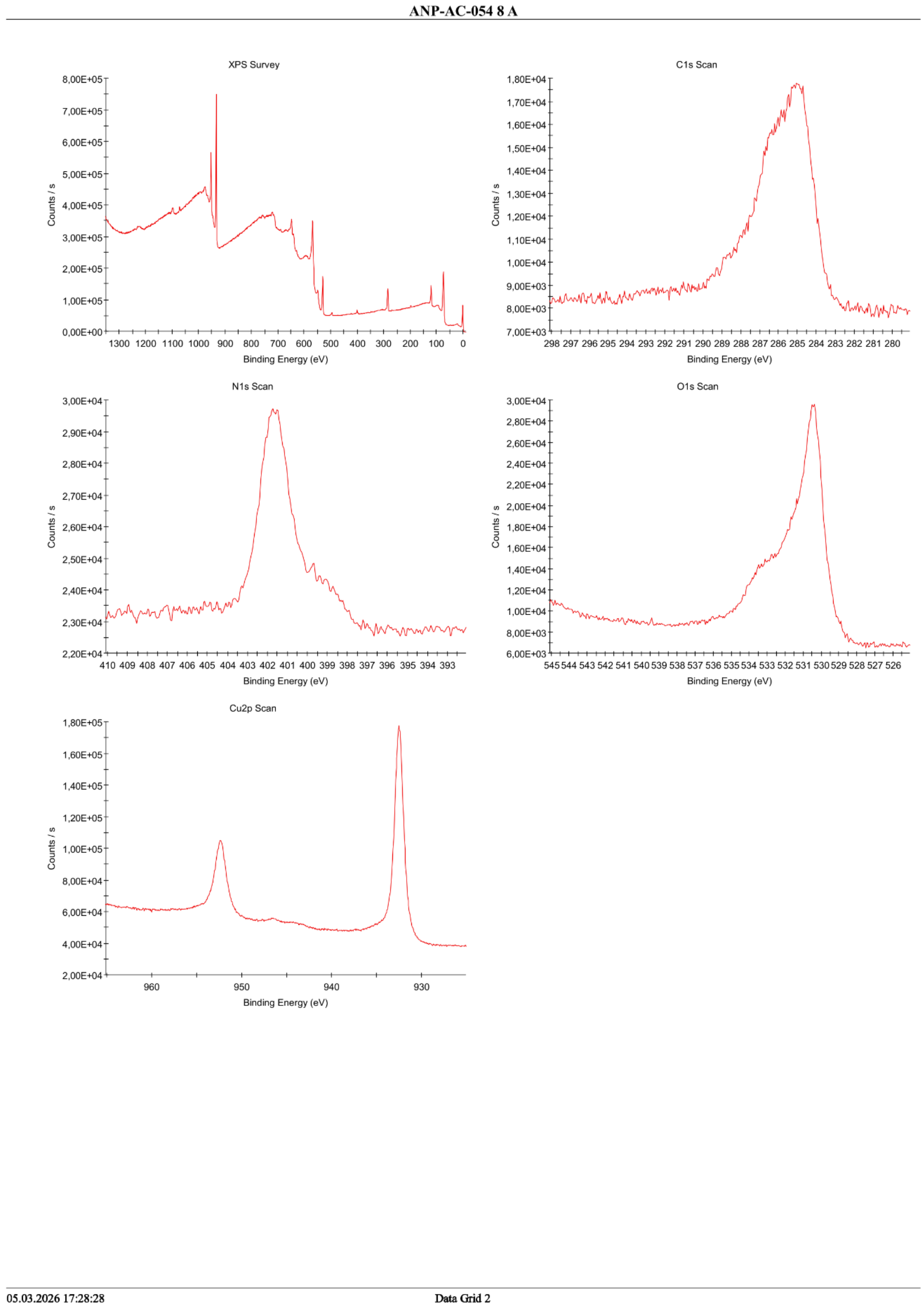

Figure S74: Survey and element XPS spectra of a Cu substrate modified with IMe in presence of benzoquinone. Replicate of the sample presented in Figure S70. Preparation details: Cu slide, 5 mg IMe·CO_2 and 5 mg of benzoquinone in 1 ml CH_2Cl_2 at 60 °C for 30 minutes. Washed with iPrOH.

Peaks					
Name	Peak BE	FWHM eV	Area (P) CPS.eV	Atomic %	Q
N1s	401.50	1.59	29562.86	91.66	Yes
N1s Scan A	399.30	1.59	2693.45	8.34	Yes

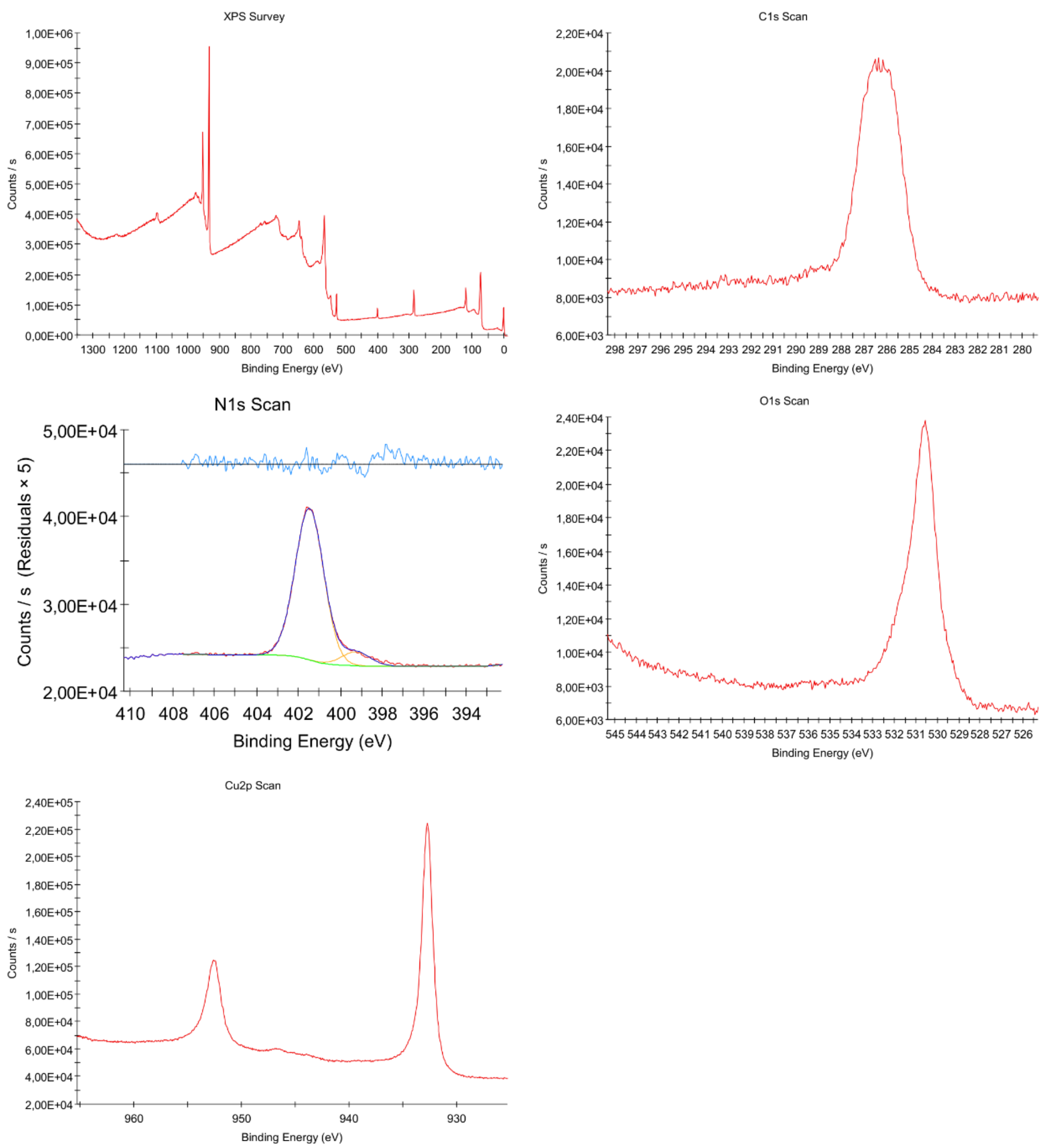

05.03.2026 17:48:49 O:\ANP\GLO_1105_ANP-AC-049\ANP-AC-049 2.vgp

Figure S75: Survey and element XPS spectra of a Cu substrate modified with IMe in presence of acetone oxime. Sample preparation details: Cu slide, 5 mg IMe·CO_2 and 2 mg of acetone oxime in 1 ml CH_2Cl_2 at 60 °C for 30 minutes. Washed with iPrOH.

Peaks					
Name	Peak BE	FWHM eV	Area (P) CPS.eV	Atomic %	Q
N1s Scan A	401.54	2.27	21395.62	77.80	Yes
N1s Scan B	399.02	2.27	6114.90	22.20	Yes

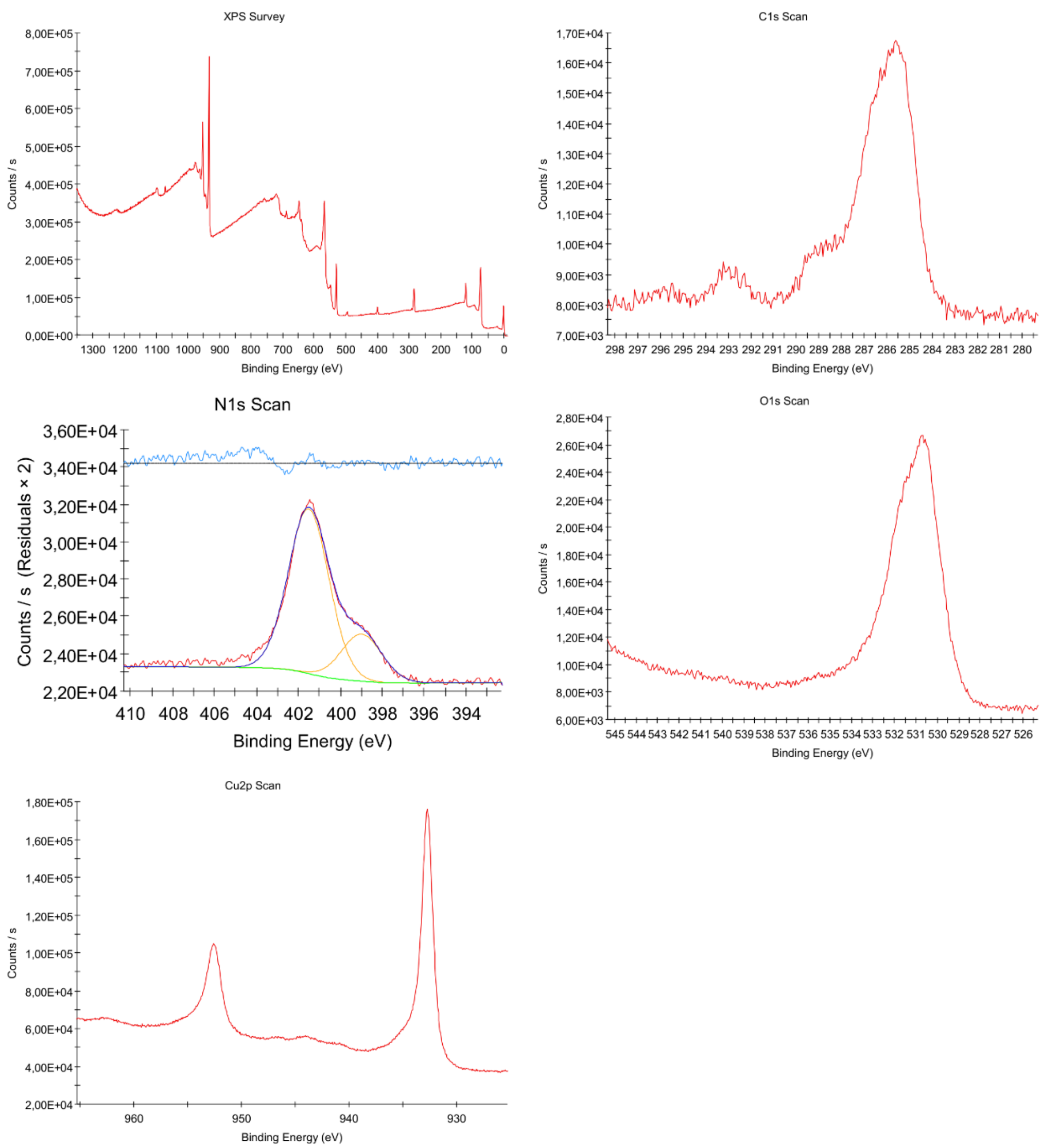

05.03.2026 18:03:18 O:\ANP\GLO_1112_ANP-AC-052\ANP-AC-052_09B.vgp

Figure S76: Survey and element XPS spectra of a Cu substrate modified with IMe and post-treated with acetone oxime. Sample preparation details: Cu slide and 5 mg IMe·CO_2 in 1 ml CH_2Cl_2 at 60 °C for 30 minutes. Washed with iPrOH. Treated with acetone oxime (2 mg in 1 ml CH_2Cl_2, 60 °C, 15 min) and washed with CH_2Cl_2 and iPrOH.

Peaks					
Name	Peak BE	FWHM eV	Area (P) CPS.eV	Atomic %	Q
N1s	401.09	2.02	15362.10	75.23	Yes
N1s Scan A	399.19	2.02	5063.84	24.77	Yes

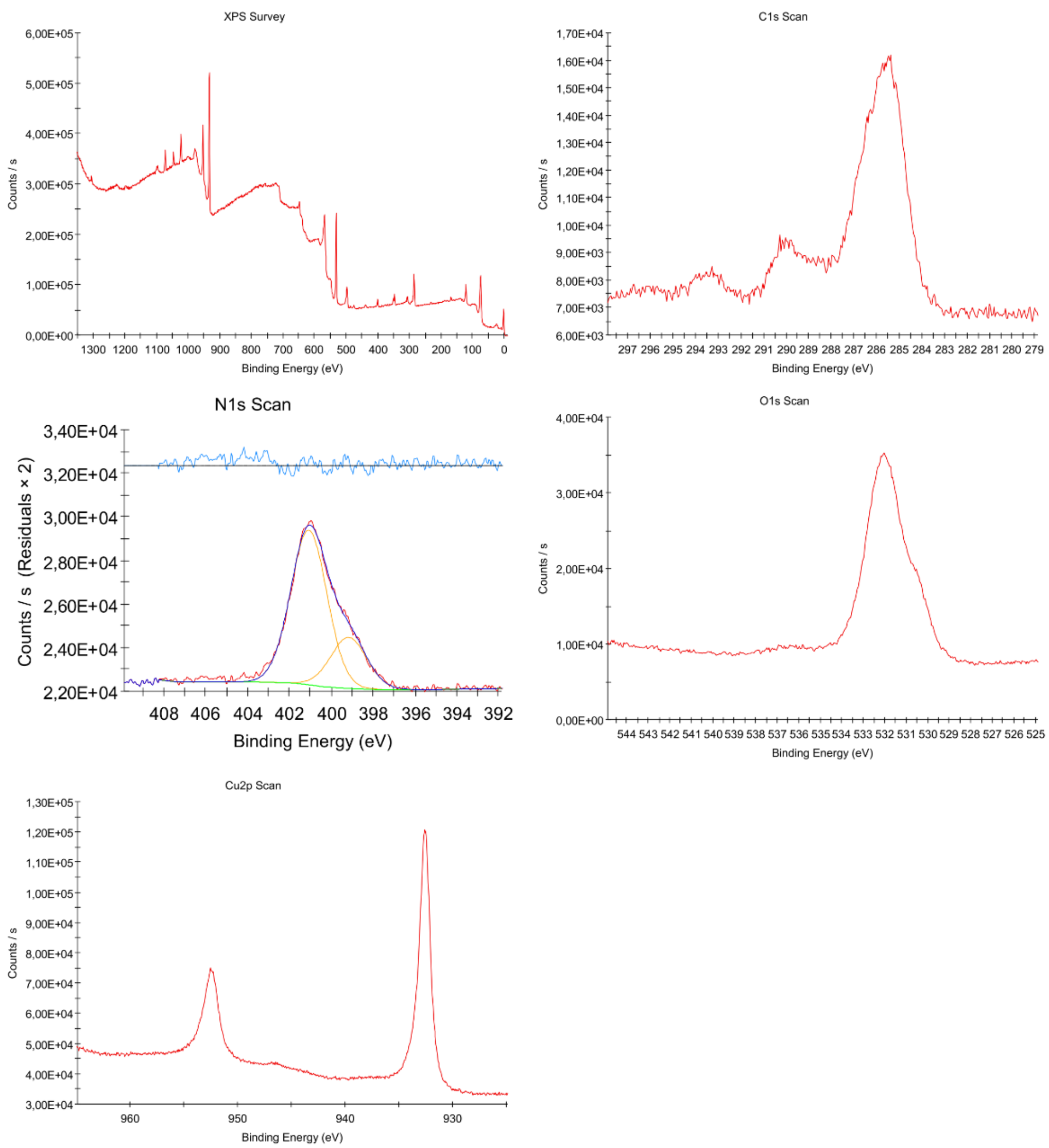

16.03.2026 18:47:11 O:\ANP\GLO_1021_ANP-AB-133\ANP-AB-133_01.vgp

Figure S77: Survey and element XPS spectra of a Cu substrate modified with BIMe. Sample preparation details: Cu slide and 12.5 mg BIMeH·HCO_3 in 1 ml MeCN and 1 ml MeOH at rt. for 3 hours. Washed with iPrOH.

Peaks					
Name	Peak BE	FWHM eV	Area (P) CPS.eV	Atomic %	Q
N1s	400.69	2.02	9695.79	60.68	Yes
N1s Scan A	399.10	2.02	6290.39	39.32	Yes

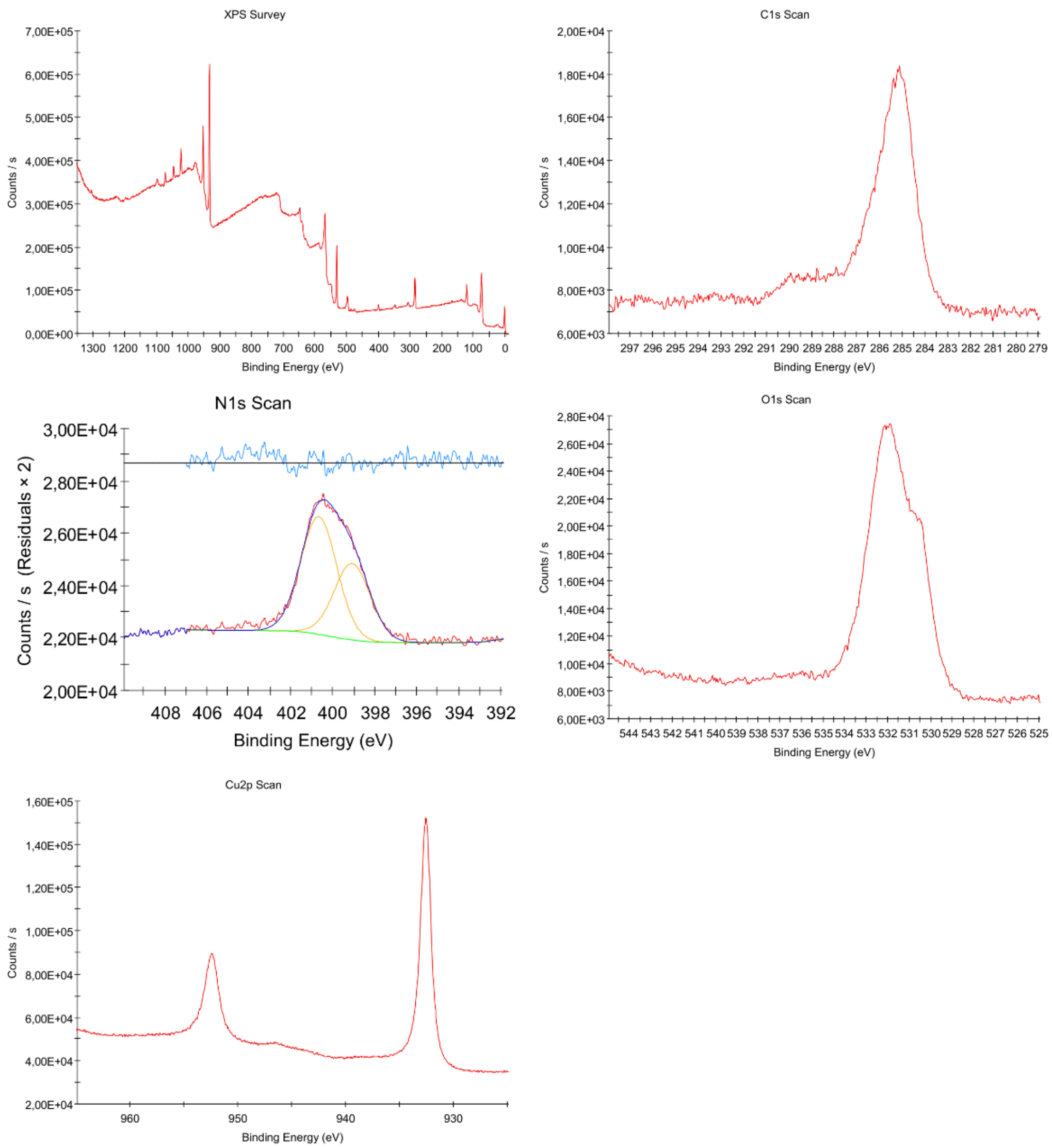

16.03.2026 18:36:17 O:\ANP\GLO_1021_ANP-AB-133\ANP-AB-133_04.vgp

Figure S78: Survey and element XPS spectra of a Cu substrate modified with BIMe. Sample preparation details: Cu slide and 12.5 mg BIMeH·HCO_3 in 1 ml MeCN and 1 ml MeOH at rt. for 3 h. Washed with iPrOH. Placed in 1 ml EtOH under 25 bar of H_2 gas for 16 h.

Peaks					
Name	Peak BE	FWHM eV	Area (P) CPS.eV	Atomic %	Q
N1s	400.86	1.94	11829.55	70.87	Yes
N1s Scan A	399.08	1.94	4867.71	29.13	Yes

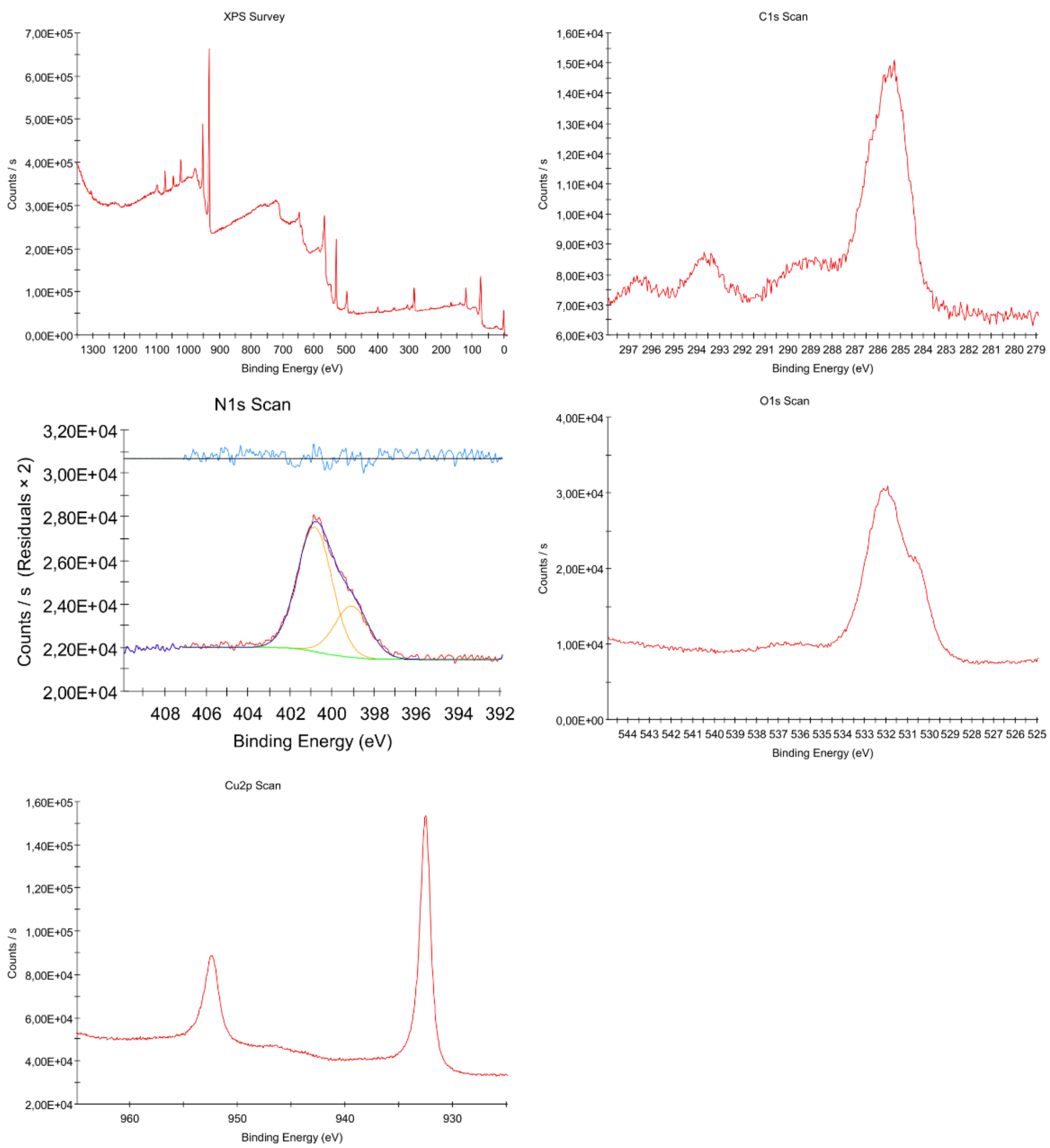

16.03.2026 18:34:32 O:\ANP\GLO_1021_ANP-AB-133\ANP-AB-133_05.vgp

Figure S79: Survey and element XPS spectra of a Cu substrate modified with BIMe. Sample preparation details: Cu slide and 12.5 mg BIMeH·HCO_3 in 1 ml MeCN and 1 ml MeOH at rt. for 3 h. Washed with iPrOH. Placed without solvent under 25 bar of H_2 gas for 16 h.

Peaks					
Name	Peak BE	FWHM eV	Area (P) CPS.eV	Atomic %	Q
N1s	401.16	1.96	3477.69	22.47	Yes
N1s Scan A	399.33	1.96	12016.57	77.53	Yes

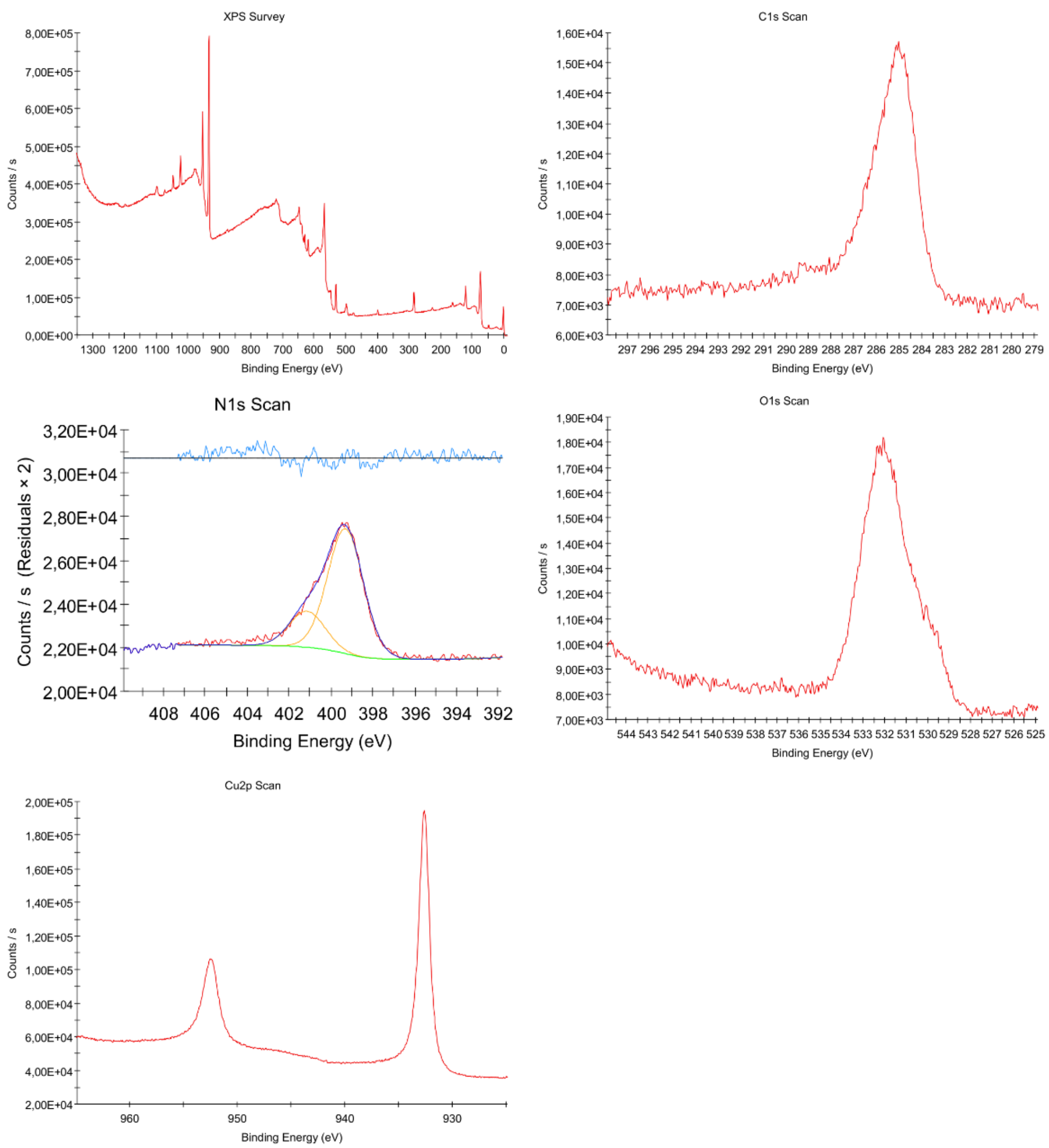

16.03.2026 18:33:48 O:\ANP\GLO_1021_ANP-AB-133\ANP-AB-133_08.vgp

Figure S80: Survey and element XPS spectra of a Cu substrate modified with BIMe. Sample preparation details: Cu slide and 12.5 mg BIMeH·HCO_3 in 1 ml MeCN and 1 ml MeOH at rt. for 3 h. Washed with iPrOH. Placed in 1 ml MeOH with 2 mg $NaBH_4$ for 5 minutes. Washed again with iPrOH.

11 EPR Data

Via electron paramagnetic resonance spectroscopy (EPR) it was found, that the Cu^{I} complexes originate from etching metallic copper, which degrade towards Cu^{II} ions.

All *continous wave* (cw) *electron paramagnetic resonance* (EPR) experiments were performed using a Bruker EMXNano X-band spectrometer (9.63 GHz). *Variable temperature* (VT) experiments at 90 K were performed using the integrated VT controller and a liquid nitrogen evaporator with integrated electrical heating.

Samples were prepared as solutions in CH_2Cl_2 and filled into 4 mm o.d. fused quartz tubes. Spectra were acquired the day of sample preparation as well as 12 days later. Acquisition parameters were optimized for each sample. Sweep rates of 2 to 4 mT/s with microwave power levels between 0.1 and 1.0 mW were used. Modulation amplitudes ranged from 0.2 to 0.4 mT at 100.0 kHz modulation frequency.

Spectra were exported with EasySpin-6.0.6[19] and plotted using Matplotlib[20].

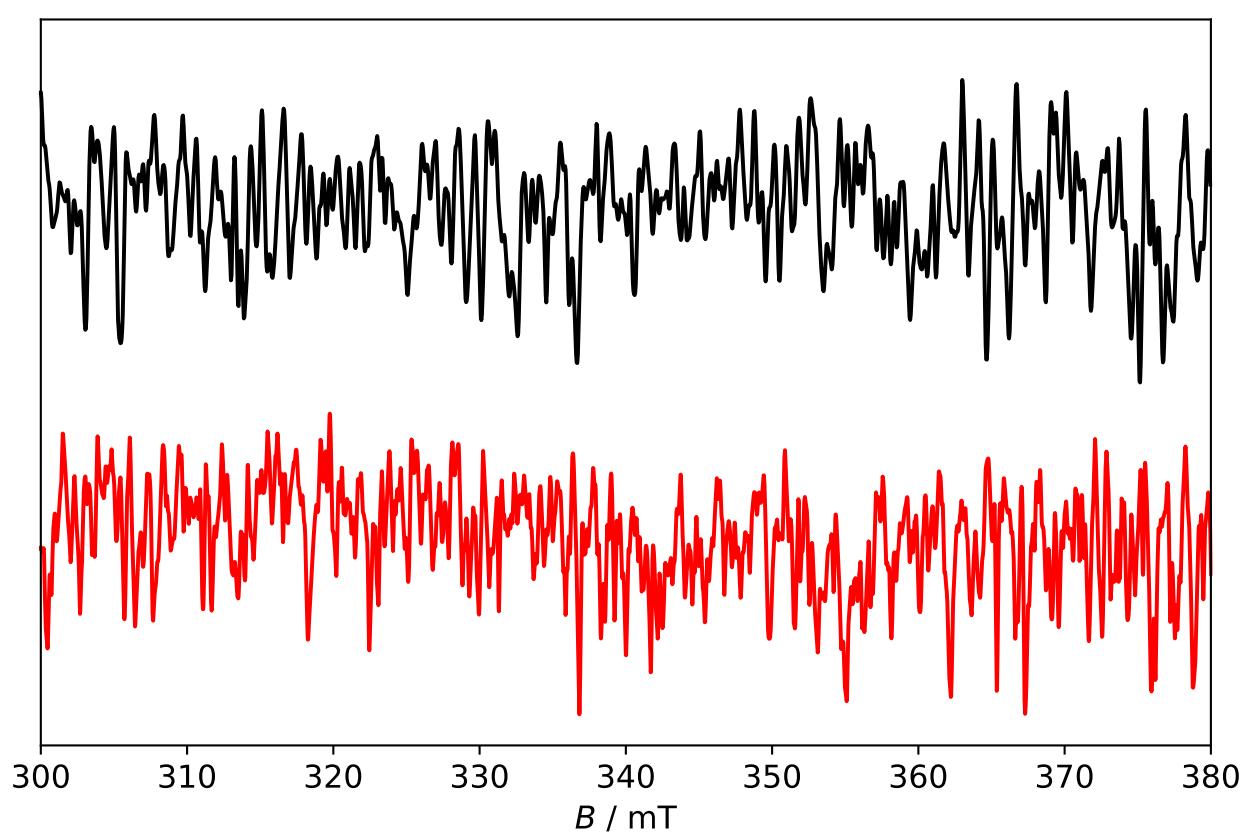

Figure S81: X-band cwEPR spectra of the Cu-NHC complex in CH_2Cl_2 measured at 90 K; measured the day of preparation (black) and after 12 days of being stored in the EPR tube under ambient conditions (red).

For the cwEPR spectra of the Cu-NHC complex (0.8 mg in 1 ml CH_2Cl_2, Figure S81), no signal could be observed. Neither directly after preparation of the sample, nor after 12 days of storage. This result is compatible with only Cu^{I} being present in the sample, since the d^{10} electron configuration results in a total spin of $S = 0$ and therefore no measurable signal.

Spectra acquired for the etching solution (5 mg BIMeH·HCO_3, 1 ml CH_2Cl_2, 0.5x0.5 cm^2 thin layer Cu substrate, two days of incubation at ambient conditions) however show a strongly broadened signal (Figure S82). This hints at the presence of Cu(II) species in the sample, since contrary to Cu^{I}, the d^9 configuration results in a total spin of $S = 1/2$. After 12 days, the spectrum still shows a residual signal. While the signal to noise ratio (S/N) is considerably lower than that of the initial spectrum, a change in lineshape can still be discerned. One possible explanation for this is the presence of multiple species in the initial solution. Since the sample tubes were not flame sealed, ingress of O_2 during the storage period is possible. This might lead to preferential decomposition of some of the species present, leading to the observed change in lineshape. The loss of signal may also be attributed so some precipitation of Cu^{II} complexes as inorganic Cu^{II} bicarbonate.

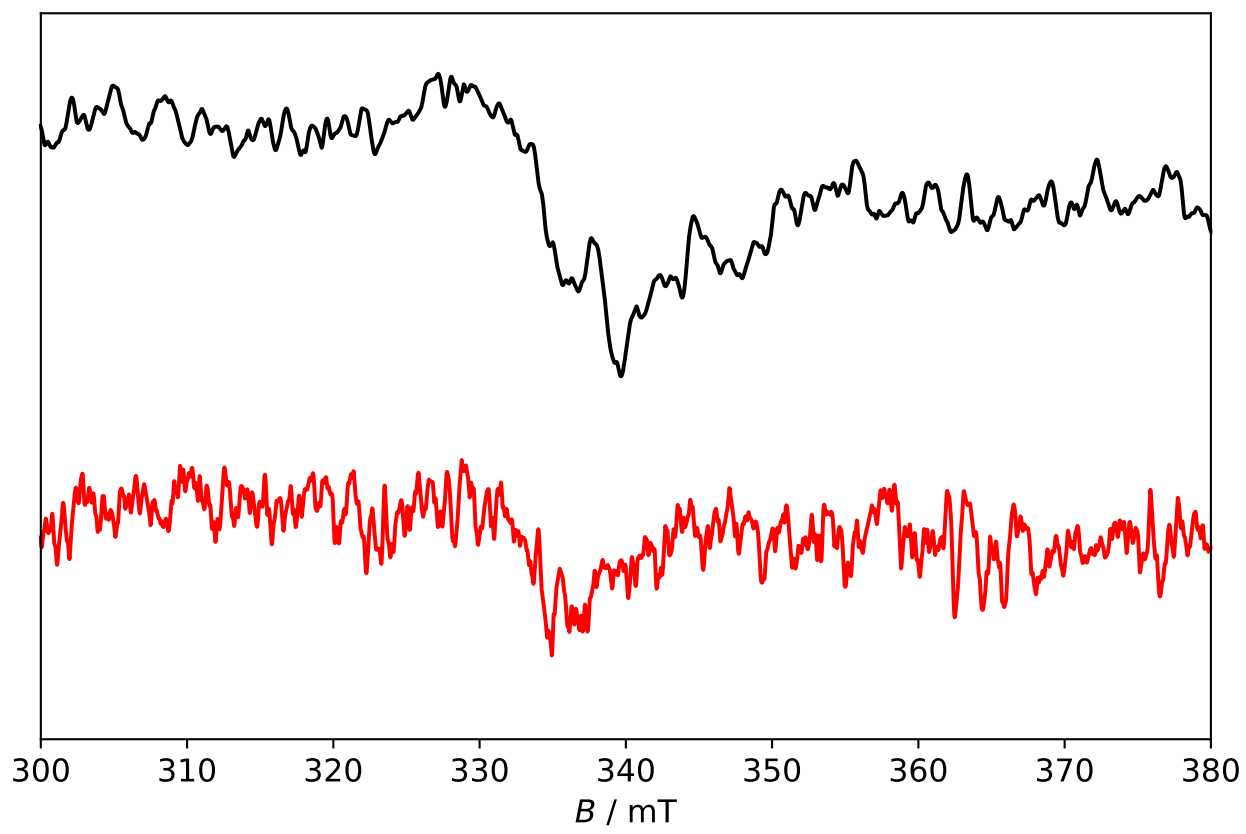

Figure S82: X-band cwEPR spectra of the etching solution measured at 90 K; measured the day of preparation (black) and after 12 days of being stored in the EPR tube under ambient conditions (red).

12 Control Experiments for Copper Etching

Several control experiments were conducted to connect the phenomenon of copper etching to the presence of active NHC precursors inside the etching solution.

12.1 Organic solvents as etching reagent

First, copper was placed in different organic solvents (CH_2Cl_2, MeCN, MeOH) for two weeks and no sign of etching was observed (substrates have not been dissolved within the studied time and no Cu complexes were found in solution by ESI).

It should be noted, that there was no aldehyde **2** or BIMeO observed via ESI upon storing $BIMeH_2CO_3$ in any organic solvent. Also, no $[Cu(BIMe)_2]^+$, aldehyde **2** or BIMeO was observed upon Cu modification with $BIMeH_2CO_3$ in alcohols.

12.2 Alkaline etching

Secondly, K_2CO_3 and $NaHCO_3$ were tested in CH_2Cl_2 to rule out the scenario of copper etching being a purely pH dependent phenomenon, regardless of NHCs. Also here, no signs of etching were observed within two weeks. Vice versa, non-alkaline NHC precursors were tested for etching: At 40 – 60 °C, the use of $IMe{\cdot}CO_2$ resulted in similar etching rates as $BIMeH{\cdot}HCO_3$. Dissolved CO_2 is less alkaline than HCO_3^- and probably leaves the reaction medium anyway as gas. Therefore, the basicity of the HCO_3^- plays a subordinate role for etching (except for activating the imidazolium salt of course).

As $BIMeH{\cdot}HCO_3$ causes copper etching and $NaHCO_3$ doesn't, it comes naturally to test alkaline coordinating ligands. Here, triethylamine (Et_3N, 10 µl in 1 ml CH_2Cl_2) and benzimidazole (BI, 4 mg in 1 ml CH_2Cl_2) were studied and showed etching rates of 14 and 7 nm/d, respectively. Thus, coordinating bases cause etching too and NHCs are not the only coordinating ligands enabling copper etching. But NHCs give comparatively high etching rates at a given set of conditions compared to other aminic ligands.

In this sense, MeCN – a solvent known to coordinate to copper[21] – plays a unique role. Whereas pure MeCN does not cause any etching within two weeks, 1 ml of MeCN with 10 mg of $NaHCO_3$ etches copper with around 15 nm/d, which gets even increased to 30 nm/d when 100 µl of H_2O were added for better solubility of $NaHCO_3$. Also, when analysing the surface after three days of modification, an intense N 1s signal is found at 399.0 eV (see Figure S83). MeCN is therefore not innocent on the copper surface and modification procedures using alkaline $BIMeH{\cdot}HCO_3$ in MeCN need be treated with caution. However, the NHC is expected to replace adsorbed MeCN due to higher binding energies[22] and the etching caused by MeCN is generally slower than the etching caused by NHCs.

12.3 Acidic etching

Complimentary, the acidity of the imidazolium salt NHC-H^+ (e.g. $BIMeH^+$) could play a role, as acidic metal(oxide) etching a well-known phenomenon. BIMeH·I (equally to $BIMeH^+ I^-$) was compared to $BIMeH{\cdot}HCO_3$ (equally to $BIMeH^+ HCO_3^-$) and to BIMeH·I and K_2CO_3 (equally to $BIMeH^+ K^+ CO_3^{2-}$). Here, the basicity of the counter anion increased from I^-, to HCO_3^- to CO_3^{2-}. If the etching was directly caused by the acidity of $BIMeH^+$, the use of BIMeH·I should result in higher etching rates than $BIMeH{\cdot}HCO_3$. However, the etching rate of the copper substrate increased from BIMeH·I (33 nm/d, 5 mg in 1 ml CH_2Cl_2) to $BIMeH{\cdot}HCO_3$ (100 nm/d, 5 mg in 1 ml CH_2Cl_2) to

BIMeH·I + K_2CO_3 (800 nm/d, 5 and 15 mg in 1 ml CH_2Cl_2). In consequence, the etching is not caused by the acidity of the imidazolium salt and the observed trend in etching rates simply correlates to the basicity of the counter anion and therefore to the NHC activity.

Peaks					
Name	Peak BE	FWHM eV	Area (P) CPS.eV	Atomic %	Q
N1s	399.02	2.18	7126.70	100.00	Yes

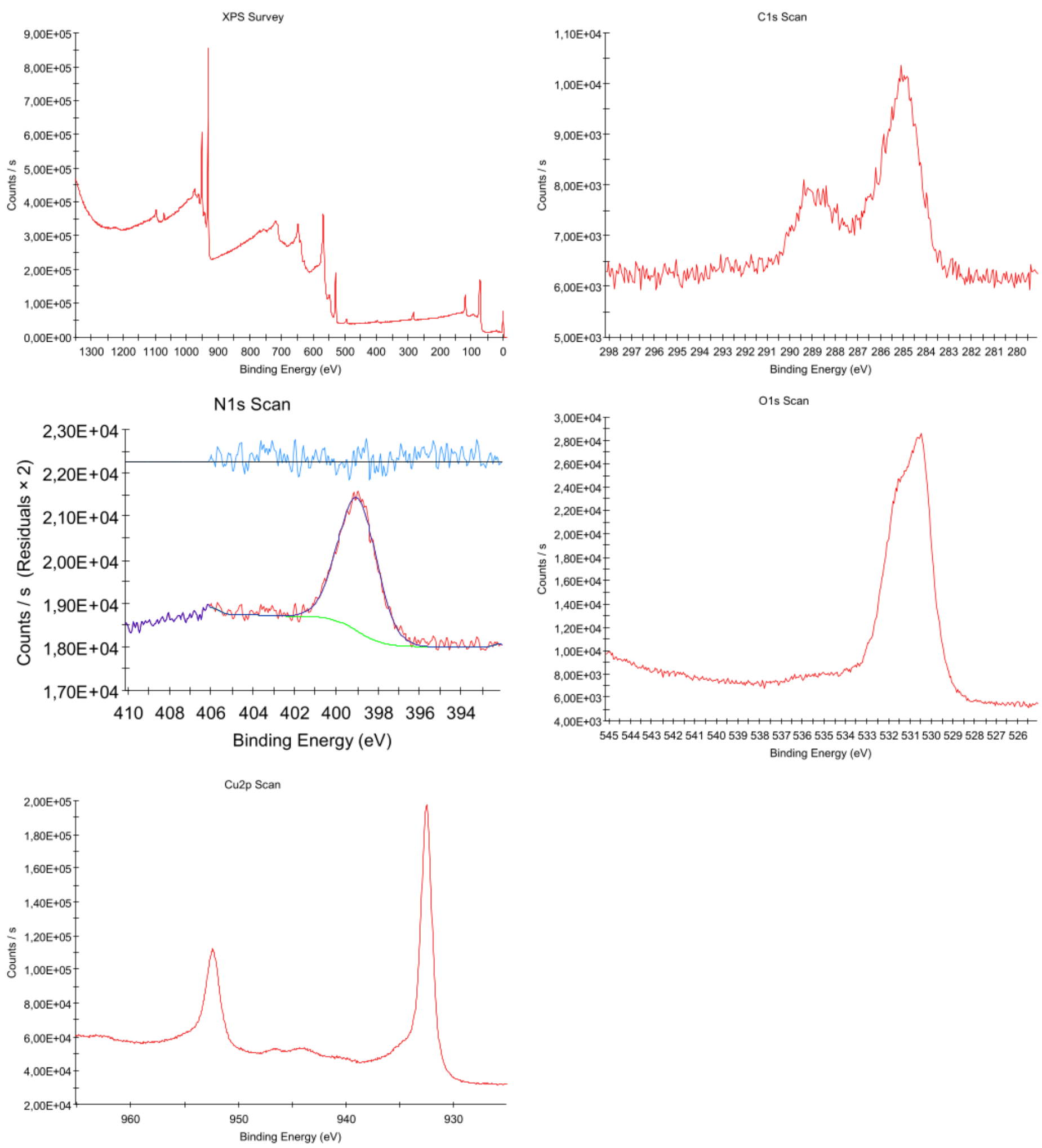

02.03.2026 15:22:11 O:\ANP\GLO_1030_HSS-HB-474\ANP-AB-137_5.vgp

Figure S83: Survey and element XPS spectra of a Cu substrate placed in MeCN (1 ml) with 10 mg of added $NaHCO_3$ for 3 days. Washed with iPrOH and acetone.

12.4 Etching with different NHCs

12.4.1 IPr

Various XPS spectra of IPr on Cu are shown in Figure 2 and Figure S25-S27 comparing IPrH·HCO_3 with IPrCO_2 and wet deposition (MeOH and CH_2Cl_2) with PVD. Similarly, to BIMe, no etching was observed when using IPrH·HCO_3 in MeOH for surface modification. Neither by eye (fading of Cu substrate) nor by HRMS (detection of IPr-Cu complexes). When switching the solvent to CH_2Cl_2, fading of the Cu substrate was observed after five days (r_e = 20 nm/d), however, no complexes were detected (maybe due to different solubility of IPr-Cu complexes compared to BIMe-Cu complexes). As for BIMe, two N 1s peaks were observed on Cu in XPS (398.9 and 400.6 eV).

12.4.2 BIiPr

Various XPS spectra of BIiPr on Cu are shown in Figure S29, S84 and S85 comparing in dichloromethane and -ethane (the latter was selected to increase comparability to literature[23]). In all cases two N 1s peaks were observed (398.7 and 400.5 eV) and the peak at lower BEs appeared more prominent. Etching was observed based on the fading of the thin layer substrate and amounts to r_e = 25 nm/d in CH_2Cl_2 and to r_e = 10 nm/d in dichloroethane. Thus, etching was slowed down in dichloroethane compared to dichloromethane and slowed down by BIiPr compared to BIMe. The predominance of the peak at lower BEs could indicate the flat-lying bis(NHC) complexes (Species III in Figure 8) to be the favored binding mode in case of BIiPr.

For BIiPr several studies discuss the orientation on Au and also on Cu in STM under ultra-high vacuum conditions:

1) BIiPr was found to bind upright to adatoms on Au(111) forming a dense and highly mobile monolayer. Upon annealing (140 °C) the upright binding mode transforms to favor flatling (bis)NHC Au complexes. At low coverage, these (bis)NHC complexes already form at room rt. Removing one methyl group in one of the wingtip groups, gives a NHC which exclusively binds as (bis)NHC adatom complex.[24]
2) BIMe forms mixtures of upright and flat-lying (bis(NHC) adatom) geometries. At elevated T, upright binding mode disappears. BIiPr binds upright to Au and Cu with high mobility due to ball-bot formation/binding to adatoms.[25]
3) BIiPr was found to bind upright on Au(111) and converts upon annealing at 90 °C to bis(NHC) complexes.[18]

It appears, that wet deposition at rt. behaves similarly to vapor phase deposition combined with thermal annealing. Unfortunately, none of the STM studies includes XPS spectra of NHCs on Cu and therefore don’t provide a clear XPS peak to structure assignment.

Just as IPr, BIiPr slows down the etching compared to BIMe due to steric hinderance of the wingtip group.

Peaks					
**Name**	**Peak BE**	**FWHM eV**	**Area (P) CPS.eV**	**Atomic %**	**Q**
N1s	398.72	1.94	11516.75	76.56	Yes
N1s Scan A	400.44	1.94	3522.53	23.44	Yes

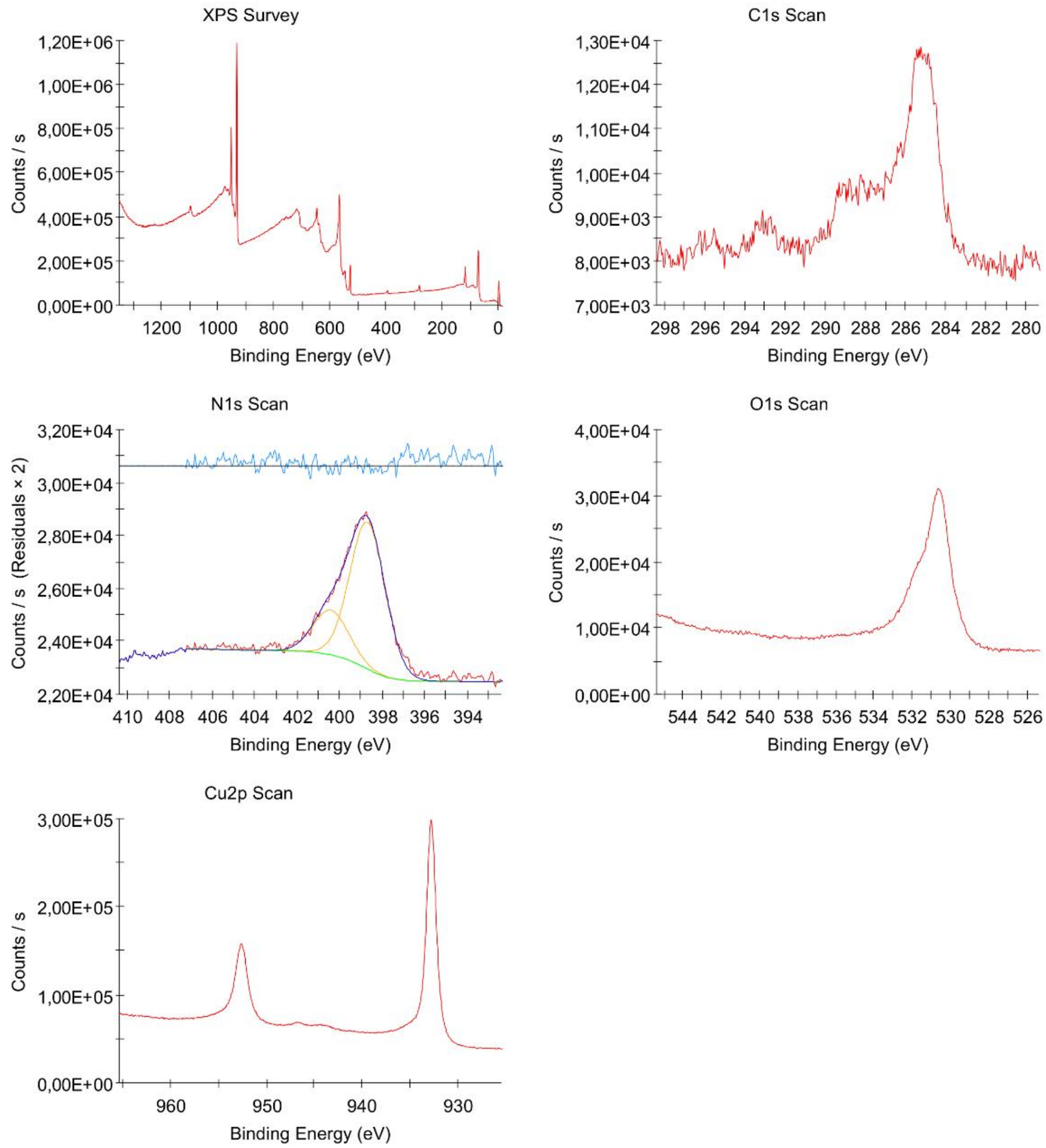

17.03.2026 16:45:18 O:\ANP\GLO_1016_HSS-HB-659 and ANP-AC-045\ANP-AC-045 05.vgp

Figure S84: Survey and element XPS spectra of a Cu substrate modified with BIiPr. Sample preparation details: Cu slide and 5 mg BIiPrH·HCO_3 in 1 ml CH_2Cl_2 at rt. for 24 h. Washed with iPrOH.

Peaks					
Name	Peak BE	FWHM eV	Area (P) CPS.eV	Atomic %	Q
N1s	398.64	1.94	4533.61	77.27	Yes
N1s Scan A	400.60	1.94	1331.97	22.73	Yes

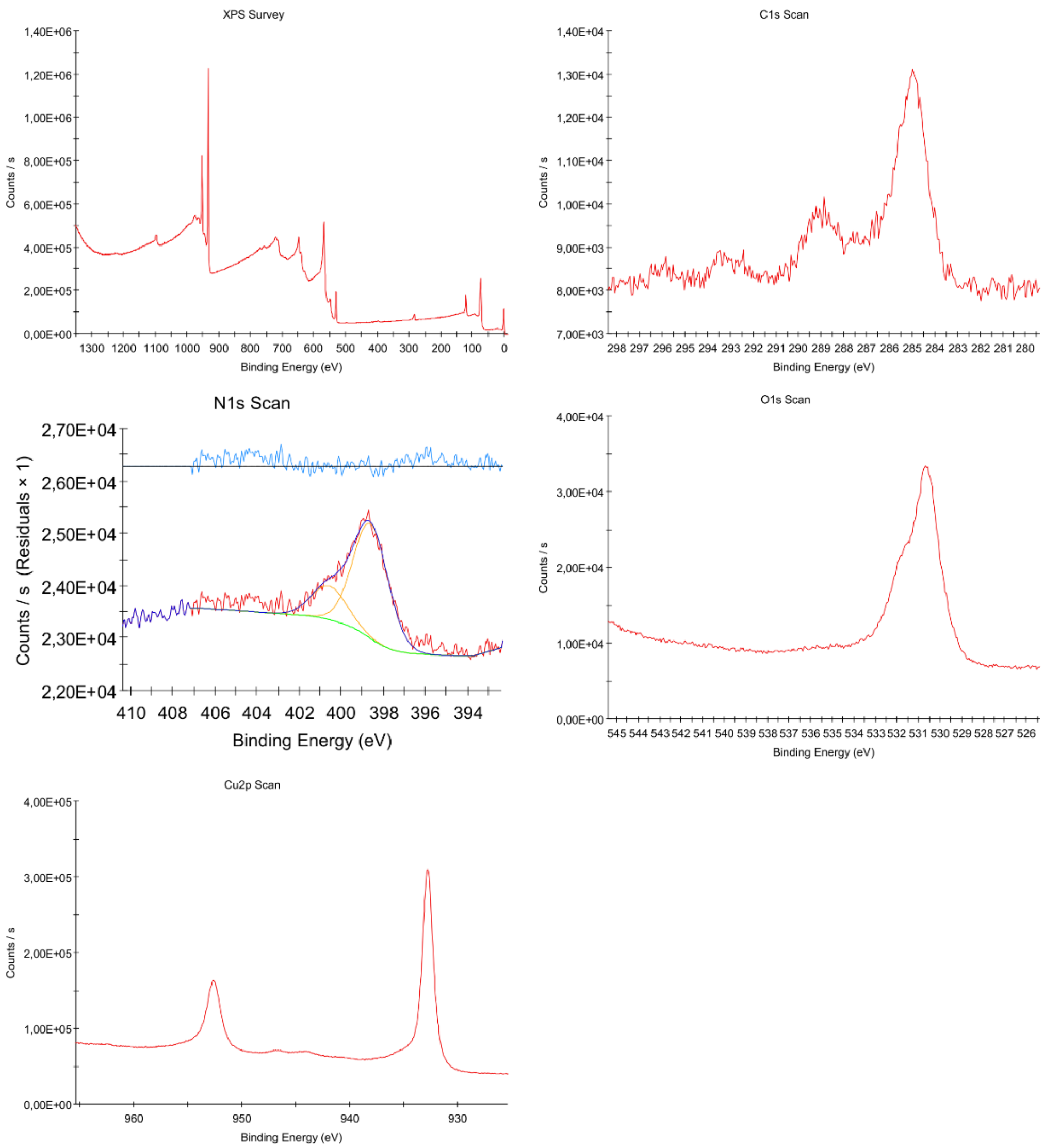

17.03.2026 16:46:21 O:\ANP\GLO_1016_HSS-HB-659 and ANP-AC-045\ANP-AC-045 07.vgp

Figure S85: Survey and element XPS spectra of a Cu substrate modified with BIiPr. Sample preparation details: Cu slide and 5 mg BIiPrH·HCO_3 in 1 ml dichloroethane at rt. for 24 h. Washed with iPrOH.

12.5 Etching of different metals

On Ni, no etching was observed, neither by eye (fading of Cu substrate) nor by HRMS (detection of NHC-Ni complexes) upon modification with BIMeH·HCO_3 or IPrH·HCO_3 in MeOH or CH_2Cl_2.

Same applies for Pt.

Using Au as substrate, traces of $[Au(BIMe)_2]^+$ complexes were found in the modification solution (m/z calculated 489.1348, found 489.1348). Procedure and XPS spectra can be found in Figure S34. After increasing BIMeH·HCO_3 concentration in CH_2Cl_2 to 5 mg/ml, extending the modification time to two weeks, and optimizing the HRMS method (nano-spray ESI in THF), $[Au(BIMe)_2]^+$ complexes were found to be the predominant THF-soluble species within the etching solution (found 489.1343, see Figure S92). Neither with BIMe, nor with IMe, IPr nor BIiPr visible fading of the thin layer Au substrate was observed within weeks. Thus, the etching is much slower on Au than on Cu and/or the equilibrium between the flat-lying (bis)NHC Au^0 complex and dissolved $[Au(BIMe)_2]^+$ is shifted towards the neutral Au^0 species due to the higher redox potential of Au compared to Cu.

Reproducing literature[26], introducing a nitro substituent in the NHC backbone was found to etch the thin layer Au substrate (6 mg BIMeH·I, 1 mg K_2CO_3, 1 ml MeCN, r_e = 50 nm/d).

In summary, the NHC mediated etching seems to be limited to metals which are prone to undergo one electron oxidations (M^0 to M^I) like coinage metals. Within the coinage metals, the redox potential governs the equilibrium between solid phase M^0 and dissolved $[M(NHC)_2]^+$.

13 XRD Measurements

X-ray diffraction (XRD) data sets for compounds **CS1** and **CS2** were collected with a Bruker D8 Venture Photon III Diffractometer. Programs used: data collection: *APEX4* Version 2021.4-0[27] (Bruker AXS Inc., **2021**); cell refinement: *SAINT* Version 8.40B (Bruker AXS Inc., **2021**); data reduction: *SAINT* Version 8.40B (Bruker AXS Inc., **2021**); absorption correction, *SADABS* Version 2016/2 (Bruker AXS Inc., **2021**); structure solution *SHELXT*-Version 2018-3[28] (Sheldrick, G. M. *Acta Cryst.,* **2015**, *A71*, 3-8); structure refinement *SHELXL*- Version 2018-3[29] (Sheldrick, G. M. *Acta Cryst.,* **2015**, *C71* (1), 3-8) and graphics, *XP*[30] (Version 5.1, Bruker AXS Inc., Madison, Wisconsin, USA, **1998**). *R*-values are given for observed reflections, and wR^2 values are given for all reflections.

13.1.1 X-ray crystal structure analysis of **CS1** (glo10594)

A red, prism-like specimen of $C_{48}H_{64}CuF_{12}N_8O_{10}P_2$, approximate dimensions 0.142 mm x 0.212 mm x 0.246 mm, was used for the X-ray crystallographic analysis. The X-ray intensity data were measured on a single crystal diffractometer Bruker D8 Venture Photon III system equipped with a micro focus tube Cu ImS (CuKα, λ = 1.54178 Å) and a MX mirror monochromator. A total of 1544 frames were collected. The total exposure time was 12.77 hours. The frames were integrated with the Bruker SAINT software package using a wide-frame algorithm. The integration of the data using a monoclinic unit cell yielded a total of 120192 reflections to a maximum θ angle of 66.81° (0.84 Å resolution), of which 9897 were independent (average redundancy 12.144, completeness = 99.4%, R_{int} = 7.84%, R_{sig} = 3.34%) and 7359 (74.36%) were greater than $2\sigma(F^2)$.

The final cell constants of a = 22.0435(4) Å, b = 11.4820(2) Å, c = 22.4636(5) Å, β = 99.7240(10)°, volume = 5603.93(19) Å^3, are based upon the refinement of the XYZ-centroids of 9933 reflections above 20 σ(I) with 8.710° < 2θ < 133.2°. Data were corrected for absorption effects using the multi-scan method (SADABS). The ratio of minimum to maximum apparent transmission was 0.817. The calculated minimum and maximum transmission coefficients (based on crystal size) are 0.6390 and 0.7650. The structure was solved and refined using the Bruker SHELXTL Software Package, using the space group $P2_1/c$, with Z = 4 for the formula unit, $C_{48}H_{64}CuF_{12}N_8O_{10}P_2$. The final anisotropic full-matrix least-squares refinement on F^2 with 983 variables converged at R1 = 4.99%, for the observed data and wR2 = 15.26% for all data. The goodness-of-fit was 1.032. The largest peak in the final difference electron density synthesis was 0.531 e^-/Å^3 and the largest hole was -0.412 e^-/Å^3 with an RMS deviation of 0.059 e^-/Å^3. On the basis of the final model, the calculated density was 1.501 g/cm^3 and F(000), 2620 e^-. CCDC number: 2532664.

13.1.2 Refinement details for **CS1**

This compound crystallised with two half-complex molecules in the asymmetric unit. The corresponding PF_6 anions and two of the three ethyl acetate solvent molecules were found disordered over two positions in the asymmetric unit. Several restraints (SADI, SAME, ISOR and SIMU) were used in order to improve refinement stability.

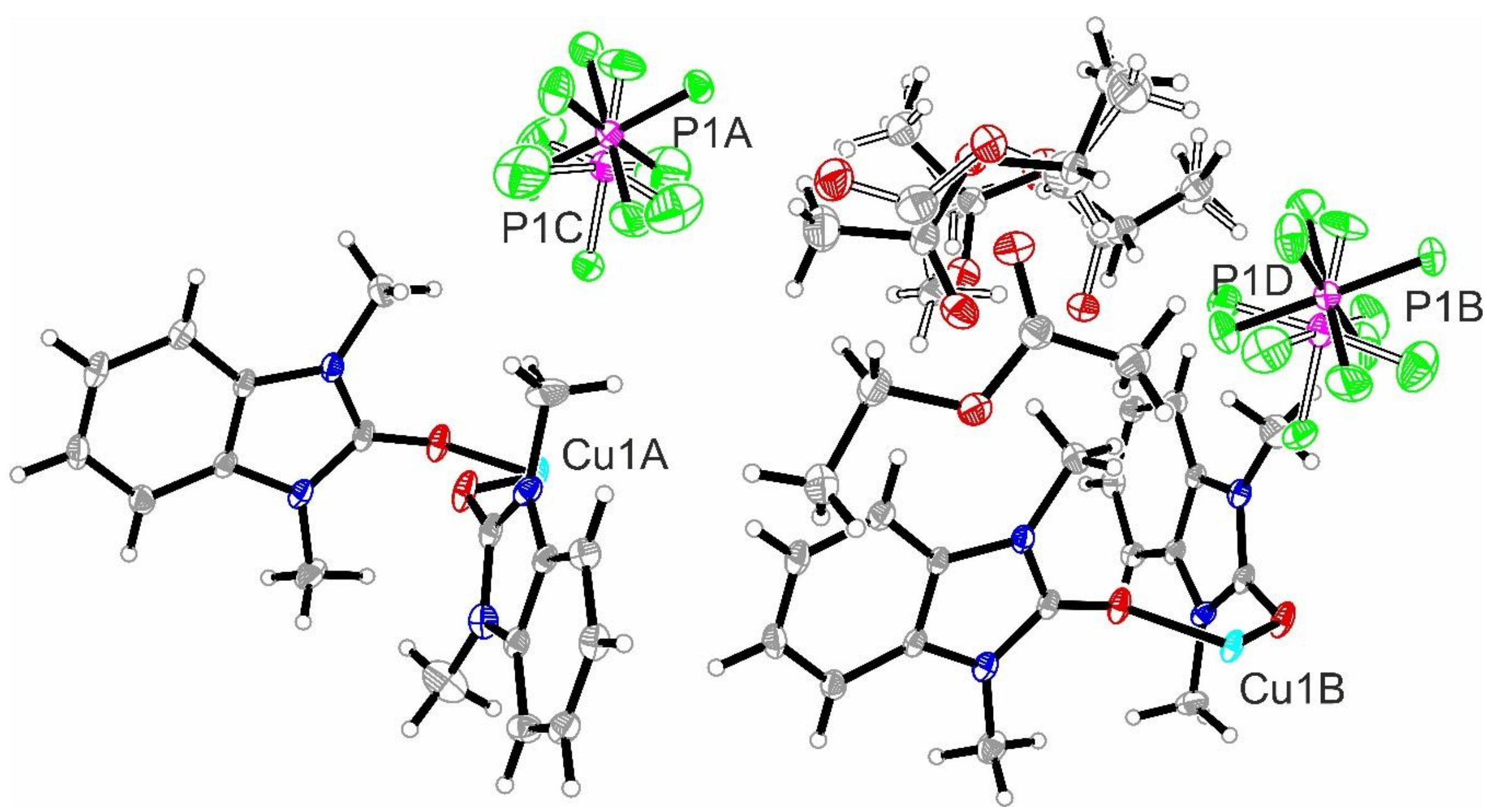

Figure S86: Asymmetric unit of compound **CS1** representing two half-complex molecules, two disordered PF_6 anions, one entire and two disordered over two positions ethyl acetate solvent molecules. Thermal ellipsoids are shown at 30% probability.

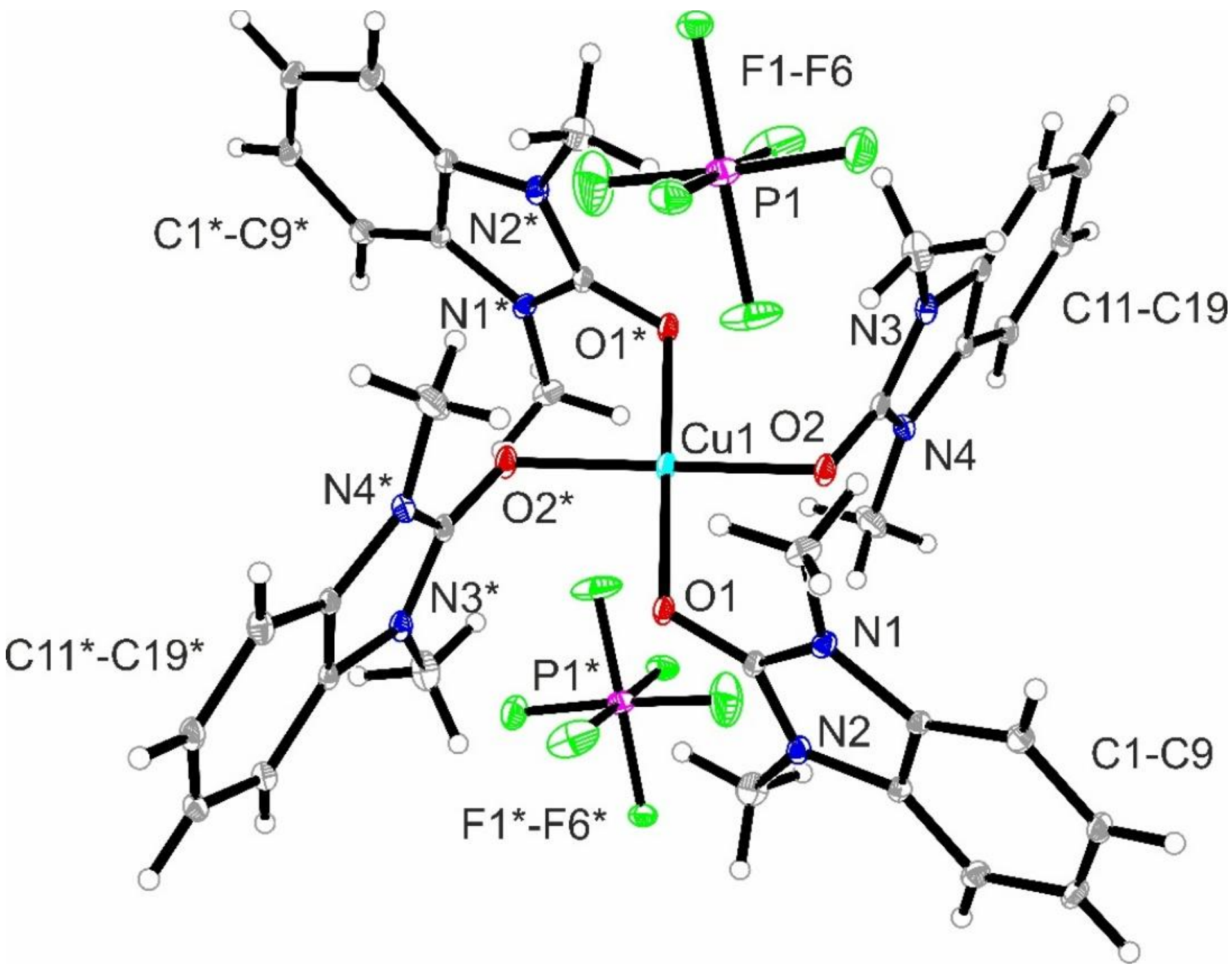

Figure S87: Crystal structure of compound **CS1**. Thermal ellipsoids are shown at 30% probability. Symmetry transformations used to generate equivalent atoms is * = -x+1, -y+1, -z+1.

13.2.1 X-ray crystal structure analysis of **CS2** (glo10604)

A colorless, prism-like specimen of $C_{72}H_{88}CuF_{12}N_{16}O_{12}P_2$, approximate dimensions 0.074 mm x 0.083 mm x 0.187 mm, was used for the X-ray crystallographic analysis. The X-ray intensity data were measured on a single crystal diffractometer Bruker D8 Venture Photon III system equipped with a micro focus tube Mo ImS (MoKα, λ = 0.71073 Å) and a MX mirror monochromator. A total of 852 frames were collected. The total exposure time was 4.73 hours. The frames were integrated with the Bruker SAINT software package using a narrow-frame algorithm. The integration of the data using a triclinic unit cell yielded a total of 40900 reflections to a maximum θ angle of 25.03° (0.84 Å resolution), of which 6742 were independent (average redundancy 6.066, completeness = 99.7%, R_{int} = 5.50%, R_{sig} = 3.33%) and 5605 (83.14%) were greater than $2\sigma(F^2)$. The final cell constants of a = 12.3751(7) Å, b = 13.8217(9) Å, c = 13.8665(8) Å, α = 106.895(2)°, β = 111.478(2)°, γ = 105.785(2)°, volume = 1911.6(2) $Å^3$, are based upon the refinement of the XYZ-centroids of 8430 reflections above 20 σ(I) with 4.999° < 2θ < 54.60°. Data were corrected for absorption effects using the multi-scan method (SADABS). The ratio of minimum to maximum apparent transmission was 0.927. The calculated minimum and maximum transmission coefficients (based on crystal size) are 0.9240 and 0.9690. The structure was solved and refined using the Bruker SHELXTL Software Package, using the space group *P*-1, with Z = 1 for the formula unit, $C_{72}H_{88}CuF_{12}N_{16}O_{12}P_2$. The final anisotropic full-matrix least-squares refinement on F^2 with 599 variables converged at R1 = 3.34%, for the observed data and wR2 = 8.62% for all data. The goodness-of-fit was 1.025. The largest peak in the final difference electron density synthesis was 0.260 $e^-/Å^3$ and the largest hole was -0.389 $e^-/Å^3$ with an RMS deviation of 0.051 $e^-/Å^3$. On the basis of the final model, the calculated density was 1.497 g/cm^3 and F(000), 895 e^-. CCDC number: 2532665.

13.2.2 Refinement details for **CS2**

This compound crystallised with one half-complex molecule and three additional free ligands in the asymmetric unit. The hydrogens at O5 and O6 atoms (coordinated water molecules) were refined freely, but with O-H distance restraints (DFIX). The corresponding PF_6 anion was found disordered over two positions in the asymmetric unit. Several restraints (SADI, SAME, ISOR and SIMU) were used in order to improve refinement stability.

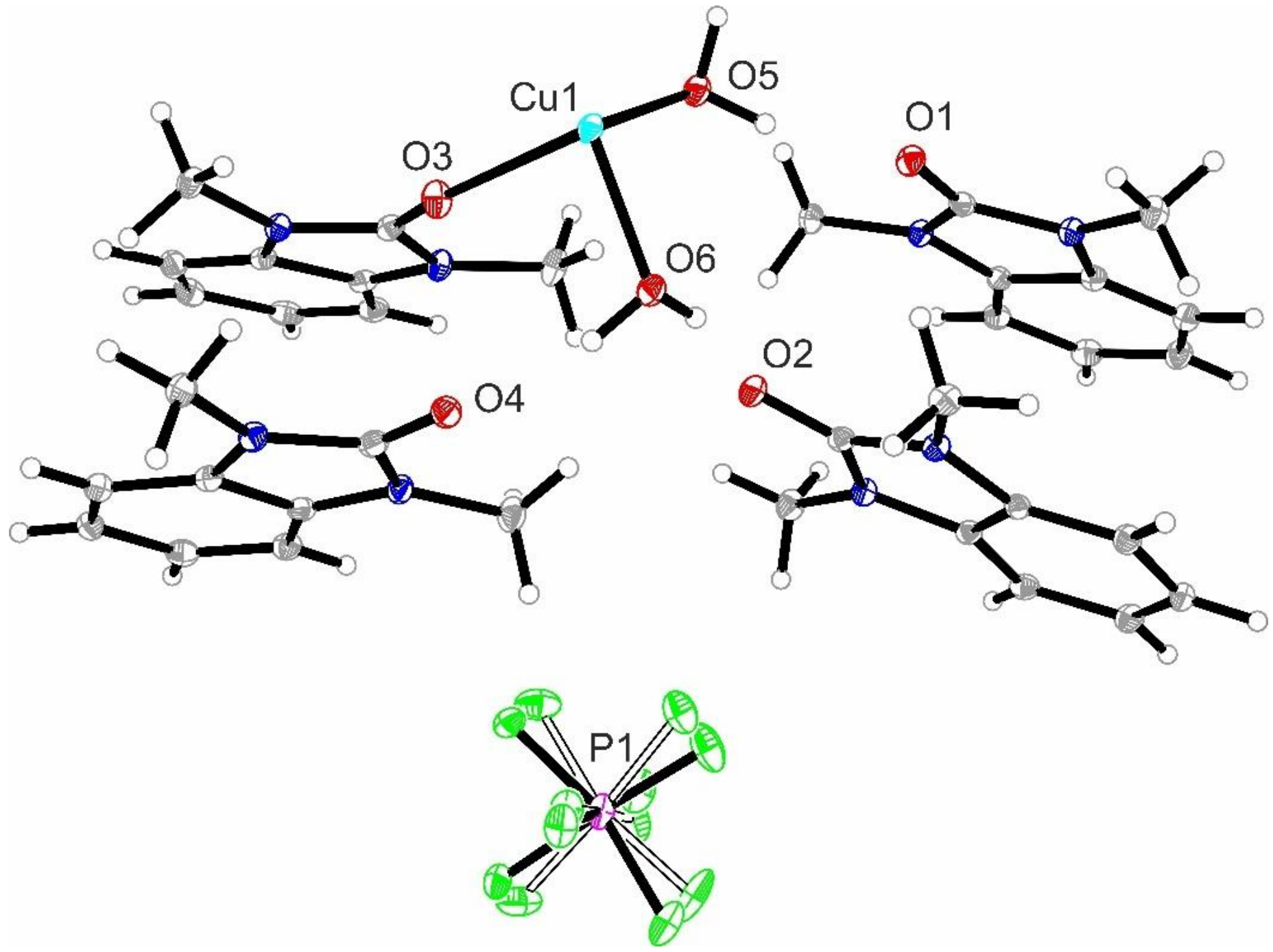

Figure S88: Asymmetric unit of compound **CS2** representing one half-complex molecules, one disordered PF_6 anion and three uncoordinated ligand molecules. Thermal ellipsoids are shown at 30% probability.

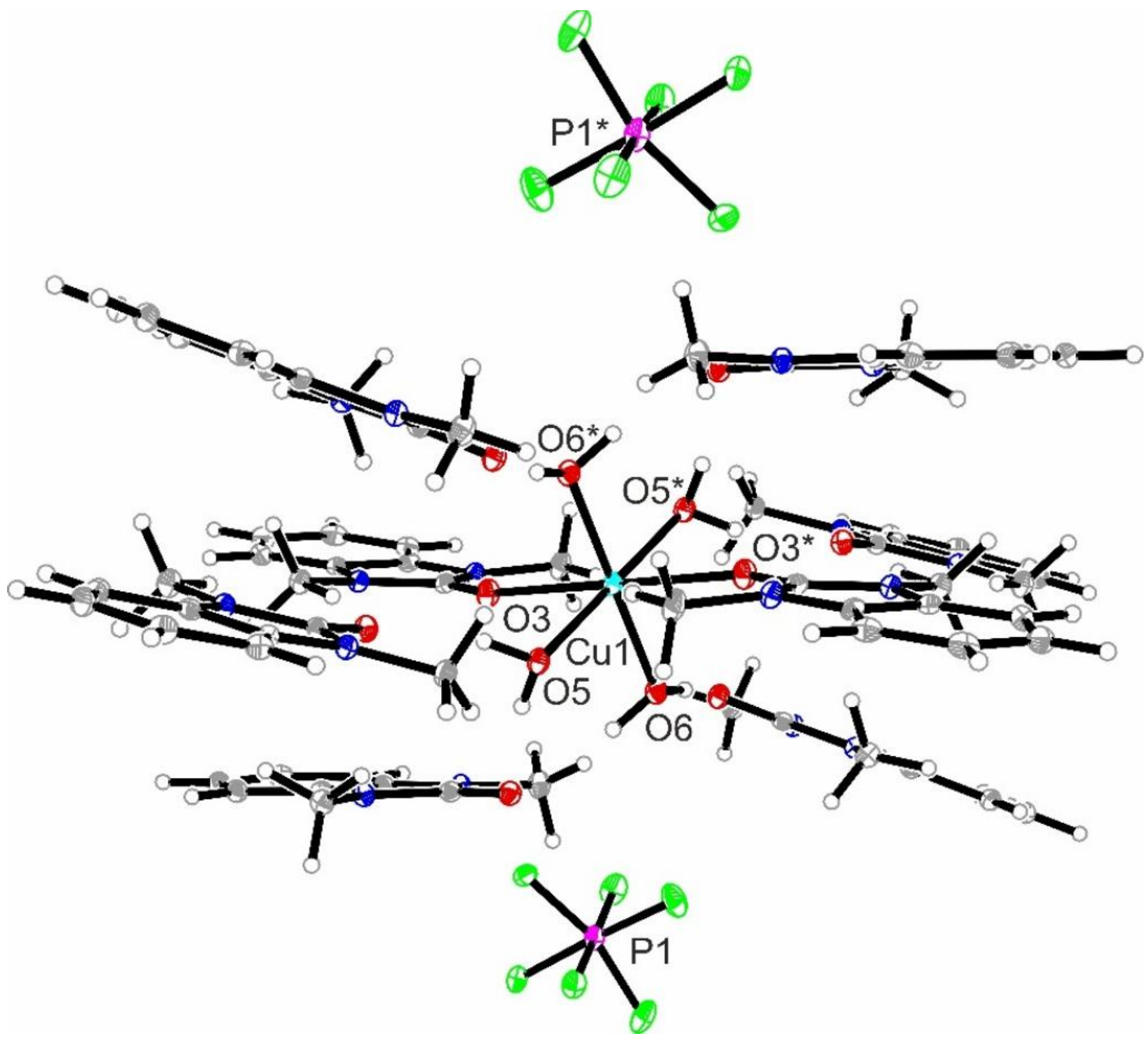

Figure S89: Completed unit cell for compound **CS2**. Symmetry transformations used to generate equivalent atoms is * = -x+2, -y+2, -z+1.

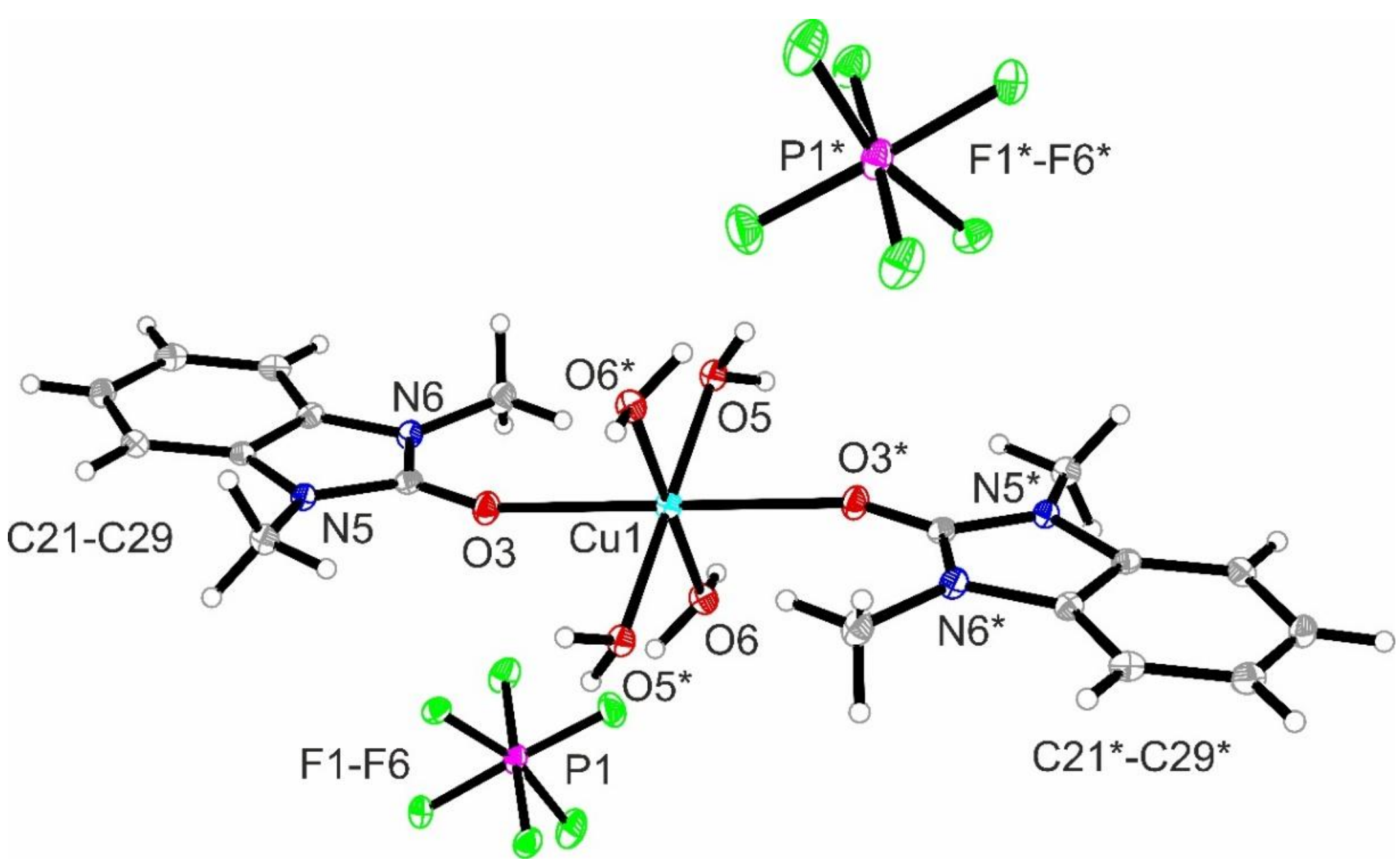

Figure S90: Crystal structure of compound **CS2**. Thermal ellipsoids are shown at 30% probability. Symmetry transformations used to generate equivalent atoms is * = -x+2, -y+2, -z+1.

14 Mass Spectrometry of Etching Solutions

ESI mass spectra of etching solutions were recorded on an LTQ Orbitrap LTQ XL (Thermo-Fisher Scientific, Bremen) with a nano-spray inlet (10 µl sample volume, 1 kV capillary voltage) and evaluated with Xcalibur (Thermo Fisher Scientific).

Some ESI nano-spray spectra of etching solutions are depicted including sample preparation details and peak assignment.

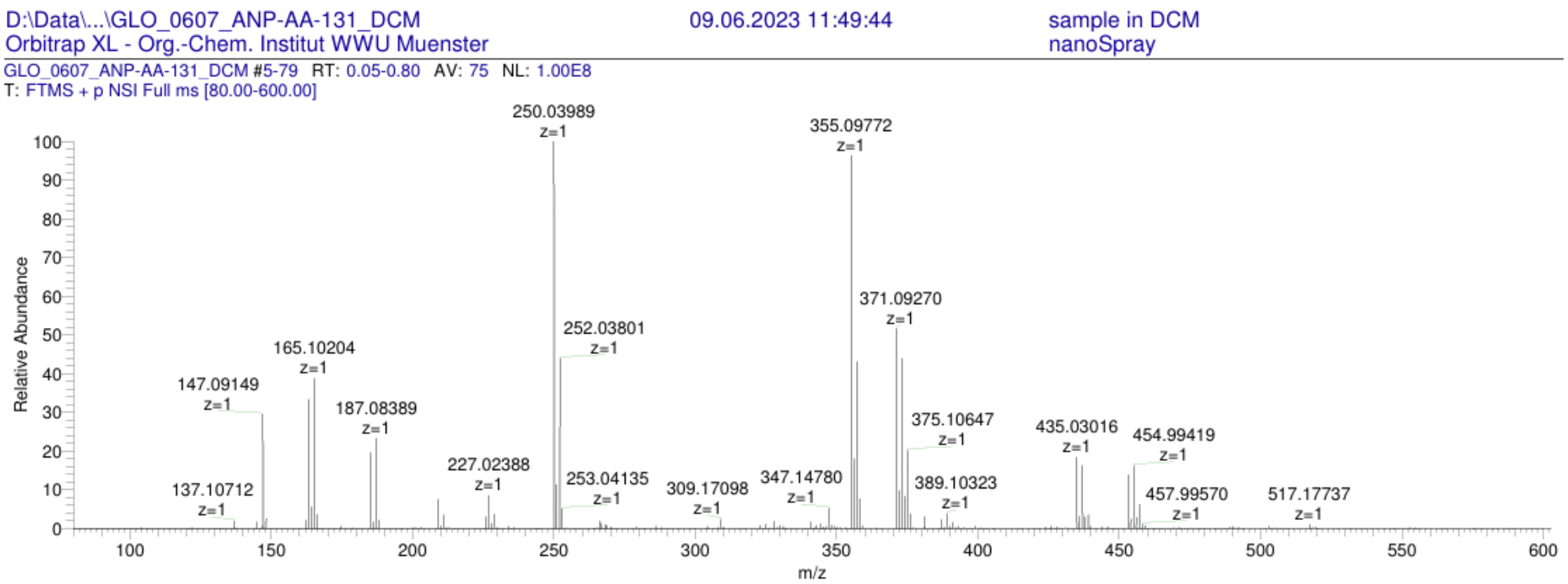

Figure S91: ESI nano-spray spectrum of an etching solution. Sample preparation details: 5 mg BIMeH·HCO_3, 2 ml CH_2Cl_2 and 20 mg Cu powder were stirred under ambient conditions for 3 d. m/z calculated for [BIMeH]$^+$ 147.0917, found 147.0915. m/z calculated for [BIMeO+H]$^+$ 163.0866, found 163.0866. m/z calculated for [**2**+H]$^+$ 165.1022, found 165.1020. m/z calculated for [BIMeO+Na]$^+$ 185.0685, found 185.0685. m/z calculated for [**2**+Na]$^+$ 187.0842, found 187.0839. m/z calculated for [Cu(BIMe)(MeCN)]$^+$ 250.0400, found 250.0399. m/z calculated for [Cu(BIMe)$_2$]$^+$ 355.0978, found 355.0977. m/z calculated for [Cu(BIMe)(BIMeO)]$^+$ 371.0928, found 371.0927.

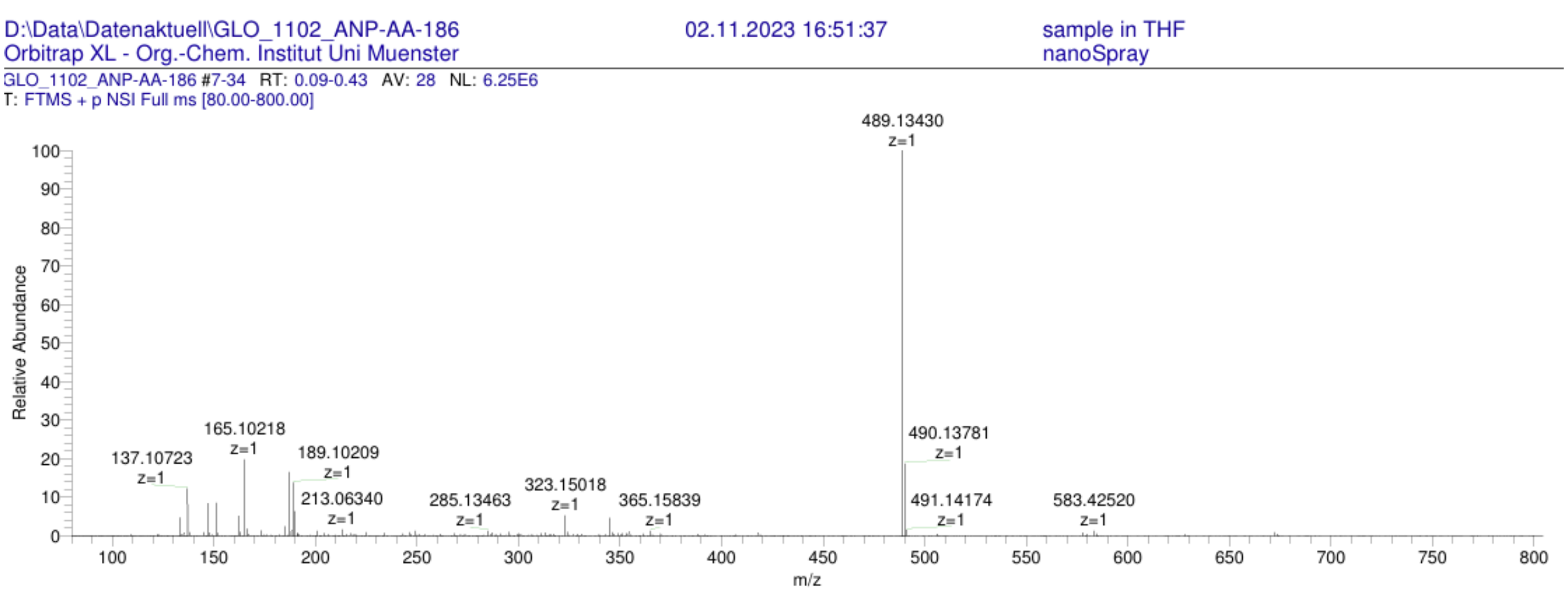

Figure S92: ESI nano-spray spectrum of an etching solution. Sample preparation details: 5 mg BIMeH·HCO_3 and a thin layer Au substrate were stored in 1 ml CH_2Cl_2 at ambient conditions for 2 weeks. m/z calculated for [**2**+H]$^+$ 165.1022, found 165.1022. m/z calculated for [Au(BIMe)$_2$]$^+$ 489.1354, found 489.1343.

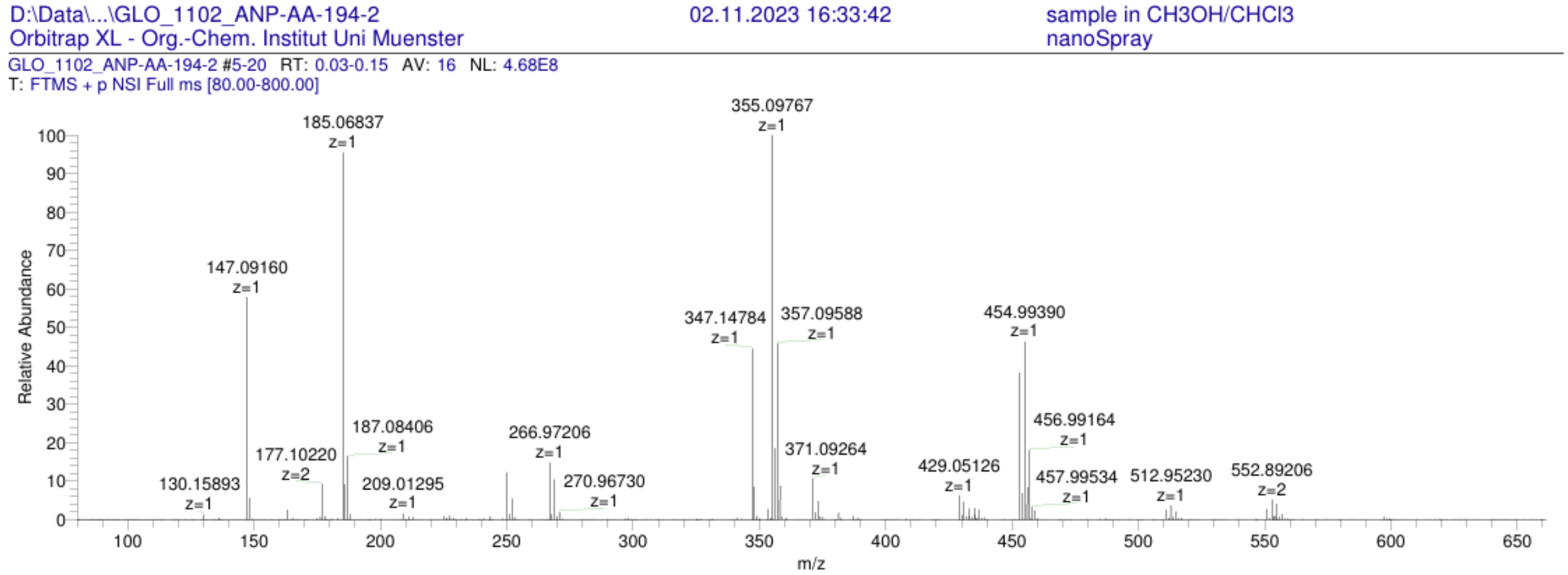

Figure S93: ESI nano-spray spectrum of an etching solution. Sample preparation details: 5 mg BIMeH·HCO_3 and Cu foil were stored in 1 ml CH_2Cl_2 under ambient conditions for one week. m/z calculated for $[BIMeH]^+$ 147.0917, found 147.0916. m/z calculated for $[BIMeO+Na]^+$ 185.0685, found 185.0684. m/z calculated for [**2**+Na]$^+$ 187.0842, found 187.0841. m/z calculated for $[Cu(BIMe)(MeCN)]^+$ 250.0400, found 250.0400. m/z calculated for $[Cu(BIMe)_2]^+$ 355.0978, found 355.0977. m/z calculated for $[Cu(BIMe)(BIMeO)]^+$ 371.0928, found 371.0926.

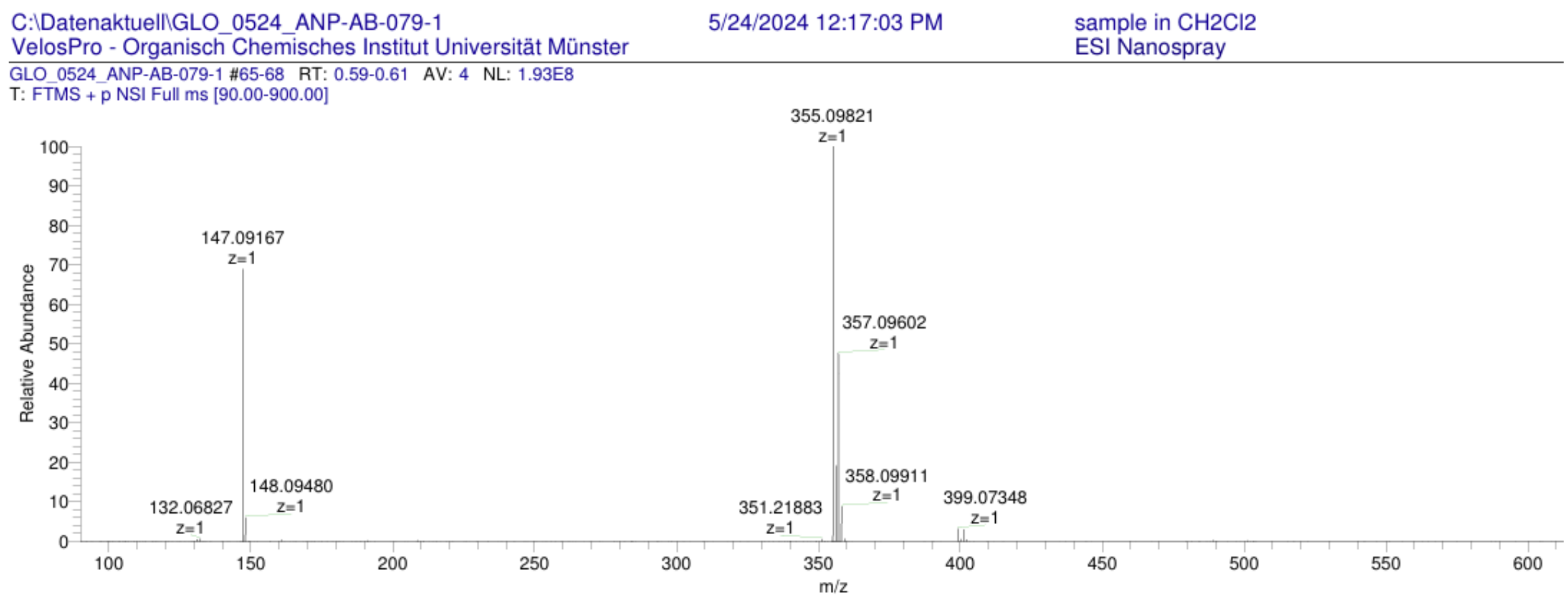

Figure S94: ESI nano-spray spectrum of an etching solution. Sample preparation details: 6.5 mg BIMeH·HCO_3, 1 ml CH_2Cl_2 and a thin layer Cu substrate (2.0x1.0 cm^2) were stored at room temperature inside a glovebox (O_2 and H_2O levels below 0.5 ppm) for 2 weeks. m/z calculated for $[BIMeH]^+$ 147.0917, found 147.0917. m/z calculated for $[Cu(BIMe)_2]^+$ 355.0978, found 355.0982.

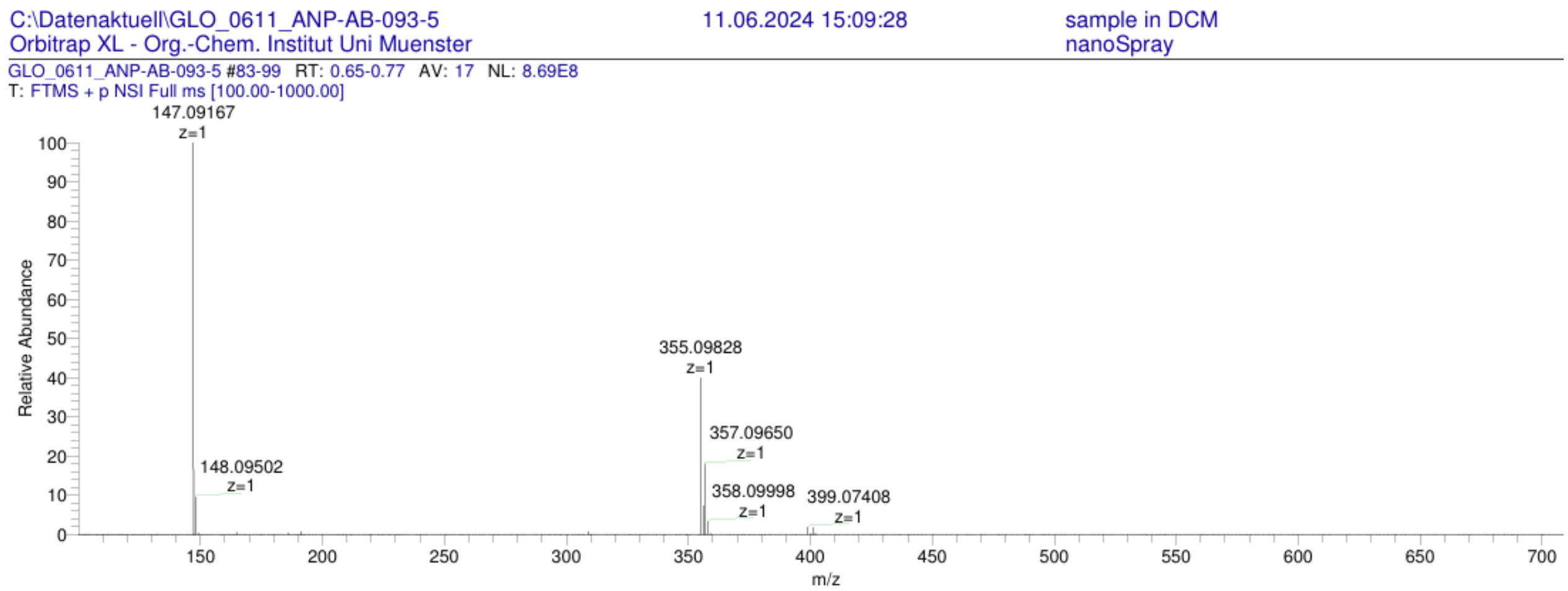

Figure S95: ESI nano-spray spectrum of an etching solution. Sample preparation details: 5 mg BIMeH·HCO_3, 0.5 ml MeCN and a thin layer Cu substrate (0.5x0.5 cm^2) were stored at room temperature inside a glovebox (O_2 and H_2O levels below 0.5 ppm) for 1 d. m/z calculated for $[BIMeH]^+$ 147.0917, found 147.0917. m/z calculated for $[Cu(BIMe)_2]^+$ 355.0978, found 355.0983.

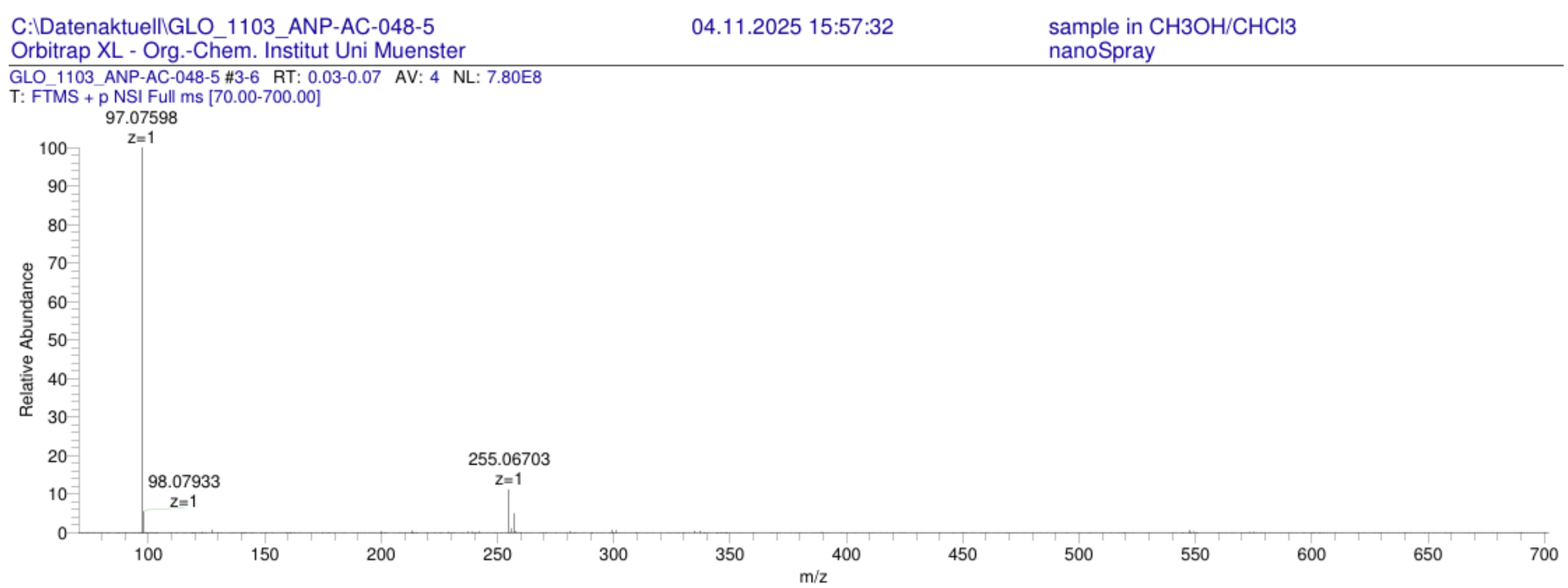

Figure S96: ESI nano-spray spectrum of an etching solution. Sample preparation details: 10 mg $IMeCO_2$, 1 ml CH_2Cl_2 and a thin layer Cu substrate (0.5x0.5 cm^2) were sealed in a Schlenk flask and stored at 60 °C for 1 h. m/z calculated for $[IMeH]^+$ 97.0760, found 97.0760. m/z calculated for $[Cu(IMe)_2]^+$ 255.0665, found 255.0670.

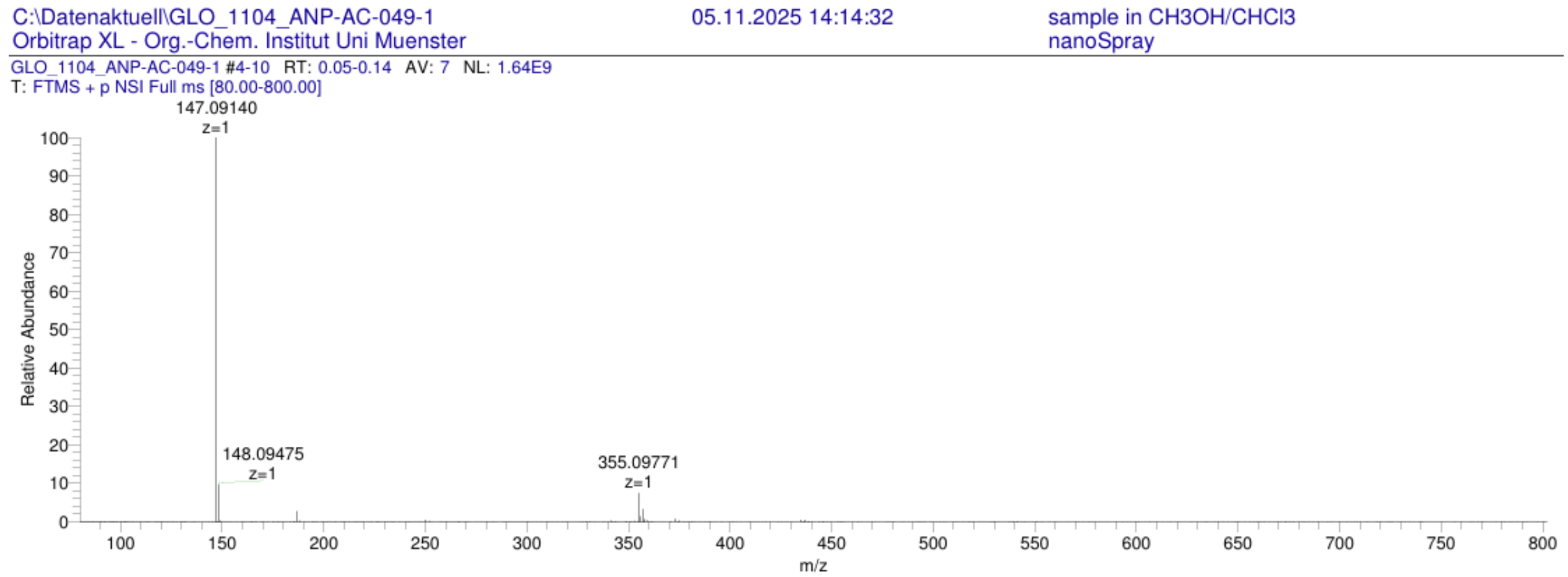

Figure S97: ESI nano-spray spectrum of an etching solution. Sample preparation details: 5 mg BIMeH·HCO_3, 1 ml CH_2Cl_2 and a thin layer Cu substrate (0.5x0.5 cm^2) were sealed in a Schlenk flask and stored at 60 °C for 30 minutes. m/z calculated for $[BIMeH]^+$ 147.0917, found 147.0914. m/z calculated for $[Cu(BIMe)_2]^+$ 355.0978, found 355.0977.

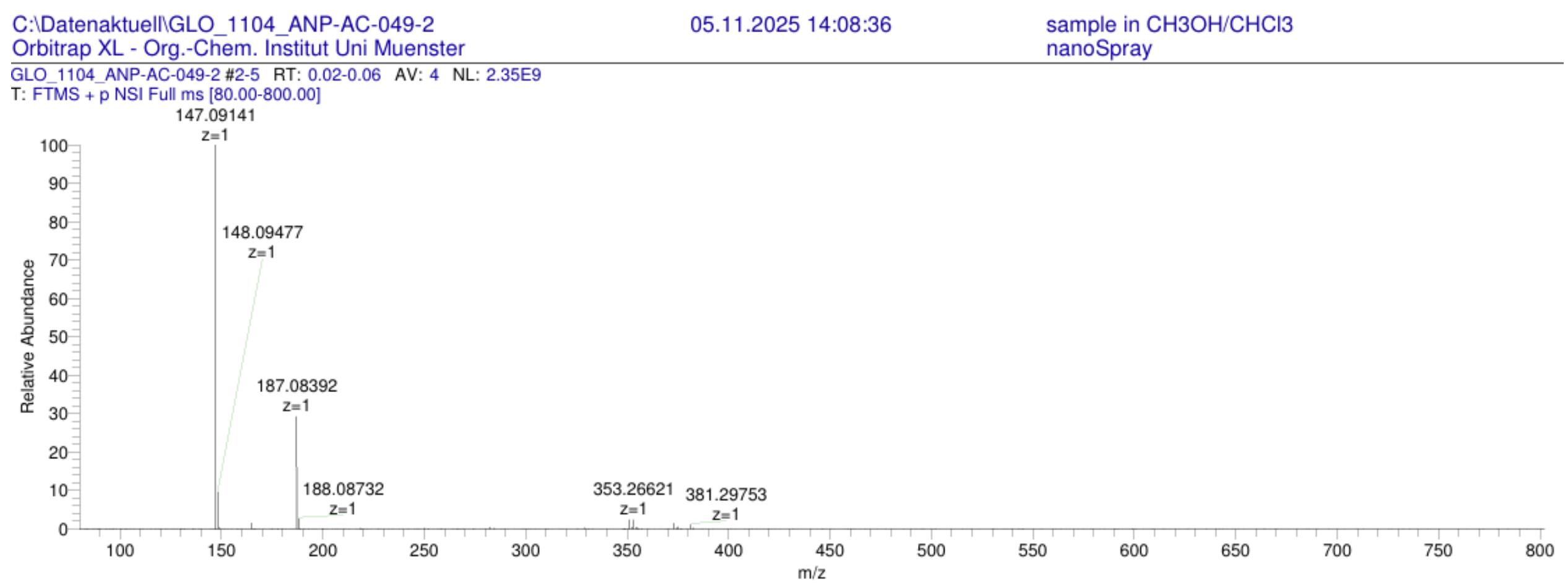

Figure S98: ESI nano-spray spectrum of an etching solution. Sample preparation details: 5 mg BIMeH·HCO_3, 2 mg acetone oxime, 1 ml CH_2Cl_2 and a thin layer Cu substrate (0.5x0.5 cm^2) were sealed in a Schlenk flask and stored at 60 °C for 30 minutes. m/z calculated for $[BIMeH]^+$ 147.0917, found 147.0914. m/z calculated for [**2**+Na]$^+$ 187.0842, found 187.0839.

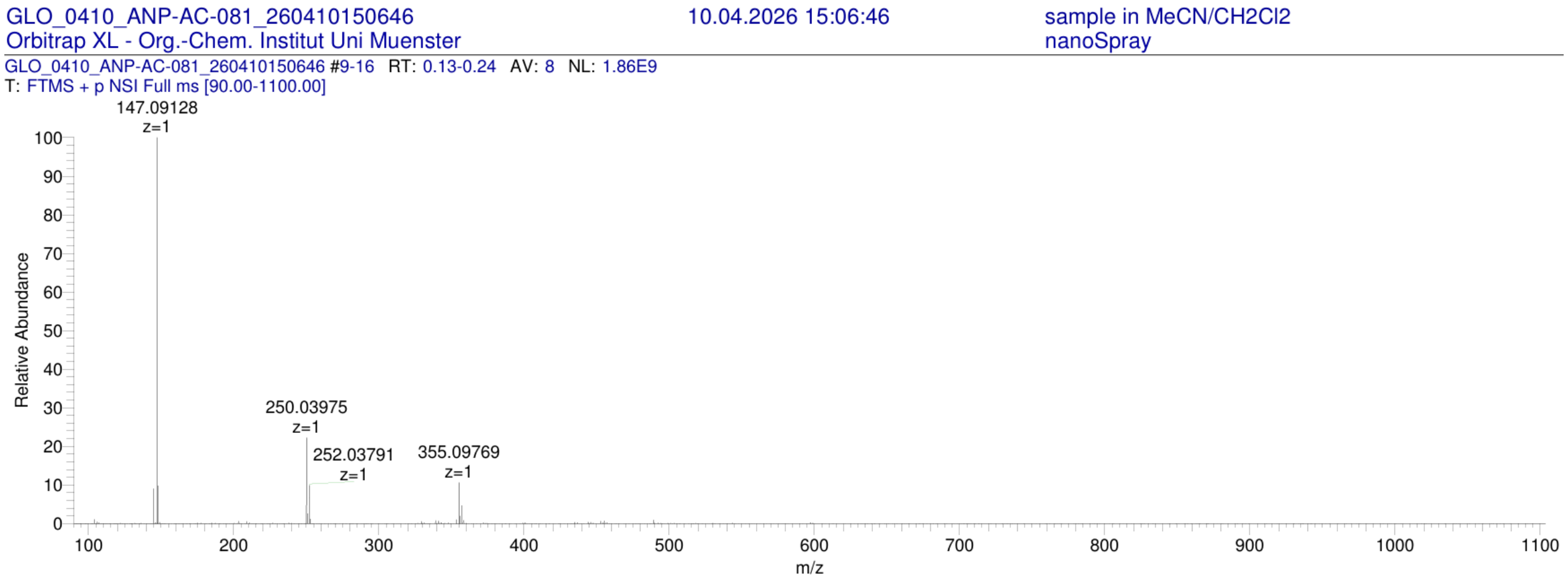

Figure S99: ESI nano-spray spectrum of a washing solution. Sample preparation details: PVD modification of thin layer Cu substrate (1x2 cm^2) with BIMeH·HCO_3 at 100 °C for 15 minutes. Sample was washed with 0.5 ml CH_2Cl_2 by repeatedly rinsing the surface with the same solution. m/z calculated for $[BIMeH]^+$ 147.0917, found 147.0913. m/z calculated for $[Cu(BIMe)(CH_3CN)]^+$ 250.0400, found 250.0398. m/z calculated for $[Cu(BIMe)_2]^+$ 355.0978, found 355.0977.

15 DFT Studies

Periodic non-spin-polarized DFT calculations were carried out using the VASP package within the framework of the Projector Augmented Wave (PAW) method.[31–34] The electronic structure was described using PAW pseudopotentials in combination with the non-local optPBE-vdW functional and an energy cutoff of 450 eV.[35] Brillouin zone integrations were performed using a Gamma-centered 1×1×1 k-point grid.[36] Convergence study of the k-point mesh on the surface energy and NHC adsorption energy can be found in Table S7, showing that a simple Gamma centered mesh is sufficient with a large enough supercell. Electronic occupancies were treated with the Methfessel–Paxton smearing scheme (first order, σ = 0.02).[37]

Adsorption energies were evaluated according to:

$$E_{ads} = E_{surface+molecule} - (E_{surface} + E_{molecule})$$

The surface model was constructed starting from the experimental Cu bulk unit cell obtained from the Materials Project database.[38] The slab was generated by cleaving the surface and expanding the unit cell to an 8×8×3 supercell, resulting in a three-layer model. The 3-layer model was deemed sufficient for the proprieties that were explored in the article, according to the convergence studies conducted on the surface energies (Table S8) and NHC adsorption energies on the Cu (111) surface.

Density of states (DOS) and projected-DOS (PDOS) simulations were conducted on VASP optimized structures using the Lobster software package.[39] The pbeVaspFit basis set was used to project onto the DOS, using the following orbitals for each atom: Cu 3d, 4s; C: 2s, 2p, N: 2s, 2p; H: 1s.

Table S7. Convergence tests of the surface energy and NHC adsorption energy for Cu(111) surface with respect to the density of the k-point mesh. Only the first layer was frozen in all cases.

K-point grid	Surface energy (eV/Å^2)	Adsorption energy (eV)
1x1x1	0.19	-1.49
2x2x2	0.18	-1.53
3x3x3	0.18	-1.49
4x4x4	0.18	-1.50
5x5x5	0.18	-1.48

Table S8. Convergence tests of the surface energy and NHC adsorption energy for Cu(111) surface with respect to Cu layers included. Only the first layer was frozen in all cases.

Number of Layers	Surface energy (eV/Å^2)	Adsorption energy (eV)
2	0.19	-1.41
3	0.19	-1.49
4	0.19	-1.43
5	0.19	-1.36

To better understand the proprieties of the molecular species, simulations were performed using the Gaussian 09 package.[40] These employed the PBE0 hybrid functional together with the def2-

TZVP triple-zeta basis set and an ultrafine integration grid.[41–43] The neutral open-shell species were calculated using the unrestricted DFT framework, while the cationic ones were studied within the restricted methodology. All geometries were optimized using tight convergence criteria, and their nature as local minima on the potential energy surfaces was confirmed by verifying the absence of imaginary frequencies. Theoretical XPS binding energies (BE) were approximated as being the energies of the corresponding internal molecular orbitals.[44] Natural population analysis (NPA) was conducted using the gas phase Gaussian optimized structures using the NBO 3.1 package built into the Gaussian package.[45] Mulliken composition analysis of the molecular orbitals was done using the Multiwfn code.[46,47]

XYZ coordinates of the optimized systems (both VASP and Gaussian) can be found at the end of this chapter.

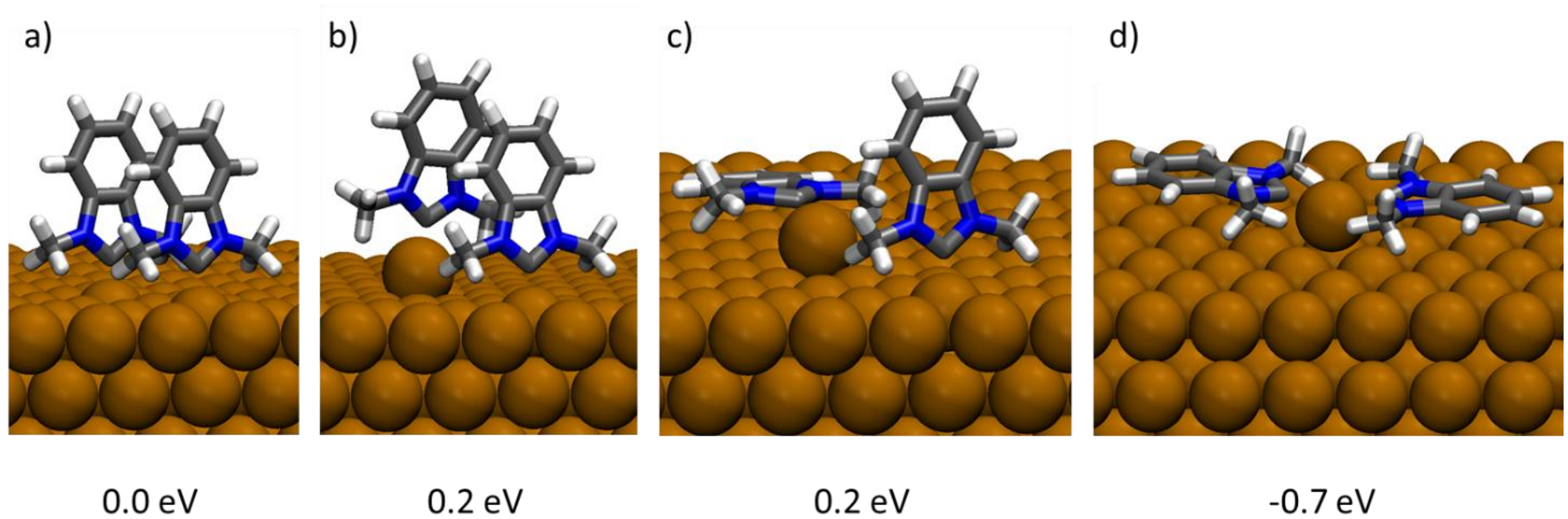

Figure S100: Adsorbed species on Cu: a) Species II, b-c) intermediate binding modes and d) species III. Their relative energies with respect to the upright adsorbed carbene (species II) are noted.

All the most important systems which are part of the etching mechanism were optimized using VASP, according to the settings presented in the computational details. For the energy of the models to be comparable, the number of Cu atoms was conserved among all. Therefore, we simulate the adatom complex formation by extracting a Cu atom from the surface and the hole is filled by an atom from the second layer. Based on the optimized geometries shown in Figure S100, molecular footprints were estimated for both binding modes: The footprint of the upright binding mode of BIMe on Cu amounts to 22.6 $Å^2$ (corresponds to a coverage of 4.43 nm^{-2}) and the footprint of the (bis)BIMe adatom complex amounts to 133.3 $Å^2$ (coverage of 0.75 nm^{-2} with respect to the bis-complex or 1.50 nm^{-2} with respect to BIMe). Here, both binding modes were treated as ideal squares and each hydrogen atom was associated with a van der Waals radius[48] of 1.0 Å. Comparing the coverages obtained by DFT with the ones measured by XPS (see Section 5), the DFT based monolayer coverage of species II is (slightly) above the measured values and for species III the reverse applies, it being below the measured values. This is expected, as the measured sample assumingly contains a mixture of both binding modes. Associating species II to the XPS N 1s peak at 400.7 eV and the (bis)NHC adatom complex to the N 1s peak at 398.8 eV, the coverages of both geometries were weighted according to their peak area ratio (see Table S2, Figure S52). This gives an ideal monolayer coverage of 3.8 nm^{-2} for BIMe on Cu, which agrees roughly with the coverage determined by XPS.

The relative energies depicted in Figure S100 show that the formation of the (bis)NHC adatom complex is favored in energy. Further, the redox potentials calculated from the gas phase α-HOMO energies of the complexes [Cu(BIMe)] and [Cu(BIMe)$_2$] indicate the (bis)NHC complex to be more prone to undergo oxidation due to its high reduction potential (see Figure S102). The calculated adsorption energies follow the same trend as the relative energies of the different binding geometries. Species II has an adsorption energy of -1.6 eV per NHC, and species III is favored with an adsorption energy of -2.4 eV per NHC. Note that these adsorption energies do not imply any restructuring of the metal substrate. The restructuring energy needed to form a defect (refilled by an underlying layer), and an adatom are given as E_{struct} = +0.9 eV.

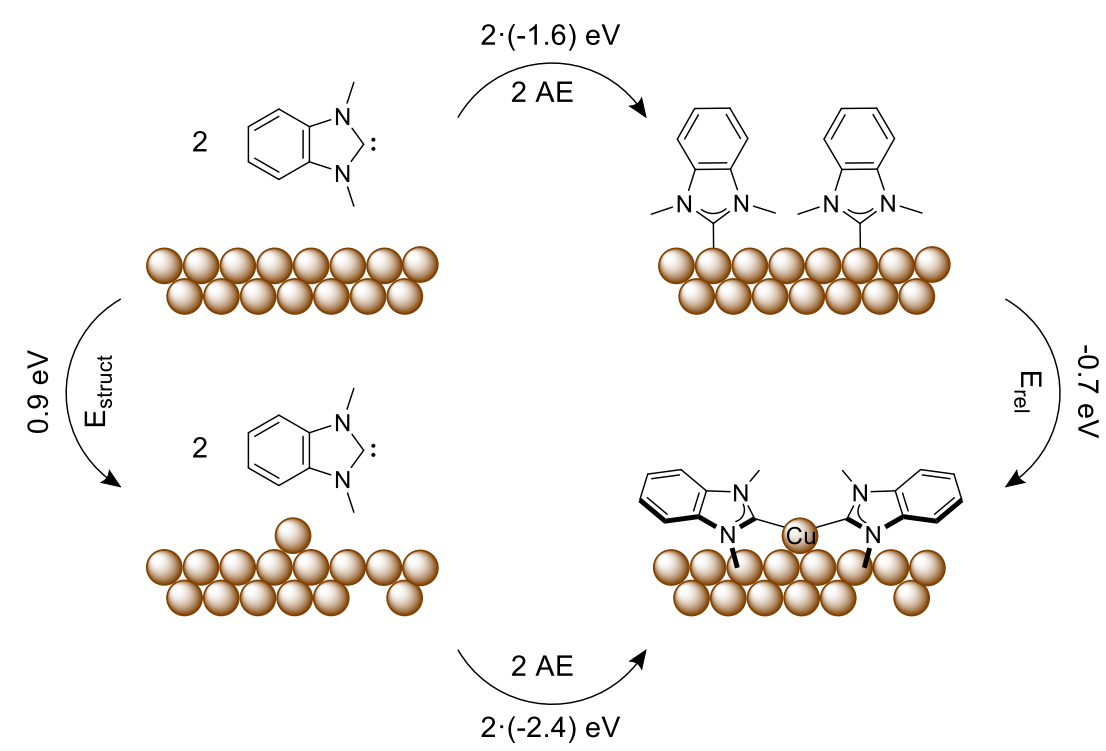

Figure S101: Connection between AE, E_{ads} and E_{rel}. As a control, adding up all four values, one ends up with 0 eV.

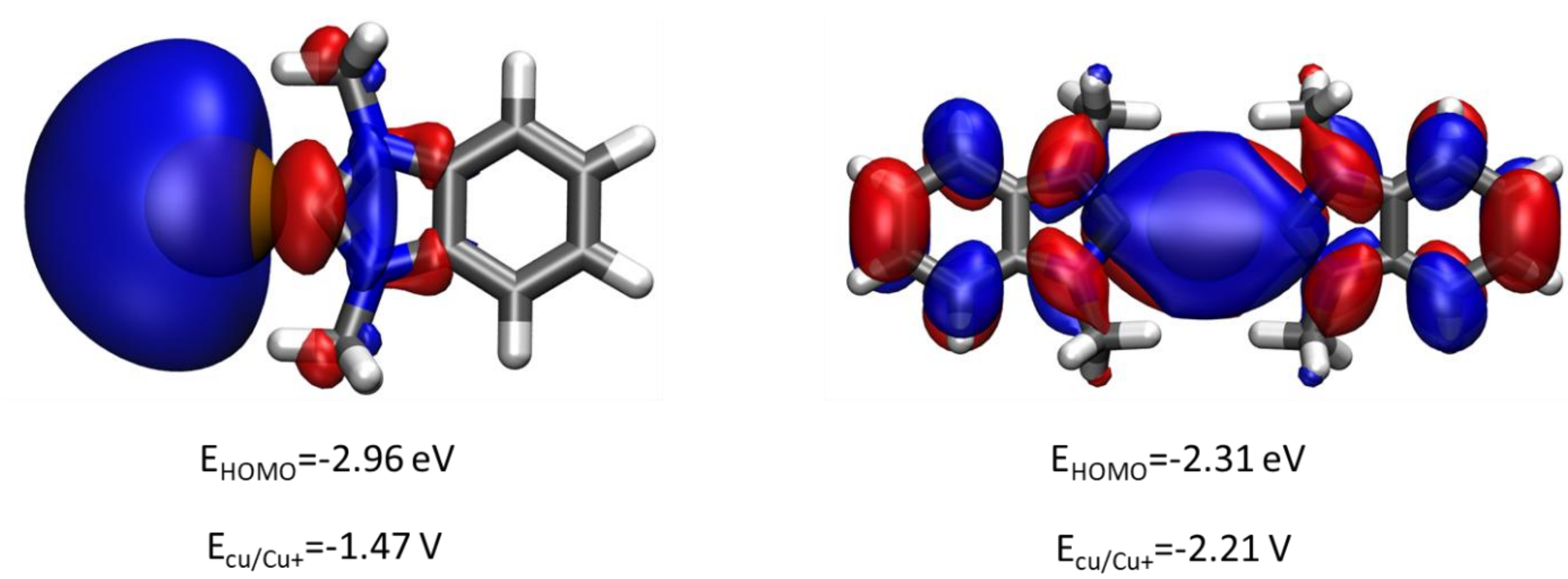

Figure S102: The singly occupied molecular orbitals (SOMO, corresponding to the α-HOMO in the unrestricted calculation) of [Cu(BIMe)] on the left and [Cu(BIMe)$_2$] on the right. Their Cu^0/Cu^I redox potentials calculated from the α-HOMO energy (relative to the standard hydrogen electrode).

Multiplying the adsorption energy per NHC with the respective theoretical coverage (molecules per nm^2) gives the surface specific adsorption energy: -7.1 eV/nm^2 for the upright geometry (species II) and -3.6 eV/nm^2 for the flat lying geometry (species III).

In summary, at sub-monolayer coverages the formation of species III from species II is energetically favored. At higher coverages (close to ideal monolayer) species II is the favored surface binding state. This matches the observation of both the experiments in the dark (Figure S62) and without air (Figure S44) as inhibited etching and long deposition times achieved high surface coverage and resulted in a favored species II.

In Table S9 theoretical XPS N 1s and Cu $2p_{3/2}$ binding energies of both complexes are listed. As all experimental XPS data in this work was referenced to Cu $2p_{3/2}$ at 932.8 eV, also the theoretical N 1s BEs were calibrated according to Cu $2p_{3/2}$. Here, the two complexes are 1.82 eV apart, which agrees very well with our experimental results (e.g., 400.7 eV and 398.8 eV are 1.9 eV apart). The theoretical N 1s BEs being generally lower than the experimental ones can be explained as follows: A) Theoretical values refer to gas phase complexes and measured values refer to solid phase, adsorbed complexes. B) Theoretical XPS BEs refer to the onset of an XPS peak without any broadening, whereas experimental XPS peaks are broadened due to kinetic energy loss of photoelectrons prior to their detection. If one considers our N 1s peaks presented in Section 4 being broader (e.g., FWHM = 2.0 eV) than the Cu $2p_{3/2}$ peaks (e.g., FWHM = 1.1 eV), one could argue to shift the computed N 1s BEs by 0.5-1 eV to higher BEs (lower kinetic energies). This approach would fit the experimental values even better but is quite vague and was therefore not considered.

Table S9: Theoretical XPS N 1s and Cu $2p_{3/2}$ BEs.

	N 1s [eV]	Cu $2p_{3/2}$ [eV]	Shift [eV]	Calibrated N 1s [eV]
[Cu(BIMe)]	393.539	926.448	6.352	399.891
[Cu(BIMe)$_2$]	392.714	927.443	5.357	398.071

Figure S103 shows the EPM of both complexes and agrees qualitatively with the theoretical XPS BEs, the measured XPS BEs and the redox potentials, as the N atoms are shown to be more electron rich in case of the (bis)NHC Cu^0 complex. Whereas Cu(BIMe) shows the negative electrostatic potential to be mostly localized at the Cu atom, Cu(BIMe)$_2$ shows pronounced delocalization of the negative potential across both heterocycles and the Cu center.

Further, natural population analysis (NPA) was performed (Table S10) resulting in a partial charge of -0.339 e at each Nitrogen atom of [Cu(BIMe)] and of -0.347 e at each Nitrogen atom of [Cu(BIMe)$_2$]. While the lower partial charges at the Nitrogen atoms of [Cu(BIMe)$_2$] qualitatively agree with the simulated XPS data as well as the EPM, differences are almost negligible and do not capture similar distinctions as XPS simulation or EPM. Looking at the charges at the Cu atoms in the both complexes, Cu in [Cu(BIMe)] is more negatively charged (-0.121), according to NPA. Adding a second ligand to the system shifts the partial negative charge from the Cu center to the organic ligand as indicated by the more positively charged Cu atom, and the more negative carbene C atoms. Thus, NPA supports the results from the EPM regarding the transition from localized partial negative charge at the Cu center in [Cu(BIMe)] towards delocalized partial negative charge across both ligands in [Cu(BIMe)$_2$].

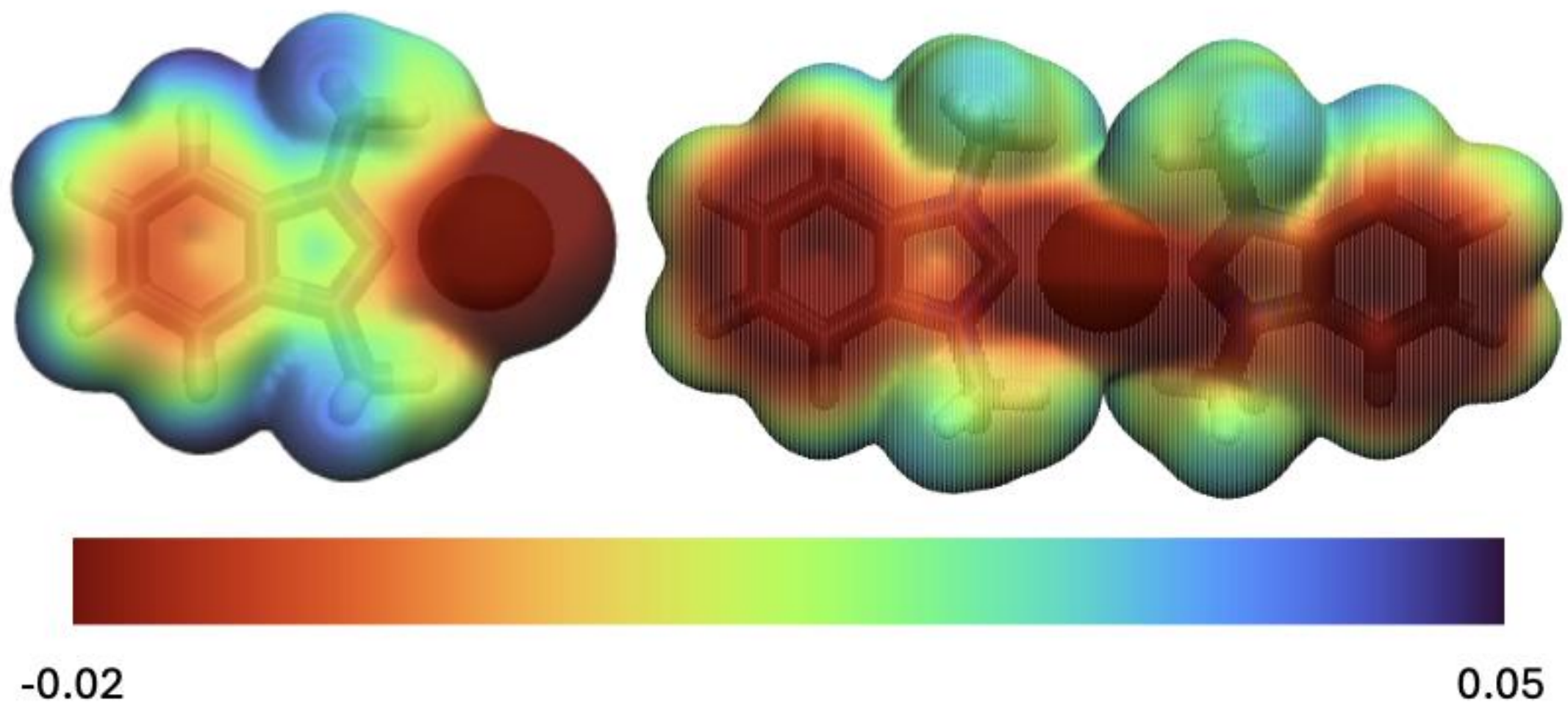

Figure S103: Electrostatic potential maps (EPM) of [Cu(BIMe)] on the left and [Cu(BIMe)$_2$] on the right. Scale: Red depicts electron rich regions (down to -0.02 atomic units) and blue depicts electron poor regions (up to 0.05 atomic units).

Tables S10. NPA charge analysis of [Cu(BIMe)] and [Cu(BIMe)$_2$] for N, Cu and carbenic C atoms (given in atomic units).

	Q N (NPA)	Q Cu (NPA)	Q C - carbene (NPA)
Cu(BIMe)	-0.339	-0.121	0.079
Cu(BIMe)$_2$	-0.347	0.218	-0.101

A Mulliken analysis of the compositions of the highest occupied orbitals (α-HOMO) reveals that in the case of [Cu(BIMe)] the orbital is highly concentrated at the Cu atom (97 %) with 87% s orbital contribution (see Table S11). In contrast, [Cu(BIMe)$_2$] shows delocalization of the α-HOMO across the π system of the ligand with p orbital contribution of 84% (Figure S102 and table S11).

Table S11. Mulliken orbital composition analysis of the α-HOMO in the molecules of interest.

	highest atomic %	highest orbital %	Orbital Delocalization Index
Cu(BIMe)	97% Cu	87% s	95.04
Cu(BIMe)$_2$	20% C (carbene)	84% p	11.12

To further understand the electronic structure of the adsorbed complexes [Cu(BIMe)] and [Cu(BIMe)$_2$], we have attempted to analyze the PDOS of the most important structures in the etching mechanism, namely the models in Figure S100 a and d. Using the lobster package, we project the DOS onto an atomic orbital basis set. Examining the total DOS, which was split into the atomic and orbital contributions (Figure S104 a, c), reveals a highly uninterpretable nature of the spectrum, due to the large amount of Cu atoms in the surface which hide the electronic

structure of the interacting complex. Therefore, we decided to split the contributions of the Cu atoms into the two categories: Cu atoms directly bound to the complex and all other atoms. Plotting only the former mentioned atoms, a totally different spectrum is revealed (Figure S104 b, d), showing the expected spikes for the molecular states, while the Cu atoms have only the d-states contributing mostly. Nonetheless, very little information can be collected about the possible orbital interactions in the Fermi energy area, due to small contributions from the Cu atoms. In contrast, both spectra reveal good overlap in the -2 eV region for both complexes, with [Cu(BIMe)$_2$] showing a better overlap of Cu d-states with the organic p-states than [Cu(BIMe)]. This overlap between Cu d-states and organic p-states in PDOS simulations is yet another indication for substantial electron delocalization in [Cu(BIMe)$_2$]. This result supports the interpretation of adsorbed [Cu(BIMe)$_2$] as formal Cu^+ with the negative charge being delocalized across both ligands, which agrees well with the Mulliken orbital composition of the α-HOMO, the EPM, the NPA charge analysis and both theoretical and practical XPS.

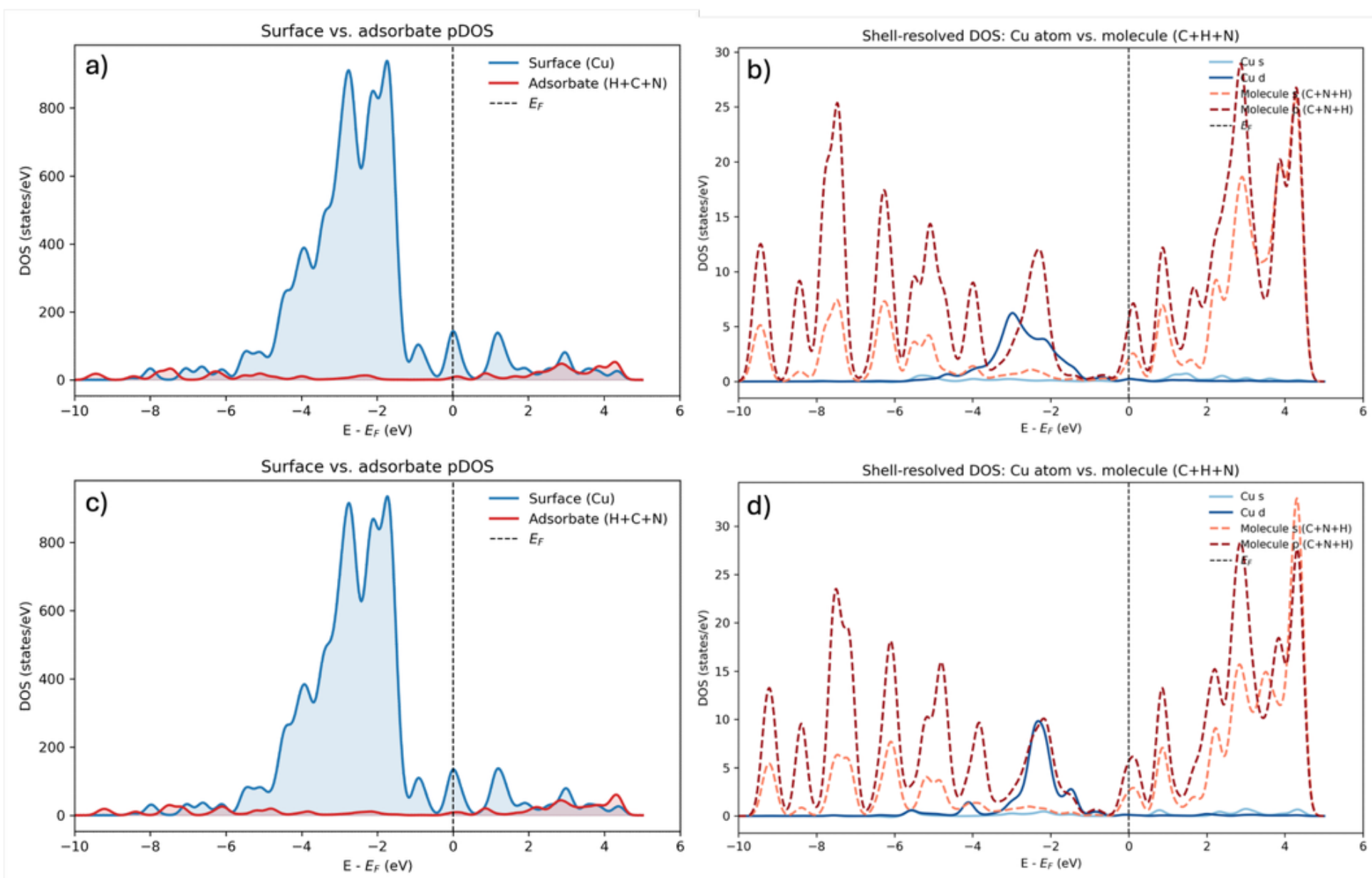

Figure S104. PDOS analysis of [Cu(BIMe)] (a, b) and [Cu(BIMe)$_2$] (c, d).

15.1 Coordinates of optimized geometries

VASP optimized coordinates:
Species from **Figure S100 a)**

```
234
Generated with con2xyz
Cu   10.22973634280144   0.67131586652722   5.08728658449724
Cu   1.28348144762368   8.80137133087048   0.90370000000011
Cu   1.28568227792491   10.27631524173066   3.01640541105490
Cu   10.22981644228912   2.15983268780656   0.90370000000011
Cu   10.25367713906027   3.63121665598981   3.05766441944757
Cu   8.94294027149689   2.89699992853354   5.05772052873164
Cu   0.00653585769462   9.53560796468316   5.12239500734852
Cu   1.28495118830118   2.89701725840631   5.13073104414892
Cu   8.95154948948265   4.37350180863156   0.90265000000002
Cu   8.96156322821266   5.87197426168445   3.06773326229103
Cu   7.67411529528458   5.09406642094461   5.07392863737401
Cu   -5.10580841645604   15.44273288040492   0.90125000000008
Cu   0.00582796479926   11.01539463875492   0.90335000000000
Cu   7.67348702667015   6.58787930357520   0.90335000000000
Cu   7.70340403776502   8.04977449517052   3.06762787313566
Cu   6.39625500860128   7.32431556874938   5.08018112161294
Cu   0.00703805924353   12.48982868842775   3.01572412246952
Cu   -1.27400508295449   11.75151616846587   5.12195880203463
Cu   6.39562905385170   8.80119423734088   0.90335000000000
Cu   6.40211566103832   10.26884138580457   3.02236275917359
Cu   5.12496256267352   9.53157818956718   5.13183798733175
Cu   -5.10733917289367   16.91690257963065   3.01358261490843
Cu   -6.38440334125068   16.17833324154532   5.12870344962460
Cu   5.11715761105111   11.01486335816588   0.90265000000002
Cu   5.12147031411723   12.48752608406211   3.01684566327710
Cu   3.84442516413683   11.74942305818474   5.13250049342506
Cu   -1.27243898800736   13.22906375957992   0.90370000000011
Cu   -1.27134193087295   14.70164206510444   3.01351164584996
Cu   3.83889065824457   13.22888666605020   0.90370000000011
Cu   3.84080700697012   14.70366200881822   3.01778603124254
Cu   2.56466830188514   13.96416232439691   5.13266674919369
Cu   -2.55232204668666   13.96360096189153   5.12243044067647
Cu   2.56093044042913   15.44237869334548   0.90404999999997
Cu   2.55861706002907   16.92149793835652   3.02053493774517
Cu   1.28373594489880   16.17983061508311   5.13056639059167
Cu   -2.55019471582868   15.44255578687520   0.90370000000011
Cu   1.28307246761067   17.65569362711116   0.90404999999997
Cu   11.51272295283955   1.45425010280318   3.07522347250431
Cu   12.78754327152880   0.68233064198616   5.13925413404534
Cu   -2.54932932976529   16.91012821624843   3.00302839763375
Cu   -3.83062150567348   16.17827666763858   5.12630867479039
Cu   12.78563463291059   2.15965559427684   0.90370000000011
Cu   12.76470440601140   3.62235763219273   3.04489897341414
Cu   11.50768816772866   2.88599702500076   5.71639567977101
Cu   1.28317471264508   4.37385599569088   0.90544999999992
Cu   11.50757217009817   4.37367890216116   0.90125000000008
Cu   11.49838461883963   5.84220663907703   2.99663936397280
Cu   10.23868755901768   5.10341768041652   5.13821068570160
Cu   -3.82825717864111   17.65657909475952   0.90335000000000
Cu   6.39331227577935   1.42155033840939   3.00033706635323
```

Cu	10.22899848230045	6.58929605181247	0.90335000000000
Cu	10.20732987325493	8.05184160076602	3.05142944435683
Cu	8.96108876223756	7.29726955096644	5.74373569355396
Cu	7.66442352756754	0.67400376089295	5.08592450578078
Cu	1.28340303115435	5.84675293168321	3.01609719996102
Cu	8.95134499947611	8.80119423734088	0.90440000000008
Cu	8.94373184083330	10.27528094225507	3.01568474400744
Cu	7.68316893503693	9.52380605889392	5.14619829680330
Cu	0.00477317995854	5.10987437597340	5.12994664720891
Cu	7.67379376167375	2.16000978133616	0.90370000000011
Cu	7.67502070161293	11.01362370345820	0.90404999999997
Cu	7.67148410629666	12.48589213992396	3.01897712829482
Cu	6.39809054165325	11.74508349909365	5.13771871145437
Cu	7.67613586630467	3.63640561940760	2.98583513830558
Cu	6.38928803165711	2.89109139337575	5.10856846877277
Cu	6.39501558385700	13.22853247899088	0.90055000000010
Cu	6.39553797902381	14.70095888603574	3.01457645534396
Cu	5.11889918944827	13.96132871715024	5.13206126216744
Cu	-3.82917738360190	13.22870957252048	0.90195000000005
Cu	-3.83182348650360	14.70283869088506	3.01433809775943
Cu	5.11674863105053	15.44220159981588	0.90370000000011
Cu	5.11423706173026	16.91329346154973	3.01935509336412
Cu	3.83963845413899	16.17564877782106	5.13729665754150
Cu	6.39583354385823	4.37350180863156	0.90404999999997
Cu	6.40124744742495	5.85131638748069	2.99772375263630
Cu	3.83940188321714	17.65569362711116	0.90370000000011
Cu	14.06015383001809	1.41039630488508	3.00617706464700
Cu	15.33854251317532	0.68065420799076	5.13124229186236
Cu	5.11035946175093	5.10157275311060	5.08641807466639
Cu	0.00531673982669	6.58752511651588	0.90370000000011
Cu	15.34196404851714	2.15930140721752	0.90299999999988
Cu	15.33852867865144	3.63377753333967	2.99391891752222
Cu	14.06428750915371	2.88236122567099	5.06490282229617
Cu	2.56322946161833	0.68350770660710	5.12939273186559
Cu	5.11664638607854	6.58699383592684	0.90229999999991
Cu	14.06349260571656	4.37226215392388	0.90089999999996
Cu	14.06180216372667	5.84731348928190	2.99069317536541
Cu	12.78081170437918	5.11728163876211	5.06957784293965
Cu	5.12120390837829	8.06461266864344	3.00219407030339
Cu	3.84262769706319	7.32099354616629	5.11757597507063
Cu	12.78543014290405	6.58982733240151	0.90159999999994
Cu	12.78527230912565	8.06126514726949	2.99248927381718
Cu	11.51283506218079	7.30938683297406	5.05840209673240
Cu	-6.38468883924485	17.65640200122992	0.90335000000000
Cu	3.83692323406308	1.41932851427783	3.01465277173247
Cu	11.50695870010339	8.80491320146391	0.90825000000006
Cu	11.50211555356373	10.27181560806785	3.00697369127509
Cu	10.22012797556006	9.53585829943891	5.09571310894561
Cu	3.83919739324809	8.80172551792992	0.90404999999997
Cu	3.84489566127484	10.27692060191224	3.01660714768072
Cu	10.23247481218681	11.01238404875052	0.90895000000003
Cu	10.22733986126108	12.48582825574090	3.01632571272943
Cu	8.94987457133336	11.74344723681972	5.13564681856651
Cu	2.56682097483000	9.53610239466270	5.12565872965627
Cu	5.11700944457077	0.68130938491946	5.12769639704876
Cu	8.95277642942183	13.22782410487224	0.90229999999991
Cu	8.94891791766529	14.70057307412338	3.01295189742663
Cu	7.67150278663849	13.96100106186488	5.13077714163677

Cu	-3.83258821368188	11.74872927230191	5.12851096496599
Cu	2.56113493043560	11.01504045169548	0.90404999999997
Cu	7.67205559668686	15.44290997393452	0.90159999999994
Cu	7.67002711053177	16.91515421154686	3.01480898948899
Cu	6.39332754559433	16.17467855753725	5.13101962192952
Cu	2.56622296132313	12.49070143563499	3.01631901553803
Cu	1.28800338636299	11.75186566792159	5.12221508356140
Cu	6.39511782884163	17.65604781417048	0.90404999999997
Cu	16.61598185282914	1.42071649433575	3.01367428394877
Cu	17.89326338869094	0.68205065109919	5.12223944393324
Cu	2.56123717545758	2.16018687486588	0.90404999999997
Cu	-5.10910417461707	13.96336586292545	5.12807228592067
Cu	17.89808897412964	2.16000978133616	0.90404999999997
Cu	17.89727915874970	3.63564696607625	3.01328180319480
Cu	16.61636281299347	2.89834517301764	5.11974910961706
Cu	1.28358369260824	13.22888666605020	0.90335000000000
Cu	1.28713658798884	14.70404533842120	3.01638208974046
Cu	16.61992426632008	4.37314762157224	0.90335000000000
Cu	16.62577055750518	5.85218564049274	3.00016940744589
Cu	15.34433829467768	5.11326906334032	5.07912031846857
Cu	0.00697418819252	13.96557529255941	5.12516140560686
Cu	2.56360184189376	5.10852208418984	5.12769434696459
Cu	15.34237302849272	6.58734802298616	0.90019999999999
Cu	15.34469955526917	8.06297980846119	3.00647108278544
Cu	14.06946630307133	7.32394933784363	5.11012374344511
Cu	0.00516991782868	8.06064740520926	3.01611886595438
Cu	0.00552122979574	15.44255578687520	0.90370000000011
Cu	14.06400383068912	8.80084005028156	0.90159999999994
Cu	14.06828319085484	10.27826476700392	3.00356293323004
Cu	12.78894895066729	9.53741873983873	5.08619678248285
Cu	0.01026378804915	16.91624842877064	3.00381215226705
Cu	-1.27406471728344	16.17747647376347	5.12097178858466
Cu	12.78174932299860	11.01220695522093	0.90789999999995
Cu	12.78403594818209	12.48706595364928	3.01517949552454
Cu	11.50662235667912	11.74400170415131	5.12833334395356
Cu	5.11756659106419	2.16018687486588	0.90370000000011
Cu	11.50777666006714	13.22835538546116	0.90195000000005
Cu	11.50560696094608	14.70138591427050	3.01296384869798
Cu	10.22674485567173	13.95996172551247	5.12223356541078
Cu	-1.27274572301080	17.65587072064088	0.90335000000000
Cu	8.95804234561087	1.41999602606082	2.99583091025210
Cu	10.22910072727251	15.44273288040492	0.90125000000008
Cu	10.22715669834670	16.91561038805009	3.01498992289315
Cu	8.94705244031759	16.17473191846465	5.12348833071819
Cu	8.95062928447193	17.65604781417048	0.90370000000011
Cu	19.17356509641510	1.42080244024585	3.01513390210942
Cu	0.00293584380413	0.68362442705384	5.12236182708645
Cu	-1.27501087643786	7.32209665491987	5.12940079382328
Cu	0.00511224984508	2.16018687486588	0.90404999999997
Cu	0.00576052452628	3.63370173681350	3.01805792940198
Cu	-1.27538719687275	2.89766038553317	5.12334510722429
Cu	2.56133942044221	6.58770221004548	0.90370000000011
Cu	2.56176576966200	8.06368553290084	3.01275907905398
Cu	-1.27284796797029	4.37403308922048	0.90370000000011
Cu	-1.27083534807609	5.84849141845865	3.01800501945111
Cu	-2.55550480960487	5.11228683952438	5.12785308981835
Cu	-1.27203000800663	8.80172551792992	0.90299999999988
Cu	-1.27292176748267	10.27586144536226	3.01425233526166

Cu	-2.54968349081861	6.58663964886752	0.90125000000008
Cu	-2.54915729672843	8.06268216495646	3.01506838451784
Cu	-3.83011245056246	7.32257210243262	5.12758847871082
Cu	-2.55458155542977	9.53508353969745	5.12722774634695
Cu	5.11657429351721	3.63101429090631	2.99910086702006
Cu	-3.82846166861030	8.80154842440020	0.90089999999996
Cu	-3.82816110089490	10.27679906365902	3.01485213313744
Cu	-5.10896318109769	9.53533966052518	5.12772745675405
Cu	3.83925706834846	2.89514906715194	5.12877871969618
Cu	2.56199287916724	3.63427415195822	3.01551197631216
Cu	-5.10948923633635	11.01397789051752	0.90299999999988
Cu	-5.10789204344701	12.49046766738160	3.01671664783271
Cu	-6.38627093537535	11.74534829958244	5.12923063100991
Cu	1.28477795153052	7.32292593483370	5.12385340657152
Cu	3.83929963825765	4.37421018275020	0.90370000000011
Cu	-6.38632475918468	13.22729282428320	0.90195000000005
Cu	-6.38705172807159	14.70372552894858	3.01478028779228
Cu	-7.66433660669761	13.96147295492305	5.12216998271605
Cu	3.83684702580162	5.85199750996932	3.00370593573588
Cu	-7.66305803703581	15.44290997393452	0.90019999999999
Cu	-7.66334077172400	16.91584238578357	3.01617277649647
Cu	-8.94188680191163	16.17545717971704	5.12167996039876
Cu	-2.54988798082509	11.01574882581424	0.90125000000008
Cu	-2.55319140721920	12.48956641326422	3.01470713593600
Cu	-8.94163172483352	17.65711037534856	0.90229999999991
Cu	1.28252067490564	1.42172196043382	3.01780103963486
N	8.02531449894741	6.74060800833731	8.59855215068792
N	10.01421554357927	7.63931652824910	8.59544847643038
N	10.34527514154494	2.78102669218397	8.54282593743918
N	12.32441680915871	3.69994063884435	8.54699960460570
H	6.08923520362599	6.94831748266896	7.76560531045468
H	6.90176519423863	5.39098256070072	7.43560846182019
H	11.28956930112280	8.29932578999942	11.13801686750695
H	6.69662308334216	6.21791949203311	11.14782928517518
H	10.09969265593769	7.82558118066105	13.26831190132975
H	7.84503713614176	6.80314067145174	13.27294645220176
H	6.25536016527987	5.72596067617721	9.06264043768596
H	11.74776597203188	7.60939151961608	7.40273815061335
H	11.14289970461214	9.24542931113342	7.79767209130367
H	11.96082000639927	8.25702894213824	9.04848457880356
H	8.33616214358713	2.70428221245106	7.85983195609760
H	9.38010525406707	1.34047466465286	7.36635698510964
H	13.36240101142775	4.85044722399481	11.01864167591281
H	8.78152900817019	2.73289416644180	11.00125211742631
H	11.98344563021291	4.74976763059321	13.09129947337974
H	9.73742249861199	3.71431341780590	13.08264574529237
H	8.81573076459365	1.44762945456276	9.05124232844402
H	14.06392534395211	3.49217688688927	7.39533905857998
H	13.67145066351292	5.17948549082515	7.83415199661966
H	14.32017898672397	4.04529570156897	9.06756848667191
C	9.01691821761742	7.19296862494057	7.75432293533568
C	8.37111548704720	6.92271814501852	9.93893174536602
C	9.65189211636577	7.50232075519652	9.93674696761731
C	9.62680766006375	7.57207260516387	12.32019989112127
C	6.74404176443990	6.17328752408863	8.19051453483161
C	10.30320556119260	7.83908157887991	11.12857035875798
C	8.34409169556848	6.98997047827626	12.32278563369432

```
C   7.69315640731125   6.65546577703584   11.13374778703461
C   11.28554484187343   8.22671501317265   8.18625082057952
C   11.41271201933136   3.07646513175382   7.72108850673233
C   10.55546779369249   3.24500609070716   9.84125631551067
C   11.82994827330608   3.83560628598148   9.84474466618693
C   11.59520805806381   4.32864497308571   12.16462899693738
C   9.14614731652514   2.03244415437872   8.18330415917062
C   12.37566130208224   4.39026237993220   11.00734697767857
C   10.31636234807199   3.73816903843923   12.16024578846782
C   9.77159819404024   3.18614358032271   10.99828879616641
C   13.66942940228120   4.13753853079346   8.18909368798417
```

Species from **Figure S100 b)**

```
234
Generated with con2xyz
Cu   10.22960353395594   0.66912051743587   5.09150479945521
Cu   1.28348144762368   8.80137133087048   0.90370000000011
Cu   1.29261289465688   10.27455774917227   3.01941900755696
Cu   10.22981644228912   2.15983268780656   0.90370000000011
Cu   10.25642791783212   3.62471165828554   3.05596217585195
Cu   8.94696734606824   2.90186376248413   5.06788282940512
Cu   0.00250467472907   9.53867521577704   5.13096445824432
Cu   1.28618552712949   2.89918212381143   5.13292716673612
Cu   8.95154948948265   4.37350180863156   0.90265000000002
Cu   8.94455987099217   5.85795280856768   2.99344360472933
Cu   7.67694157891174   5.11966706764322   5.13857377386816
Cu   -5.10580841645604   15.44273288040492   0.90125000000008
Cu   0.00582796479926   11.01539463875492   0.90335000000000
Cu   7.67348702667015   6.58787930357520   0.90335000000000
Cu   8.95448550962954   7.31908894389213   5.04384479365056
Cu   6.40288952785061   7.33085736684458   5.12316278837161
Cu   0.00905453697742   12.48719210561307   3.01636935366783
Cu   -1.27426755434229   11.75073451662214   5.12745775269281
Cu   6.39562905385170   8.80119423734088   0.90335000000000
Cu   6.41102845169063   10.25304987189875   3.00789334953266
Cu   5.12705254519596   9.53340107476134   5.14232735928298
Cu   -5.10665423511725   16.92318051738173   3.01521241809177
Cu   -6.38605752155749   16.18287758104111   5.12950852990227
Cu   5.11715761105111   11.01486335816588   0.90265000000002
Cu   5.11749238217561   12.48707327734724   3.01298221681797
Cu   3.84223076176122   11.75431770237281   5.13584129138254
Cu   -1.27243898800736   13.22906375957992   0.90370000000011
Cu   -1.27169947174191   14.70142167820302   3.01335970789398
Cu   3.83889065824457   13.22888666605020   0.90370000000011
Cu   3.84015065750540   14.70163499285886   3.01770532026172
Cu   2.56008150115282   13.96694115587941   5.13781932444568
Cu   -2.55175535594592   13.96382437600608   5.11961233864785
Cu   2.56093044042913   15.44237869334548   0.90404999999997
Cu   2.55714903886697   16.92206733089717   3.01951187343304
Cu   1.28571372855192   16.17960778650219   5.13176188707955
Cu   -2.55019471582868   15.44255578687520   0.90370000000011
Cu   1.28307246761067   17.65569362711116   0.90404999999997
Cu   11.51443394877215   1.45495909353637   3.07940320322228
Cu   12.79044903027473   0.68255040775847   5.14299385594932
Cu   -2.54900658440773   16.91616568887387   3.00416527351586
Cu   -3.83095722726755   16.18011077026641   5.12700898829915
Cu   12.78563463291059   2.15965559427684   0.90370000000011
```

Cu	12.75962937251878	3.63264016138959	3.05062907937700
Cu	11.50469689974433	2.88723422969665	5.71009659483406
Cu	1.28317471264508	4.37385599569088	0.90544999999992
Cu	11.50757217009817	4.37367890216116	0.90125000000008
Cu	11.48324855519991	5.85998074680885	3.00534393317644
Cu	10.23159181539185	5.10922943785284	5.11676000547294
Cu	-3.82825717864111	17.65657909475952	0.90335000000000
Cu	6.39518858228958	1.43472483503512	3.00760877286134
Cu	10.22899848230045	6.58929605181247	0.90335000000000
Cu	10.20395440638465	8.06092675146172	2.99019974554471
Cu	7.79318240828724	8.00667677511216	7.05777420728821
Cu	7.66466319976986	0.67649584774444	5.09065743201359
Cu	1.29669566212287	5.85073310970839	3.01886128479202
Cu	8.95134499947611	8.80119423734088	0.90440000000008
Cu	8.93245707225573	10.24711411303018	3.00710116590498
Cu	7.66955991674650	9.52929286294739	5.11475419559380
Cu	0.00760609166653	5.11021813858092	5.12938313919072
Cu	7.67379376167375	2.16000978133616	0.90370000000011
Cu	7.67522519160704	11.01362370345820	0.90404999999997
Cu	7.67337815166162	12.46647471491591	3.01423038753894
Cu	6.39613450695061	11.74555114231434	5.11382103497870
Cu	7.67230779204615	3.66017494571921	3.00796663378706
Cu	6.39171968235359	2.90020464087847	5.12483133349692
Cu	6.39501558385700	13.22853247899088	0.90055000000010
Cu	6.39861185180435	14.69075388391963	3.01665834938826
Cu	5.11888271634050	13.96092843472467	5.13140513772838
Cu	-3.82917738360190	13.22870957252048	0.90195000000005
Cu	-3.83118981928924	14.70273193649487	3.01458403663237
Cu	5.11674863105053	15.44220159981588	0.90370000000011
Cu	5.11595748267342	16.91066888294607	3.01702801457229
Cu	3.84179593146206	16.17582936748277	5.13266982830611
Cu	6.39583354385823	4.37350180863156	0.90404999999997
Cu	6.41637361334126	5.88348257468140	3.00914838575159
Cu	3.83940188321714	17.65569362711116	0.90370000000011
Cu	14.05758239667670	1.41403266324659	3.00946143656341
Cu	15.34141573699608	0.68037378943900	5.12980318838034
Cu	5.12694602443283	5.11530866442328	5.11198582667853
Cu	0.00531673982669	6.58752511651588	0.90370000000011
Cu	15.34196404851714	2.15930140721752	0.90299999999988
Cu	15.33537820568017	3.63788375270584	2.99502833464765
Cu	14.06464316511878	2.88533447837401	5.06672309421469
Cu	2.56286749527210	0.68772850622542	5.13166117638045
Cu	5.11664638607854	6.58699383592684	0.90229999999991
Cu	14.06349260571656	4.37226215392388	0.90089999999996
Cu	14.05132638224048	5.85132052654128	2.99820565374996
Cu	12.77468422930807	5.12294270192901	5.07872975069825
Cu	5.15229776924814	8.06298298276885	3.00827851345685
Cu	3.84884996576690	7.32644590488039	5.10847530148821
Cu	12.78543014290405	6.58982733240151	0.90159999999994
Cu	12.78733518880463	8.06473815485707	3.00924487559506
Cu	11.50566451136488	7.32670337670661	5.10624005111626
Cu	-6.38468883924485	17.65640200122992	0.90335000000000
Cu	3.84023230853310	1.42531262706877	3.01858296165978
Cu	11.50695870010339	8.80491320146391	0.90825000000006
Cu	11.48967418092082	10.26484056966163	3.02358347686641
Cu	10.21966366346423	9.52946625875376	5.12314301646814
Cu	3.83919739324809	8.80172551792992	0.90404999999997
Cu	3.86212836883397	10.26554956076314	3.02483883409938

Cu	10.23247481218681	11.01238404875052	0.90895000000003
Cu	10.23018535490891	12.48453706852250	3.01365650933527
Cu	8.95086506944946	11.74147497173842	5.11311180774812
Cu	2.56336319097902	9.53657030993686	5.13125051380517
Cu	5.11749831334812	0.68354872606321	5.13683452814715
Cu	8.95277642942183	13.22782410487224	0.90229999999991
Cu	8.94753996444877	14.69221332885615	3.01662277082512
Cu	7.67253935067805	13.95939317071950	5.13037620365064
Cu	-3.83145971319397	11.74799346317812	5.13099109819791
Cu	2.56113493043560	11.01504045169548	0.90404999999997
Cu	7.67205559668686	15.44290997393452	0.90159999999994
Cu	7.67160592277051	16.91054628780059	3.01306570835272
Cu	6.39578209757462	16.17624809581542	5.12762427110685
Cu	2.56910644001859	12.48456683515520	3.01923374585827
Cu	1.28435230080382	11.75345672868456	5.13018287793957
Cu	6.39511782884163	17.65604781417048	0.90404999999997
Cu	16.61820269256641	1.42119990389460	3.01209551422413
Cu	17.89694776128976	0.68154174024354	5.11968307859013
Cu	2.56123717545758	2.16018687486588	0.90404999999997
Cu	-5.10907408143652	13.96533567620369	5.12757817132883
Cu	17.89808897412964	2.16000978133616	0.90404999999997
Cu	17.89942109845175	3.63616688352109	3.01174451812556
Cu	16.61881902787924	2.89934464742589	5.11332029867824
Cu	1.28378818260220	13.22888666605020	0.90335000000000
Cu	1.28801611134760	14.70249032672857	3.01604285064641
Cu	16.61992426632008	4.37314762157224	0.90335000000000
Cu	16.62301456515078	5.85467238248930	3.00345537755963
Cu	15.34339784910443	5.11444840781673	5.08221574374331
Cu	0.00713459200262	13.96612489253668	5.12878065837170
Cu	2.57008116150478	5.11268174864340	5.13162175466663
Cu	15.34237302849272	6.58734802298616	0.90019999999999
Cu	15.34082437722640	8.06248142675811	3.01455672916603
Cu	14.06098134760294	7.32571180185712	5.12820976284100
Cu	0.00892162614916	8.06237567596488	3.01807865889267
Cu	0.00552122979574	15.44255578687520	0.90370000000011
Cu	14.06400383068912	8.80084005028156	0.90159999999994
Cu	14.05612881124553	10.27343763309358	3.02010677030548
Cu	12.78424810351117	9.53309593039298	5.13767720277768
Cu	0.00993872694355	16.92071339232898	3.00495541668021
Cu	-1.27327532332786	16.17740606416976	5.12370114957428
Cu	12.78174932299860	11.01220695522093	0.90789999999995
Cu	12.77910769479352	12.48369431569406	3.02052156179839
Cu	11.50457148029643	11.74587627354883	5.13649615150474
Cu	5.11756659106419	2.16018687486588	0.90370000000011
Cu	11.50777666006714	13.22835538546116	0.90195000000005
Cu	11.50527536626507	14.70086562844309	3.01628830497342
Cu	10.22699145270581	13.95896219821975	5.12489337751663
Cu	-1.27274572301080	17.65587072064088	0.90335000000000
Cu	8.95497431055958	1.42855074916293	2.99983295669617
Cu	10.22910072727251	15.44273288040492	0.90125000000008
Cu	10.22828153201610	16.91363826892309	3.01657834771412
Cu	8.94876549076412	16.17788888174765	5.12497928645949
Cu	8.95062928447193	17.65604781417048	0.90370000000011
Cu	19.17538343401029	1.42208521233732	3.01444446162362
Cu	0.00557475268957	0.68487748143341	5.13050182405698
Cu	-1.27345370552068	7.32191135837362	5.12797284307072
Cu	0.00511224984508	2.16018687486588	0.90404999999997
Cu	0.01265128628195	3.63726184036615	3.01682685342176

Cu	-1.27078686303766	2.89915061422155	5.12138083802683
Cu	2.56133942044221	6.58770221004548	0.90370000000011
Cu	2.56567891682945	8.06561324563953	3.01271337938985
Cu	-1.27284796797029	4.37403308922048	0.90370000000011
Cu	-1.26610649982836	5.85031285725936	3.01801559963213
Cu	-2.55269019987231	5.11293610900148	5.12906804507301
Cu	-1.27203000800663	8.80172551792992	0.90299999999988
Cu	-1.27094417434543	10.27425993025359	3.01635250851782
Cu	-2.54968349081861	6.58663964886752	0.90125000000008
Cu	-2.55010560331834	8.06399053667341	3.01558628723403
Cu	-3.83177069192845	7.32142404730991	5.13066915436214
Cu	-2.55387549790632	9.53356269769300	5.13235868297196
Cu	5.11966579297757	3.63922546785927	3.00871371757807
Cu	-3.82846166861030	8.80154842440020	0.90089999999996
Cu	-3.83370663988565	10.27361530973723	3.01663313882478
Cu	-5.10630575751762	9.53613976489323	5.13841101326180
Cu	3.84418720940569	2.90247084385630	5.13364027756484
Cu	2.56961915715451	3.64269724472659	3.01978370493266
Cu	-5.10948923633635	11.01397789051752	0.90299999999988
Cu	-5.11197382531966	12.48592667940081	3.01726486870516
Cu	-6.38703392265349	11.74706259366047	5.13494664171720
Cu	1.28897111631006	7.32458372315828	5.12649065840594
Cu	3.83929963825765	4.37421018275020	0.90370000000011
Cu	-6.38632475918468	13.22729282428320	0.90195000000005
Cu	-6.38979438628710	14.70308250628824	3.01478904854919
Cu	-7.66218222550009	13.96525279086419	5.13038142567475
Cu	3.86068949186447	5.85992567372959	3.01287248100072
Cu	-7.66305803703581	15.44290997393452	0.90019999999999
Cu	-7.66340631794452	16.91901215644871	3.01535773780949
Cu	-8.94309629836821	16.17968933788143	5.12449813012977
Cu	-2.54988798082509	11.01574882581424	0.90125000000008
Cu	-2.55372472893442	12.48926453537294	3.01539100160388
Cu	-8.94163172483352	17.65711037534856	0.90229999999991
Cu	1.28717900528974	1.42592824056385	3.02029835085251
N	7.34450352249128	7.16141481043556	9.85824310879248
N	9.25801587470672	8.19124885212133	9.62541010837175
N	10.34925381908614	2.75913255446739	8.54177669355749
N	12.32294088756192	3.68842632758935	8.54642002938800
H	5.25755717361097	7.06777966496267	10.15360814917849
H	5.82942878334883	6.71658265510448	8.49305493208666
H	10.99981565192673	8.51911981691597	11.93127648803791
H	6.57131246459403	6.15363713153461	12.48021091437986
H	10.27046614079687	7.64760688903296	14.15566162064814
H	8.10227151877794	6.49115993790589	14.42400767373208
H	6.04754312596676	5.49936076240405	9.78577881895595
H	10.17124351617876	9.10581470346079	7.98406523945458
H	10.54140381763472	9.86410791401828	9.56222903072669
H	11.30284417964995	8.30141666916383	9.10908048417597
H	8.32203938302528	2.66511938270807	7.92270535488614
H	9.37852540169189	1.34533202619150	7.34038732331164
H	13.32386737159677	4.90816284675495	10.99938385871977
H	8.75748780996288	2.75836253762928	10.98030609053687
H	11.91411551059671	4.86277175676995	13.05480814755499
H	9.67565351704582	3.81289120709757	13.04564083580358
H	8.86750953280417	1.37086124682133	9.04533789683182
H	14.05663355413449	3.49675258737528	7.38574334074046
H	13.67458454253994	5.17678331210750	7.86479496440768
H	14.32205548647261	4.01018560409386	9.06917275086485

C   8.13865735485970   7.79726766967486   8.93928512483064
C   7.94380118443469   7.15453276670016   11.11814061595457
C   9.17911435599877   7.81719873606944   10.96678265292437
C   9.62772468567704   7.51432753977782   13.28610476491304
C   6.03913934987374   6.57503494538025   9.55890550823437
C   10.04558266804498   8.00688275383890   12.04614806453689
C   8.39245769306615   6.85433225334644   13.43866578856918
C   7.52754421207620   6.65988740924080   12.35696920722312
C   10.38911111524528   8.90965868483704   9.03924028900227
C   11.42028910129812   3.04916438601830   7.72269470156810
C   10.54321590310641   3.25222363332937   9.83116910637939
C   11.81393618469094   3.85029716867179   9.83502882767293
C   11.54257942224768   4.41064444184208   12.13597754777899
C   9.15564655821664   1.99824455663466   8.19146268051118
C   12.34018183148934   4.44179146138716   10.98854198821206
C   10.26774068517836   3.81131624005798   12.13148008804590
C   9.74356681684584   3.22038667202669   10.97864963819533
C   13.66914094233235   4.12685376700408   8.19475586402263

Species from **Figure S100 c)**

234
Generated with con2xyz
Cu   10.22754724442588   0.66307549775268   5.08299690465969
Cu   1.28348144762368   8.80137133087048   0.90370000000011
Cu   1.29001882843034   10.27452026959007   3.01517442643402
Cu   10.22981644228912   2.15983268780656   0.90370000000011
Cu   10.25477696920518   3.62798370989486   3.06156149184442
Cu   8.94463352190443   2.89737151194105   5.07138942302667
Cu   0.00585250752713   9.53846090735608   5.13189332734788
Cu   1.28512956029246   2.89782435460703   5.13413470770868
Cu   8.95154948948265   4.37350180863156   0.90265000000002
Cu   8.93655804149669   5.87373628441135   3.02490139126967
Cu   7.67559889769867   5.10874209436540   5.14861118452323
Cu   -5.10580841645604   15.44273288040492   0.90125000000008
Cu   0.00582796479926   11.01539463875492   0.90335000000000
Cu   7.67348702667015   6.58787930357520   0.90335000000000
Cu   8.94681717925912   7.30868996449220   5.20117747242183
Cu   6.41168693963092   7.30661872968650   5.13921134388615
Cu   0.00834352054341   12.48665603441088   3.01676339886725
Cu   -1.27152628509955   11.74865255598683   5.12808256957522
Cu   6.39562905385170   8.80119423734088   0.90335000000000

Cu   6.41172023137697   10.25722330314467   2.98132433529502
Cu   5.13139411055015   9.53201427196956   5.08148862664528
Cu   -5.10594038726139   16.92134993654480   3.01538198850971
Cu   -6.38665172074737   16.18011921759182   5.13033487565432
Cu   5.11715761105111   11.01486335816588   0.90265000000002
Cu   5.11272320577894   12.49244395581464   3.00597684187206
Cu   3.84473852598180   11.75145687515944   5.12685739776492
Cu   -1.27243898800736   13.22906375957992   0.90370000000011
Cu   -1.27154685332694   14.69890274249500   3.01337561728186
Cu   3.83889065824457   13.22888666605020   0.90370000000011
Cu   3.83663247892111   14.70557568060461   3.01669921434391
Cu   2.55956608247970   13.96583355843334   5.13597765497920
Cu   -2.55127758405181   13.96126317757988   5.12093667354148
Cu   2.56093044042913   15.44237869334548   0.90404999999997
Cu   2.55488248232179   16.92444645115364   3.01956382789429
Cu   1.28671185231338   16.17687699283164   5.13445680173640

Cu	-2.55019471582868	15.44255578687520	0.90370000000011
Cu	1.28307246761067	17.65569362711116	0.90404999999997
Cu	11.51390818760828	1.45838146632344	3.07953728843663
Cu	12.79301035534024	0.67643797911953	5.14391008025683
Cu	-2.54849079949955	16.91371385138810	3.00334304311717
Cu	-3.83144360435518	16.17581186251957	5.12770918957573
Cu	12.78563463291059	2.15965559427684	0.90370000000011
Cu	12.75865892057911	3.63370207146276	3.05024733202010
Cu	11.50633906381553	2.88250310137348	5.67999211097134
Cu	1.28317471264508	4.37385599569088	0.90544999999992
Cu	11.50757217009817	4.37367890216116	0.90125000000008
Cu	11.47830284607559	5.86168123018146	3.00985069544619
Cu	10.23144638354775	5.10701344427503	5.12707591118842
Cu	-3.82825717864111	17.65657909475952	0.90335000000000
Cu	6.39544223516706	1.43261469583539	3.00489089575398
Cu	10.22899848230045	6.58929605181247	0.90335000000000
Cu	10.17706769968109	8.05308266157360	3.02417334877306
Cu	7.62553178542923	8.23449612095219	7.11282751120091
Cu	7.66138493853320	0.67167314656276	5.08531855017890
Cu	1.29604485396436	5.84969958973204	3.01827312289733
Cu	8.95134499947611	8.80119423734088	0.90440000000008
Cu	8.94376894855393	10.25544757311813	2.98383719074614
Cu	7.68119693689908	9.52034865474064	5.02850365527302
Cu	0.00827794639638	5.11051843312886	5.12890694975345
Cu	7.67379376167375	2.16000978133616	0.90370000000011
Cu	7.67502070161293	11.01362370345820	0.90404999999997
Cu	7.67259026859656	12.47505360763472	2.99702510362356
Cu	6.39941179189373	11.74405434737709	5.08098045509053
Cu	7.67419865466326	3.66183535606832	3.01079563873894
Cu	6.38953108805152	2.89248493389775	5.12250408285961
Cu	6.39501558385700	13.22853247899088	0.90055000000010
Cu	6.39686870777428	14.69395173394748	3.01613105360917
Cu	5.11837097368600	13.96053195654477	5.12543527112342
Cu	-3.82917738360190	13.22870957252048	0.90195000000005
Cu	-3.83007292750086	14.70010063254733	3.01447363707484
Cu	5.11674863105053	15.44220159981588	0.90370000000011
Cu	5.11504808642443	16.91220648526951	3.01644724208430
Cu	3.84243051811226	16.17468906485124	5.13549017090552
Cu	6.39583354385823	4.37350180863156	0.90404999999997
Cu	6.41333438696529	5.87380067019595	3.00932585301298
Cu	3.83940188321714	17.65569362711116	0.90370000000011
Cu	14.05713171393419	1.41423702443333	3.00941076604793
Cu	15.34273253135209	0.67784530876717	5.13219275361738
Cu	5.12322542765772	5.10695979883633	5.11167543095100
Cu	0.00531673982669	6.58752511651588	0.90370000000011
Cu	15.34196404851714	2.15930140721752	0.90299999999988
Cu	15.33588520651733	3.63809828622721	2.99303360666843
Cu	14.06916139553229	2.88344474263132	5.06173432644221
Cu	2.56079163400170	0.68420399115942	5.13255090757454
Cu	5.11664638607854	6.58699383592684	0.90229999999991
Cu	14.06349260571656	4.37226215392388	0.90089999999996
Cu	14.04956099713787	5.85077309408107	2.99691134398770
Cu	12.77561598255331	5.12295121751148	5.07689755562633
Cu	5.16605456229638	8.05472486582645	3.00496857961125
Cu	3.85256220436883	7.32260011061244	5.09195419530625
Cu	12.78543014290405	6.58982733240151	0.90159999999994
Cu	12.77731990353658	8.06140708515311	3.01269067063129
Cu	11.49999702529403	7.32339379771957	5.12009610800585

Cu	-6.38468883924485	17.65640200122992	0.90335000000000
Cu	3.84017969244467	1.42354010546447	3.01849362452267
Cu	11.50695870010339	8.80491320146391	0.90825000000006
Cu	11.48950822044525	10.25954606769414	3.02148341626721
Cu	10.22284675987658	9.52504411575760	5.12156457605413
Cu	3.83919739324809	8.80172551792992	0.90404999999997
Cu	3.85855595176044	10.26640770300189	3.00624596749411
Cu	10.23247481218681	11.01238404875052	0.90895000000003
Cu	10.23927250118141	12.49234726219921	2.99956062183290
Cu	8.95544548009073	11.74563862525665	5.06556576202739
Cu	2.56550409693769	9.53696864993854	5.11458888924287
Cu	5.11578667951230	0.67935297569490	5.13561900935346
Cu	8.95277642942183	13.22782410487224	0.90229999999991
Cu	8.94994914614826	14.69544876247025	3.01497212230047
Cu	7.67380438467671	13.95671317166789	5.12597537665111
Cu	-3.83042983570821	11.74605161551789	5.13077804893399
Cu	2.56113493043560	11.01504045169548	0.90404999999997
Cu	7.67205559668686	15.44290997393452	0.90159999999994
Cu	7.67200569397032	16.91172902259725	3.01285925036195
Cu	6.39627226895257	16.17592459609604	5.12738371966087
Cu	2.56649682195109	12.48577103164040	3.01779823915867
Cu	1.28757843427749	11.75123864131286	5.13240710289490
Cu	6.39511782884163	17.65604781417048	0.90404999999997
Cu	16.61837213239517	1.42098293338478	3.01283252734148
Cu	17.89678770717005	0.67978448863403	5.12158384532412
Cu	2.56123717545758	2.16018687486588	0.90404999999997
Cu	-5.10865474255421	13.96321430820861	5.12861952810094
Cu	17.89808897412964	2.16000978133616	0.90404999999997
Cu	17.89990480684789	3.63606959322462	3.01165895988905
Cu	16.62118131377239	2.89887902732334	5.11239250302277
Cu	1.28358369260824	13.22888666605020	0.90335000000000
Cu	1.28651489537445	14.70312312718294	3.01449620512254
Cu	16.61992426632008	4.37314762157224	0.90335000000000
Cu	16.62278548895317	5.85492807212634	3.00254375836997
Cu	15.34614373092503	5.11520393743935	5.07754030540432
Cu	0.00954687765213	13.96349049975610	5.13055585668432
Cu	2.56929110104739	5.11135197738534	5.13024347710538
Cu	15.34237302849272	6.58734802298616	0.90019999999999
Cu	15.33787038158404	8.06175696693635	3.01383115080489
Cu	14.06160163277914	7.32487727586878	5.12432787374212
Cu	0.00846199811265	8.06217681935251	3.01746195146218
Cu	0.00552122979574	15.44255578687520	0.90370000000011
Cu	14.06400383068912	8.80084005028156	0.90159999999994
Cu	14.05528664189073	10.26997252934253	3.01977222153042
Cu	12.78450798786336	9.53216308880273	5.13673295792733
Cu	0.00950579743748	16.91897885122220	3.00377693726960
Cu	-1.27339354995898	16.17269460314976	5.12157784662184
Cu	12.78174932299860	11.01220695522093	0.90789999999995
Cu	12.78419586015908	12.48290325150145	3.01654389923257
Cu	11.50688264821575	11.74744153268053	5.12193967140408
Cu	5.11756659106419	2.16018687486588	0.90370000000011
Cu	11.50777666006714	13.22835538546116	0.90195000000005
Cu	11.50927017365180	14.70426855891860	3.01462538743261
Cu	10.22738246071768	13.95800756410100	5.12152573425873
Cu	-1.27274572301080	17.65587072064088	0.90335000000000
Cu	8.95516037199958	1.42923203086226	2.99853712803846
Cu	10.22910072727251	15.44273288040492	0.90125000000008
Cu	10.22857578187975	16.91445762740703	3.01699954483753

Cu	8.94779038879278	16.17522245481507	5.12848936126703
Cu	8.95062928447193	17.65604781417048	0.90370000000011
Cu	19.17556030229316	1.42208444926989	3.01565151305255
Cu	0.00473550611072	0.68276609907350	5.12983957295277
Cu	-1.27077342202837	7.32202188268892	5.12763482694357
Cu	0.00511224984508	2.16018687486588	0.90404999999997
Cu	0.01230143631181	3.63663258782258	3.01684744172563
Cu	-1.27081215272736	2.89947409532160	5.12263464146432
Cu	2.56133942044221	6.58770221004548	0.90370000000011
Cu	2.56757871798707	8.06387693227936	3.00515153725719
Cu	-1.27284796797029	4.37403308922048	0.90370000000011
Cu	-1.26557064511565	5.85036498678645	3.01798033232859
Cu	-2.55126549307784	5.11267163490725	5.13049545066010
Cu	-1.27203000800663	8.80172551792992	0.90299999999988
Cu	-1.27152127963852	10.27329246372364	3.01654274700159
Cu	-2.54968349081861	6.58663964886752	0.90125000000008
Cu	-2.55114489257091	8.06354870661624	3.01488187790852
Cu	-3.82977327724930	7.32150704919643	5.12969748576879
Cu	-2.55201435004925	9.53269144499272	5.13253377425078
Cu	5.11880683475153	3.63484160679503	3.00965521126483
Cu	-3.82846166861030	8.80154842440020	0.90089999999996
Cu	-3.83357513682402	10.27235257118036	3.01690074130535
Cu	-5.10524690273466	9.53484412406840	5.13766198396897
Cu	3.84067136108107	2.89662898304994	5.13338828584601
Cu	2.56821886035381	3.63987581342246	3.02024641149278
Cu	-5.10948923633635	11.01397789051752	0.90299999999988
Cu	-5.11059353904830	12.48391056677674	3.01745359839756
Cu	-6.38629957760280	11.74571304171157	5.13363488290923
Cu	1.29371993377291	7.32539686785569	5.12373241046097
Cu	3.83929963825765	4.37421018275020	0.90370000000011
Cu	-6.38632475918468	13.22729282428320	0.90195000000005
Cu	-6.38785951567752	14.70187297099857	3.01463057349314
Cu	-7.66187548533777	13.96360746632825	5.12934221093065
Cu	3.85966204017576	5.85468809364428	3.00883883693430
Cu	-7.66305803703581	15.44290997393452	0.90019999999999
Cu	-7.66268391203167	16.91943410176861	3.01639243167038
Cu	-8.94459875690214	16.17717639070663	5.12599138266951
Cu	-2.54988798082509	11.01574882581424	0.90125000000008
Cu	-2.55337928147013	12.48720501202407	3.01641349871810
Cu	-8.94163172483352	17.65711037534856	0.90229999999991
Cu	1.28639630118410	1.42460388227170	3.02067380391173
N	5.75147849520307	9.95860220411031	8.54214211166994
N	7.77427131900146	10.78481207061598	8.53264012722927
N	10.47745685431294	2.48664594891995	8.53439170059242
N	12.42893752694142	3.46244519427418	8.53779506159196
H	3.92345270475273	9.25781812031645	7.74973368590218
H	5.02397655463583	8.00839390247511	8.39760193218162
H	8.14677778137326	13.66924251393575	8.67140602988007
H	3.46727043919383	11.75901387848939	8.68978834988502
H	6.14444113755807	15.16008106720474	8.72089349827495
H	3.85465163477679	14.22517569904794	8.72882134985844
H	4.11785191775062	9.07034612438433	9.52492889587034
H	9.65905803000093	9.90562629205064	8.36357606859965
H	9.55904840177589	11.56953832855237	7.72087872023177
H	9.57306907988214	11.29575844670898	9.49515553004291
H	8.54119790547407	2.50411541705096	7.67368365519757
H	9.48648920075073	1.00495365512489	7.41812748329034
H	13.58385442236898	4.34102685302040	11.07217964006137

```
H   9.07532816501770   2.08242963645018   11.06332062057512
H   12.34149342402225   3.94602895293009   13.18900093563532
H   10.12851636439046   2.83721996096316   13.18455128109739
H   8.79069799259295   1.34922189529986   9.01845662049545
H   14.20194790666431   3.37288493378769   7.41162304613276
H   13.58212368313863   5.02880554724237   7.69548201611827
H   14.35732538353028   4.10904122886401   9.02305129643317
C   7.07321631861591   9.61117084151543   8.38328915652537
C   5.60663081084684   11.33959742421922   8.67147259005120
C   6.91172304967611   11.87272566884290   8.66545730235481
C   6.01189387351810   14.07813235895707   8.73170604258514
C   4.63400487218170   9.01911375740942   8.55532101650904
C   7.14069892804033   13.25215041651929   8.70376226905662
C   4.70660117295877   13.54516312057363   8.73630202235391
C   4.47808357723757   12.16503729046145   8.71416750283918
C   9.22913186874062   10.89887941540107   8.52891985989631
C   11.48488160330412   2.91421829513922   7.69682343772192
C   10.76114399393886   2.78298102538855   9.86834729676300
C   12.01762730200354   3.41175535371327   9.87064767318924
C   11.90942177358904   3.62215014786834   12.24330285015238
C   9.25265294537954   1.80284025208711   8.13518994866786
C   12.61515972994908   3.84553572305198   11.05933461105753
C   10.64975413032075   2.99115505096212   12.24085018373034
C   10.05209850806690   2.56188396884327   11.05428590295916
C   13.71205910521837   4.03161919994761   8.14145161224874
```

Species from **Figure S100 d)**

```
234
Generated with con2xyz
Cu   10.22868310745960   0.68210151038097   5.13603362604442
Cu   1.28348144762368   8.80137133087048   0.90370000000011
Cu   1.28618358548897   10.27694122925838   3.01402260386554
Cu   10.22981644228912   2.15983268780656   0.90370000000011
Cu   10.22711319043871   3.63445454185888   2.99867173802472
Cu   8.94890918618233   2.89983365012614   5.10736769260792
Cu   0.00374315542355   9.54163442834902   5.13965125309924
Cu   1.28549253705952   2.89997931175686   5.13313472303485
Cu   8.95154948948265   4.37350180863156   0.90265000000002
Cu   8.93026564776408   5.89422503151029   3.01003480249837
Cu   7.66792462477840   5.11669357074617   5.08304659325597
Cu   -5.10580841645604   15.44273288040492   0.90125000000008
Cu   0.00582796479926   11.01539463875492   0.90335000000000
Cu   7.67348702667015   6.58787930357520   0.90335000000000
Cu   8.92900493950832   7.33013637804817   5.20422689352959
Cu   6.40980487748457   7.33541751166284   5.15961532098322
Cu   0.00662755534031   12.48918026799318   3.01894677332300
Cu   -1.27361803213745   11.75114365315121   5.13296140773617
Cu   6.39562905385170   8.80119423734088   0.90335000000000
Cu   6.42711669335552   10.23138281752486   3.01539712899724
Cu   5.11843876523526   9.53353070975626   5.07386520319874
Cu   -5.10981624426461   16.91877113622861   3.01886196101622
Cu   -6.39023539299284   16.18011348989139   5.13676392143786
Cu   5.11715761105111   11.01486335816588   0.90265000000002
Cu   5.11522301405277   12.48667197227952   3.00391973933951
Cu   3.83794386719527   11.75359630730384   5.11750785132909
Cu   -1.27243898800736   13.22906375957992   0.90370000000011
Cu   -1.27478694289273   14.70256683205186   3.01806846321622
Cu   3.83889065824457   13.22888666605020   0.90370000000011
```

Cu	3.83719592947707	14.70418168228374	3.01783089877487
Cu	2.55567900811462	13.96857839362137	5.13454351156068
Cu	-2.55300006008306	13.96633015802070	5.12675122592632
Cu	2.56093044042913	15.44237869334548	0.90404999999997
Cu	2.56020263634038	16.91921582597506	3.01825278654486
Cu	1.28468350355835	16.18270384231596	5.13890842475725
Cu	-2.55019471582868	15.44255578687520	0.90370000000011
Cu	1.28307246761067	17.65569362711116	0.90404999999997
Cu	11.50614312914514	1.42248879551714	3.02000645862966
Cu	12.78421874304067	0.68709035673228	5.13736858164064
Cu	-2.55203859014415	16.91814865034942	3.01762707283575
Cu	-3.83224606831343	16.18073256825455	5.13406603062241
Cu	12.78563463291059	2.15965559427684	0.90370000000011
Cu	12.77943652544277	3.64147170068337	3.01330747851591
Cu	11.50295066529679	2.90246177864673	5.12623101206270
Cu	1.28317471264508	4.37385599569088	0.90544999999992
Cu	11.50757217009817	4.37367890216116	0.90125000000008
Cu	11.49442567555066	5.85983323934159	2.99430844703398
Cu	10.22341806031726	5.11167870842912	5.06764878246195
Cu	-3.82825717864111	17.65657909475952	0.90335000000000
Cu	6.39624430837274	1.42481727845683	3.01204283392251
Cu	10.22899848230045	6.58929605181247	0.90335000000000
Cu	10.18548460923907	8.05522337702122	3.01885582861875
Cu	7.73615507914407	7.96136040219276	7.68364167555973
Cu	7.67037131924416	0.68354564273375	5.12946555165059
Cu	1.28824021031786	5.85264203211515	3.01978337166276
Cu	8.95134499947611	8.80119423734088	0.90440000000008
Cu	8.92273266157165	10.23089594918829	3.02303273241550
Cu	7.66870478443779	9.50957774952008	5.23087755868728
Cu	0.00744323000039	5.11389163637337	5.13295642679507
Cu	7.67379376167375	2.16000978133616	0.90370000000011
Cu	7.67522519160704	11.01362370345820	0.90404999999997
Cu	7.67185809176554	12.47176959227684	2.99247806217617
Cu	6.39604060856415	11.74296305227509	5.07542231313764
Cu	7.67226036407196	3.65704446968496	3.00346725689134
Cu	6.38905084654274	2.89515156241135	5.10927350179328
Cu	6.39501558385700	13.22853247899088	0.90055000000010
Cu	6.39639144778903	14.69495056590933	3.01608694228938
Cu	5.11425932503692	13.96434865827414	5.12070230218736
Cu	-3.82917738360190	13.22870957252048	0.90195000000005
Cu	-3.83044509159136	14.70222599159705	3.01759585540011
Cu	5.11674863105053	15.44220159981588	0.90370000000011
Cu	5.11640330972087	16.91533474591567	3.01791434921344
Cu	3.83828928111669	16.18143301532485	5.13248769707421
Cu	6.39583354385823	4.37350180863156	0.90404999999997
Cu	6.40898147586941	5.88690082431225	3.00199712754103
Cu	3.83940188321714	17.65569362711116	0.90370000000011
Cu	14.06109649338083	1.42375333572061	3.01996512709025
Cu	15.33857902313554	0.68420591153644	5.13588165354675
Cu	5.11678771527641	5.11625193171423	5.07009380636251
Cu	0.00531673982669	6.58752511651588	0.90370000000011
Cu	15.34196404851714	2.15930140721752	0.90299999999988
Cu	15.33662640356060	3.63893049505393	3.01630213864074
Cu	14.05842971672429	2.90085076902697	5.13349656958767
Cu	2.56049545489185	0.68577713822622	5.13533422132595
Cu	5.11664638607854	6.58699383592684	0.90229999999991
Cu	14.06349260571656	4.37226215392388	0.90089999999996
Cu	14.05769997205913	5.85391181037993	3.01244313430710

Cu	12.77863548277059	5.11472703682500	5.11572551683417
Cu	5.15134461767602	8.05435029782995	3.00286532081799
Cu	3.84624770785256	7.32640050554592	5.09527483620408
Cu	12.78543014290405	6.58982733240151	0.90159999999994
Cu	12.78297822597139	8.06694319624448	3.00776563376303
Cu	11.49714557754623	7.32817842485580	5.08850423010186
Cu	-6.38468883924485	17.65640200122992	0.90335000000000
Cu	3.83840699462618	1.42118966110510	3.01895337585127
Cu	11.50695870010339	8.80491320146391	0.90825000000006
Cu	11.48435382099714	10.26216680149184	3.02067735240091
Cu	10.21878397609589	9.52457786149564	5.12124617645415
Cu	3.83919739324809	8.80172551792992	0.90404999999997
Cu	3.85655482432307	10.26737218384823	3.00166118640402
Cu	10.23247481218681	11.01238404875052	0.90895000000003
Cu	10.23223350403769	12.48536314418664	2.99945175786655
Cu	8.94899369731094	11.74216393529636	5.06586625241968
Cu	2.55714645236595	9.53935150147392	5.11574935218716
Cu	5.11546225628952	0.68224196790127	5.13958974968828
Cu	8.95277642942183	13.22782410487224	0.90229999999991
Cu	8.95062146124013	14.69482618751730	3.01375202113393
Cu	7.67365112152596	13.95633117607657	5.11985770379916
Cu	-3.83149764589629	11.74996319195653	5.13291295698881
Cu	2.56113493043560	11.01504045169548	0.90404999999997
Cu	7.67205559668686	15.44290997393452	0.90159999999994
Cu	7.67419103127657	16.91254016191116	3.01543987547544
Cu	6.39481702967765	16.17896166955379	5.12672482973187
Cu	2.56369025882118	12.48747227292613	3.01549297816419
Cu	1.28334594534052	11.75265218098201	5.13628212655386
Cu	6.39511782884163	17.65604781417048	0.90404999999997
Cu	16.62059525968475	1.42276894664461	3.01768221300216
Cu	17.89675337335444	0.68101949459133	5.12809731174386
Cu	2.56123717545758	2.16018687486588	0.90404999999997
Cu	-5.11160023566376	13.96647002113163	5.13302652293116
Cu	17.89808897412964	2.16000978133616	0.90404999999997
Cu	17.89795932246980	3.63579120355400	3.01787928160250
Cu	16.61560500270533	2.89931071967174	5.12683353107272
Cu	1.28378818260220	13.22888666605020	0.90335000000000
Cu	1.28396754024100	14.70493910596321	3.01768379169181
Cu	16.61992426632008	4.37314762157224	0.90335000000000
Cu	16.61707752959117	5.85146009472290	3.01837582285864
Cu	15.33586924561223	5.11300501089999	5.13195542200902
Cu	0.00567665629597	13.96755922120846	5.13382554072847
Cu	2.56701460920279	5.11615658590198	5.13008629226706
Cu	15.34237302849272	6.58734802298616	0.90019999999999
Cu	15.33959333352293	8.06408631966885	3.01833340805636
Cu	14.05549499817403	7.32581552844504	5.13063993388331
Cu	0.00573241735790	8.06413633618407	3.02103870984586
Cu	0.00552122979574	15.44255578687520	0.90370000000011
Cu	14.06400383068912	8.80084005028156	0.90159999999994
Cu	14.05491077346697	10.27431036187124	3.02060890659941
Cu	12.78139241760318	9.53496573467707	5.13729595706566
Cu	0.00526843761243	16.91933264664403	3.01870229238090
Cu	-1.27309986287742	16.18124034105011	5.13354428316976
Cu	12.78174932299860	11.01220695522093	0.90789999999995
Cu	12.78139233577614	12.48490708268858	3.01674083026668
Cu	11.50386833161298	11.75104497774955	5.12107980003478
Cu	5.11756659106419	2.16018687486588	0.90370000000011
Cu	11.50777666006714	13.22835538546116	0.90195000000005

Cu	11.50785552807144	14.70231152025678	3.01598743728101
Cu	10.22656888209170	13.95839636132432	5.12549284936629
Cu	-1.27274572301080	17.65587072064088	0.90335000000000
Cu	8.94842106524429	1.42590469913191	3.01552430139074
Cu	10.22910072727251	15.44273288040492	0.90125000000008
Cu	10.22948817743953	16.91416724373341	3.01811490852378
Cu	8.94967297606726	16.17476256445765	5.13090522450561
Cu	8.95062928447193	17.65604781417048	0.90370000000011
Cu	19.17503889861479	1.42271889327838	3.01798977134867
Cu	0.00523410588034	0.68224225196341	5.13372045172099
Cu	-1.27142028379726	7.32507168702667	5.13481624867673
Cu	0.00511224984508	2.16018687486588	0.90404999999997
Cu	0.00872011587998	3.63855413420097	3.01909697902208
Cu	-1.27239498714115	2.89906877416660	5.12795667850221
Cu	2.56133942044221	6.58770221004548	0.90370000000011
Cu	2.56135678435606	8.06614096898747	3.00650453999969
Cu	-1.27284796797029	4.37403308922048	0.90370000000011
Cu	-1.27138871908286	5.85158864221715	3.02008745698882
Cu	-2.55280099972711	5.11335289003642	5.13451310130065
Cu	-1.27203000800663	8.80172551792992	0.90299999999988
Cu	-1.27264178684351	10.27454710156233	3.01872905707509
Cu	-2.54968349081861	6.58663964886752	0.90125000000008
Cu	-2.55197728370446	8.06292935983833	3.01835400272060
Cu	-3.83321933977437	7.32444512310368	5.13387438626683
Cu	-2.55266991417531	9.53604393995179	5.13656746768096
Cu	5.11686773701679	3.63386748262261	2.99963851759732
Cu	-3.82846166861030	8.80154842440020	0.90089999999996
Cu	-3.83284724825776	10.27393570777247	3.01854182398621
Cu	-5.10914980137418	9.53898466579466	5.13928181214656
Cu	3.84248134291088	2.90033069769832	5.13036945714828
Cu	2.56522895420522	3.64067047649928	3.01956683612206
Cu	-5.10948923633635	11.01397789051752	0.90299999999988
Cu	-5.11239599567077	12.48710880368501	3.01909063571707
Cu	-6.38963469230537	11.75064112766039	5.13665166954406
Cu	1.28929656280869	7.32818301493332	5.13405221515612
Cu	3.83929963825765	4.37421018275020	0.90370000000011
Cu	-6.38632475918468	13.22729282428320	0.90195000000005
Cu	-6.38945741457341	14.70226311746262	3.01796068770302
Cu	-7.66546180536491	13.96486806292607	5.13923161124362
Cu	3.84762083075286	5.85746679565274	2.99605975501601
Cu	-7.66305803703581	15.44290997393452	0.90019999999999
Cu	-7.66463489240795	16.91738616338088	3.01877860673793
Cu	-8.94538984379969	16.17612161936319	5.13434162707200
Cu	-2.54988798082509	11.01574882581424	0.90125000000008
Cu	-2.55319230802880	12.49004721795713	3.01757701340600
Cu	-8.94163172483352	17.65711037534856	0.90229999999991
Cu	1.28481165464430	1.42311872461218	3.01999921113051
N	5.79421816930748	10.09790930612887	8.39711918696021
N	7.83389489883175	10.87818864170554	8.38052007865605
N	7.39690219817769	5.08142782526206	8.35169656586146
N	9.50633459841031	5.64566109503579	8.39513445676091
H	3.91282152112505	9.43278816375610	7.70214115890499
H	5.01028217851526	8.16844334739793	8.31773644991980
H	8.26741639937392	13.75633180105564	8.56091378669496
H	3.55016904478495	11.94120348874557	8.58162077974753
H	6.29704350759211	15.28594217729442	8.65561342647697
H	3.98783647604502	14.39906017447456	8.66599514375441
H	4.18791900264895	9.26836621175125	9.46986226412452

```
H   9.70596718552421   9.98333640874369   8.15226915481601
H   9.66142069912263   11.71653158760001   7.74194218482817
H   9.60904324859183   11.20691301315803   9.46375355921031
H   5.59651703577571   5.21396335359830   9.44727638370337
H   5.61711643230417   6.06065712467129   7.85973473305145
H   11.53705571944866   3.57468144802013   8.68048985652168
H   6.65535563447878   2.27371320724920   8.61594797723460
H   10.83664496621888   1.18373769863203   8.83512614282523
H   8.44565561562488   0.54771088317564   8.80518833111939
H   5.49955910496401   4.29114923543710   7.90854829463263
H   10.50870599776689   7.46039890058027   8.15048694660328
H   11.11392699167133   6.44035687108548   9.49943299526770
H   11.49296878087050   6.01896342462317   7.79423406070984
C   7.10795235787557   9.71813626421802   8.24087994719149
C   5.68049267197885   11.48103991124205   8.53090790598480
C   6.99603186423925   11.98506895722820   8.52307306823331
C   6.14150754331319   14.20718757930878   8.64069384719765
C   4.65526230658328   9.19034672236520   8.47683210156303
C   7.25357482760214   13.35847437470294   8.58568223117564
C   4.82580011315217   13.70213294938997   8.64649187245461
C   4.56911369965321   12.32694251265833   8.59884109591815
C   9.28964324526166   10.95392903227988   8.44153268946153
C   8.24419064865675   6.15789921587010   8.21263819799485
C   8.10990839836925   3.89778156460984   8.55061948151972
C   9.47057517726369   4.26071139675177   8.57251572127894
C   10.07879722232748   1.96483184451097   8.77517462214940
C   5.94077146285645   5.16626913317253   8.40269686151540
C   10.48237255194373   3.30206465605516   8.68844375091757
C   8.71727216477281   1.60234106760435   8.75751188623076
C   7.70526390001266   2.56255729091486   8.65015019026987
C   10.73120921587640   6.43641130392852   8.46781186045516
```

Species **[Cu(BIMe)]**

```
22
Cu-NHC-2_1_complex_neutral_opt.log
Energy: -1316738.3384431
N         -0.09109        -1.07648         0.04078
H         -0.19920        -2.95967         0.94695
H         -1.64325        -2.42844         0.05146
H          2.42241         2.50546        -0.01459
C         -0.90981         0.00000         0.06028
C          1.23702        -0.69802         0.00945
C          1.23702         0.69802         0.00945
N         -0.09109         1.07649         0.04078
H          2.42241        -2.50546        -0.01459
C          3.60050         0.69827        -0.03890
H          4.54579         1.22730        -0.05757
H          4.54578        -1.22731        -0.05757
C         -0.55365        -2.44218         0.05269
H         -0.19933        -2.97302        -0.83370
C          2.41841         1.42231        -0.01484
C          3.60050        -0.69827        -0.03890
C          2.41841        -1.42231        -0.01484
C         -0.55364         2.44218         0.05269
```

H	-1.64325	2.42844	0.05146
H	-0.19919	2.95967	0.94695
H	-0.19933	2.97302	-0.83370
Cu	-2.88090	-0.00000	-0.04202

Species **[Cu(BIMe)]$^{+}$**

22
Cu-NHC-2_1_complex_positive.log
Energy: -1316630.6036579

N	0.12037	-1.08275	0.00000
H	0.19266	-2.97266	-0.89029
H	1.64804	-2.48566	-0.00001
H	-2.39619	2.51021	-0.00000
C	0.91669	0.00000	0.00000
C	-1.20876	-0.69655	-0.00000
C	-1.20876	0.69655	-0.00000
N	0.12036	1.08275	-0.00000
H	-2.39619	-2.51021	0.00001
C	-3.56404	0.70034	0.00000
H	-4.51077	1.22616	0.00000
H	-4.51077	-1.22616	0.00001
C	0.55939	-2.45971	-0.00001
H	0.19267	-2.97268	0.89026
C	-2.38867	1.42755	-0.00000
C	-3.56404	-0.70034	0.00000
C	-2.38867	-1.42755	0.00000
C	0.55939	2.45971	-0.00000
H	1.64804	2.48566	0.00001
H	0.19267	2.97267	-0.89028
H	0.19266	2.97267	0.89027
Cu	2.82023	-0.00000	0.00000

Species **[Cu(BIMe)$_2$]**

43
Cu-NHC-2_2_complex_neutral.log
Energy: -1604224.9237036

N	-2.72753	-1.05489	0.24533
H	-2.38039	-3.04879	-0.31412
H	-1.23056	-2.33157	0.84172
H	-5.23603	2.45227	-0.51597
C	-1.88128	0.00001	0.00000
C	-4.04718	-0.68416	0.15121
C	-4.04718	0.68415	-0.15121
N	-2.72754	1.05490	-0.24533
H	-5.23602	-2.45229	0.51597
C	-6.42074	0.67912	-0.14468

H	-7.36518	1.19908	-0.25402
H	-7.36517	-1.19911	0.25403
C	-2.27976	-2.38305	0.54872
H	-2.85624	-2.79511	1.38047
C	-5.23025	1.39163	-0.29711
C	-6.42073	-0.67914	0.14469
C	-5.23024	-1.39164	0.29712
C	-2.27978	2.38306	-0.54873
H	-1.23057	2.33159	-0.84169
H	-2.85624	2.79509	-1.38050
H	-2.38045	3.04881	0.31410
N	2.72753	-1.05489	-0.24533
H	2.38041	-3.04880	0.31411
H	1.23056	-2.33157	-0.84171
H	5.23604	2.45227	0.51597
C	1.88128	0.00001	-0.00000
C	4.04718	-0.68416	-0.15121
C	4.04718	0.68415	0.15121
N	2.72754	1.05490	0.24533
H	5.23602	-2.45229	-0.51597
C	6.42074	0.67911	0.14469
H	7.36518	1.19908	0.25403
H	7.36517	-1.19911	-0.25402
C	2.27976	-2.38305	-0.54872
H	2.85623	-2.79510	-1.38049
C	5.23025	1.39163	0.29712
C	6.42073	-0.67914	-0.14468
C	5.23024	-1.39165	-0.29711
C	2.27978	2.38306	0.54872
H	1.23057	2.33160	0.84169
H	2.85624	2.79510	1.38050
H	2.38045	3.04881	-0.31410
Cu	0.00000	0.00001	-0.00000

Species **$[Cu(BIMe)_2]^+$**
43
Cu-NHC-2_2_complex_positive.log
Energy: -1604150.0887378

N	-2.73731	-0.76276	0.76238
H	-2.65837	-2.72898	1.47016
H	-1.20522	-1.74516	1.74432
H	-5.25365	1.77404	-1.77314
C	-1.92686	0.00001	-0.00001
C	-4.06628	-0.49272	0.49247
C	-4.06629	0.49272	-0.49247
N	-2.73732	0.76277	-0.76238
H	-5.25362	-1.77406	1.77315
C	-6.42468	0.49481	-0.49456

H	-7.37093	0.86745	-0.86700
H	-7.37091	-0.86748	0.86704
C	-2.29374	-1.73446	1.73357
H	-2.65843	-1.47159	2.72822
C	-5.24687	1.00817	-1.00765
C	-6.42467	-0.49484	0.49458
C	-5.24685	-1.00819	1.00767
C	-2.29377	1.73446	-1.73358
H	-1.20525	1.74517	-1.74434
H	-2.65848	1.47160	-2.72822
H	-2.65840	2.72899	-1.47016
N	2.73732	-0.76241	-0.76274
H	2.65844	-2.72828	-1.47149
H	1.20524	-1.74438	-1.74511
H	5.25364	1.77321	1.77398
C	1.92686	0.00001	-0.00000
C	4.06629	-0.49249	-0.49270
C	4.06629	0.49249	0.49270
N	2.73732	0.76241	0.76273
H	5.25363	-1.77322	-1.77398
C	6.42468	0.49458	0.49481
H	7.37092	0.86704	0.86744
H	7.37092	-0.86707	-0.86742
C	2.29376	-1.73364	-1.73440
H	2.65840	-1.47027	-2.72893
C	5.24686	1.00770	1.00814
C	6.42468	-0.49460	-0.49480
C	5.24686	-1.00771	-1.00813
C	2.29376	1.73365	1.73439
H	1.20524	1.74440	1.74509
H	2.65839	1.47028	2.72892
H	2.65845	2.72829	1.47148
Cu	-0.00000	0.00001	-0.00001

Supplementary References

[1] C. Gutheil, G. Roß, S. Amirjalayer, B. Mo, A. H. Schäfer, N. L. Doltsinis, B. Braunschweig, F. Glorius, *ACS Nano* **2024**, *18*, 3043.

[2] J. Ren, M. Koy, H. Osthues, B. S. Lammers, C. Gutheil, M. Nyenhuis, Q. Zheng, Y. Xiao, L. Huang, A. Nalop et al., *Nature Chemistry* **2023**, *15*, 1737.

[3] A. Bakker, A. Timmer, E. Kolodzeiski, M. Freitag, H. Y. Gao, H. Mönig, S. Amirjalayer, F. Glorius, H. Fuchs, *Journal of the American Chemical Society* **2018**, *140*, 11889.

[4] M. Franz, A. Das, S. Chandola, M. Kubicki, M. Das, A. Sette, D. Corona, M. Palummo, L. Chiodo, R. Schöder et al., *Angewandte Chemie (International ed. in English)* **2025**, *64*, e202511094.

[5] M. Schedler, R. Fröhlich, C.-G. Daniliuc, F. Glorius, *European Journal of Organic Chemistry* **2012**, *2012*, 4164.

[6] V. Jurkauskas, J. P. Sadighi, S. L. Buchwald, *Organic Letters* **2003**, *5*, 2417.

[7] B. Liu, X. Ma, F. Wu, W. Chen, *Dalton Transactions* **2015**, *44*, 1836.

[8] D. Li, T. Ollevier, *Journal of Organometallic Chemistry* **2020**, *906*, 121025.

[9] R. Das, M. Banerjee, R. K. Rai, R. Karri, G. Roy, *Organic & Biomolecular Chemistry* **2018**, *16*, 4243.

[10] O. D. Thomas, K. J. W. Y. Soo, T. J. Peckham, M. P. Kulkarni, S. Holdcroft, *Polymer Chemistry* **2011**, *2*, 1641.

[11] T. O. Ely, C. Amiens, B. Chaudret, E. Snoeck, M. Verelst, M. Respaud, J.-M. Broto, *Chemistry of Materials* **1999**, *11*, 526.

[12] A. Jablonski, *Surface Science Reports* **2002**, *47*, 33.

[13] H. K. Kim, A. S. Hyla, P. Winget, H. Li, C. M. Wyss, A. J. Jordan, F. A. Larrain, J. P. Sadighi, C. Fuentes-Hernandez, B. Kippelen et al., *Chemistry of Materials* **2017**, *29*, 3403.

[14] M. P. Seah, W. A. Dench, *Surface & Interface Analysis* **1979**, *1*, 2.

[15] S. Zhao, Y. Li, Y. Wang, Z. Ma, X. Huang, *Fuel* **2019**, *244*, 78.

[16] E. Goodwin, M. Davies, M. Bakiro, E. Desroche, F. Tumino, M. Aloisio, C. M. Crudden, P. J. Ragogna, M. Karttunen, S. T. Barry, *ACS Nano* **2025**, *19*, 15617.

[17] E. Amit, L. Dery, S. Dery, S. Kim, A. Roy, Q. Hu, V. Gutkin, H. Eisenberg, T. Stein, D. Mandler et al., *Nature Communications* **2020**, *11*, 5714.

[18] G. Lovat, E. A. Doud, D. Lu, G. Kladnik, M. S. Inkpen, M. L. Steigerwald, D. Cvetko, M. S. Hybertsen, A. Morgante, X. Roy et al., *Chemical Science* **2019**, *10*, 930.

[19] S. Stoll, A. Schweiger, *Journal of Magnetic Resonance* **2006**, *178*, 42.

[20] J. D. Hunter, *Computing in Science & Engineering* **2007**, *9*, 90.

[21] U. B. Steiner, W. R. Caseri, U. W. Suter, *Langmuir* **1992**, *8*, 2771.

[22] C. M. Crudden, J. H. Horton, I. I. Ebralidze, O. V. Zenkina, A. B. McLean, B. Drevniok, Z. She, H.-B. Kraatz, N. J. Mosey, T. Seki et al., *Nature Chemistry* **2014**, *6*, 409.

[23] A. J. Veinot, A. Al-Rashed, J. D. Padmos, I. Singh, D. S. Lee, M. R. Narouz, P. A. Lummis, C. J. Baddeley, C. M. Crudden, J. H. Horton, *Chemistry - A European Journal* **2020**, *26*, 11431.

[24] A. Inayeh, R. R. K. Groome, I. Singh, A. J. Veinot, F. C. de Lima, R. H. Miwa, C. M. Crudden, A. B. McLean, *Nature Communications* **2021**, *12*, 4034.

[25] C. R. Larrea, C. J. Baddeley, M. R. Narouz, N. J. Mosey, J. H. Horton, C. M. Crudden, *Chemphyschem : a European journal of chemical physics and physical chemistry* **2017**, *18*, 3536.

[26] R. B. Chevalier, J. Pantano, M. K. Kiesewetter, J. R. Dwyer, *Beilstein Journal of Nanotechnology* **2023**, *14*, 865.

[27] Bruker AXS, *APEX4 Version 2021.4-0, SAINT Version 8.40 B and SADABS Bruker AXS Area Detector Scaling and Absorption Correction Version 2016/2*, **2021**, Bruker AXS Inc., Madison, Wisconsin, USA.
[28] G. M. Sheldrick, *Foundations of Crystallography* **2015**, *71*, 3.
[29] G. M. Sheldrick, *Acta Crystallographica. Section C, Structural Chemistry* **2015**, *71*, 3.
[30] Bruker AXS, *XP – Interactive Molecular Graphics, Version 5.1*, **1998**, Bruker AXS Inc., Madison, Wisconsin, USA.
[31] G. Kresse, J. Furthmüller, *Computational Materials Science* **1996**, *6*, 15.
[32] G. Kresse, J. Furthmüller, *Physical review. B, Condensed matter* **1996**, *54*, 11169.
[33] G. Kresse, D. Joubert, *Physical review. B, Condensed matter* **1999**, *59*, 1758.
[34] P. E. Blöchl, *Physical review. B, Condensed matter* **1994**, *50*, 17953.
[35] J. Klimeš, D. R. Bowler, A. Michaelides, *Journal of physics. Condensed matter : an Institute of Physics journal* **2010**, *22*, 22201.
[36] H. J. Monkhorst, J. D. Pack, *Phys. Rev. B* **1976**, *13*, 5188.
[37] M. Methfessel, A. T. Paxton, *Physical review. B, Condensed matter* **1989**, *40*, 3616.
[38] A. Jain, S. P. Ong, G. Hautier, W. Chen, W. D. Richards, S. Dacek, S. Cholia, D. Gunter, D. Skinner, G. Ceder et al., *APL Materials* **2013**, *1*.
[39] S. Maintz, V. L. Deringer, A. L. Tchougréeff, R. Dronskowski, *Journal of computational chemistry* **2016**, *37*, 1030.
[40] Gaussian 09, Revision D.01, M. J. Frisch, G. W. Trucks, H. B. Schlegel, G. E. Scuseria, M. A. Robb, J. R. Cheeseman, G. Scalmani, V. Barone, B. Mennucci, G. A. Petersson, H. Nakatsuji, M. Caricato, X. Li, H. P. Hratchian, A. F. Izmaylov, J. Bloino, G. Zheng, J. L. Sonnenberg, M. Hada, M. Ehara, K. Toyota, R. Fukuda, J. Hasegawa, M. Ishida, T. Nakajima, Y. Honda, O. Kitao, H. Nakai, T. Vreven, J. A. Montgomery, Jr.,J. E. Peralta, F. Ogliaro, M. Bearpark, J. J. Heyd, E. Brothers,K. N. Kudin, V. N. Staroverov, T. Keith, R. Kobayashi, J. Normand, K. Raghavachari, A. Rendell, J. C. Burant, S. S. Iyengar, J. Tomasi, M. Cossi, N. Rega, J. M. Millam, M. Klene, J. E. Knox, J. B. Cross, V. Bakken, C. Adamo, J. Jaramillo, R. Gomperts, R. E. Stratmann, O. Yazyev, A. J. Austin, R. Cammi, C. Pomelli, J. W. Ochterski, R. L. Martin, K. Morokuma, V. G. Zakrzewski, G. A. Voth, P. Salvador, J. J. Dannenberg, S. Dapprich, A. D. Daniels, O. Farkas, J. B. Foresman, J. V. Ortiz, J. Cioslowski, and D. J. Fox, *Gaussian, Inc., Wallingford CT* **2013**.
[41] C. Adamo, M. Cossi, V. Barone, *Journal of Molecular Structure: THEOCHEM* **1999**, *493*, 145.
[42] F. Weigend, *Physical chemistry chemical physics : PCCP* **2006**, *8*, 1057.
[43] F. Weigend, R. Ahlrichs, *Physical chemistry chemical physics : PCCP* **2005**, *7*, 3297.
[44] S. Tardio, P. J. Cumpson, *Surface & Interface Analysis* **2018**, *50*, 5.
[45] F. Weinhold, C. R. Landis, E. D. Glendening, *International Reviews in Physical Chemistry* **2016**, *35*, 399.
[46] T. Lu, F. Chen, *Journal of computational chemistry* **2012**, *33*, 580.
[47] T. Lu, *The Journal of chemical physics* **2024**, *161*.
[48] S. S. Batsanov, *Inorganic Materials* **2001**, *37*, 871.
